# Transcriptome analysis of floral bud development and function analysis of a novel *CO* gene in *Paeonia* × *lemoinei* ‘High Noon’

**DOI:** 10.1038/s41598-022-22195-z

**Published:** 2022-10-14

**Authors:** Yanting Chang, Wenbo Zhang, Yanjun Ma, Mengsi Xia, Keke Fan, Zehui Jiang, Tao Hu

**Affiliations:** Key Laboratory of Bamboo and Rattan Science and Technology, State Forestry Administration, Institute of Horticultural Flower and Landscape, International Center for Bamboo and Rattan, Futongdong Rd, Wang Jing, Chaoyang District, Beijing, 100102 China

**Keywords:** Transcriptomics, Plant breeding

## Abstract

*Paeonia* × *lemoinei* 'High Noon' is one of the most important cultivars in tree peony (*Paeonia* sect. Moutan), a traditional horticultural plant in China, with a re-blooming characteristic which was quite different from other cultivars. So, the genetic resources in 'High Noon' were incredibly valuable in flowering-time-modified molecular breeding in tree peony. However, the molecular mechanism underlying the floral bud formation of 'High Noon' was not clear yet. To explore the molecular mechanism in this process, the transcriptomes of three stages during floral bud development were deeply analyzed in this study. As a result, a total of 5816 differentially expressed genes (DEGs) were identified between the three developmental stages, and pathways including ''DNA replication'', ''metabolic pathways'', ''circadian rhythm'', and ''plant hormone signal transduction'' were mostly enriched in the functional enrichment and expression pattern analysis. Furthermore, a total of 584 genes related to the photoperiod pathway were further identified and a novel CO homolog gene *PlCO* was identified to be a stable hydrophilic protein, which contained both CCT domain and B-box domain. Over-expression of *PlCO* in *Arabidopsis* resulted in early flowering, which suggested a promotion role of flowering. The PlCO protein localized in nucleus and possessed a transcription activity ability, which implied that PlCO might function as a transcription factor. The transcriptome analysis revealed pathways involved in floral bud development in tree peony and provided new insight into the regulatory network underlying the floral bud development. The gene identification in 'High Noon' provided new valuable candidate genes for flowering-time-modified molecular breeding in tree peony.

## Introduction

Tree peony (*Paeonia* sect. Moutan) is one of the most famous horticultural plants in the world due to its large, fragrant, and colorful flowers. Tree peony originated in China and had a long history of cultivation. Since the Tang dynasty, tree peony was spread to Europe, America, and Japan, and the relevant cultivar groups emerged subsequently^1^. To date, more than 2000 cultivars were generated around the world and grouped into nine color-series and ten shape-series^2^. However, the characteristics of only 20-to-25-days-flowering-phase and the concentrated flowering period limit the commercial applications of tree peony. 'High Noon' (*Paeonia* × *lemoinei*) is an interspecies cultivar that has yellow color, semi-double and cup shape, and reblooming trait. It is one of the most popular cultivars of tree peony and contributes a reblooming trait for breeding. Studies about flowering-related genes and exploring the molecular mechanism of flowering are important for developing approaches to change the flowering time by manipulating flowering-related genes of tree peony thus extending the flowering period.

To date little is known about the genetic and molecular basis of the floral bud development for tree peony. Floral bud formation is one of the most important processes along with the life of plants, which contains a transition from vegetative to reproduction phase and a differential development stage of the meristems. In model plants like *Arabidopsis thaliana* and rice (*Oryza sativa*), the factors that influence the transition include photoperiod, vernalization, gibberellins (GA) signaling, age, and autonomous pathway^3–11^. The pathway that plants sense the change of daylength to influence floral transition was called photoperiod, which contains genes regulating circadian rhythm, like *Circadian Clock Associated 1* (*CCA1*), *Flavin-Binding*, *Kelch Repeat*, *F-Box 1* (*FKF1*), *Fiona1* (*FIO1*), *Far-Red Elongated Hypocotyl 1* (*FRY1*), *Nuclear Factor Y Subunit B8* (*NF*-*YB8*), *Nuclear Factor Y Subunit C* (*NF*-*YC*)^12–14^. Genes included in these pathways were identified in many plant species, and the regulatory network of floral transition and development was deeply researched in model plants. Previous studies showed that all these five pathways regulate flowering in tree peony and high amounts of gene information have been obtained^15–17^, including *PsFT*, *PsCRY1*, *PsSOC1*, and *PsGA20ox*^15,18,19^*.* For perennial woody plants, the flowering was along with bud dormancy and release, whose mechanism was researched in decades. The floral transition in blueberry (*Vaccinium ashei*) was regulated by phytohormone biosynthesis and signaling, transporter proteins, photosynthesis, anthocyanins biosynthesis^20^. In apple (*Malus domestica* Borkh.), epigenetic regulatory mechanism, sugar and cytokinin signaling pathway were thought to indicate the floral bud formation^21,22^. Also, the antioxidant system, secondary metabolism, cell cycle and division, cell wall metabolism, and carbohydrates metabolism were identified to regulate the floral bud dormancy and flowering in grape^23^. However, the molecular mechanism of floral formation in tree peony was still unclear.

*CONSTANS* (*CO*) is the output gene of photoperiod pathway. It regulates the flowering time through conducting the light signal to the flowering integrator *FLOWERING LOCUS T* (*FT*), which subsequently induces the floral meristem development from vegetative state to reproductive state^24^. *CO* genes belong to the BBX family which have both CO, CO-like, and TOC1 (CCT) domain and B-box domains. The CCT domain endows gene a DNA binding ability and the B-box domain participate in the protein–protein interaction^25^. The expression level of *CO* was regulated by circadian clock. The CO-FT module was conserved in plants, like *Arabidopsis*, rice, and soybean (*Glycine max*)^24,26^, which stand a core location in the flowering regulation. Yet, the function of *CO* genes was not identified and it limited the molecular mechanism analysis of photoperiod pathway in flowering time regulation of tree peony.

Transcriptome analysis has been frequently used to investigate the molecular mechanism of floral development in many plant species with no or poor open genome information. In previous studies, the unigenes were assembled de novo from the transcriptome of different cultivars, ignoring the variants between cultivars, and the DEGs were screened from buds at only one stage, which cannot represent the true expression pattern of genes during floral transition and floral development^27,28^. In consideration of the temporal and spatial pattern of gene expression, three stages in floral bud development were sequenced using full-length isoform sequencing and RNA-seq to generate a reference transcriptome and gene expression profile in our previous study^29^, though deep analysis was not done in the previous study. In this study, the regulation pathways underlying the floral bud development in tree peony were revealed, and candidate genes were screened out based on these data. A candidate *CO* homolog gene was identified and its function on flowering was investigated in *Arabidopsis*. Our research will provide a profound understanding of the floral bud development and provide genetic resources for the molecular breeding of flowering-time-changed in tree peony.

## Materials and methods

### Plant material and experiment procedures

The floral buds of 'High Noon' in the general first-time flowering were collected in the farm of Guanyu Peony Seedling Co., LTD in Heze, Shandong Province, China (E, 115° 32′ 30.7818″; N, 35° 20′ 4.794″). All the samples were obtained from 3 to 5 years-old plants every 10 days from August to November in 2018 with three biological replicates. The adjacent scales and leaves were removed, and the apical floral bud was transferred to liquid nitrogen for RNA isolation or to FAA solution for microscope observation. Total RNA was extracted by using a RNeasy Plant Mini kit (Qiagen, 74904, Germany) according to the manufacture's protocol. The quality of RNA was verified with RNase-free agarose gel electrophoresis, and the concentrate was measured using Nanodrop2000 (Thermo Fisher Scientific, Waltham, USA). All the plant material collection and research were permitted and complied with relevant institutional, national, and international guidelines and legislation.

### Microscope observation

Three floral buds were collected for each sample and fixed in FAA for 24 h at room temperature. After dehydrating in graded ethanol and xylene and embedded in paraffin, the samples were slit sectioned into 8-μm slices in Manual Rotary Microtome (Leica, RM2165, Germany). Then the slices were stained with Safranin and Fast Green and sealed using neutral gum. The microscope observation was conducted under Zeiss Primo Star (Germany).

### Data processing of RNA-seq

Total RNA was extracted from tree peony using Qiagen RNeasy Mini Kit (Qiagen, 74904, Germany). A total of 5 μg RNA samples were reverse transcribed to cDNA using SMARTer PCR cDNA Synthesis Kit (Clotech, 634925, USA). An equal amount of mRNA from floral buds in VS, DS and DCS was mixed and conducted to the ISO-seq, and three independent biological replicates in VS, DS and DCS were subjected in the RNA-seq. The sequencing method was described in detail in our previous study^29^. The referencing ISO-seq data and RNA-seq data were downloaded from the NCBI Sequence Read Archive (SRA) database (SRP212254) and Gene Expression Omnibus (GEO) (GSE133476). The raw data was cleaned using ng_QC with screening criteria of removing adapters, the reads with more than 10% unknown base, and the reads with Q20 < 50%. The acquired clean data was mapped to the ISO-seq data with bowtie2 (V2.3.4)^30^ with a parameter of “end-to-end” and “sensitive”. The read counts of each transcript were calculated with RSEM^31^ and transformed into FPKM value to represent gene expression level. The differential expression genes (DEGs) were analyzed with DESeq R package^32^ using read counts (fold change threshold ≥ 2 and *q*-value < 0.001).

### Gene ontology, KEGG pathway enrichment analysis and cluster analysis

The amino acid sequences of all the DEGs were subjected to an online enrichment website KOBAS 3.0 (http://kobas.cbi.pku.edu.cn/kobas3)^33^ to analyze the Gene Ontology (GO) annotation and KEGG pathway enrichment^34^ with a threshold of *p*-value < 0.01.

The expression cluster analysis was executed using Short Time-series Expression Miner (STEM, v1.3.11)^35^ with default parameters. The clusters with *p*-value < 0.001 were highlighted.

### Gene identification and cloning

A total of 626 flowering-related genes from *Arabidopsis*^36,37^ and rice^38^ (Table S4) were used to align with our reference transcriptome to screen genes associated with the flowering time and floral meristem development in tree peony. The alignment was conducted using local BLAST (v2.9.5) with a threshold of *e*-value < 1e−5 and identity > 50%.

The specific primers were designed to amplify the candidate gene from cDNA of tree peony. Sequence of *PlCO* gene was retrieved from transcript annotation of the cDNA libraries derived from floral buds in 'High Noon'. Full-length coding sequence of *PlCO* gene was amplified with KOD-Neo-Plus. The thermocycling conditions were as follows: denaturation of cDNA at 98 °C for 2 min, 32 cycles of 98 °C for 10 s, 60 °C for 10 s, and 72 °C for 5 s. PCR products were cloned into pMD-19T Vector (TAKARA) and sequenced at Introvigen Technologies Co, LTD.

Phylogenetic analysis was performed using MEGA 7 software^39^. Conserved motifs in protein sequence were identified using MEME tool^40^ (http://meme-suite.org/) with default parameters.

### Bioinformation analysis

The protein structure was predicted using SOPMA (SECONDARY STRUCTURE PREDICTION METHOD) software^41^ (https://npsa-prabi.ibcp.fr/cgi-bin/npsa_automat.pl?page=npsa_sopma.html) and SWISS-MODEL^42^ (https://www.swissmodel.expasy.org/). The trans-membrane prediction was predicted in TMHMM-2.0^43^ (http://www.cbs.dtu.dk/services/TMHMM/). The signal peptide and hydrophilicity were predicted in SignalP 5.0^44^ (https://services.healthtech.dtu.dk/service.php?SignalP-5.0) and online analysis website Protein-Sol^45^ (https://protein-sol.manchester.ac.uk/), respectively.

### Over-expression Arabidopsis plant obtaining

The ORF of *PlCO* was cloned to binary vector PHG and fused with GFP. The fused vector was introduced to the binary vector fusion were introduced to Agrobacterium strain GV3101 line and then introduced into the *A. thaliana* using the floral dip method. The primers and restriction sites are listed in Table S5. T1 generation transgenic seeds were grown and screened on the MS medium containing 50 mg/L hygromycin B. Seeds of the T3 generation were collected for follow-up experiments to confirm the results. The untransformed WT (Col-0) was used as the control. The plants were grown in a 22 h/2 h light/night conditions with a humidity of 75–80%. The flowering time and rosette leaf number were observed and counted with at least 10 plants in WT and transgenic plants.

### Protein interaction network

The protein interaction network was predicted in an online software STRING (V11.5) (https://cn.string-db.org/)^46^ using the amino acid sequence of PlCO with default parameters.

### Subcellular localization and transcription activity assay

The *A. strain* GV3101 line with fused 35S::PlCO-GFP vector was introduced to tobacco (*Nicotiana benthamiana*) leaves infiltrated into leaves and then the GFP fluorescence signal was observed after 2–7 days using the laser scanning confocal microscope (Zeiss LSM 510 Meta, Germany) in 488 nm. All transient expression assays were repeated at least three times.

The CDS sequence of the target gene was fused with pGBKT7 vector and expressed in yeast cell AH109 on plate of SD/-TRP. Growth and display were observed on SD/-Trp-HIS. The color change was observed on SD/-Trp-HIS/X-Gal plate.

## Results

### Transcriptome dynamics during floral bud development

**Figure 1:**
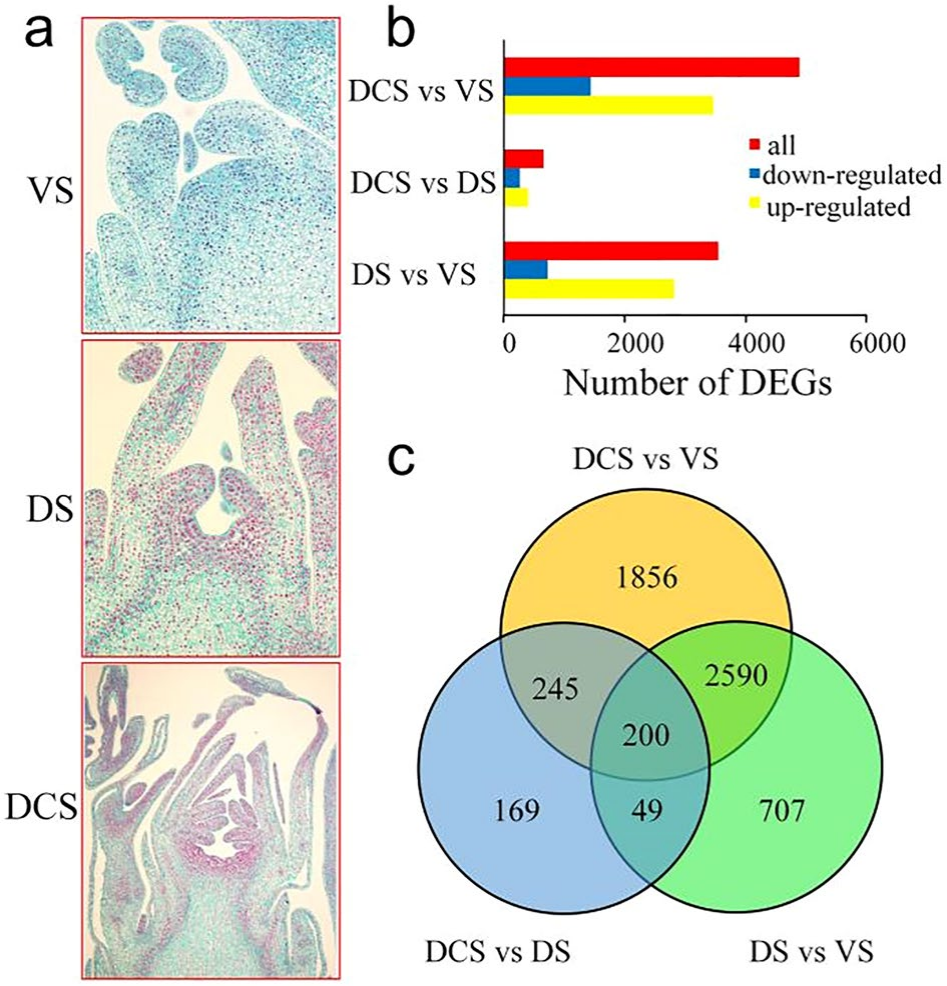
Analysis of the DEGs during the floral development in tree peony 'High Noon'. (**a**) The morphology of floral bud in 'High Noon'; VS, vegetative stage, DS, differentiation stage, DCS, differentiation completed stage; (**b**) The number of DEGs during bud development in 'High Noon'; (**c**) Venn diagram of DEGs involved in the phase change of floral development in tree peony 'High Noon'.

The floral meristem development is one of the most important prerequisites to the blooming of plants. To distinguish the different phases of the floral bud, the collected buds were sectioned into slices and observed on the microscope. The bud of vegetative stage (VS) had an uneven apical meristem with adjacent leaf primordium on both sides (Fig. 1a). After perceiving the environmental stimulation or endogenous cues, the bud apical became flat and the sepal primordium emerged, which suggested a transition from the vegetative phase to the differentiation stage (DS) (Fig. 1a). Then the primordium of petal, stamens and pistil differentiated rapidly in the second phase transition, which turned to the formation of a differentiation completed stage (DCS) (Fig. 1a). The completed differentiated meristem was ready for the dormancy and blooming.

To uncover the underlying mechanism of the floral bud formation, the transcriptome dynamics of the three stages were analyzed using the transcriptome data containing a reference isoform transcriptome sequencing data and an expression profile of the floral meristem development described in the previous study^29^. A total of 5816 DEGs were identified with a fold change threshold ≥ 2 and *q-*value ≤ 0.001 and ten genes were selected to verify the reliability of RNA-seq data (Table S1, Fig. S1). In detail, 2824 genes were up-regulated in DS compared with VS. Similarly, 3455 up-regulated DEGs were found in DCS compared with DS. A total of 403 DEGs were up-regulated in DCS compared with that of DS, respectively (Fig. 1b). A total of 1856 DEGs were special between DCS and VS (Fig. 1c). Meanwhile, 707 and 169 DEGs were filtered out only between DS and VS and between DCS and DS, which implied a stage-specific expressed pattern. Only 200 DEGs were shared among three comparisons, which means that the transitions between flower bud developmental stages were quite distinct.

### Functional enrichment analysis of DEGs

**Figure 2:**
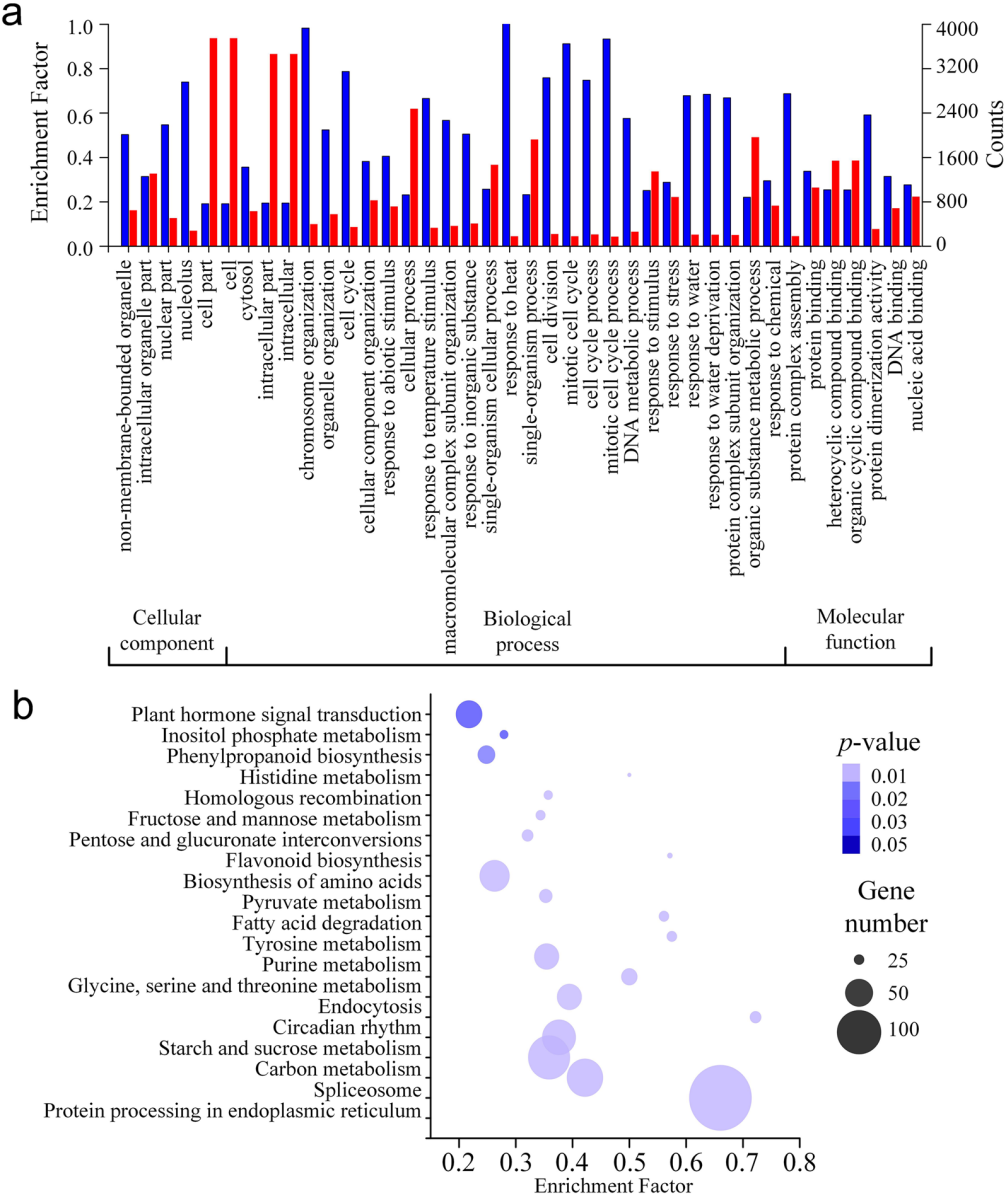
GO and KEGG pathway enrichment of DEGs during the floral development in tree peony 'High Noon'. (**a**) GO classification of DEGs during the floral development. The red columns represent the enriched DEG counts and the blue columns represent the enrichment factor; (**b**) KEGG pathways classification of DEGs during the floral development. The size of bubble represents gene number and the color represents *p*-value.

To get a comprehensive understanding of the regulation mechanism, the amino acid sequences of the 5816 DEGs were subjected to the function enrichment analysis for GO terms enrichment on a web server called KOBAS (version 3.0) with a *q*-value threshold of 0.01. As a result, a total of 3941 DEGs were enriched in 941 GO terms. The top 40 GO terms were then classed into three GO categories: cellular component, biological process and molecular function (Fig. 2a). Within cellular component category, the nucleolus was the most abundant term. While in the biological process, the largest highly represented terms were "response to heat", "chromosome organization", "cell cycle" and "cell division". In the molecular function, "protein dimerization activity" and "DNA binding" were most enriched (Fig. 2a).

To further determine the mechanism of flowering bud meristem development, all DEGs were annotated with KEGG pathways. As a result, a total of 992 DEGs were enriched to 49 KEGG pathways, including "plant hormone signal transduction", "fructose and mannose metabolism", "fatty acid degradation", and "circadian rhythm" (Table S2, Fig. 2a), which mostly related to metabolism and response to environment during the flowering bud development. To understand the regulation mechanism deeply, we analyzed the KEGG pathway enrichment between each two stages. As a result, the pathways including circadian rhythm and DNA replication were enriched and up-regulated in the transfer from VS to DS. Similarly, the plant hormone signal transduction pathway and circadian rhythm pathway were enriched in the up-regulated genes from DS to DCS. These two pathways were also enriched in the up-regulated genes of DCS to DS, implying an important role in the floral bud development.

### Gene expression profiling analysis during floral meristem development

**Figure 3:**
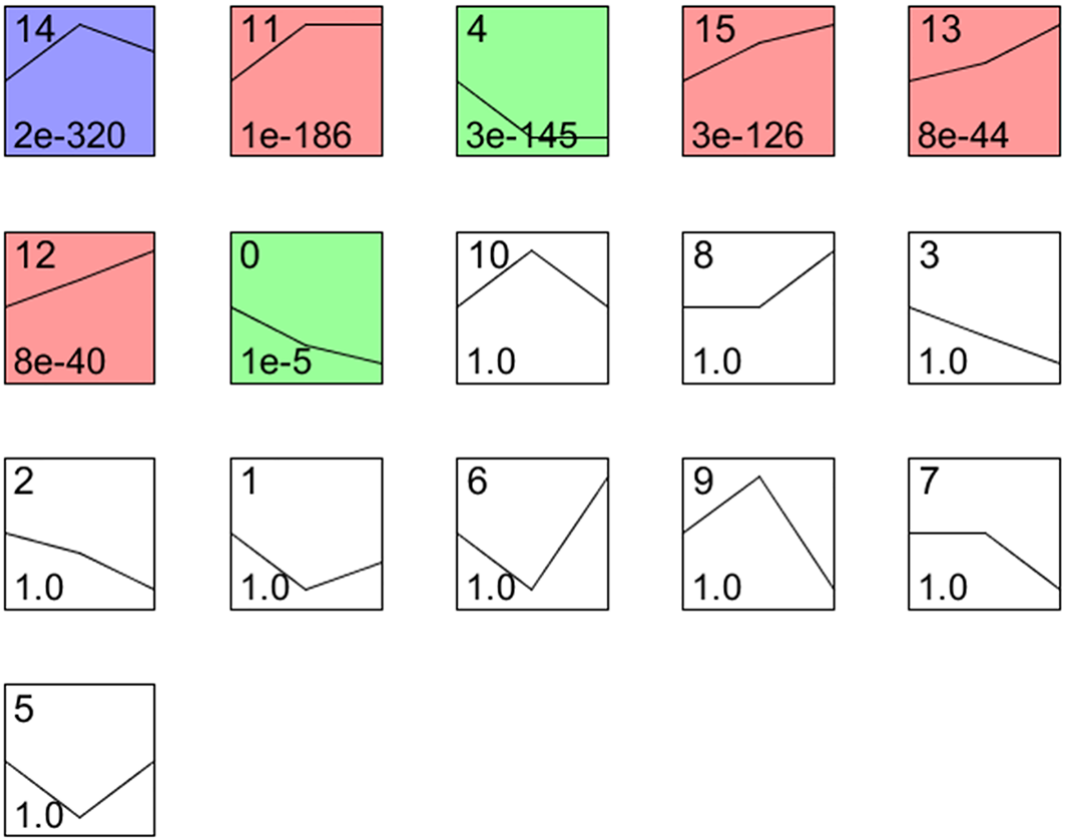
Profiles clustered during floral development in tree peony. The upper number represents profile name. The lower number represents *p*-value. The line represents the gene expression pattern of each profile. The colors in the boxes mean statistically significant with a *p*-value less than 0.001.

To get a better understanding of the DEGs between development stages, the expression trends of DEGs during the floral bud development of 'High Noon' were analyzed using STEM software (v1.3.11)^35^. According to the results, the DEGs were clustered into 16 profiles which showed different gene expression trends. Among them, profiles 0, 4, 11, 12, 13, 14, and 15 were statistically significant, with a p-value less than 0.001 (Fig. 3). The expression trends of the clusters suggested that the DEGs in a cluster regulated similar development processes.

Profile 14 had the most DEGs of 1373, which up-regulated in the fast differentiation stage (DS) and down-regulated in VS and DCS (Table 1). In this profile, genes were most enriched in DNA replication, mismatch repair, Cysteine and methionine metabolism, and homologous recombination. These pathways are most related to chromosome duplication occurred during cell division.

**Table 1:** The 7 significant expression profiles and their top 10 most significantly enriched functional pathways.

Profile	KEGG pathways	#Genes involved	Enrichment factor	*p*-value
Profile14	DNA replication	30	0.60	3.07E−21
	Mismatch repair	14	0.36	2.24E−08
	Phagosome	18	0.21	2.71E−07
	Pyrimidine metabolism	20	0.17	9.15E−07
	Cysteine and methionine metabolism	16	0.14	8.43E−05
	Photosynthesis—antenna proteins	6	0.27	8.73E−04
	Homologous recombination	9	0.16	1.41E−03
	Nucleotide excision repair	10	0.14	1.58E−03
	Base excision repair	7	0.16	4.41E−03
	Alanine, aspartate and glutamate metabolism	7	0.15	7.49E−03
Profile11	DNA replication	39	0.78	1.19E−35
	Metabolic pathways	143	0.07	1.63E−21
	Purine metabolism	26	0.16	6.09E−11
	Base excision repair	15	0.35	1.17E−10
	Pyrimidine metabolism	22	0.19	1.69E−10
	Biosynthesis of secondary metabolites	75	0.07	3.12E−10
	Nucleotide excision repair	17	0.25	6.49E−10
	Glycolysis/gluconeogenesis	19	0.16	2.75E−08
	Arginine and proline metabolism	13	0.25	7.00E−08
	Mismatch repair	11	0.28	2.17E−07
Profile4	Protein processing in endoplasmic reticulum	71	0.33	1.10E−50
	Spliceosome	30	0.16	3.83E−14
	Endocytosis	16	0.11	1.90E−06
	Carbon metabolism	19	0.07	7.49E−05
	Ribosome biogenesis in eukaryotes	10	0.10	3.85E−04
	Thiamine metabolism	4	0.36	3.96E−04
	RNA transport	13	0.08	5.91E−04
	Carbon fixation in photosynthetic organisms	8	0.12	6.00E−04
	Plant-pathogen interaction	12	0.07	1.63E−03
	mRNA surveillance pathway	9	0.08	3.38E−03
Profile15	Metabolic pathways	80	0.04	2.04E−10
	Biosynthesis of secondary metabolites	53	0.05	1.97E−09
	Biosynthesis of amino acids	21	0.08	9.79E−08
	Carbon fixation in photosynthetic organisms	11	0.16	3.57E−07
	Biosynthesis of unsaturated fatty acids	7	0.24	4.62E−06
	Glycolysis/gluconeogenesis	12	0.10	7.23E−06
	Carbon metabolism	18	0.07	8.63E−06
	Fatty acid metabolism	9	0.13	1.45E−05
	Cysteine and methionine metabolism	11	0.10	2.51E−05
	Terpenoid backbone biosynthesis	8	0.14	3.66E−05
Profile13	Circadian rhythm—plant	10	0.28	3.40E−11
	Flavonoid biosynthesis	6	0.29	2.81E−07
	Starch and sucrose metabolism	12	0.06	2.53E−06
	Metabolic pathways	41	0.02	1.10E−05
	Biosynthesis of secondary metabolites	27	0.03	3.22E−05
	Inositol phosphate metabolism	6	0.09	1.17E−04
	Glycolysis/gluconeogenesis	7	0.06	3.09E−04
	Stilbenoid, diarylheptanoid and gingerol biosynthesis	4	0.09	1.75E−03
	Carotenoid biosynthesis	3	0.10	4.31E−03
	Carbon fixation in photosynthetic organisms	4	0.06	6.89E−03
Profile12	Glycolysis/gluconeogenesis	11	0.09	1.36E−07
	Biosynthesis of secondary metabolites	31	0.03	1.08E−06
	Metabolic pathways	42	0.02	1.12E−05
	Glycine, serine and threonine metabolism	6	0.08	1.89E−04
	Carbon metabolism	9	0.03	2.65E−03
	Terpenoid backbone biosynthesis	4	0.07	4.35E−03
	Pentose phosphate pathway	4	0.07	4.35E−03
	Circadian rhythm—plant	3	0.08	8.26E−03
	Carbon fixation in photosynthetic organisms	4	0.06	7.75E−03
	Biosynthesis of amino acids	8	0.03	7.52E−03
Profile0	Protein processing in endoplasmic reticulum	23	0.11	1.42E−21
	Spliceosome	18	0.09	4.55E−16
	Endocytosis	5	0.04	1.78E−03
	RNA transport	5	0.03	3.69E−03
	Photosynthesis—antenna proteins	2	0.09	8.67E−03
	Plant-pathogen interaction	4	0.02	1.84E−02
	Circadian rhythm—plant	2	0.06	2.09E−02
	*N*-Glycan biosynthesis	2	0.05	2.99E−02

A total of 966 DEGs included in profile 11 were also upregulated in the DS and expressed at similar levels in DS and DCS. Significant pathways enriched in this profile were DNA replication, metabolic pathways, purine metabolism and glycolysis/gluconeogenesis, which suggested an accompany of carbohydrate metabolic pathway and protein biosynthesis during the formation of reproductive meristems and organs.

The profile 4 consisted of 1163 DEGs whose expression was down-regulated when the floral bud began to differentiate and reached a similar level in DCS. The profile 0 had 227 DEGs which showed a similar expression pattern to those in profile 4 but had a lower expression level in the DCS. The top three significantly enriched pathways in profile 4 were protein processing in endoplasmic reticulum, spliceosome, and endocytosis, which were also the most enriched ones in profile 0. Besides, circadian rhythm and photosynthesis were among the most enriched pathways in profile 0.

The profile 15 included 644 DEGs, which showed a robust up-regulation in the floral meristem differentiation stage but expressed most highly in DCS. The 376 DEGs involved in profile 13 and profile 12 showed a similar expression pattern to those in profile 15, but the rise of expression was gentler.

### Identification of CONSTANS genes involved in the photoperiod pathways

**Figure 4:**
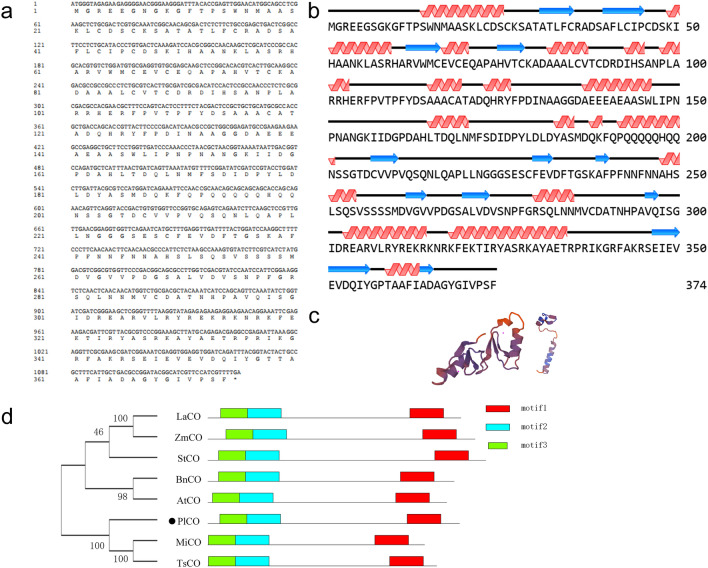
Gene sequence analysis and protein structure prediction of *PlCO*. (**a**) The sequence of ORF and deduced amino acid of *PlCO*; (**b**) The secondary structure of PlCO, red represents helix, blue represents coil and black lines represent strands; (**c**) Tertiary structure prediction of PlCO protein; (**d**) Phylogenetic and motif analysis of CO proteins. Phylogenetic tree of CO homologous proteins using the neighbor-joining method by MEGA 7. Bootstrap: 1000 replicates. Conservative motif analysis of CO homologous proteins using MEME. The proteins are as follows: LaCO: *Lolium arundinaceum*, ADA67904.1; ZmCO, *Zea* *mays*, ABV55996.1; *Solanum* *tuberosum*, NP_001274795.1; *Brassica* *napus*, AAC27694.1; AtCO, *Arabidopsis* *thaliana*, CAA71587.1; MiCO, *Mangifera* *indica*, AGA19018.1; TsCO, *Triadica* *sebifera*, ARS25033.1.

According to the DEG function enrichment, quite a few DEGs were enriched to circle rhythm pathway. So, we focus on photoperiod pathway genes downstream of the circadian rhythm and regulate the flowering in plants. As a result, a total of 584 genes involved in the photoperiod pathway with a similarity of more than 50% to those in Arabidopsis and rice were screened out, including *CO*, *CCA1*, *FKF1*, *FIO1*, *FRY1*, *NF-YB8* and *NF-YC*, which were critical genes regulated flowering in this pathway (Table S3). Including of these genes, *CO* is the output of the light pathway critical to the photoperiod pathway signal transduction to the floral bud. A total of 35 *CO* genes were identified, including 8 DEGs (Table 2). Among these 8 differential expressed *CO* genes, 6 of them were highly expressed in VS, implying a potential role in regulating floral bud transition from vegetative stage to reproductive stage. So, one of the 8 candidate *CO* genes, i1_LQ_HNbud_c108459/f1p9/1496 (HNbud_c108459) was selected as a representative in this study which expressed higher in VS and lower in DS and DCS. Therefore, this gene was cloned and its basic physiology character was analyzed online. The results showed that HNbud_c108459 coded a protein with 374 amino acids, which contained 27.81% alpha helix and 58.82% coils (Fig. 4a,b). Its 3-dimensional structure protein was predicted using SWISS-MODEL online software, which showed a model with 65.09% coincidence with CO proteins (Fig. 4c), which suggested a potential function similar to CO in other species. The deduced protein sequence encoded by the candidate gene was compared with other functional FT protein sequences, through phylogenetic analysis using MEGA 7 sofware28 (Fig. 4d). The results showed that HNbud_c108459 clustered with MiCO from *Mangifera indica* and TsCO from *Triadica sebifera*, which suggests that HNbud_c108459 was evolutionary related to CCT proteins of dicots. Conserved motifs in these proteins were then analyzed to further explore the function of the HNbud_c108459 protein. The results showed that the proteins shared the same motifs (Fig. 4d), indicating that the protein structure is highly conserved in plants, and they might have similar functions. The candidate CO homologous proteins are implicated in promoting flowering of tree peony. Notably, the candidate gene was named *PlCO* according to the above results.

**Table 2:** The *CO* genes identified in 'High Noon'.

ATH/rice gene ID	Gene ID	Identity %	Alignment length (aa)	FPKM in VS	FPKM in DS	FPKM in DCS
AT5G24930.1	i1_LQ_HNbud_c107688/f1p0/1380	84.66	365	0	0	0
AT5G24930.1	i1_LQ_HNbud_c112244/f1p0/1772	83.79	364	0	0	0
AT5G24930.1	i1_LQ_HNbud_c130667/f1p0/1505	80.18	338	0	0	0
AT5G24930.1	i1_HQ_HNbud_c107758/f2p14/1419	58.47	378	0	0	0
AT5G24930.1	i1_HQ_HNbud_c127026/f2p11/1376	58.47	378	0	0	0
AT5G24930.1	i1_HQ_HNbud_c167879/f4p19/1491	58.47	378	34.40	83.58	62.68
AT5G24930.1	i1_HQ_HNbud_c220871/f2p14/1357	58.47	378	0	0	0
AT5G24930.1	i1_LQ_HNbud_c103446/f1p10/1416	58.47	378	0	0	0
AT5G24930.1	i1_LQ_HNbud_c75179/f1p12/1377	58.47	378	0	0	0
AT5G24930.1	i1_LQ_HNbud_c75258/f1p15/1495	58.47	378	0.75	0.34	0.19
AT5G15850.1	i1_LQ_HNbud_c246352/f5p6/1385	51.94	310	0	0	0
AT3G02380.1	i1_LQ_HNbud_c75514/f1p4/1347	59.01	383	0	0	0
AT3G02380.1	i1_HQ_HNbud_c243873/f12p6/1451	58.96	385	52.26	24.18	16.61
AT3G02380.1	i1_LQ_HNbud_c36726/f1p4/1281	58.96	385	0	0	0
AT3G02380.1	i1_HQ_HNbud_c37538/f2p4/1333	58.7	385	0.02	0.04	0
AT3G02380.1	i1_HQ_HNbud_c7853/f9p5/1191	57.44	390	27.62	15.23	12.12
AT3G02380.1	i1_LQ_HNbud_c12325/f1p5/1137	57.14	336	0	0	0
AT5G57660.1	i1_LQ_HNbud_c180058/f1p3/1235	81.75	263	0	0	0
AT5G57660.1	i1_LQ_HNbud_c33880/f1p3/1237	81.75	263	0	0	0
AT5G57660.1	i1_LQ_HNbud_c52656/f1p3/1472	81.75	263	0	0	0
AT5G57660.1	i1_LQ_HNbud_c91177/f1p3/1430	81.75	263	0	0	0
AT5G57660.1	i1_LQ_HNbud_c251312/f1p3/1548	81.66	338	0	0	0
AT5G57660.1	i1_LQ_HNbud_c6871/f5p3/1425	81.66	338	0	0	0
AT5G57660.1	i1_LQ_HNbud_c19314/f1p3/1533	80.85	329	0	0	0
AT5G57660.1	i1_LQ_HNbud_c170194/f2p3/1499	79.29	338	0	0	0
Os08t0249000_01	i1_LQ_HNbud_c108459/f1p9/1496	60.98	41	2.68	0.79	0.78
AT3G07650.1	i1_LQ_HNbud_c227363/f1p0/1658	67.54	382	0	0	0
AT3G07650.1	i1_LQ_HNbud_c39779/f1p0/1640	55.67	203	0	0	0
AT3G07650.1	i1_LQ_HNbud_c103793/f2p1/1942	52.31	346	12.02	5.35	6.47
AT3G07650.1	i1_LQ_HNbud_c251477/f1p1/2324	52.31	346	0.44	0.11	0.08
AT3G07650.1	i1_HQ_HNbud_c21321/f5p1/1896	52.02	346	0	0	0

**Figure 5:**
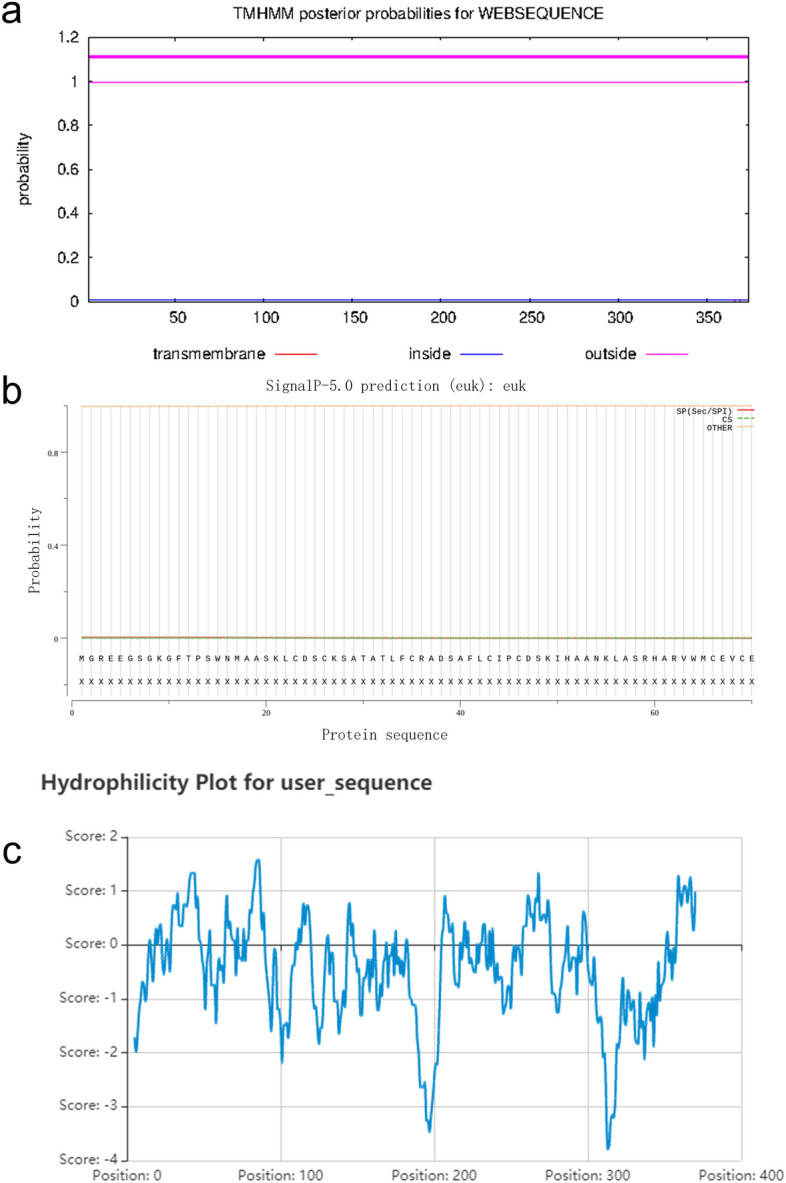
Bioinformational analysis of PlCO protein. (**a**) Trans-membrane prediction of PlCO; (**b**) signal peptide predicton of PlCO; (**c**) hydrophilicity prediction of PlCO.

The physiology and chemical character of PlCO protein were predicted online and the results showed that the PlCO protein did not have trans-membrane motif and signal peptide (Fig. 5a,b). The solubility of PlCO protein was predicted to 0.309, and the GRAVY of this protein was − 0.454, which indicated that PlCO protein was a hydrophilic protein (Fig. 5c).

**Figure 6:**
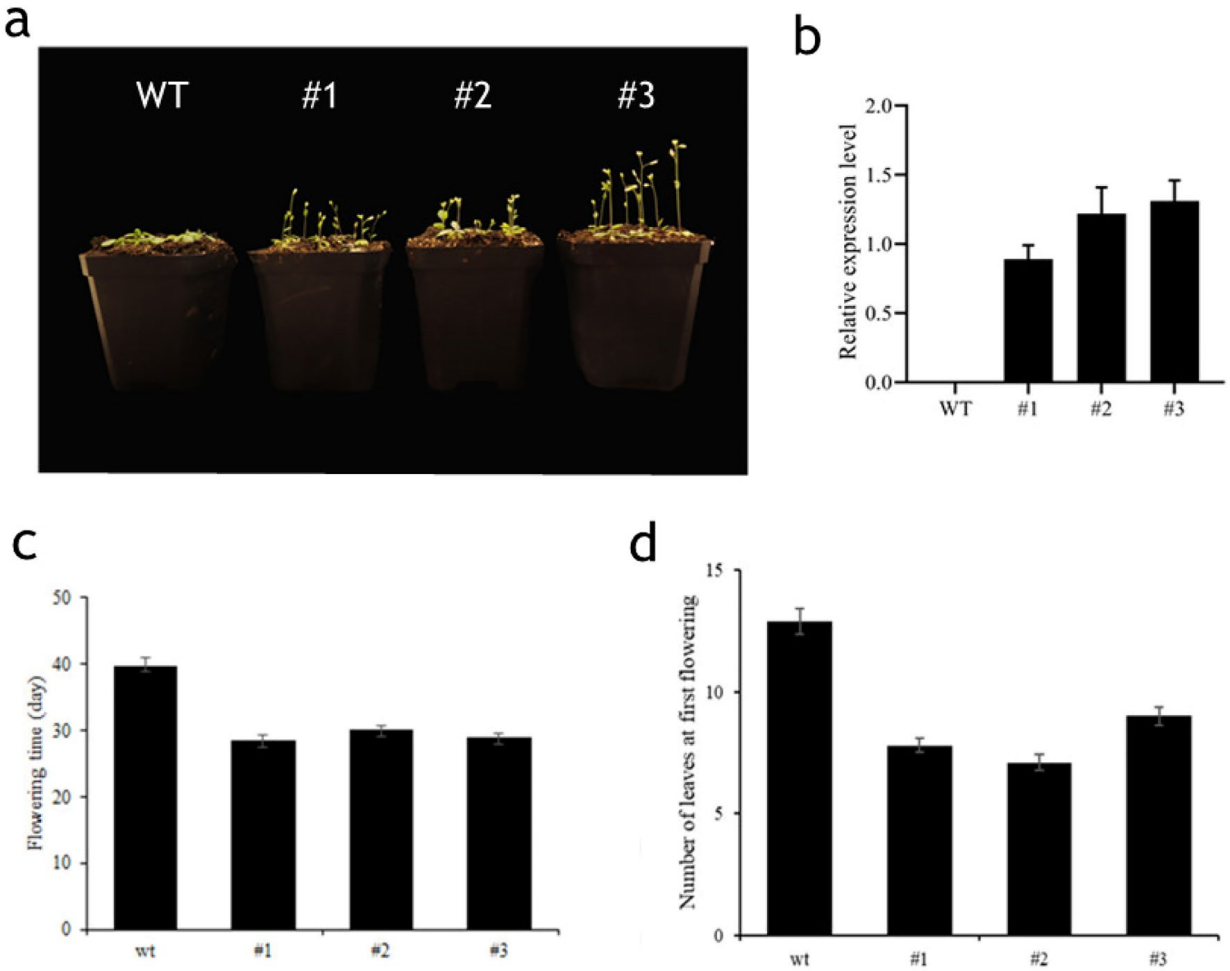
Flowering phenotype of *PlCO* over-expression in transgenic Arabidopsis plants. (**a**) The flowering phenotype of wild type and three trans-gene plants. WT, wild type; #1, #2 and #3 represent three lines of the *PlCO* over-expression transgenic plants. (**b**) The expression of PlCO gene in WT, #1, #2 and #3 transgenic plants. (**c**) The flowering time of transgenic Arabidopsis was less than WT. *p-value < 0.05. (**d**) The number of leaves at first flowering of WT and transgenic Arabidopsis. *p-value < 0.05.

In order to understand the biological function on flowering of *PlCO* gene comprehensively, we over-expressed *PlCO* gene in *Arabidopsis.* As a result, the over-expression of *PlCO* caused an early-flowering in *Arabidopsis* (Fig. 6a–d), which implied that these genes conduct a function of inducing flowering in tree peony.

**Figure 7:**
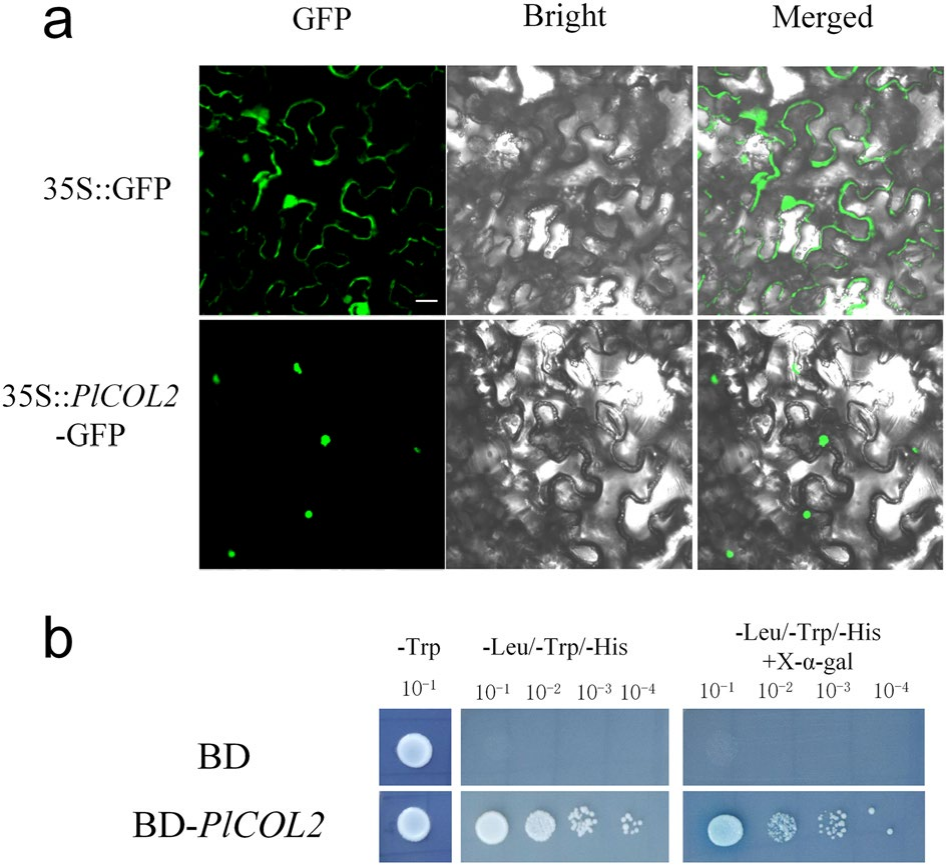
Subcellular localization and transcription activity ability of PlCO. (**a**) Subcellular localization of PlCO in tobacco leaves, Bar = 20 µm. (**b**) Transcription activation ability of PlCO in yeast.

To get a deep comprehension of molecular function of *PlCO* gene, we explored the subcellular localization of *PlCO*. The results showed that *PlCO* localized in the nucleus of tobacco whereas the control in both membrane and nucleus (Fig. 7a), which implied a potential function of transcription factor. So, then the transcription activity assay was conducted in yeast to deeply understand the function of *PlCO*, which resulted in an obvious transcription activation ability in yeast (Fig. 7b) whereas the negative control showed no activation. As a result, the *PlCO* gene possessed a nucleus localization and transcription activation ability, which might confer the coded protein an activation function for the expression of downstream genes regulating flowering.

**Figure 8:**
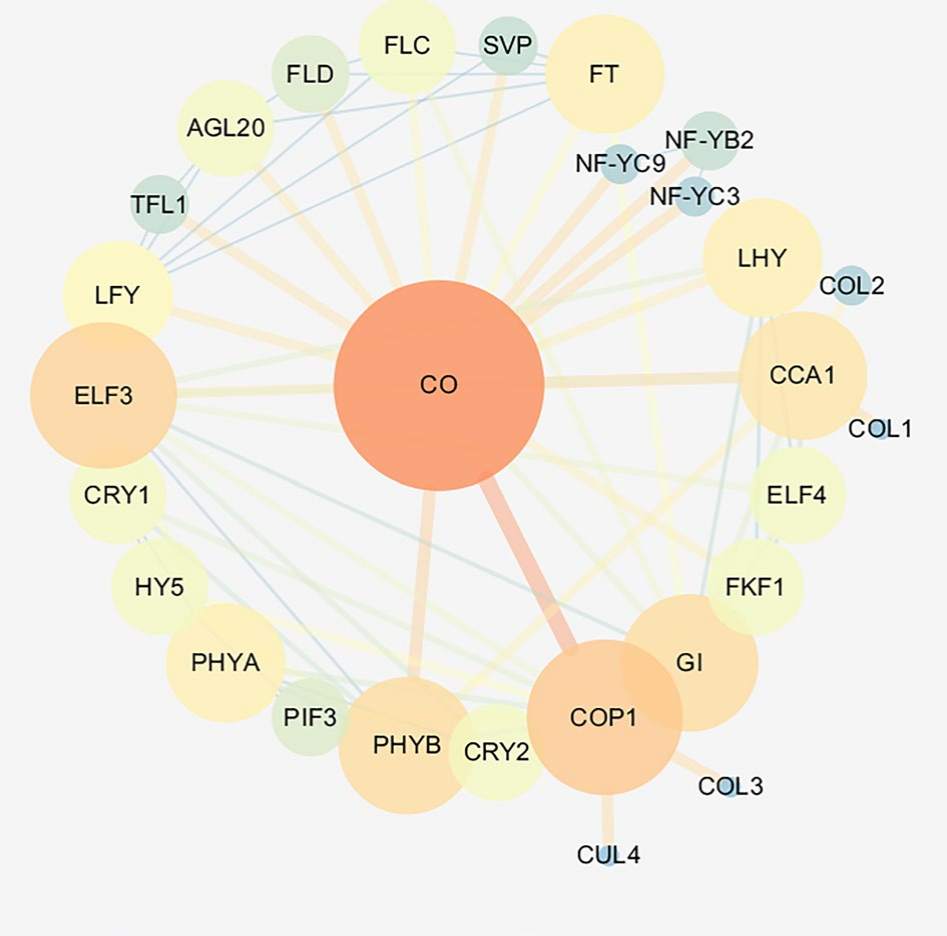
Predicted interaction network of PlCO. The circles represent proteins; the lines represent interaction relationships; the colors represent the correlation.

To explore the function mechanism of PlCO protein, the protein interaction network was predicted through STRING software using PlCO protein sequence. In this network, there were 29 proteins containing in circadian rhythm pathway genes, light signal pathways and flowering integrator *FT*, which implied that PlCO might interact or have a co-expression relationship with these proteins (Fig. 8).

## Discussion

Tree peony is a perennial plant that exhibits a seasonal flowering, along with which the complement of floral bud development is critical to the flowering. For the sake of successfully flowering, the floral bud began its development in the autumn and accomplished before winter dormancy. Generally, the floral bud of tree peony is formed from a vegetative bud, which undergoes a transition into the reproductive stage and a differentiation into a completed floral bud composed of all floral organ primordium. In this study, both the morphology and transcriptomic change of the vegetative stage, the floral stage, and the floral organ meristem stage were analyzed. The vegetative shoot meristem showed a "peak" while the reproductive meristem was flat, which was coincident with other species, such as *Dactylis glomerata*^47^. As the plant material in this study was 'High Noon', a re-blooming cultivar, the first flowering was similar to other cultivars which experienced winter dormancy. So, the floral bud development could represent the universal regular in tree peony. Nevertheless, the re-blooming characteristics and the *P. delavayi* lineage made this cultivar special for the floral bud development in genetics. So, the genetic analysis in 'High Noon' was necessary to clarify the molecular mechanism of floral bud development.

The DEG analysis revealed that the most DEGs were between VS and DCS, next was between VS and DS, which suggested that the floral transition from VS to DS was a remarkable process for plant development. The DEGs shared in the comparison between VS vs. DS and VS vs. DCS suggested that there were somehow similar regulatory patterns behind these stage transitions. GO terms enriched by the DEGs, including cell division, cell cycle, and chromosome organization revealed a probable vigorous cell division during floral bud development (Fig. 2). Correspondingly, the top enriched KEGG pathways containing plant hormone signal transduction, fructose, and mannose metabolism, circadian rhythm, implied the underlying metabolism pathways during floral bud development (Fig. 2). These findings were consistent with the analyses in the floral bud development of *Litchi chinensis*^48^. The expression pattern analyses and KEGG pathway enrichment of DEG profiles gave us a much clearer understanding of the regulatory mechanism of floral development (Fig. 3). A remarkable up-regulated expression pattern of genes involved in cell division and protein biosynthesis and down-regulated expression of genes involved in protein processes in floral bud development stages implied that the vegetative bud was making preparations for the material reserves of floral bud formation. The up-regulated expression of genes in plant hormone signal transduction and circadian rhythm pathway showed that the floral bud development in tree peony was regulated by both environment cues and endogenous signals. However, the issue that how these signals coordinate the genes in floral bud development needs an in-depth study.

The flowering transition is influenced by five pathways: photoperiod, vernalization, age, GA and autonomous pathway in plants. In the analyses of the gene expression pattern, the circadian rhythm pathway was upregulated during floral bud transition from VS to DS (Fig. 3, Table 1), which suggested that the photoperiod pathway conducted an important role in this process. In the previous study, the photoperiod pathway was an important regulating network in which FKF interacts with GI and induce the activity of CO, NF-YB and NF-YC, which was genetically required for CO-mediated floral promotion^49^. In this study, the specifically high expression before the floral transition of *FKF1*, *NF*-*YBs* and *NF*-*YCs* (Table S3) suggested a promotion role of photoperiod pathway in the floral transition and development of tree peony. In consideration of the both blooming of the 'High Noon' was in long-day condition, we suggested that the photoperiod pathway played an important role in the flowering of 'High Noon'. Further research about the molecular mechanism of light cycle underlying the flowering of 'High Noon' is needed.

*CO* was a critical gene in photoperiod signal pathway, which was the output gene of circle rhythm and connected it with flowering integrator genes *FT* to induce flowering^24^. Therefore, we selected one of them, *PlCO*, to verify the function during this process. The PlCO protein had a zinc-finger structure and a CCT motif, the latter conducted the protein a nucleus localization signal^25^. In this study, PlCO possessed the both motif, which implied that PlCO might had a similar potential function with CO proteins in other plants. The subcellular localization assay showed that the PlCO protein localized in nucleus, which together with the transcription activity ability confirmed the transcription factor potential of PlCO protein to regulate gene expression. Overexpression of *PlCO* in *Arabidopsis* exhibited an early flowering phenotype (Fig. 6). All of these results suggested that this candidate gene *PlCO* might conduct a positive function in the flowering time of tree peony through transcription regulation. The protein interaction network analysis of PlCO in this study showed that PlCO had a close relationship with other members in photoperiod pathway, like FKF1, PHYB, CCA, and floral integrator FT (Fig. 8). In rice, CO and HEADING DATE1 (HD1) coordinate with the NF-Y B/C dimer to specifically target the promoters of their target genes^25,50^. In *Atft* mutant, the overexpression of *CO* gene did not affect flowering time, which implied that CO regulated flowering through *FT* gene^24,51^. The *FT* gene in 'High Noon' was cloned and proved to promote flowering in *Arabidopsis*^19^. However, the connection between *PlCO* and other genes regulating flowering bud development in tree peony was not revealed. Therefore, whether PlCO protein binds to the promoter of *PlFT* or functions with other members in photoperiod pathway needs to be focused in the further study.

## Conclusions

The intricate regulatory network of floral bud development in tree peony 'High Noon' was revealed by transcriptome analyses in this study. Genes involved in photoperiod pathway were identified and a CO homolog gene, *PlCO*, was identified to exhibited a nucleus localization and transcription activity ability. Overexpression of *PlCO* significantly advanced the flowering time in transgenic *A. thaliana* plants. Our results provide a valuable resource for further studying the molecular mechanism and flowering time modified genetic resources for molecular breeding in tree peony or other closely relative cultivars.

## Supplementary Information

Supplementary Information.

## Data Availability

All the data analyzed during the current study are publicly available in NCBI Sequence Read Archive (SRA) database (SRX6373700-SRX6373708) and Gene Expression Omnibus (GSE133476).

## References

[1] Zhou SL (2014). Multiple species of wild tree peonies gave rise to the 'king of flowers' *Paeonia*
*suffruticosa* Andrews. Proc. Biol. Sci..

[2] Yang Y (2020). Germplasm resources and genetic breeding of Paeonia: A systematic review. Hortic. Res..

[3] Wang Y, Lu Y, Guo Z, Ding Y, Ding C (2020). RICE CENTRORADIALIS 1, a TFL1-like gene, responses to drought stress and regulates rice flowering transition. Rice (N Y).

[4] Izawa T (2021). What is going on with the hormonal control of flowering in plants?. Plant J..

[5] Wang Y (2020). Molecular variation in a functionally divergent homolog of FCA regulates flowering time in *Arabidopsis*
*thaliana*. Nat. Commun..

[6] Tayengwa R, Sharma Koirala P, Pierce CF, Werner BE, Neff MM (2020). Overexpression of AtAHL20 causes delayed flowering in Arabidopsis via repression of FT expression. BMC Plant Biol..

[7] Manuela D, Xu M (2020). Juvenile leaves or adult leaves: determinants for vegetative phase change in flowering plants. Int. J. Mol. Sci..

[8] Lee KC (2022). The splicing factor 1-FLOWERING LOCUS M module spatially regulates temperature-dependent flowering by modulating FLOWERING LOCUS T and LEAFY expression. Plant Cell Rep..

[9] Chu L, Yang C, Zhuang F, Gao Y, Luo M (2022). The HDA9-HY5 module epigenetically regulates flowering time in *Arabidopsis thaliana*. J. Cell. Physiol..

[10] Praena J, van Veen E, Henriques R, Benlloch R (2022). Assessing flowering time under different photoperiods. Methods Mol. Biol..

[11] Maeda AE, Nakamichi N (2022). Plant clock modifications for adapting flowering time to local environments. Plant Physiol..

[12] Park DH (1999). Control of circadian rhythms and photoperiodic flowering by the Arabidopsis GIGANTEA gene. Science.

[13] Wang ZY, Tobin EM (1998). Constitutive expression of the CIRCADIAN CLOCK ASSOCIATED 1 (CCA1) gene disrupts circadian rhythms and suppresses its own expression. Cell.

[14] Kumimoto RW, Zhang Y, Siefers N, Holt BF (2010). NF-YC3, NF-YC4 and NF-YC9 are required for CONSTANS-mediated, photoperiod-dependent flowering in *Arabidopsis thaliana*. Plant J..

[15] Wang S (2015). Molecular cloning and potential function prediction of homologous SOC1 genes in tree peony. Plant Cell Rep..

[16] Hao Q (2017). Overexpression of PSK1, a SKP1-like gene homologue, from *Paeonia suffruticosa*, confers salinity tolerance in Arabidopsis. Plant Cell Rep..

[17] Shu Q (2012). Analysis of the formation of flower shapes in wild species and cultivars of tree peony using the MADS-box subfamily gene. Gene.

[18] Ren, X., Wang, S., Xue, J., Zhu, F. & Zhang, X. Molecular cloning and expression analysis of cryptochrome gene PsCRY2 in tree peony. *Hortic. Plant J.* **2** (2017).

[19] Zhou H (2015). Isolation and functional analysis of flowering locus T in tree peonies (PsFT). J. Am. Soc. Hortic. Sci..

[20] Gao X (2021). Transcriptome analysis and identification of genes associated with floral transition and fruit development in rabbiteye blueberry (*Vaccinium ashei*). PLoS One.

[21] Xing L (2019). Comparative RNA-sequencing and DNA methylation analyses of apple (*Malus domestica* Borkh.) buds with diverse flowering capabilities reveal novel insights into the regulatory mechanisms of flower bud formation. Plant Cell Physiol..

[22] Li Y (2018). A transcriptome analysis of two apple (*Malus *× *domestica*) cultivars with different flowering abilities reveals a gene network module associated with floral transitions. Entia Hortic..

[23] Shangguan L (2020). Comparative transcriptome analysis provides insight into regulation pathways and temporal and spatial expression characteristics of grapevine (*Vitis*
*vinifera*) dormant buds in different nodes. BMC Plant Biol..

[24] Suárez-López P, Wheatley K, Robson F, Onouchi H, Coupland G (2001). CONSTANS mediates between the circadian clock and the control of flowering in Arabidopsis. Nature.

[25] Shen C, Liu H, Guan Z, Yan J, Xing Y (2020). Structural insight into DNA recognition by CCT/NF-YB/YC complexes in plant photoperiodic flowering. Plant Cell.

[26] Fan C (2014). Conserved CO-FT regulons contribute to the photoperiod flowering control in soybean. BMC Plant Biol..

[27] Wang S (2019). De novo sequencing of tree peony (*Paeonia suffruticosa*) transcriptome to identify critical genes involved in flowering and floral organ development. BMC Genom..

[28] Wang X (2020). Defoliation, not gibberellin, induces tree peony autumn reflowering regulated by carbon allocation and metabolism in buds and leaves. Plant Physiol. Biochem..

[29] Chang Y (2019). Transcriptome profiling for floral development in reblooming cultivar 'High Noon' of *Paeonia suffruticosa*. Sci. Data.

[30] Langmead B, Salzberg SL (2012). Fast gapped-read alignment with Bowtie 2. Nat. Methods.

[31] Li B, Dewey CN (2011). RSEM: Accurate transcript quantification from RNA-Seq data with or without a reference genome. BMC Bioinform..

[32] Anders S (2013). Count-based differential expression analysis of RNA sequencing data using R and bioconductor. Nat. Protoc..

[33] Xie C (2011). KOBAS 2.0: A web server for annotation and identification of enriched pathways and diseases. Nucleic Acids Res..

[34] Minoru K, Yoko S, Masayuki K, Miho F, Mao T (2016). KEGG as a reference resource for gene and protein annotation. Nucleic Acids Res..

[35] Ernst J, Bar-Joseph Z (2006). STEM: A tool for the analysis of short time series gene expression data. BMC Bioinform..

[36] Ehrenreich IM (2009). Candidate gene association mapping of Arabidopsis flowering time. Genetics.

[37] Irish VF (2010). The flowering of Arabidopsis flower development. Plant J..

[38] Shrestha R, Gomez-Ariza J, Brambilla V, Fornara F (2014). Molecular control of seasonal flowering in rice, arabidopsis and temperate cereals. Ann. Bot..

[39] Kumar S, Stecher G, Tamura K (2016). MEGA7: Molecular evolutionary genetics analysis version 7.0 for bigger datasets. Mol. Biol. Evol..

[40] Bailey TL (2009). MEME SUITE: Tools for motif discovery and searching. Nucleic Acids Res..

[41] Geourjon C, Deleage G (1995). SOPMA: Significant improvements in protein secondary structure prediction by consensus prediction from multiple alignments. Comput. Appl. Biosci..

[42] Waterhouse A (2018). SWISS-MODEL: Homology modelling of protein structures and complexes. Nucleic Acids Res..

[43] Chen Y, Yu P, Luo J, Jiang Y (2003). Secreted protein prediction system combining CJ-SPHMM, TMHMM, and PSORT. Mamm. Genome.

[44] Almagro Armenteros JJ (2019). SignalP 5.0 improves signal peptide predictions using deep neural networks. Nat. Biotechnol..

[45] Hebditch M, Carballo-Amador MA, Charonis S, Curtis R, Warwicker J (2017). Protein-Sol: A web tool for predicting protein solubility from sequence. Bioinformatics.

[46] Szklarczyk D (2019). STRING v11: Protein–protein association networks with increased coverage, supporting functional discovery in genome-wide experimental datasets. Nucleic Acids Res..

[47] Feng G (2017). Comprehensive transcriptome analysis reveals distinct regulatory programs during vernalization and floral bud development of orchardgrass (*Dactylis*
*glomerata* L.). BMC Plant Biol..

[48] Zhang H (2016). Morphological characterization and gene expression profiling during bud development in a tropical perennial *Litchi chinensis* Sonn. Front. Plant Sci..

[49] Song YH (2014). Distinct roles of FKF1, Gigantea, and Zeitlupe proteins in the regulation of Constans stability in Arabidopsis photoperiodic flowering. Proc. Natl. Acad. Sci. U.S.A..

[50] Nakamichi N (2020). Flowering time control in rice by introducing Arabidopsis clock-associated PSEUDO-RESPONSE REGULATOR 5. Biosci. Biotechnol. Biochem..

[51] Yoo SK (2005). CONSTANS activates SUPPRESSOR OF OVEREXPRESSION OF CONSTANS 1 through FLOWERING LOCUS T to promote flowering in Arabidopsis. Plant Physiol..

