# Supplementary material for: Transcriptome analysis of floral bud development and function analysis of a novel CO gene in Paeonia × lemoinei ‘High Noon’
Source: Sci Rep. 2022 Oct 14;12:17281. doi: 10.1038/s41598-022-22195-z (PMC9568513; doi:10.1038/s41598-022-22195-z)
Supplement: Supplementary file 1 — Supplementary Information. [file 41598_2022_22195_MOESM1_ESM.docx]

Supplementary Figures:


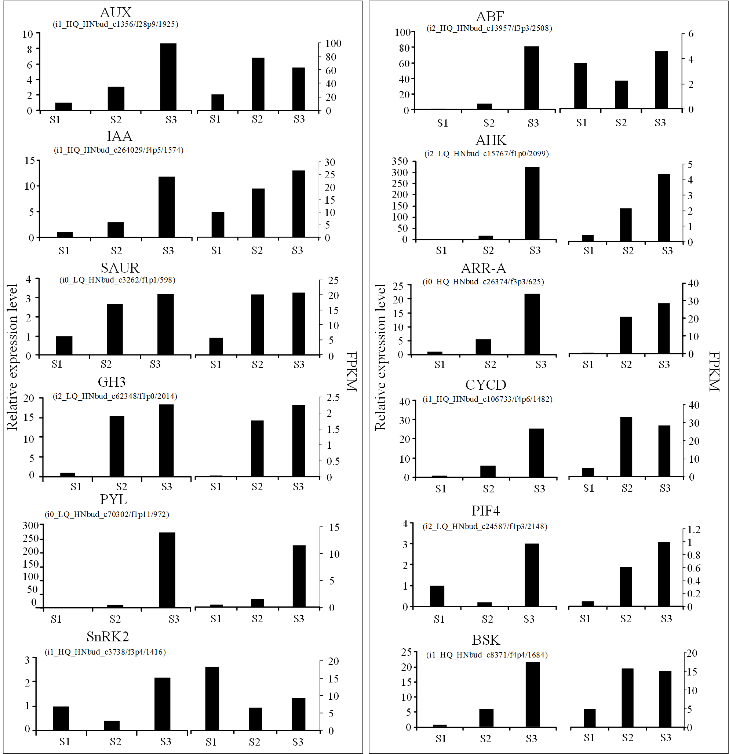


Figure S1. qRT-PCR verification for RNA-seq data, validation of the expression level of plant hormone-related genes with qRT-PCR and FPKM value. Data from qRT-PCR are means of three replicates and FPKM are means of replicates. The qRT-PCR data are on the left and the FPKM value were on the right. Bar represents standard error (SE).

Supplementary Tables:

**TableS1. List of DEGs during the floral development in tree peony.**

| Transcript ID | Transcript ID | Transcript ID |  |
| --- | --- | --- | --- |
| i2_LQ_HNbud_c52674/f1p46/2753 | i2_HQ_HNbud_c14685/f5p28/2197 | i3_HQ_HNbud_c1712/f2p1/3282 | i1_LQ_HNbud_c68783/f1p8/1784 |
| i1_HQ_HNbud_c26034/f2p9/1431 | i1_LQ_HNbud_c199997/f1p6/1965 | i1_LQ_HNbud_c222374/f1p5/1265 | i1_HQ_HNbud_c46145/f43p5/1275 |
| i2_HQ_HNbud_c1673/f3p3/2271 | i1_LQ_HNbud_c127097/f1p18/1725 | i1_LQ_HNbud_c89054/f1p8/1781 | i2_LQ_HNbud_c7029/f1p0/2138 |
| i2_LQ_HNbud_c23550/f1p1/2876 | i0_LQ_HNbud_c5425/f1p0/877 | i0_HQ_HNbud_c379/f11p0/842 | i1_HQ_HNbud_c33731/f5p7/1855 |
| i3_LQ_HNbud_c4577/f1p3/3538 | i2_HQ_HNbud_c47578/f8p3/2529 | i0_LQ_HNbud_c77438/f1p0/872 | i1_LQ_HNbud_c20814/f1p54/1186 |
| i2_HQ_HNbud_c47931/f4p2/2851 | i3_LQ_HNbud_c13159/f1p0/3705 | i1_HQ_HNbud_c14526/f2p37/1054 | i1_HQ_HNbud_c120912/f8p4/1861 |
| i0_LQ_HNbud_c98375/f3p0/764 | i0_LQ_HNbud_c98275/f14p1/727 | i1_LQ_HNbud_c109371/f1p23/1806 | i1_HQ_HNbud_c5104/f16p4/1926 |
| i4_HQ_HNbud_c51/f6p0/5036 | i3_LQ_HNbud_c2230/f1p1/3195 | i0_HQ_HNbud_c4351/f3p3/544 | i3_HQ_HNbud_c938/f5p2/3358 |
| i0_HQ_HNbud_c7477/f3p1/733 | i0_LQ_HNbud_c25396/f1p7/743 | i1_LQ_HNbud_c222805/f1p3/1646 | i1_LQ_HNbud_c124651/f1p6/1948 |
| i1_LQ_HNbud_c30092/f1p9/1141 | i2_LQ_HNbud_c747/f14p3/2535 | i0_LQ_HNbud_c72693/f1p0/869 | i0_LQ_HNbud_c46976/f1p0/809 |
| i3_LQ_HNbud_c3677/f1p4/4014 | i1_LQ_HNbud_c8071/f2p3/1665 | i0_HQ_HNbud_c1137/f16p3/662 | i0_HQ_HNbud_c50432/f2p4/578 |
| i1_HQ_HNbud_c2030/f7p1/1184 | i2_LQ_HNbud_c5003/f1p8/2311 | i0_LQ_HNbud_c82095/f1p0/942 | i1_LQ_HNbud_c214019/f1p19/901 |
| i1_LQ_HNbud_c26109/f1p0/1554 | i1_LQ_HNbud_c37716/f4p9/1433 | i4_LQ_HNbud_c2786/f1p1/4542 | i1_HQ_HNbud_c264754/f104p24/1739 |
| i2_HQ_HNbud_c38392/f2p2/2880 | i0_HQ_HNbud_c1832/f4p1/921 | i0_HQ_HNbud_c98677/f3p0/712 | i1_HQ_HNbud_c216922/f6p1/1101 |
| i1_HQ_HNbud_c3311/f2p0/1506 | i1_HQ_HNbud_c263384/f7p0/1211 | i0_LQ_HNbud_c44303/f1p2/837 | i4_HQ_HNbud_c68/f3p0/4476 |
| i1_LQ_HNbud_c127215/f1p3/1750 | i2_LQ_HNbud_c26046/f1p7/2858 | i1_LQ_HNbud_c175217/f1p4/1233 | i1_LQ_HNbud_c55300/f1p8/1514 |
| i3_LQ_HNbud_c16022/f1p2/3037 | i0_HQ_HNbud_c24435/f2p1/484 | i1_LQ_HNbud_c111603/f1p4/1629 | i3_LQ_HNbud_c15408/f1p5/3020 |
| i0_LQ_HNbud_c83156/f1p0/880 | i1_LQ_HNbud_c248263/f1p2/1243 | i1_LQ_HNbud_c201765/f1p0/1569 | i0_HQ_HNbud_c30222/f2p0/706 |
| i0_HQ_HNbud_c10126/f2p4/964 | i1_HQ_HNbud_c217772/f5p2/1987 | i1_HQ_HNbud_c264866/f31p4/1933 | i0_HQ_HNbud_c1998/f11p11/755 |
| i2_HQ_HNbud_c31720/f2p3/2047 | i0_LQ_HNbud_c20308/f1p2/898 | i2_LQ_HNbud_c57638/f1p3/2947 | i1_HQ_HNbud_c23351/f2p4/2010 |
| i1_LQ_HNbud_c56601/f1p2/1875 | i1_LQ_HNbud_c204117/f1p16/1274 | i1_LQ_HNbud_c99024/f1p4/1030 | i2_LQ_HNbud_c10602/f1p0/2310 |
| i2_LQ_HNbud_c36453/f1p2/2973 | i0_HQ_HNbud_c6390/f7p22/552 | i1_HQ_HNbud_c1623/f44p15/1812 | i1_HQ_HNbud_c265032/f6p1/1955 |
| i0_HQ_HNbud_c52458/f2p1/703 | i0_LQ_HNbud_c4451/f1p2/870 | i0_HQ_HNbud_c64616/f14p0/669 | i2_LQ_HNbud_c11192/f1p2/2596 |
| i2_LQ_HNbud_c4161/f1p2/2801 | i0_LQ_HNbud_c73451/f1p9/609 | i0_HQ_HNbud_c19105/f3p0/692 | i0_HQ_HNbud_c5685/f2p1/758 |
| i0_HQ_HNbud_c732/f18p0/690 | i2_LQ_HNbud_c54862/f1p2/2681 | i0_LQ_HNbud_c85934/f2p0/589 | i3_HQ_HNbud_c1835/f2p1/3595 |
| i1_HQ_HNbud_c14251/f8p1/1753 | i0_HQ_HNbud_c98276/f13p1/666 | i3_LQ_HNbud_c13324/f1p0/3983 | i4_LQ_HNbud_c912/f1p3/4177 |
| i2_HQ_HNbud_c1181/f31p18/2198 | i1_LQ_HNbud_c241628/f1p8/1090 | i1_HQ_HNbud_c159627/f2p1/1510 | i1_HQ_HNbud_c1913/f19p8/1657 |
| i2_HQ_HNbud_c831/f10p3/2956 | i0_LQ_HNbud_c102307/f1p0/909 | i0_LQ_HNbud_c66472/f1p3/932 | i1_LQ_HNbud_c202561/f1p11/1862 |
| i0_HQ_HNbud_c27612/f2p0/990 | i1_LQ_HNbud_c280165/f1p15/1019 | i1_HQ_HNbud_c32324/f7p2/1677 | i3_LQ_HNbud_c5859/f1p16/3134 |
| i0_HQ_HNbud_c1880/f7p7/646 | i3_HQ_HNbud_c16904/f4p5/3443 | i0_HQ_HNbud_c20344/f2p11/619 | i0_LQ_HNbud_c24069/f2p8/861 |
| i1_HQ_HNbud_c122439/f21p7/1205 | i2_LQ_HNbud_c47202/f1p0/2040 | i1_LQ_HNbud_c279446/f1p4/952 | i2_LQ_HNbud_c30656/f1p0/2021 |
| i0_LQ_HNbud_c8172/f2p2/587 | i0_HQ_HNbud_c64719/f17p0/631 | i0_HQ_HNbud_c99066/f14p0/660 | i2_HQ_HNbud_c2099/f4p2/2923 |
| i0_HQ_HNbud_c2273/f2p4/693 | i0_HQ_HNbud_c32413/f8p0/709 | i0_LQ_HNbud_c5105/f1p1/958 | i0_LQ_HNbud_c103458/f1p0/507 |
| i1_LQ_HNbud_c38282/f1p7/1884 | i1_LQ_HNbud_c92741/f1p0/1419 | i1_HQ_HNbud_c219658/f172p4/1672 | i3_LQ_HNbud_c1623/f2p13/3788 |
| i1_LQ_HNbud_c110845/f1p1/1619 | i0_LQ_HNbud_c19197/f2p17/567 | i2_LQ_HNbud_c26920/f1p17/2901 | i0_HQ_HNbud_c98237/f7p0/555 |
| i1_HQ_HNbud_c46577/f2p5/1089 | i0_HQ_HNbud_c37914/f40p5/715 | i1_HQ_HNbud_c63087/f9p3/1191 | i1_LQ_HNbud_c129292/f1p2/1189 |
| i0_LQ_HNbud_c374/f22p0/676 | i0_HQ_HNbud_c8043/f2p0/790 | i3_LQ_HNbud_c3604/f1p1/3102 | i1_HQ_HNbud_c86854/f2p4/1364 |
| i1_LQ_HNbud_c109815/f2p2/1152 | i3_HQ_HNbud_c1913/f2p7/3178 | i0_LQ_HNbud_c64641/f1p0/703 | i1_HQ_HNbud_c74681/f4p5/1322 |
| i0_HQ_HNbud_c66677/f2p0/797 | i0_LQ_HNbud_c3296/f1p2/650 | i1_LQ_HNbud_c98910/f1p14/1059 | i1_LQ_HNbud_c224020/f1p24/1715 |
| i1_HQ_HNbud_c93542/f2p2/1851 | i1_HQ_HNbud_c11249/f4p3/1309 | i0_LQ_HNbud_c68188/f1p2/727 | i1_LQ_HNbud_c75274/f1p7/1609 |
| i2_HQ_HNbud_c70206/f2p31/2592 | i0_HQ_HNbud_c5109/f3p8/812 | i1_LQ_HNbud_c106324/f1p11/1134 | i2_HQ_HNbud_c49835/f2p0/2505 |
| i3_LQ_HNbud_c11737/f1p2/3884 | i3_HQ_HNbud_c1988/f2p0/3147 | i1_HQ_HNbud_c12271/f2p2/1712 | i2_HQ_HNbud_c65838/f12p10/2556 |
| i0_HQ_HNbud_c20913/f4p12/948 | i0_LQ_HNbud_c43669/f1p0/914 | i1_LQ_HNbud_c245431/f3p9/1054 | i0_HQ_HNbud_c98378/f58p0/660 |
| i3_LQ_HNbud_c2683/f1p0/3326 | i3_HQ_HNbud_c17044/f3p0/3084 | i0_HQ_HNbud_c18340/f2p2/990 | i1_LQ_HNbud_c268124/f1p1/1456 |
| i3_HQ_HNbud_c10974/f2p1/3696 | i0_HQ_HNbud_c66852/f2p0/732 | i1_LQ_HNbud_c269024/f1p16/1184 | i0_HQ_HNbud_c13075/f3p1/684 |
| i1_HQ_HNbud_c208886/f2p10/1238 | i4_HQ_HNbud_c252/f2p0/4096 | i0_LQ_HNbud_c20030/f1p3/853 | i2_LQ_HNbud_c41171/f1p0/2357 |
| i0_HQ_HNbud_c13669/f7p1/687 | i2_HQ_HNbud_c70396/f3p3/2576 | i0_LQ_HNbud_c39756/f4p36/670 | i3_LQ_HNbud_c8499/f1p1/3085 |
| i2_HQ_HNbud_c47924/f2p0/2041 | i1_LQ_HNbud_c105074/f1p7/1403 | i0_HQ_HNbud_c22483/f2p0/881 | i0_HQ_HNbud_c1773/f8p2/578 |
| i2_HQ_HNbud_c5231/f4p5/2790 | i1_HQ_HNbud_c17199/f13p4/1341 | i1_LQ_HNbud_c34336/f1p5/1840 | i1_LQ_HNbud_c133057/f1p43/1549 |
| i1_LQ_HNbud_c264776/f119p16/1206 | i1_LQ_HNbud_c53947/f3p1/1551 | i1_HQ_HNbud_c192030/f53p8/1793 | i2_HQ_HNbud_c12732/f2p1/2834 |
| i1_HQ_HNbud_c207660/f5p3/1045 | i0_HQ_HNbud_c95392/f2p0/671 | i0_HQ_HNbud_c7694/f2p0/722 | i1_HQ_HNbud_c26226/f2p2/1828 |
| i1_HQ_HNbud_c33480/f3p3/1816 | i0_LQ_HNbud_c12768/f1p0/635 | i0_LQ_HNbud_c23134/f1p3/639 | i1_LQ_HNbud_c108677/f1p2/1601 |
| i2_LQ_HNbud_c26132/f1p15/2463 | i0_LQ_HNbud_c29734/f1p0/750 | i0_LQ_HNbud_c69775/f1p0/834 | i1_LQ_HNbud_c130357/f2p0/1517 |
| i2_HQ_HNbud_c22210/f2p10/2513 | i0_LQ_HNbud_c42171/f1p1/945 | i2_HQ_HNbud_c12364/f2p24/2690 | i0_HQ_HNbud_c42094/f4p0/508 |
| i0_LQ_HNbud_c85105/f1p0/541 | i0_LQ_HNbud_c74133/f1p0/913 | i3_LQ_HNbud_c10210/f1p6/3200 | i3_LQ_HNbud_c6054/f1p0/3396 |
| i0_LQ_HNbud_c58849/f2p0/688 | i2_LQ_HNbud_c33772/f1p0/2533 | i0_LQ_HNbud_c12558/f1p1/824 | i0_LQ_HNbud_c12994/f1p0/644 |
| i1_LQ_HNbud_c151540/f1p9/1387 | i1_LQ_HNbud_c40083/f1p7/1663 | i2_LQ_HNbud_c23165/f1p3/2781 | i1_HQ_HNbud_c174283/f2p4/1310 |
| i1_LQ_HNbud_c195309/f1p2/1479 | i3_LQ_HNbud_c16068/f1p1/3040 | i2_HQ_HNbud_c4439/f2p1/2820 | i2_LQ_HNbud_c64636/f11p0/2287 |
| i0_LQ_HNbud_c27720/f1p0/721 | i0_HQ_HNbud_c13109/f3p1/513 | i0_HQ_HNbud_c98480/f49p0/647 | i0_HQ_HNbud_c12638/f8p0/691 |
| i0_HQ_HNbud_c6080/f6p0/643 | i0_LQ_HNbud_c9930/f1p0/722 | i1_LQ_HNbud_c195976/f1p1/1935 | i0_HQ_HNbud_c1509/f6p11/642 |
| i0_HQ_HNbud_c17085/f17p0/757 | i0_HQ_HNbud_c10282/f15p1/709 | i1_LQ_HNbud_c4401/f2p0/1736 | i0_HQ_HNbud_c1373/f7p9/698 |
| i3_LQ_HNbud_c5283/f1p0/3837 | i0_HQ_HNbud_c1029/f14p0/543 | i0_LQ_HNbud_c5885/f1p0/828 | i1_LQ_HNbud_c73826/f1p12/1435 |
| i0_HQ_HNbud_c39143/f5p0/641 | i2_LQ_HNbud_c41470/f1p1/2579 | i0_LQ_HNbud_c26398/f1p0/767 | i1_LQ_HNbud_c28601/f1p11/1087 |
| i1_HQ_HNbud_c229043/f3p1/1645 | i0_HQ_HNbud_c1019/f10p0/696 | i0_HQ_HNbud_c3207/f3p1/569 | i0_LQ_HNbud_c76323/f1p0/856 |
| i0_LQ_HNbud_c79814/f1p0/764 | i1_HQ_HNbud_c6074/f10p2/1817 | i2_HQ_HNbud_c33549/f3p5/2744 | i0_HQ_HNbud_c98337/f2p0/747 |
| i1_LQ_HNbud_c198922/f1p31/1689 | i1_HQ_HNbud_c100218/f18p8/1214 | i2_LQ_HNbud_c62679/f1p1/2057 | i0_LQ_HNbud_c72870/f1p0/636 |
| i0_HQ_HNbud_c39556/f2p0/689 | i1_HQ_HNbud_c12640/f10p3/1233 | i2_HQ_HNbud_c19024/f2p4/2783 | i1_LQ_HNbud_c242130/f1p0/1094 |
| i0_LQ_HNbud_c108996/f1p3/718 | i0_LQ_HNbud_c45359/f1p0/606 | i1_HQ_HNbud_c265809/f43p0/1107 | i3_LQ_HNbud_c14958/f1p0/3553 |
| i0_HQ_HNbud_c64614/f11p0/661 | i1_LQ_HNbud_c227827/f1p6/1826 | i1_HQ_HNbud_c32450/f8p2/1507 | i1_LQ_HNbud_c25770/f8p2/1301 |
| i0_LQ_HNbud_c74942/f1p3/845 | i0_HQ_HNbud_c7354/f4p3/732 | i1_HQ_HNbud_c54110/f5p0/1854 | i0_HQ_HNbud_c25047/f2p5/585 |
| i7_LQ_HNbud_c645/f1p0/7989 | i0_LQ_HNbud_c30255/f2p4/453 | i2_LQ_HNbud_c39954/f1p1/2116 | i0_LQ_HNbud_c11402/f1p2/695 |
| i2_LQ_HNbud_c42369/f1p25/2830 | i2_LQ_HNbud_c54745/f1p2/2256 | i0_HQ_HNbud_c2361/f4p7/502 | i2_LQ_HNbud_c39116/f1p1/2393 |
| i0_HQ_HNbud_c904/f9p0/677 | i0_HQ_HNbud_c18606/f9p1/519 | i1_LQ_HNbud_c111625/f1p5/1244 | i3_LQ_HNbud_c9419/f1p12/3565 |
| i0_HQ_HNbud_c1201/f12p7/663 | i3_LQ_HNbud_c13358/f1p6/3387 | i0_LQ_HNbud_c10215/f2p1/484 | i1_LQ_HNbud_c197258/f2p2/1294 |
| i1_HQ_HNbud_c40668/f4p2/1331 | i0_HQ_HNbud_c1775/f3p0/601 | i2_HQ_HNbud_c44858/f4p0/2042 | i1_HQ_HNbud_c89215/f4p10/1752 |
| i1_LQ_HNbud_c203443/f1p5/1278 | i0_LQ_HNbud_c53095/f1p1/766 | i2_LQ_HNbud_c38243/f1p0/2191 | i4_LQ_HNbud_c444/f1p0/4235 |
| i0_HQ_HNbud_c1893/f8p6/771 | i0_HQ_HNbud_c11802/f3p8/639 | i0_HQ_HNbud_c38291/f14p0/716 | i1_LQ_HNbud_c75230/f1p4/1285 |
| i0_HQ_HNbud_c24129/f5p0/630 | i1_LQ_HNbud_c23193/f1p15/1671 | i1_LQ_HNbud_c70281/f1p2/1368 | i0_LQ_HNbud_c27460/f1p2/548 |
| i1_HQ_HNbud_c7251/f4p1/1564 | i1_LQ_HNbud_c22251/f1p4/1201 | i4_LQ_HNbud_c2989/f1p9/4673 | i2_LQ_HNbud_c6595/f1p9/2622 |
| i1_LQ_HNbud_c279516/f1p6/1009 | i1_HQ_HNbud_c18750/f6p4/1581 | i1_LQ_HNbud_c112913/f1p5/1244 | i1_HQ_HNbud_c57171/f5p0/1404 |
| i1_LQ_HNbud_c132564/f1p15/1799 | i3_LQ_HNbud_c12242/f1p1/3599 | i3_LQ_HNbud_c3512/f1p13/3938 | i0_HQ_HNbud_c12417/f3p27/589 |
| i1_HQ_HNbud_c220457/f4p8/1280 | i0_HQ_HNbud_c97995/f3p0/659 | i2_LQ_HNbud_c43272/f1p1/2365 | i4_LQ_HNbud_c2332/f1p0/4215 |
| i1_LQ_HNbud_c132035/f1p19/1934 | i2_LQ_HNbud_c15470/f1p2/2063 | i0_HQ_HNbud_c47098/f2p0/728 | i2_LQ_HNbud_c57004/f1p0/2133 |
| i2_HQ_HNbud_c61601/f2p1/2018 | i0_LQ_HNbud_c88478/f1p3/699 | i2_HQ_HNbud_c64531/f12p0/2145 | i2_LQ_HNbud_c5945/f1p0/2549 |
| i4_HQ_HNbud_c4379/f4p1/4610 | i2_HQ_HNbud_c33648/f3p1/2730 | i1_HQ_HNbud_c39079/f2p2/1456 | i3_HQ_HNbud_c1342/f3p0/3975 |
| i1_HQ_HNbud_c217363/f14p0/1310 | i4_LQ_HNbud_c1328/f1p0/4479 | i1_HQ_HNbud_c100205/f6p0/1367 | i3_LQ_HNbud_c13377/f1p0/3766 |
| i0_HQ_HNbud_c1957/f7p3/692 | i2_HQ_HNbud_c70509/f2p2/2528 | i2_LQ_HNbud_c63215/f1p10/2046 | i1_HQ_HNbud_c100304/f6p1/1869 |
| i2_HQ_HNbud_c11894/f2p0/2284 | i3_HQ_HNbud_c1965/f2p0/3483 | i0_LQ_HNbud_c12473/f1p1/771 | i1_LQ_HNbud_c170907/f1p3/1172 |
| i1_HQ_HNbud_c83165/f47p0/1094 | i1_LQ_HNbud_c199362/f1p0/1326 | i0_LQ_HNbud_c75723/f1p2/754 | i1_LQ_HNbud_c108554/f1p2/1117 |
| i1_LQ_HNbud_c68091/f1p0/1703 | i1_HQ_HNbud_c24406/f3p6/1370 | i1_HQ_HNbud_c16322/f2p11/1127 | i3_LQ_HNbud_c8360/f1p0/3983 |
| i0_HQ_HNbud_c2090/f4p2/664 | i1_LQ_HNbud_c9162/f1p0/1168 | i1_LQ_HNbud_c110025/f1p0/1517 | i5_LQ_HNbud_c281/f1p0/5523 |
| i0_LQ_HNbud_c94535/f1p0/721 | i1_LQ_HNbud_c105775/f1p16/1408 | i0_HQ_HNbud_c98091/f43p0/657 | i3_LQ_HNbud_c16360/f1p1/3758 |
| i2_LQ_HNbud_c24693/f1p9/2303 | i0_LQ_HNbud_c48354/f1p0/824 | i2_HQ_HNbud_c18009/f3p4/2423 | i3_LQ_HNbud_c4695/f1p1/3195 |
| i2_HQ_HNbud_c64526/f14p1/2827 | i5_LQ_HNbud_c156/f1p0/5768 | i1_LQ_HNbud_c278650/f1p3/1005 | i1_LQ_HNbud_c149434/f1p4/1453 |
| i0_LQ_HNbud_c13391/f1p4/700 | i2_LQ_HNbud_c66538/f2p2/2304 | i1_HQ_HNbud_c93301/f2p9/1475 | i3_LQ_HNbud_c10263/f1p0/3277 |
| i2_HQ_HNbud_c3382/f2p0/2917 | i2_LQ_HNbud_c24971/f1p0/2422 | i0_LQ_HNbud_c74695/f1p0/535 | i1_LQ_HNbud_c188746/f1p3/1055 |
| i3_HQ_HNbud_c1208/f3p0/3855 | i0_LQ_HNbud_c3213/f1p1/738 | i0_LQ_HNbud_c99631/f1p0/839 | i0_LQ_HNbud_c47894/f1p0/824 |
| i2_LQ_HNbud_c41293/f1p10/2414 | i1_LQ_HNbud_c14930/f1p3/1010 | i0_HQ_HNbud_c65721/f47p0/709 | i2_LQ_HNbud_c28505/f1p3/2498 |
| i3_HQ_HNbud_c658/f8p0/3808 | i1_LQ_HNbud_c85759/f1p27/1634 | i1_LQ_HNbud_c36680/f1p3/1126 | i1_LQ_HNbud_c68093/f2p1/1840 |
| i4_LQ_HNbud_c2193/f1p0/4508 | i0_HQ_HNbud_c97952/f5p0/690 | i7_LQ_HNbud_c808/f1p0/7577 | i0_LQ_HNbud_c84704/f1p0/387 |
| i0_HQ_HNbud_c98414/f30p0/700 | i0_HQ_HNbud_c2219/f3p2/858 | i0_HQ_HNbud_c39123/f3p0/657 | i1_HQ_HNbud_c17757/f4p4/1226 |
| i0_LQ_HNbud_c40352/f1p46/906 | i0_LQ_HNbud_c27856/f2p10/590 | i1_LQ_HNbud_c67885/f2p1/1379 | i4_LQ_HNbud_c2748/f1p5/4502 |
| i1_HQ_HNbud_c9399/f2p0/1122 | i1_HQ_HNbud_c191018/f23p19/1607 | i0_HQ_HNbud_c66681/f2p0/794 | i0_HQ_HNbud_c98450/f58p0/679 |
| i3_LQ_HNbud_c16047/f1p14/3041 | i1_LQ_HNbud_c91506/f1p24/1990 | i0_LQ_HNbud_c21840/f1p10/908 | i1_HQ_HNbud_c157323/f11p8/1738 |
| i1_HQ_HNbud_c38070/f6p2/1780 | i1_HQ_HNbud_c71467/f4p5/2023 | i0_HQ_HNbud_c40487/f2p20/600 | i2_HQ_HNbud_c3564/f5p1/2783 |
| i0_LQ_HNbud_c4469/f1p0/782 | i1_HQ_HNbud_c264605/f86p19/1639 | i2_LQ_HNbud_c10794/f1p1/2730 | i0_LQ_HNbud_c96084/f1p0/665 |
| i1_HQ_HNbud_c192055/f32p8/1376 | i0_HQ_HNbud_c2077/f2p5/598 | i2_LQ_HNbud_c36219/f1p1/2682 | i1_HQ_HNbud_c196473/f2p1/1987 |
| i2_LQ_HNbud_c20101/f1p3/2057 | i0_HQ_HNbud_c66437/f3p89/736 | i0_LQ_HNbud_c7830/f1p6/504 | i1_LQ_HNbud_c91558/f1p9/1303 |
| i1_HQ_HNbud_c125994/f3p1/1151 | i2_HQ_HNbud_c66198/f56p8/2364 | i1_HQ_HNbud_c7426/f3p8/1867 | i1_LQ_HNbud_c73619/f1p3/1877 |
| i3_HQ_HNbud_c16648/f14p0/3423 | i0_LQ_HNbud_c10993/f1p0/862 | i3_LQ_HNbud_c7923/f1p1/3604 | i2_HQ_HNbud_c18101/f2p2/2676 |
| i0_LQ_HNbud_c11515/f1p5/710 | i2_LQ_HNbud_c3349/f1p1/2567 | i0_HQ_HNbud_c382/f23p0/555 | i3_LQ_HNbud_c12681/f1p2/3596 |
| i2_LQ_HNbud_c33957/f1p8/2221 | i2_HQ_HNbud_c26333/f2p5/2796 | i2_LQ_HNbud_c1725/f3p0/2269 | i0_LQ_HNbud_c50095/f1p3/717 |
| i2_LQ_HNbud_c36424/f1p21/2408 | i0_LQ_HNbud_c39870/f2p1/798 | i1_LQ_HNbud_c98508/f1p1/1297 | i2_LQ_HNbud_c40021/f1p2/2857 |
| i2_HQ_HNbud_c4208/f5p6/2734 | i1_LQ_HNbud_c14681/f1p1/1085 | i0_LQ_HNbud_c68449/f2p0/711 | i5_LQ_HNbud_c1732/f1p2/5551 |
| i1_HQ_HNbud_c16210/f27p26/1793 | i0_LQ_HNbud_c75117/f1p0/725 | i0_LQ_HNbud_c50247/f1p1/701 | i1_LQ_HNbud_c249088/f1p0/1753 |
| i0_HQ_HNbud_c98466/f11p13/529 | i1_HQ_HNbud_c262568/f2p0/1016 | i2_HQ_HNbud_c49705/f4p2/2207 | i0_LQ_HNbud_c69845/f1p0/850 |
| i1_LQ_HNbud_c225494/f1p11/1106 | i0_LQ_HNbud_c27568/f1p3/497 | i1_LQ_HNbud_c209968/f1p22/1028 | i0_HQ_HNbud_c12/f28p0/612 |
| i2_LQ_HNbud_c52155/f1p2/2715 | i0_HQ_HNbud_c8542/f2p25/800 | i1_LQ_HNbud_c74412/f1p0/1690 | i0_LQ_HNbud_c4893/f4p0/684 |
| i1_HQ_HNbud_c146550/f3p1/1965 | i3_LQ_HNbud_c7475/f1p0/3942 | i0_HQ_HNbud_c8765/f3p0/620 | i2_LQ_HNbud_c35719/f1p8/2873 |
| i0_LQ_HNbud_c20730/f1p0/990 | i1_LQ_HNbud_c3318/f3p2/1860 | i1_LQ_HNbud_c175250/f1p8/1697 | i0_HQ_HNbud_c27703/f7p1/865 |
| i3_LQ_HNbud_c13965/f1p2/3197 | i1_LQ_HNbud_c213653/f1p0/1016 | i0_HQ_HNbud_c1802/f5p2/698 | i2_HQ_HNbud_c23972/f3p7/2374 |
| i0_HQ_HNbud_c169/f24p0/909 | i1_LQ_HNbud_c180388/f1p3/1829 | i1_HQ_HNbud_c1995/f9p1/1772 | i2_LQ_HNbud_c54757/f1p18/2806 |
| i3_LQ_HNbud_c15246/f1p0/3039 | i0_LQ_HNbud_c20428/f1p14/854 | i1_HQ_HNbud_c30901/f6p1/1174 | i1_LQ_HNbud_c24240/f1p7/1807 |
| i1_HQ_HNbud_c101305/f20p2/1275 | i2_LQ_HNbud_c35054/f1p2/2271 | i1_HQ_HNbud_c8471/f5p0/1257 | i0_HQ_HNbud_c893/f21p0/997 |
| i2_HQ_HNbud_c65472/f8p3/2135 | i0_LQ_HNbud_c47843/f1p0/742 | i0_HQ_HNbud_c2347/f5p0/655 | i1_HQ_HNbud_c148989/f4p1/1470 |
| i0_HQ_HNbud_c26198/f3p2/621 | i0_HQ_HNbud_c12836/f3p0/598 | i0_LQ_HNbud_c28082/f1p0/783 | i1_LQ_HNbud_c231973/f1p1/1936 |
| i1_LQ_HNbud_c171528/f1p13/1431 | i4_LQ_HNbud_c4091/f1p0/4044 | i1_HQ_HNbud_c10830/f3p1/1575 | i0_LQ_HNbud_c1937/f9p8/648 |
| i1_LQ_HNbud_c91152/f1p6/1453 | i0_HQ_HNbud_c20147/f2p3/846 | i1_HQ_HNbud_c10989/f3p1/1285 | i4_LQ_HNbud_c2487/f1p1/4684 |
| i3_HQ_HNbud_c16912/f4p0/3440 | i0_LQ_HNbud_c20152/f1p0/813 | i2_LQ_HNbud_c55934/f1p2/2621 | i1_HQ_HNbud_c264056/f4p3/1135 |
| i1_LQ_HNbud_c112334/f1p10/1332 | i3_LQ_HNbud_c3231/f1p1/3610 | i1_LQ_HNbud_c178275/f1p2/1789 | i0_LQ_HNbud_c8163/f1p0/603 |
| i1_LQ_HNbud_c228671/f1p1/1776 | i1_HQ_HNbud_c2760/f2p353/1697 | i1_LQ_HNbud_c278307/f1p55/1080 | i1_HQ_HNbud_c55922/f3p0/1378 |
| i3_LQ_HNbud_c1697/f2p3/3690 | i0_HQ_HNbud_c28850/f2p0/680 | i0_LQ_HNbud_c83254/f1p0/720 | i0_HQ_HNbud_c97833/f7p0/791 |
| i0_HQ_HNbud_c2054/f9p0/534 | i0_LQ_HNbud_c24427/f1p0/775 | i4_LQ_HNbud_c1421/f1p0/4181 | i1_LQ_HNbud_c23754/f1p17/1735 |
| i1_LQ_HNbud_c174377/f1p0/1844 | i0_LQ_HNbud_c8782/f1p2/849 | i0_LQ_HNbud_c26926/f1p0/804 | i2_LQ_HNbud_c31726/f2p4/2046 |
| i1_LQ_HNbud_c4318/f1p4/1278 | i1_LQ_HNbud_c89335/f1p13/1277 | i1_HQ_HNbud_c244804/f6p5/1123 | i1_LQ_HNbud_c242142/f1p3/1036 |
| i3_HQ_HNbud_c1584/f2p3/3691 | i1_LQ_HNbud_c29956/f1p19/1076 | i1_LQ_HNbud_c35123/f1p4/1299 | i0_HQ_HNbud_c18503/f2p0/655 |
| i2_HQ_HNbud_c6329/f2p1/2571 | i2_LQ_HNbud_c41124/f1p726/2604 | i0_HQ_HNbud_c38297/f30p2/695 | i0_LQ_HNbud_c77103/f1p0/814 |
| i1_HQ_HNbud_c1309/f36p9/1643 | i0_LQ_HNbud_c31624/f2p0/908 | i3_LQ_HNbud_c14061/f1p0/3863 | i1_LQ_HNbud_c72834/f1p15/1320 |
| i1_LQ_HNbud_c13252/f2p6/1890 | i1_LQ_HNbud_c40230/f1p2/1390 | i2_LQ_HNbud_c39108/f1p4/2247 | i0_LQ_HNbud_c71964/f1p1/855 |
| i1_LQ_HNbud_c201508/f1p2/1115 | i1_LQ_HNbud_c203685/f1p17/1773 | i0_HQ_HNbud_c54944/f2p2/811 | i3_LQ_HNbud_c15538/f1p0/3010 |
| i1_HQ_HNbud_c235102/f2p8/1066 | i2_LQ_HNbud_c71566/f1p0/2011 | i1_LQ_HNbud_c72774/f1p0/1242 | i1_LQ_HNbud_c149645/f1p4/1495 |
| i2_LQ_HNbud_c38006/f1p8/2829 | i2_LQ_HNbud_c25325/f1p5/2250 | i1_LQ_HNbud_c15111/f1p0/1018 | i3_LQ_HNbud_c13915/f1p1/3348 |
| i1_HQ_HNbud_c88501/f3p2/1574 | i4_LQ_HNbud_c630/f1p0/4549 | i2_LQ_HNbud_c66904/f1p1/2775 | i4_HQ_HNbud_c4360/f13p0/4359 |
| i2_LQ_HNbud_c42090/f1p2/2517 | i0_LQ_HNbud_c7113/f2p7/745 | i4_LQ_HNbud_c1197/f1p0/4134 | i1_LQ_HNbud_c54170/f1p0/1937 |
| i0_HQ_HNbud_c4284/f2p0/886 | i2_LQ_HNbud_c12425/f1p1/2540 | i5_LQ_HNbud_c260/f1p0/5305 | i1_HQ_HNbud_c267789/f2p18/1674 |
| i2_LQ_HNbud_c46383/f1p1/2050 | i2_LQ_HNbud_c59026/f1p1/2132 | i0_HQ_HNbud_c26374/f3p3/625 | i0_LQ_HNbud_c93509/f1p0/532 |
| i1_HQ_HNbud_c237239/f4p1/1269 | i4_LQ_HNbud_c3290/f1p52/4326 | i1_LQ_HNbud_c224468/f1p4/1328 | i2_LQ_HNbud_c39925/f1p4/2326 |
| i2_HQ_HNbud_c64682/f4p2/2815 | i2_LQ_HNbud_c39122/f1p2/2551 | i3_HQ_HNbud_c1735/f2p1/3229 | i3_LQ_HNbud_c12858/f1p6/3955 |
| i0_HQ_HNbud_c27187/f2p0/819 | i1_LQ_HNbud_c39503/f2p2/1622 | i1_LQ_HNbud_c40480/f1p6/1245 | i1_LQ_HNbud_c34306/f1p1/1949 |
| i2_HQ_HNbud_c8397/f2p3/2644 | i1_LQ_HNbud_c24430/f1p0/1722 | i1_HQ_HNbud_c18518/f2p5/1756 | i0_LQ_HNbud_c12109/f2p0/774 |
| i1_LQ_HNbud_c27705/f1p0/1300 | i2_LQ_HNbud_c62651/f1p5/2043 | i2_LQ_HNbud_c56190/f1p3/2427 | i2_LQ_HNbud_c33590/f1p4/2431 |
| i0_LQ_HNbud_c72757/f1p13/338 | i2_LQ_HNbud_c52550/f1p1/2900 | i3_LQ_HNbud_c14206/f1p0/3494 | i1_LQ_HNbud_c66014/f2p1/1377 |
| i1_LQ_HNbud_c155884/f1p1/1542 | i0_LQ_HNbud_c65592/f1p0/803 | i1_LQ_HNbud_c152606/f1p2/1290 | i1_LQ_HNbud_c148974/f1p1/1699 |
| i0_LQ_HNbud_c97877/f2p1/810 | i4_LQ_HNbud_c1299/f1p2/4465 | i1_LQ_HNbud_c89991/f1p0/1826 | i4_LQ_HNbud_c2861/f1p2/4507 |
| i0_HQ_HNbud_c66664/f2p0/831 | i1_HQ_HNbud_c93417/f4p3/1647 | i0_LQ_HNbud_c35013/f1p1/689 | i3_LQ_HNbud_c15664/f1p0/3023 |
| i4_HQ_HNbud_c275/f2p0/4760 | i1_HQ_HNbud_c20600/f7p1/1245 | i0_HQ_HNbud_c97851/f3p0/614 | i0_LQ_HNbud_c20374/f1p4/992 |
| i1_LQ_HNbud_c74006/f1p1/1983 | i1_LQ_HNbud_c231912/f1p4/1476 | i1_LQ_HNbud_c252071/f1p17/1708 | i1_LQ_HNbud_c228464/f1p0/1959 |
| i1_LQ_HNbud_c7500/f1p0/1768 | i1_HQ_HNbud_c263538/f2p6/1235 | i0_HQ_HNbud_c18542/f2p0/916 | i1_HQ_HNbud_c46456/f24p6/1524 |
| i0_LQ_HNbud_c39708/f3p0/721 | i3_LQ_HNbud_c11547/f1p0/3695 | i0_HQ_HNbud_c64581/f15p1/659 | i0_HQ_HNbud_c6314/f3p1/896 |
| i1_LQ_HNbud_c40520/f1p2/1778 | i1_LQ_HNbud_c78769/f1p2/1070 | i1_HQ_HNbud_c32551/f2p15/1148 | i0_HQ_HNbud_c97938/f11p0/641 |
| i0_LQ_HNbud_c73471/f1p35/566 | i1_LQ_HNbud_c42468/f2p4/1306 | i0_HQ_HNbud_c50284/f2p22/634 | i1_LQ_HNbud_c54887/f1p0/1387 |
| i1_HQ_HNbud_c17679/f4p1/1687 | i3_LQ_HNbud_c4362/f1p0/3186 | i1_HQ_HNbud_c1816/f4p1/1786 | i2_LQ_HNbud_c45557/f1p0/2023 |
| i1_LQ_HNbud_c88755/f1p5/1574 | i1_LQ_HNbud_c146572/f1p3/1564 | i4_LQ_HNbud_c1126/f1p5/4183 | i1_LQ_HNbud_c21924/f1p6/1993 |
| i0_LQ_HNbud_c10514/f1p8/640 | i1_LQ_HNbud_c5787/f1p2/1864 | i0_HQ_HNbud_c99030/f25p0/846 | i1_LQ_HNbud_c205410/f1p4/1468 |
| i1_HQ_HNbud_c166353/f4p4/1153 | i0_HQ_HNbud_c1672/f10p2/779 | i2_LQ_HNbud_c34415/f1p5/2282 | i0_HQ_HNbud_c23970/f3p1/961 |
| i0_LQ_HNbud_c77892/f1p0/807 | i1_LQ_HNbud_c5853/f1p1/1519 | i2_LQ_HNbud_c4504/f1p3/2570 | i1_LQ_HNbud_c19827/f1p0/1464 |
| i3_LQ_HNbud_c11159/f1p1/3648 | i0_LQ_HNbud_c71978/f1p2/537 | i1_LQ_HNbud_c171491/f1p0/1958 | i2_LQ_HNbud_c21766/f2p9/2181 |
| i1_LQ_HNbud_c67970/f2p1/1928 | i0_HQ_HNbud_c64748/f5p3/667 | i1_LQ_HNbud_c132897/f1p2/1912 | i2_HQ_HNbud_c48135/f3p1/2090 |
| i0_HQ_HNbud_c1479/f9p0/952 | i1_LQ_HNbud_c26824/f1p1/1856 | i1_HQ_HNbud_c220992/f2p30/1744 | i3_HQ_HNbud_c15242/f2p2/3042 |
| i4_LQ_HNbud_c3664/f1p0/4489 | i1_LQ_HNbud_c57400/f1p14/1447 | i2_LQ_HNbud_c41447/f1p9/2963 | i0_LQ_HNbud_c75666/f1p0/733 |
| i1_LQ_HNbud_c132359/f1p0/1830 | i1_LQ_HNbud_c149313/f1p10/1813 | i1_HQ_HNbud_c62811/f46p9/1570 | i1_LQ_HNbud_c25662/f1p0/1480 |
| i2_LQ_HNbud_c30363/f1p1/2038 | i1_LQ_HNbud_c5156/f1p35/1606 | i1_HQ_HNbud_c20742/f2p11/1388 | i0_LQ_HNbud_c28530/f1p0/845 |
| i1_HQ_HNbud_c264861/f4p1/1553 | i2_LQ_HNbud_c51217/f1p5/2791 | i0_LQ_HNbud_c6418/f1p0/968 | i1_HQ_HNbud_c55677/f9p2/1995 |
| i3_LQ_HNbud_c7466/f1p1/3911 | i1_HQ_HNbud_c243158/f4p1/1469 | i2_LQ_HNbud_c25755/f1p2/2143 | i2_LQ_HNbud_c58490/f1p8/2340 |
| i1_LQ_HNbud_c52151/f1p133/1237 | i5_LQ_HNbud_c1181/f1p19/5248 | i3_LQ_HNbud_c14265/f1p1/3317 | i2_LQ_HNbud_c50200/f1p12/2500 |
| i1_LQ_HNbud_c41571/f1p1/2073 | i2_LQ_HNbud_c56813/f1p0/2370 | i0_LQ_HNbud_c4947/f4p4/906 | i2_LQ_HNbud_c12311/f1p9/2131 |
| i2_LQ_HNbud_c39795/f1p1/2149 | i1_LQ_HNbud_c155594/f1p0/1580 | i0_HQ_HNbud_c18724/f3p0/827 | i0_LQ_HNbud_c96561/f1p0/846 |
| i3_LQ_HNbud_c18604/f1p0/3002 | i0_LQ_HNbud_c1908/f2p1/974 | i1_LQ_HNbud_c105795/f1p13/1196 | i2_LQ_HNbud_c28503/f1p5/2860 |
| i1_HQ_HNbud_c23225/f2p2/1823 | i1_LQ_HNbud_c250445/f1p0/1211 | i2_HQ_HNbud_c34629/f2p3/2209 | i1_HQ_HNbud_c246727/f4p6/1526 |
| i0_HQ_HNbud_c98418/f16p2/754 | i1_HQ_HNbud_c101073/f22p10/1494 | i2_LQ_HNbud_c43541/f1p1/2251 | i1_HQ_HNbud_c23056/f2p1/1242 |
| i0_HQ_HNbud_c1507/f14p0/836 | i1_LQ_HNbud_c41804/f1p1/1593 | i0_LQ_HNbud_c2680/f1p4/901 | i2_HQ_HNbud_c4503/f2p1/2876 |
| i1_LQ_HNbud_c21469/f1p1/1295 | i2_HQ_HNbud_c66515/f2p0/2207 | i0_HQ_HNbud_c1593/f3p4/866 | i1_HQ_HNbud_c23181/f2p9/1996 |
| i2_LQ_HNbud_c73580/f1p1/1966 | i1_LQ_HNbud_c91976/f1p4/1521 | i1_HQ_HNbud_c234163/f4p2/1072 | i2_HQ_HNbud_c45272/f2p1/2014 |
| i0_HQ_HNbud_c98303/f16p0/544 | i3_LQ_HNbud_c12208/f1p4/3789 | i1_HQ_HNbud_c225799/f2p1/1378 | i0_LQ_HNbud_c12969/f1p0/749 |
| i2_LQ_HNbud_c30888/f1p0/2035 | i1_HQ_HNbud_c13404/f2p3/1291 | i1_HQ_HNbud_c2552/f11p6/1452 | i1_HQ_HNbud_c36972/f6p1/1219 |
| i1_HQ_HNbud_c11916/f7p2/1846 | i1_HQ_HNbud_c245301/f7p1/1030 | i2_LQ_HNbud_c47089/f1p6/2047 | i1_LQ_HNbud_c22238/f4p1/1453 |
| i1_LQ_HNbud_c27325/f1p10/1470 | i1_LQ_HNbud_c55942/f1p3/1965 | i1_HQ_HNbud_c6867/f21p10/1681 | i3_HQ_HNbud_c1311/f3p5/3199 |
| i0_HQ_HNbud_c2117/f5p1/816 | i0_LQ_HNbud_c31243/f1p0/683 | i2_LQ_HNbud_c57277/f1p7/2731 | i1_HQ_HNbud_c265663/f16p9/1466 |
| i1_LQ_HNbud_c88246/f2p1/1805 | i3_LQ_HNbud_c9840/f1p6/3103 | i1_LQ_HNbud_c171556/f1p1/1538 | i1_LQ_HNbud_c278860/f1p1/1015 |
| i0_HQ_HNbud_c5055/f5p0/412 | i2_LQ_HNbud_c6140/f1p3/2721 | i1_LQ_HNbud_c249768/f1p6/1518 | i1_LQ_HNbud_c253859/f1p11/1234 |
| i1_LQ_HNbud_c127036/f1p0/1872 | i0_HQ_HNbud_c55927/f7p0/806 | i1_LQ_HNbud_c39169/f1p1/1684 | i0_HQ_HNbud_c493/f14p0/517 |
| i4_LQ_HNbud_c1098/f1p0/4270 | i1_HQ_HNbud_c65613/f7p0/1292 | i1_LQ_HNbud_c56133/f1p4/1702 | i1_HQ_HNbud_c166153/f15p12/1404 |
| i2_LQ_HNbud_c21001/f1p3/2285 | i1_HQ_HNbud_c265134/f5p8/1238 | i4_LQ_HNbud_c708/f1p0/4482 | i1_LQ_HNbud_c257519/f1p2/1066 |
| i1_LQ_HNbud_c139278/f1p5/1960 | i5_HQ_HNbud_c99/f2p0/5234 | i0_LQ_HNbud_c5698/f1p7/797 | i1_LQ_HNbud_c34376/f6p10/1297 |
| i2_LQ_HNbud_c24258/f1p3/2410 | i1_LQ_HNbud_c39400/f1p2/1563 | i1_LQ_HNbud_c221255/f1p2/1334 | i1_LQ_HNbud_c53968/f1p5/1571 |
| i2_LQ_HNbud_c8845/f1p4/2013 | i0_LQ_HNbud_c19162/f1p6/813 | i0_LQ_HNbud_c79785/f1p0/679 | i2_LQ_HNbud_c27141/f1p1/2978 |
| i2_LQ_HNbud_c68976/f1p2/2309 | i1_LQ_HNbud_c248708/f1p5/1568 | i1_LQ_HNbud_c59965/f1p10/1040 | i1_LQ_HNbud_c226128/f1p2/1486 |
| i1_HQ_HNbud_c86058/f4p2/1613 | i0_HQ_HNbud_c29575/f3p0/548 | i0_HQ_HNbud_c2972/f3p4/830 | i0_HQ_HNbud_c18549/f2p15/520 |
| i1_HQ_HNbud_c22337/f2p0/1856 | i1_HQ_HNbud_c255149/f5p3/1030 | i3_HQ_HNbud_c16857/f5p0/3180 | i2_LQ_HNbud_c52367/f1p0/2342 |
| i0_LQ_HNbud_c4329/f1p0/901 | i1_HQ_HNbud_c39277/f4p2/1533 | i4_LQ_HNbud_c4175/f1p1/4055 | i0_HQ_HNbud_c4925/f3p0/492 |
| i1_LQ_HNbud_c163091/f1p3/1449 | i3_LQ_HNbud_c11941/f1p4/3207 | i2_LQ_HNbud_c53713/f1p0/2113 | i2_HQ_HNbud_c34950/f2p20/2556 |
| i1_HQ_HNbud_c3813/f4p2/1840 | i2_LQ_HNbud_c33345/f3p0/2299 | i3_LQ_HNbud_c8077/f1p6/3452 | i2_LQ_HNbud_c6125/f2p3/2467 |
| i2_LQ_HNbud_c14460/f1p13/2528 | i1_HQ_HNbud_c17835/f20p10/1761 | i1_LQ_HNbud_c6429/f1p2/1505 | i2_LQ_HNbud_c35178/f2p3/2529 |
| i0_LQ_HNbud_c79137/f1p0/705 | i1_LQ_HNbud_c1377/f24p5/1670 | i0_LQ_HNbud_c13381/f1p4/694 | i1_HQ_HNbud_c11522/f9p1/1336 |
| i4_LQ_HNbud_c2523/f1p0/4074 | i2_LQ_HNbud_c6223/f1p2/2464 | i1_LQ_HNbud_c196325/f1p4/1178 | i1_LQ_HNbud_c11388/f1p3/1293 |
| i0_HQ_HNbud_c37973/f31p13/725 | i1_LQ_HNbud_c150191/f1p6/1800 | i1_LQ_HNbud_c222540/f1p2/1625 | i1_LQ_HNbud_c174343/f1p4/1514 |
| i0_LQ_HNbud_c53477/f1p13/865 | i0_HQ_HNbud_c1117/f9p0/503 | i0_LQ_HNbud_c99513/f3p0/750 | i0_LQ_HNbud_c28511/f1p0/863 |
| i2_LQ_HNbud_c18635/f1p3/2834 | i1_LQ_HNbud_c42507/f2p3/1249 | i0_HQ_HNbud_c66270/f5p1/954 | i2_HQ_HNbud_c61767/f2p0/2040 |
| i2_HQ_HNbud_c7279/f4p1/2385 | i0_HQ_HNbud_c1896/f6p2/529 | i1_LQ_HNbud_c56377/f1p1/1481 | i1_HQ_HNbud_c2263/f19p6/1342 |
| i0_LQ_HNbud_c2902/f1p0/837 | i0_LQ_HNbud_c44513/f1p0/865 | i2_LQ_HNbud_c56293/f1p3/2768 | i3_LQ_HNbud_c16363/f1p0/3050 |
| i1_HQ_HNbud_c27309/f2p0/1620 | i1_LQ_HNbud_c74264/f1p31/1752 | i2_HQ_HNbud_c9260/f2p0/2800 | i2_HQ_HNbud_c15082/f4p0/2083 |
| i1_HQ_HNbud_c263245/f12p2/1146 | i1_LQ_HNbud_c67903/f1p1/1751 | i1_LQ_HNbud_c195824/f1p8/1297 | i1_LQ_HNbud_c159260/f3p1/1063 |
| i1_LQ_HNbud_c37304/f3p0/1213 | i1_LQ_HNbud_c67657/f1p4/1223 | i1_LQ_HNbud_c14705/f1p6/1073 | i0_HQ_HNbud_c6933/f6p2/989 |
| i0_LQ_HNbud_c46597/f2p0/812 | i0_HQ_HNbud_c2134/f2p2/748 | i2_HQ_HNbud_c12282/f3p1/2771 | i2_LQ_HNbud_c53861/f1p3/2872 |
| i2_LQ_HNbud_c14663/f1p2/2625 | i0_LQ_HNbud_c38435/f5p20/747 | i1_HQ_HNbud_c92881/f3p1/1718 | i2_HQ_HNbud_c45136/f2p0/2029 |
| i3_LQ_HNbud_c9152/f1p2/3256 | i2_LQ_HNbud_c12346/f1p7/2438 | i1_LQ_HNbud_c110492/f1p0/1721 | i1_LQ_HNbud_c34819/f1p2/1242 |
| i0_LQ_HNbud_c74044/f1p0/810 | i0_LQ_HNbud_c77582/f1p0/965 | i2_LQ_HNbud_c16195/f1p0/2077 | i2_LQ_HNbud_c37536/f1p4/2134 |
| i2_LQ_HNbud_c6394/f1p7/2641 | i4_LQ_HNbud_c381/f1p0/4383 | i1_HQ_HNbud_c10562/f13p10/1624 | i1_HQ_HNbud_c2826/f4p2/1470 |
| i0_LQ_HNbud_c3566/f4p3/546 | i4_LQ_HNbud_c517/f1p0/4164 | i3_LQ_HNbud_c7409/f1p3/3663 | i2_LQ_HNbud_c56488/f1p20/3105 |
| i0_LQ_HNbud_c19685/f1p0/666 | i2_LQ_HNbud_c54884/f1p1/2244 | i1_LQ_HNbud_c262610/f1p4/1029 | i3_HQ_HNbud_c1964/f2p2/3385 |
| i1_HQ_HNbud_c5558/f3p0/1730 | i2_LQ_HNbud_c9436/f1p7/2221 | i1_HQ_HNbud_c3736/f16p14/1586 | i1_LQ_HNbud_c60826/f1p11/1797 |
| i1_LQ_HNbud_c260290/f1p12/1594 | i1_HQ_HNbud_c11171/f6p2/1663 | i4_LQ_HNbud_c658/f1p0/4165 | i0_HQ_HNbud_c4414/f2p20/616 |
| i1_LQ_HNbud_c106072/f1p18/1264 | i3_LQ_HNbud_c3866/f1p15/3246 | i2_LQ_HNbud_c25598/f1p4/2204 | i0_LQ_HNbud_c19067/f2p12/518 |
| i1_LQ_HNbud_c26521/f1p5/1999 | i1_LQ_HNbud_c66748/f1p4/1595 | i1_LQ_HNbud_c12422/f1p2/1711 | i1_LQ_HNbud_c98492/f1p7/926 |
| i1_HQ_HNbud_c20483/f3p3/1455 | i2_LQ_HNbud_c55220/f1p4/2099 | i0_LQ_HNbud_c81068/f1p0/652 | i1_LQ_HNbud_c163376/f1p1/1040 |
| i1_LQ_HNbud_c40689/f1p0/1329 | i2_LQ_HNbud_c39590/f1p2/2616 | i1_LQ_HNbud_c152864/f1p11/1350 | i2_HQ_HNbud_c19242/f3p1/2263 |
| i2_LQ_HNbud_c22091/f1p0/2593 | i2_LQ_HNbud_c60005/f1p1/2830 | i1_LQ_HNbud_c109398/f2p3/1854 | i0_LQ_HNbud_c4212/f3p3/738 |
| i2_LQ_HNbud_c21959/f1p3/2383 | i1_LQ_HNbud_c227058/f1p22/1948 | i3_LQ_HNbud_c1747/f2p0/3207 | i1_LQ_HNbud_c202444/f1p1/1761 |
| i1_HQ_HNbud_c226180/f4p8/1506 | i1_HQ_HNbud_c53741/f3p6/1955 | i2_LQ_HNbud_c2446/f1p12/2124 | i2_LQ_HNbud_c53288/f1p1/2445 |
| i2_LQ_HNbud_c41847/f1p15/2645 | i4_LQ_HNbud_c2447/f1p0/4420 | i1_LQ_HNbud_c172723/f1p0/1472 | i3_LQ_HNbud_c17686/f1p3/3380 |
| i0_LQ_HNbud_c75274/f1p7/775 | i2_HQ_HNbud_c36889/f2p3/2750 | i2_LQ_HNbud_c25165/f1p5/2474 | i1_LQ_HNbud_c203384/f1p13/1171 |
| i2_LQ_HNbud_c11106/f1p5/2415 | i0_LQ_HNbud_c18750/f2p2/712 | i2_LQ_HNbud_c39975/f1p0/2643 | i1_HQ_HNbud_c41131/f3p2/1632 |
| i0_HQ_HNbud_c39475/f3p0/580 | i2_LQ_HNbud_c11749/f1p7/2465 | i1_LQ_HNbud_c50282/f1p1/1423 | i2_LQ_HNbud_c40173/f1p3/2333 |
| i0_LQ_HNbud_c44135/f1p8/982 | i2_LQ_HNbud_c35568/f1p3/2289 | i0_HQ_HNbud_c45082/f2p1/826 | i2_HQ_HNbud_c21506/f3p9/2101 |
| i0_HQ_HNbud_c22552/f2p0/974 | i1_LQ_HNbud_c13901/f1p0/1574 | i2_HQ_HNbud_c29682/f2p1/2020 | i1_LQ_HNbud_c204132/f1p1/1596 |
| i1_HQ_HNbud_c190883/f2p1/1230 | i3_LQ_HNbud_c4473/f1p18/3737 | i0_LQ_HNbud_c70806/f1p4/714 | i1_HQ_HNbud_c53984/f4p9/1871 |
| i2_HQ_HNbud_c1058/f19p16/2485 | i3_LQ_HNbud_c1477/f1p0/3726 | i2_LQ_HNbud_c4746/f1p3/2140 | i1_LQ_HNbud_c253483/f1p0/1205 |
| i3_HQ_HNbud_c1950/f2p12/3924 | i0_LQ_HNbud_c5164/f1p1/807 | i0_LQ_HNbud_c10498/f1p0/683 | i1_LQ_HNbud_c227857/f1p0/1401 |
| i2_LQ_HNbud_c27188/f1p0/2204 | i1_LQ_HNbud_c36071/f1p1/1088 | i1_LQ_HNbud_c126893/f1p25/1352 | i2_LQ_HNbud_c9855/f1p0/2377 |
| i3_LQ_HNbud_c12911/f1p5/3478 | i1_LQ_HNbud_c150363/f1p0/1404 | i0_LQ_HNbud_c44949/f1p9/935 | i2_LQ_HNbud_c5518/f1p2/2243 |
| i2_LQ_HNbud_c6653/f1p1/2677 | i1_LQ_HNbud_c152236/f1p2/1194 | i0_HQ_HNbud_c99097/f92p0/814 | i2_LQ_HNbud_c36406/f1p2/2658 |
| i2_LQ_HNbud_c27008/f1p6/2271 | i2_HQ_HNbud_c2095/f3p0/2335 | i1_LQ_HNbud_c223132/f1p2/1634 | i0_LQ_HNbud_c99395/f6p0/607 |
| i1_LQ_HNbud_c34122/f1p0/1571 | i1_LQ_HNbud_c72885/f1p1/1696 | i1_HQ_HNbud_c21322/f6p8/1402 | i1_HQ_HNbud_c230408/f2p1/1575 |
| i1_LQ_HNbud_c92263/f1p2/1679 | i1_LQ_HNbud_c73925/f1p117/1353 | i1_LQ_HNbud_c248170/f1p2/1412 | i1_LQ_HNbud_c128302/f1p3/1882 |
| i1_LQ_HNbud_c106597/f1p1/1282 | i1_LQ_HNbud_c5075/f1p65/1606 | i3_LQ_HNbud_c5849/f1p7/3652 | i2_LQ_HNbud_c22902/f1p0/2392 |
| i2_LQ_HNbud_c36439/f1p1/2456 | i2_LQ_HNbud_c26322/f1p3/2446 | i0_HQ_HNbud_c39683/f3p9/798 | i2_LQ_HNbud_c53835/f1p23/2969 |
| i1_HQ_HNbud_c265461/f3p13/1054 | i0_LQ_HNbud_c21794/f1p0/884 | i0_LQ_HNbud_c77848/f1p0/834 | i1_HQ_HNbud_c190837/f13p6/1359 |
| i0_HQ_HNbud_c38728/f29p14/981 | i2_HQ_HNbud_c20344/f3p12/2478 | i1_HQ_HNbud_c55961/f2p3/1572 | i1_LQ_HNbud_c26487/f1p0/1258 |
| i1_LQ_HNbud_c77392/f2p1/1132 | i0_LQ_HNbud_c3968/f1p2/791 | i1_LQ_HNbud_c112751/f1p8/1557 | i1_HQ_HNbud_c12146/f2p43/1592 |
| i2_LQ_HNbud_c11757/f1p0/2334 | i1_LQ_HNbud_c35736/f1p2/1708 | i1_HQ_HNbud_c20036/f11p0/1415 | i1_LQ_HNbud_c15311/f1p3/1038 |
| i0_HQ_HNbud_c105400/f2p0/488 | i0_HQ_HNbud_c29827/f2p0/987 | i1_HQ_HNbud_c24257/f3p7/1371 | i2_LQ_HNbud_c8258/f1p3/2269 |
| i1_HQ_HNbud_c120377/f3p1/1454 | i1_LQ_HNbud_c108989/f2p0/1448 | i0_HQ_HNbud_c2145/f17p2/799 | i0_HQ_HNbud_c98401/f62p0/694 |
| i1_LQ_HNbud_c9429/f1p3/1605 | i0_LQ_HNbud_c9929/f1p0/979 | i1_HQ_HNbud_c40960/f11p8/1769 | i1_LQ_HNbud_c210527/f1p4/1037 |
| i2_HQ_HNbud_c65037/f36p7/2570 | i1_LQ_HNbud_c149915/f1p8/1762 | i2_LQ_HNbud_c40982/f1p0/2399 | i1_HQ_HNbud_c46389/f31p5/1653 |
| i3_LQ_HNbud_c7950/f1p0/3549 | i1_HQ_HNbud_c265673/f5p6/1850 | i1_LQ_HNbud_c127089/f1p18/1591 | i1_HQ_HNbud_c23572/f2p0/1446 |
| i2_LQ_HNbud_c5172/f1p4/2868 | i1_LQ_HNbud_c204278/f1p22/1721 | i3_LQ_HNbud_c15272/f1p3/3065 | i1_LQ_HNbud_c175148/f1p10/1512 |
| i0_HQ_HNbud_c1698/f5p130/862 | i0_LQ_HNbud_c29534/f1p0/985 | i0_LQ_HNbud_c48100/f1p0/946 | i2_LQ_HNbud_c38207/f1p5/2505 |
| i2_HQ_HNbud_c19518/f5p1/2204 | i2_HQ_HNbud_c22350/f4p7/2157 | i1_HQ_HNbud_c243330/f135p27/1388 | i0_LQ_HNbud_c1167/f7p0/719 |
| i1_HQ_HNbud_c38732/f3p2/1347 | i2_LQ_HNbud_c59911/f1p0/2649 | i1_LQ_HNbud_c128863/f1p1/1720 | i1_HQ_HNbud_c41752/f3p11/1734 |
| i0_LQ_HNbud_c74145/f1p0/478 | i1_LQ_HNbud_c38382/f1p2/1613 | i0_LQ_HNbud_c26559/f1p13/980 | i1_HQ_HNbud_c81132/f9p2/1370 |
| i1_HQ_HNbud_c19743/f2p6/1725 | i1_LQ_HNbud_c222216/f1p2/1559 | i1_LQ_HNbud_c9789/f1p2/1603 | i3_LQ_HNbud_c4360/f1p1/3862 |
| i1_LQ_HNbud_c180922/f1p0/1448 | i1_LQ_HNbud_c51344/f3p4/1275 | i1_HQ_HNbud_c13119/f3p1/1475 | i1_LQ_HNbud_c39479/f1p0/1171 |
| i2_LQ_HNbud_c25823/f1p1/2388 | i2_HQ_HNbud_c25774/f6p2/2298 | i1_LQ_HNbud_c255449/f1p7/1051 | i0_LQ_HNbud_c71718/f1p0/885 |
| i2_HQ_HNbud_c34795/f2p0/2534 | i1_HQ_HNbud_c247868/f2p178/1742 | i1_HQ_HNbud_c5951/f12p9/1334 | i0_LQ_HNbud_c42845/f1p0/898 |
| i1_LQ_HNbud_c201891/f1p0/1979 | i2_HQ_HNbud_c65479/f9p4/2090 | i0_LQ_HNbud_c68436/f1p1/710 | i0_LQ_HNbud_c5538/f1p0/698 |
| i1_HQ_HNbud_c233865/f5p0/1083 | i2_LQ_HNbud_c55140/f1p1/2160 | i2_LQ_HNbud_c30358/f1p14/2504 | i0_LQ_HNbud_c5351/f1p9/901 |
| i1_HQ_HNbud_c46065/f132p23/1485 | i3_LQ_HNbud_c14126/f1p12/3665 | i3_LQ_HNbud_c5374/f1p0/3545 | i1_HQ_HNbud_c68955/f3p1/1259 |
| i1_HQ_HNbud_c22309/f2p19/1508 | i1_LQ_HNbud_c174926/f1p0/1570 | i1_LQ_HNbud_c60333/f1p3/1037 | i1_HQ_HNbud_c4553/f5p3/1557 |
| i0_HQ_HNbud_c97910/f3p0/565 | i0_LQ_HNbud_c20275/f1p2/526 | i1_LQ_HNbud_c148236/f1p1/1363 | i0_LQ_HNbud_c42841/f1p8/743 |
| i2_LQ_HNbud_c18320/f1p0/2262 | i2_LQ_HNbud_c4182/f2p0/2352 | i1_LQ_HNbud_c67192/f1p1/1312 | i1_LQ_HNbud_c68343/f1p3/1190 |
| i1_HQ_HNbud_c264088/f7p5/1114 | i0_LQ_HNbud_c46552/f1p0/433 | i0_LQ_HNbud_c24979/f2p11/886 | i2_LQ_HNbud_c52104/f1p7/2792 |
| i1_LQ_HNbud_c73191/f1p5/1502 | i2_LQ_HNbud_c11291/f1p7/1917 | i2_LQ_HNbud_c47151/f1p4/2062 | i1_LQ_HNbud_c199695/f1p2/1944 |
| i0_HQ_HNbud_c2833/f2p0/726 | i2_LQ_HNbud_c15843/f1p3/1981 | i1_LQ_HNbud_c8951/f1p25/1475 | i0_LQ_HNbud_c66774/f1p0/743 |
| i0_LQ_HNbud_c23816/f1p1/934 | i1_HQ_HNbud_c24323/f12p1/1823 | i0_LQ_HNbud_c22302/f1p2/608 | i1_HQ_HNbud_c20508/f6p1/1479 |
| i1_LQ_HNbud_c260206/f2p3/1833 | i1_LQ_HNbud_c221099/f1p0/1284 | i1_HQ_HNbud_c244135/f6p18/1257 | i1_LQ_HNbud_c262722/f1p38/1065 |
| i2_LQ_HNbud_c59656/f1p5/2594 | i1_HQ_HNbud_c53466/f6p7/1713 | i1_LQ_HNbud_c56135/f1p9/1125 | i1_HQ_HNbud_c627/f34p27/1706 |
| i1_LQ_HNbud_c36675/f1p2/1878 | i1_LQ_HNbud_c86311/f1p0/2010 | i2_LQ_HNbud_c20192/f1p2/2453 | i1_HQ_HNbud_c6989/f4p1/1959 |
| i1_HQ_HNbud_c14108/f6p0/1923 | i0_LQ_HNbud_c75583/f1p1/689 | i0_LQ_HNbud_c82696/f1p0/834 | i0_LQ_HNbud_c51463/f1p19/602 |
| i2_LQ_HNbud_c26470/f1p11/2333 | i1_LQ_HNbud_c203085/f1p0/2001 | i1_LQ_HNbud_c88613/f1p1/1503 | i1_HQ_HNbud_c109084/f2p1/2013 |
| i1_LQ_HNbud_c235911/f1p1/1717 | i2_LQ_HNbud_c46369/f1p2/2041 | i1_LQ_HNbud_c147972/f1p8/1710 | i1_LQ_HNbud_c133180/f1p110/1693 |
| i1_LQ_HNbud_c208852/f2p4/1020 | i2_LQ_HNbud_c56012/f1p5/2268 | i0_LQ_HNbud_c25619/f1p0/954 | i2_LQ_HNbud_c70450/f2p2/2633 |
| i4_LQ_HNbud_c2583/f1p2/4898 | i1_LQ_HNbud_c153668/f1p8/1749 | i1_HQ_HNbud_c108570/f2p0/1159 | i1_LQ_HNbud_c204690/f1p1/1367 |
| i0_LQ_HNbud_c71100/f1p9/651 | i2_LQ_HNbud_c36572/f1p14/2592 | i1_HQ_HNbud_c20527/f3p1/1411 | i0_LQ_HNbud_c45673/f1p0/655 |
| i2_LQ_HNbud_c39966/f1p23/2362 | i1_LQ_HNbud_c26578/f1p3/2056 | i0_HQ_HNbud_c66690/f2p2/778 | i2_LQ_HNbud_c27278/f1p4/3056 |
| i0_HQ_HNbud_c97533/f2p0/738 | i2_LQ_HNbud_c24651/f2p2/2491 | i0_LQ_HNbud_c10908/f1p1/572 | i1_LQ_HNbud_c213751/f1p6/1011 |
| i0_LQ_HNbud_c1464/f3p0/489 | i1_LQ_HNbud_c64420/f1p0/1224 | i0_HQ_HNbud_c7696/f7p4/831 | i2_LQ_HNbud_c51966/f1p9/2273 |
| i1_LQ_HNbud_c20126/f1p2/1603 | i4_HQ_HNbud_c231/f2p0/4130 | i1_LQ_HNbud_c141247/f1p14/1025 | i2_LQ_HNbud_c22000/f1p1/2223 |
| i3_LQ_HNbud_c7925/f1p0/3778 | i1_LQ_HNbud_c54709/f1p10/1121 | i2_HQ_HNbud_c10826/f4p18/2563 | i1_LQ_HNbud_c253354/f1p3/1319 |
| i2_LQ_HNbud_c39350/f1p3/2274 | i2_LQ_HNbud_c10007/f1p2/2225 | i1_HQ_HNbud_c111351/f2p1/1249 | i1_LQ_HNbud_c38891/f1p10/1820 |
| i1_LQ_HNbud_c197134/f1p5/1550 | i1_LQ_HNbud_c72163/f2p2/1632 | i0_LQ_HNbud_c83819/f1p0/930 | i1_LQ_HNbud_c68916/f1p3/1916 |
| i1_HQ_HNbud_c22371/f3p2/1980 | i0_LQ_HNbud_c29264/f1p0/575 | i0_HQ_HNbud_c11573/f8p1/553 | i2_HQ_HNbud_c66463/f2p2/2849 |
| i1_HQ_HNbud_c42427/f8p0/1465 | i0_HQ_HNbud_c4082/f3p7/630 | i0_LQ_HNbud_c3271/f2p12/506 | i1_HQ_HNbud_c92751/f3p0/1191 |
| i0_HQ_HNbud_c66939/f2p13/774 | i2_LQ_HNbud_c59784/f1p0/2864 | i2_HQ_HNbud_c1863/f6p4/2318 | i3_HQ_HNbud_c1496/f2p1/3743 |
| i3_LQ_HNbud_c9033/f1p6/3278 | i1_LQ_HNbud_c104012/f1p5/1814 | i0_HQ_HNbud_c97823/f10p0/574 | i1_HQ_HNbud_c8186/f2p9/1615 |
| i0_HQ_HNbud_c6739/f8p0/906 | i5_LQ_HNbud_c996/f1p0/5882 | i1_LQ_HNbud_c13544/f1p13/1221 | i0_LQ_HNbud_c53806/f1p0/834 |
| i1_LQ_HNbud_c150911/f1p3/1334 | i1_HQ_HNbud_c76203/f30p30/1059 | i1_LQ_HNbud_c278930/f1p1/1006 | i1_HQ_HNbud_c24535/f2p2/1354 |
| i0_LQ_HNbud_c54294/f1p7/639 | i1_HQ_HNbud_c4666/f2p1/1693 | i1_HQ_HNbud_c264925/f12p6/1182 | i1_LQ_HNbud_c262938/f1p0/1037 |
| i1_HQ_HNbud_c34309/f3p3/1135 | i1_HQ_HNbud_c95464/f3p4/1045 | i3_LQ_HNbud_c6622/f1p0/3159 | i0_HQ_HNbud_c98549/f88p0/723 |
| i4_LQ_HNbud_c2860/f1p1/4432 | i0_HQ_HNbud_c51856/f2p0/791 | i2_HQ_HNbud_c14108/f2p5/2394 | i1_LQ_HNbud_c227508/f1p2/1552 |
| i1_LQ_HNbud_c198916/f1p2/1245 | i1_HQ_HNbud_c264775/f87p11/1653 | i0_LQ_HNbud_c48728/f1p4/787 | i1_HQ_HNbud_c242924/f3p3/1838 |
| i1_LQ_HNbud_c112732/f1p1/1416 | i1_HQ_HNbud_c63214/f4p17/1129 | i1_LQ_HNbud_c10383/f1p2/1322 | i3_LQ_HNbud_c13621/f1p28/3657 |
| i1_LQ_HNbud_c68712/f1p0/1828 | i0_LQ_HNbud_c4843/f1p2/680 | i2_LQ_HNbud_c73473/f1p1/2039 | i2_LQ_HNbud_c30418/f1p1/2033 |
| i0_LQ_HNbud_c76244/f1p0/834 | i0_LQ_HNbud_c11391/f1p0/987 | i1_LQ_HNbud_c228346/f1p7/1761 | i2_LQ_HNbud_c23101/f1p0/2503 |
| i1_LQ_HNbud_c90649/f1p18/1560 | i1_HQ_HNbud_c172049/f2p3/1470 | i2_LQ_HNbud_c52889/f1p2/2813 | i2_LQ_HNbud_c36987/f1p16/2468 |
| i2_LQ_HNbud_c65510/f1p0/2095 | i1_HQ_HNbud_c191547/f5p0/1164 | i4_LQ_HNbud_c2253/f1p1/4439 | i1_LQ_HNbud_c8168/f1p3/1974 |
| i2_LQ_HNbud_c61919/f1p2/2036 | i2_LQ_HNbud_c45670/f1p1/2026 | i2_LQ_HNbud_c62227/f1p8/2068 | i0_HQ_HNbud_c38097/f7p2/999 |
| i2_HQ_HNbud_c910/f9p9/2531 | i2_LQ_HNbud_c31589/f9p4/2403 | i1_LQ_HNbud_c153978/f1p10/2063 | i1_HQ_HNbud_c40445/f2p2/1636 |
| i3_LQ_HNbud_c16420/f1p0/3058 | i1_LQ_HNbud_c49178/f2p3/1214 | i6_LQ_HNbud_c1015/f1p0/6328 | i2_LQ_HNbud_c4487/f1p3/2920 |
| i1_HQ_HNbud_c246624/f3p17/1662 | i1_HQ_HNbud_c41230/f5p1/1736 | i3_HQ_HNbud_c1783/f2p1/3598 | i4_LQ_HNbud_c4103/f1p2/4076 |
| i0_HQ_HNbud_c3686/f5p13/538 | i2_LQ_HNbud_c9634/f1p2/2819 | i2_HQ_HNbud_c1093/f4p0/2353 | i4_LQ_HNbud_c4168/f1p2/4069 |
| i1_LQ_HNbud_c177362/f1p0/1395 | i6_LQ_HNbud_c505/f1p0/6382 | i1_HQ_HNbud_c73720/f2p7/1703 | i1_HQ_HNbud_c143548/f18p16/1764 |
| i2_LQ_HNbud_c6965/f1p2/2589 | i1_HQ_HNbud_c234166/f4p5/1079 | i1_LQ_HNbud_c251023/f1p5/1801 | i2_LQ_HNbud_c54225/f1p4/2678 |
| i0_LQ_HNbud_c53666/f1p10/530 | i3_LQ_HNbud_c14319/f1p0/3505 | i1_HQ_HNbud_c13088/f4p3/1784 | i0_LQ_HNbud_c7732/f1p0/921 |
| i2_LQ_HNbud_c21783/f1p0/2694 | i3_LQ_HNbud_c8180/f1p0/3514 | i4_LQ_HNbud_c1561/f1p0/4630 | i0_HQ_HNbud_c97772/f15p0/748 |
| i1_LQ_HNbud_c19228/f1p11/1126 | i2_HQ_HNbud_c26025/f3p1/2931 | i1_LQ_HNbud_c153931/f1p9/1722 | i1_HQ_HNbud_c3087/f2p3/1272 |
| i1_HQ_HNbud_c1413/f22p10/1779 | i1_LQ_HNbud_c18746/f1p18/1784 | i3_HQ_HNbud_c1234/f4p3/3393 | i3_LQ_HNbud_c10797/f1p0/3318 |
| i1_HQ_HNbud_c37017/f2p3/1212 | i1_LQ_HNbud_c262855/f1p1/1048 | i1_HQ_HNbud_c48786/f2p0/1760 | i1_HQ_HNbud_c7884/f3p2/1194 |
| i4_LQ_HNbud_c3362/f1p7/4402 | i1_LQ_HNbud_c222906/f1p4/2009 | i2_LQ_HNbud_c23319/f1p0/2245 | i3_LQ_HNbud_c9264/f1p2/3126 |
| i1_LQ_HNbud_c20054/f1p2/1132 | i0_LQ_HNbud_c41401/f1p2/720 | i1_LQ_HNbud_c226920/f1p3/1923 | i3_LQ_HNbud_c15287/f1p1/3031 |
| i3_LQ_HNbud_c12556/f1p3/3374 | i1_HQ_HNbud_c1949/f12p6/1479 | i0_LQ_HNbud_c5102/f1p0/760 | i0_LQ_HNbud_c41870/f1p5/831 |
| i1_LQ_HNbud_c177045/f1p13/1703 | i2_LQ_HNbud_c28830/f1p5/2742 | i1_HQ_HNbud_c132074/f2p8/1893 | i0_HQ_HNbud_c8599/f3p0/770 |
| i0_HQ_HNbud_c60086/f2p2/453 | i1_LQ_HNbud_c279833/f1p3/1007 | i2_LQ_HNbud_c37312/f1p0/2507 | i2_LQ_HNbud_c3962/f1p24/2378 |
| i0_LQ_HNbud_c25277/f1p2/591 | i1_LQ_HNbud_c106738/f1p0/1136 | i1_LQ_HNbud_c132241/f1p0/1918 | i2_LQ_HNbud_c62913/f1p2/2020 |
| i2_LQ_HNbud_c45591/f1p6/2052 | i1_LQ_HNbud_c178036/f1p15/1897 | i0_HQ_HNbud_c30023/f2p7/940 | i2_LQ_HNbud_c16022/f1p2/2042 |
| i0_LQ_HNbud_c47247/f1p0/708 | i1_LQ_HNbud_c153891/f1p2/1650 | i1_HQ_HNbud_c255347/f2p16/1964 | i0_LQ_HNbud_c4160/f1p2/833 |
| i1_LQ_HNbud_c23564/f1p3/1697 | i1_LQ_HNbud_c279150/f1p2/1009 | i0_HQ_HNbud_c66318/f4p1/678 | i1_LQ_HNbud_c229118/f1p1/1480 |
| i2_LQ_HNbud_c43445/f1p2/2933 | i1_LQ_HNbud_c189511/f1p0/1191 | i2_LQ_HNbud_c41998/f1p18/2909 | i3_HQ_HNbud_c16812/f6p0/3360 |
| i1_LQ_HNbud_c170855/f1p0/1131 | i1_HQ_HNbud_c23968/f4p0/1401 | i1_LQ_HNbud_c19395/f1p3/1532 | i1_LQ_HNbud_c110317/f1p1/1389 |
| i1_LQ_HNbud_c50888/f1p0/1519 | i1_LQ_HNbud_c149898/f1p3/1272 | i3_LQ_HNbud_c6788/f1p0/3090 | i2_LQ_HNbud_c67537/f1p0/2213 |
| i2_LQ_HNbud_c18949/f2p7/2513 | i0_LQ_HNbud_c53846/f1p2/854 | i1_HQ_HNbud_c2163/f3p3/1735 | i2_LQ_HNbud_c52967/f1p0/2928 |
| i4_LQ_HNbud_c2276/f1p3/4361 | i1_HQ_HNbud_c265615/f14p0/1221 | i1_HQ_HNbud_c20844/f3p1/1743 | i1_LQ_HNbud_c247984/f1p10/1192 |
| i1_HQ_HNbud_c106733/f4p6/1482 | i1_LQ_HNbud_c74941/f1p0/1230 | i1_HQ_HNbud_c33132/f5p5/1821 | i1_LQ_HNbud_c152926/f1p11/1563 |
| i2_HQ_HNbud_c18272/f4p2/2844 | i2_HQ_HNbud_c70643/f25p5/2177 | i2_HQ_HNbud_c31824/f2p2/2019 | i1_HQ_HNbud_c120785/f18p0/1282 |
| i2_LQ_HNbud_c31483/f1p1/2010 | i2_LQ_HNbud_c43771/f1p3/2160 | i2_LQ_HNbud_c9337/f1p1/2395 | i2_LQ_HNbud_c28952/f1p1/2852 |
| i2_HQ_HNbud_c65300/f5p1/2087 | i2_LQ_HNbud_c20369/f1p2/2103 | i1_LQ_HNbud_c79316/f1p4/1069 | i1_LQ_HNbud_c52516/f1p4/1382 |
| i2_LQ_HNbud_c26861/f1p13/2893 | i1_LQ_HNbud_c59169/f1p11/1675 | i1_HQ_HNbud_c19118/f4p2/1525 | i1_LQ_HNbud_c50821/f1p7/1450 |
| i1_HQ_HNbud_c10414/f4p0/1484 | i1_LQ_HNbud_c52413/f1p1/1177 | i1_HQ_HNbud_c25375/f8p0/1491 | i1_LQ_HNbud_c215057/f1p0/1050 |
| i1_LQ_HNbud_c224062/f1p0/1682 | i1_HQ_HNbud_c37682/f2p3/1679 | i2_HQ_HNbud_c1897/f9p5/2458 | i1_HQ_HNbud_c143834/f7p0/1923 |
| i1_HQ_HNbud_c36418/f2p11/1133 | i2_LQ_HNbud_c35726/f1p4/2279 | i1_HQ_HNbud_c81110/f42p13/1524 | i1_HQ_HNbud_c66694/f4p1/1662 |
| i1_HQ_HNbud_c51064/f4p1/1413 | i1_LQ_HNbud_c53425/f1p1/1384 | i1_LQ_HNbud_c24301/f1p42/1559 | i1_LQ_HNbud_c103702/f1p2/1585 |
| i1_LQ_HNbud_c214620/f1p5/1011 | i1_LQ_HNbud_c8120/f3p6/1492 | i1_LQ_HNbud_c107731/f1p10/1537 | i3_HQ_HNbud_c1367/f3p0/3589 |
| i2_LQ_HNbud_c71673/f1p4/2045 | i1_LQ_HNbud_c19911/f1p7/1228 | i0_HQ_HNbud_c2167/f3p1/962 | i1_HQ_HNbud_c55346/f2p9/1824 |
| i3_LQ_HNbud_c3725/f1p0/3148 | i0_LQ_HNbud_c53099/f1p3/702 | i1_LQ_HNbud_c152353/f2p6/1293 | i0_LQ_HNbud_c53271/f1p0/867 |
| i1_HQ_HNbud_c217816/f12p1/1433 | i0_HQ_HNbud_c12235/f2p1/964 | i1_HQ_HNbud_c219938/f3p3/1392 | i2_LQ_HNbud_c60275/f1p1/2108 |
| i4_LQ_HNbud_c4334/f1p5/4010 | i1_HQ_HNbud_c48286/f3p4/1191 | i1_LQ_HNbud_c174895/f1p4/1298 | i1_LQ_HNbud_c106437/f1p2/1426 |
| i1_LQ_HNbud_c279054/f1p49/1062 | i1_LQ_HNbud_c113102/f1p0/1541 | i1_LQ_HNbud_c109694/f1p3/1174 | i1_LQ_HNbud_c26626/f1p2/1363 |
| i0_HQ_HNbud_c2369/f4p13/982 | i1_HQ_HNbud_c256295/f2p3/1058 | i2_LQ_HNbud_c22038/f1p0/2333 | i1_LQ_HNbud_c176120/f1p2/1941 |
| i0_HQ_HNbud_c97813/f5p0/974 | i2_LQ_HNbud_c51722/f1p31/2425 | i1_HQ_HNbud_c155514/f2p3/1187 | i1_LQ_HNbud_c176157/f1p6/1207 |
| i1_HQ_HNbud_c2414/f4p0/1742 | i1_LQ_HNbud_c200729/f1p0/1431 | i1_LQ_HNbud_c60133/f1p1/1065 | i1_LQ_HNbud_c41522/f5p1/1206 |
| i1_HQ_HNbud_c7912/f2p0/1624 | i2_HQ_HNbud_c70893/f4p0/2051 | i1_LQ_HNbud_c205936/f1p0/1609 | i1_LQ_HNbud_c8847/f9p4/1477 |
| i0_LQ_HNbud_c81899/f1p0/934 | i1_HQ_HNbud_c53278/f5p1/1269 | i1_LQ_HNbud_c228546/f1p1/1767 | i1_LQ_HNbud_c131282/f1p3/1265 |
| i1_LQ_HNbud_c253260/f1p1/1593 | i1_HQ_HNbud_c6556/f6p1/1429 | i1_LQ_HNbud_c86112/f1p6/1837 | i0_LQ_HNbud_c71784/f2p0/975 |
| i2_LQ_HNbud_c50757/f1p1/2646 | i0_HQ_HNbud_c99100/f3p0/853 | i2_HQ_HNbud_c14088/f2p5/2407 | i1_LQ_HNbud_c12533/f1p0/1683 |
| i2_HQ_HNbud_c9808/f5p0/2456 | i0_LQ_HNbud_c51622/f1p6/783 | i2_LQ_HNbud_c51353/f1p16/2438 | i1_LQ_HNbud_c278479/f1p1/1025 |
| i1_HQ_HNbud_c3253/f14p0/1742 | i0_HQ_HNbud_c1803/f5p12/908 | i2_LQ_HNbud_c35816/f1p2/2742 | i1_LQ_HNbud_c249386/f1p4/1861 |
| i1_HQ_HNbud_c12966/f3p0/1337 | i2_LQ_HNbud_c55073/f1p3/2920 | i0_LQ_HNbud_c23075/f1p0/823 | i0_LQ_HNbud_c5400/f1p2/868 |
| i1_LQ_HNbud_c199591/f1p5/1742 | i1_LQ_HNbud_c263296/f3p3/1064 | i1_HQ_HNbud_c8304/f7p0/1604 | i1_HQ_HNbud_c24641/f2p1/1218 |
| i1_LQ_HNbud_c130203/f1p6/1585 | i1_LQ_HNbud_c41931/f1p32/951 | i1_LQ_HNbud_c177209/f1p4/1198 | i0_HQ_HNbud_c18510/f2p12/750 |
| i0_LQ_HNbud_c22537/f1p1/830 | i1_HQ_HNbud_c133472/f2p1/2000 | i0_LQ_HNbud_c11687/f2p15/723 | i2_HQ_HNbud_c5375/f5p2/2522 |
| i1_HQ_HNbud_c46814/f27p7/1167 | i1_LQ_HNbud_c147718/f1p2/1348 | i3_LQ_HNbud_c10017/f1p8/3277 | i1_LQ_HNbud_c93261/f1p11/1739 |
| i0_HQ_HNbud_c12222/f2p0/645 | i2_HQ_HNbud_c33551/f4p3/2670 | i1_HQ_HNbud_c17498/f15p0/1128 | i0_HQ_HNbud_c54306/f2p2/875 |
| i3_LQ_HNbud_c4730/f1p0/3149 | i2_LQ_HNbud_c5309/f1p8/2554 | i1_LQ_HNbud_c197155/f1p5/1913 | i2_LQ_HNbud_c10352/f1p8/2492 |
| i2_LQ_HNbud_c55782/f1p3/2124 | i1_LQ_HNbud_c150720/f1p19/1869 | i1_LQ_HNbud_c247323/f1p7/1109 | i2_LQ_HNbud_c22464/f1p2/1896 |
| i0_LQ_HNbud_c41102/f1p196/957 | i1_LQ_HNbud_c130686/f1p27/1699 | i3_LQ_HNbud_c12074/f1p2/3250 | i2_LQ_HNbud_c55288/f1p5/2197 |
| i2_LQ_HNbud_c41040/f1p4/2125 | i1_LQ_HNbud_c93344/f1p3/1655 | i4_LQ_HNbud_c1662/f1p2/4151 | i1_HQ_HNbud_c217358/f13p18/1935 |
| i2_LQ_HNbud_c39366/f1p1/2870 | i1_HQ_HNbud_c43586/f2p2/1154 | i2_LQ_HNbud_c35617/f1p0/2294 | i0_LQ_HNbud_c73714/f1p5/386 |
| i1_HQ_HNbud_c264021/f3p1/1749 | i1_LQ_HNbud_c24083/f1p2/1621 | i0_LQ_HNbud_c10783/f1p0/999 | i4_LQ_HNbud_c1987/f1p3/4213 |
| i1_HQ_HNbud_c118088/f7p6/1466 | i1_LQ_HNbud_c124770/f1p4/1226 | i1_LQ_HNbud_c86689/f1p8/1483 | i1_HQ_HNbud_c146570/f3p2/1398 |
| i2_LQ_HNbud_c12808/f1p2/2365 | i0_HQ_HNbud_c100873/f3p1/1012 | i1_LQ_HNbud_c180558/f1p1/1786 | i1_HQ_HNbud_c6542/f4p5/1341 |
| i1_LQ_HNbud_c106828/f1p7/1770 | i1_LQ_HNbud_c98700/f1p1/1060 | i3_LQ_HNbud_c2228/f1p2/3497 | i3_LQ_HNbud_c10626/f1p5/3487 |
| i3_LQ_HNbud_c4817/f1p6/3071 | i2_HQ_HNbud_c65533/f7p4/2077 | i2_LQ_HNbud_c63705/f1p1/2047 | i2_LQ_HNbud_c38485/f1p3/2637 |
| i0_LQ_HNbud_c55216/f1p187/567 | i1_HQ_HNbud_c4583/f2p2/1295 | i1_LQ_HNbud_c70717/f1p5/1381 | i1_LQ_HNbud_c13448/f1p12/1628 |
| i1_LQ_HNbud_c177265/f1p2/1698 | i0_HQ_HNbud_c97927/f5p0/826 | i1_LQ_HNbud_c27646/f1p2/1824 | i2_LQ_HNbud_c39439/f1p1/3014 |
| i1_LQ_HNbud_c203774/f1p5/1170 | i1_LQ_HNbud_c226886/f1p108/1123 | i2_LQ_HNbud_c59494/f1p0/2170 | i1_HQ_HNbud_c265500/f4p1/1043 |
| i0_LQ_HNbud_c71544/f1p7/764 | i3_LQ_HNbud_c16568/f1p58/3022 | i1_LQ_HNbud_c201614/f1p2/1807 | i3_HQ_HNbud_c1580/f2p3/3303 |
| i0_LQ_HNbud_c23269/f1p7/666 | i2_LQ_HNbud_c24080/f1p1/2750 | i4_LQ_HNbud_c929/f1p0/4151 | i0_LQ_HNbud_c53951/f1p3/877 |
| i1_LQ_HNbud_c103988/f1p4/1162 | i2_LQ_HNbud_c10023/f1p1/2542 | i1_HQ_HNbud_c3869/f8p4/1411 | i1_LQ_HNbud_c109394/f1p9/1647 |
| i1_LQ_HNbud_c92453/f1p0/1610 | i1_LQ_HNbud_c172236/f1p0/1693 | i0_LQ_HNbud_c24050/f2p0/681 | i1_LQ_HNbud_c25719/f3p2/1597 |
| i2_LQ_HNbud_c59581/f1p0/2639 | i0_LQ_HNbud_c78503/f1p54/881 | i3_LQ_HNbud_c2873/f1p0/3140 | i1_LQ_HNbud_c125335/f1p2/1875 |
| i0_LQ_HNbud_c83941/f1p0/956 | i1_LQ_HNbud_c23212/f1p4/1674 | i1_HQ_HNbud_c207505/f4p2/1061 | i0_HQ_HNbud_c38343/f21p0/768 |
| i1_HQ_HNbud_c122296/f22p46/1073 | i1_LQ_HNbud_c203611/f1p2/1992 | i0_HQ_HNbud_c98348/f22p0/712 | i2_LQ_HNbud_c26970/f1p0/2549 |
| i2_LQ_HNbud_c56649/f1p4/2763 | i2_LQ_HNbud_c61290/f5p3/2041 | i1_LQ_HNbud_c89704/f1p13/1357 | i2_LQ_HNbud_c7224/f1p7/2795 |
| i2_LQ_HNbud_c34594/f1p5/2457 | i2_HQ_HNbud_c2163/f2p5/2847 | i2_LQ_HNbud_c51499/f1p14/2286 | i0_LQ_HNbud_c10545/f1p0/809 |
| i2_LQ_HNbud_c3242/f1p3/2258 | i2_LQ_HNbud_c59461/f1p0/2266 | i2_LQ_HNbud_c20748/f1p15/2177 | i2_LQ_HNbud_c25477/f1p5/2757 |
| i1_HQ_HNbud_c167249/f21p10/1444 | i1_LQ_HNbud_c225620/f1p3/1397 | i1_LQ_HNbud_c9645/f1p34/1581 | i3_HQ_HNbud_c2033/f2p0/3218 |
| i1_LQ_HNbud_c205662/f1p1/1281 | i0_HQ_HNbud_c98087/f3p10/695 | i1_LQ_HNbud_c140401/f1p2/1082 | i1_LQ_HNbud_c21099/f1p7/1211 |
| i1_LQ_HNbud_c5578/f1p4/1951 | i0_HQ_HNbud_c18298/f5p8/866 | i1_LQ_HNbud_c73675/f1p1/1211 | i1_LQ_HNbud_c104939/f1p3/1679 |
| i0_HQ_HNbud_c2161/f3p3/656 | i2_LQ_HNbud_c55658/f1p4/2174 | i1_LQ_HNbud_c250713/f1p4/1264 | i2_HQ_HNbud_c9117/f3p20/2966 |
| i1_LQ_HNbud_c105465/f1p3/1544 | i1_LQ_HNbud_c150701/f1p8/1324 | i0_LQ_HNbud_c63653/f1p2/798 | i0_HQ_HNbud_c99580/f2p0/901 |
| i2_HQ_HNbud_c22087/f2p2/2087 | i2_LQ_HNbud_c12409/f1p0/2555 | i1_HQ_HNbud_c243860/f3p1/1741 | i0_LQ_HNbud_c32025/f1p3/644 |
| i1_LQ_HNbud_c133141/f1p23/1803 | i1_HQ_HNbud_c101283/f18p10/1508 | i1_LQ_HNbud_c18241/f1p4/1378 | i1_LQ_HNbud_c85573/f1p2/1155 |
| i0_LQ_HNbud_c26109/f1p0/691 | i0_LQ_HNbud_c41578/f1p1/615 | i1_HQ_HNbud_c178726/f2p0/1519 | i1_LQ_HNbud_c224717/f1p1/1286 |
| i1_LQ_HNbud_c4643/f1p7/1820 | i1_LQ_HNbud_c154581/f1p5/1467 | i0_LQ_HNbud_c26307/f3p25/999 | i0_HQ_HNbud_c97766/f24p0/600 |
| i1_LQ_HNbud_c86793/f1p0/1720 | i2_LQ_HNbud_c60428/f1p13/2290 | i2_LQ_HNbud_c40750/f1p2/2527 | i1_LQ_HNbud_c199185/f1p16/1884 |
| i2_LQ_HNbud_c37386/f1p2/2491 | i3_LQ_HNbud_c10505/f1p4/3092 | i2_LQ_HNbud_c14611/f1p0/3012 | i1_HQ_HNbud_c92296/f3p4/1747 |
| i2_LQ_HNbud_c57067/f1p0/2978 | i2_LQ_HNbud_c35879/f1p2/2870 | i1_LQ_HNbud_c23710/f1p31/1426 | i1_HQ_HNbud_c217409/f21p0/1514 |
| i2_LQ_HNbud_c6078/f2p0/2384 | i2_LQ_HNbud_c7348/f1p27/2118 | i2_LQ_HNbud_c46529/f1p0/2047 | i0_LQ_HNbud_c5495/f1p11/986 |
| i4_LQ_HNbud_c3889/f1p7/4169 | i2_LQ_HNbud_c58163/f1p1/2408 | i1_LQ_HNbud_c9872/f1p5/1414 | i1_LQ_HNbud_c27312/f1p9/1707 |
| i1_LQ_HNbud_c108368/f1p4/1609 | i1_HQ_HNbud_c171373/f2p0/1414 | i1_LQ_HNbud_c201551/f1p0/1370 | i2_LQ_HNbud_c28730/f1p0/2834 |
| i0_HQ_HNbud_c2172/f11p5/851 | i2_LQ_HNbud_c67030/f1p10/2333 | i2_LQ_HNbud_c52277/f1p1/2156 | i1_HQ_HNbud_c1881/f42p27/1838 |
| i2_LQ_HNbud_c4713/f1p2/2061 | i2_LQ_HNbud_c50130/f1p1/2467 | i1_HQ_HNbud_c90307/f2p0/1746 | i0_LQ_HNbud_c72808/f1p2/980 |
| i1_HQ_HNbud_c15061/f2p0/1105 | i2_LQ_HNbud_c62348/f1p0/2014 | i2_HQ_HNbud_c23078/f2p3/2594 | i2_LQ_HNbud_c57956/f1p3/2753 |
| i1_LQ_HNbud_c228821/f1p5/1779 | i0_HQ_HNbud_c1614/f6p21/653 | i2_LQ_HNbud_c51310/f1p2/2404 | i3_LQ_HNbud_c3079/f1p3/3665 |
| i1_LQ_HNbud_c199291/f1p5/1479 | i1_LQ_HNbud_c91209/f1p0/1513 | i3_LQ_HNbud_c7190/f1p1/3770 | i1_LQ_HNbud_c130800/f1p2/1497 |
| i1_LQ_HNbud_c30327/f1p0/1037 | i1_HQ_HNbud_c21604/f3p5/1358 | i1_LQ_HNbud_c106623/f1p128/1791 | i6_LQ_HNbud_c1746/f1p0/6759 |
| i1_HQ_HNbud_c95729/f2p0/1087 | i1_HQ_HNbud_c34418/f2p20/1737 | i4_LQ_HNbud_c1517/f1p1/4071 | i1_LQ_HNbud_c196546/f1p0/1285 |
| i0_LQ_HNbud_c13749/f1p6/790 | i1_HQ_HNbud_c75710/f2p3/1637 | i1_LQ_HNbud_c87346/f1p24/1534 | i1_LQ_HNbud_c67867/f1p14/1210 |
| i1_LQ_HNbud_c89030/f1p7/1662 | i1_LQ_HNbud_c19361/f1p0/1982 | i1_HQ_HNbud_c12090/f7p4/1446 | i1_LQ_HNbud_c89006/f1p9/1376 |
| i0_HQ_HNbud_c98906/f10p1/901 | i1_LQ_HNbud_c109163/f1p1/1296 | i1_HQ_HNbud_c187229/f2p0/1484 | i3_HQ_HNbud_c1331/f3p0/4006 |
| i2_HQ_HNbud_c34080/f2p0/2201 | i1_LQ_HNbud_c15361/f1p0/1057 | i0_LQ_HNbud_c42825/f1p0/464 | i2_LQ_HNbud_c40339/f1p0/2545 |
| i1_HQ_HNbud_c17736/f9p5/1686 | i0_LQ_HNbud_c6962/f1p0/831 | i1_HQ_HNbud_c20960/f5p2/1492 | i1_HQ_HNbud_c25108/f3p1/1619 |
| i0_HQ_HNbud_c25767/f3p1/807 | i0_LQ_HNbud_c73479/f1p0/880 | i1_LQ_HNbud_c40048/f1p5/1586 | i1_LQ_HNbud_c79911/f1p4/1094 |
| i1_HQ_HNbud_c167035/f11p3/1270 | i1_HQ_HNbud_c217767/f12p3/1278 | i1_LQ_HNbud_c108999/f1p0/1349 | i2_LQ_HNbud_c10619/f1p3/2155 |
| i1_HQ_HNbud_c131132/f2p0/1553 | i0_LQ_HNbud_c26528/f1p4/676 | i1_HQ_HNbud_c6311/f3p9/1306 | i2_HQ_HNbud_c47845/f7p4/2320 |
| i2_LQ_HNbud_c26511/f1p2/2484 | i1_HQ_HNbud_c50510/f5p1/1637 | i0_HQ_HNbud_c20621/f3p0/837 | i3_LQ_HNbud_c11376/f1p2/3687 |
| i4_LQ_HNbud_c2591/f1p2/4162 | i0_LQ_HNbud_c52088/f1p4/759 | i0_HQ_HNbud_c627/f16p19/714 | i0_LQ_HNbud_c80544/f1p0/622 |
| i2_LQ_HNbud_c8642/f1p2/2955 | i1_HQ_HNbud_c93235/f4p1/1802 | i2_HQ_HNbud_c27866/f2p1/3004 | i0_HQ_HNbud_c11968/f3p2/958 |
| i2_LQ_HNbud_c62944/f1p2/2044 | i0_LQ_HNbud_c29125/f1p1/713 | i5_LQ_HNbud_c1066/f1p0/5325 | i7_LQ_HNbud_c1160/f1p0/7046 |
| i7_LQ_HNbud_c642/f1p0/7583 | i1_LQ_HNbud_c55994/f1p1/1598 | i2_LQ_HNbud_c21594/f1p0/2385 | i1_LQ_HNbud_c84648/f1p8/1850 |
| i1_HQ_HNbud_c150873/f3p5/1864 | i1_LQ_HNbud_c171291/f1p8/1173 | i2_HQ_HNbud_c28684/f2p5/2414 | i1_HQ_HNbud_c41490/f5p0/1369 |
| i1_HQ_HNbud_c38319/f7p6/1866 | i0_HQ_HNbud_c2259/f3p6/952 | i1_LQ_HNbud_c86227/f1p0/1185 | i0_HQ_HNbud_c98397/f53p0/755 |
| i1_HQ_HNbud_c183893/f2p1/1086 | i1_LQ_HNbud_c176460/f1p1/1800 | i1_LQ_HNbud_c176105/f1p0/1098 | i1_LQ_HNbud_c92004/f1p4/1868 |
| i0_LQ_HNbud_c68772/f1p0/953 | i0_HQ_HNbud_c2292/f5p0/1000 | i3_HQ_HNbud_c1316/f3p1/3956 | i3_LQ_HNbud_c5947/f1p0/3540 |
| i1_LQ_HNbud_c49414/f1p0/1668 | i2_LQ_HNbud_c44139/f1p2/2134 | i1_LQ_HNbud_c164766/f1p0/1083 | i0_HQ_HNbud_c66533/f3p0/778 |
| i0_LQ_HNbud_c11612/f1p8/798 | i0_LQ_HNbud_c4141/f5p1/683 | i1_HQ_HNbud_c106606/f2p9/1560 | i1_LQ_HNbud_c29035/f1p5/1066 |
| i0_LQ_HNbud_c45903/f1p0/905 | i1_LQ_HNbud_c201532/f1p2/1568 | i0_LQ_HNbud_c51595/f1p1/550 | i3_LQ_HNbud_c1578/f2p3/3256 |
| i1_LQ_HNbud_c205128/f2p8/1191 | i0_LQ_HNbud_c43057/f1p0/760 | i1_LQ_HNbud_c106869/f1p16/1539 | i1_LQ_HNbud_c225541/f1p3/1791 |
| i2_LQ_HNbud_c34718/f1p2/2507 | i3_LQ_HNbud_c12804/f1p4/3078 | i0_LQ_HNbud_c84332/f1p0/955 | i2_LQ_HNbud_c46877/f1p1/2250 |
| i1_LQ_HNbud_c20256/f1p8/1698 | i1_LQ_HNbud_c51499/f1p23/1486 | i3_LQ_HNbud_c2011/f2p0/3337 | i0_LQ_HNbud_c25244/f1p1/1001 |
| i1_LQ_HNbud_c50419/f1p5/1419 | i0_LQ_HNbud_c9102/f1p1/541 | i2_LQ_HNbud_c41721/f1p0/2200 | i0_HQ_HNbud_c65170/f10p3/779 |
| i1_HQ_HNbud_c95549/f2p2/1021 | i6_LQ_HNbud_c1134/f1p0/6723 | i1_LQ_HNbud_c209001/f2p2/1018 | i4_LQ_HNbud_c3322/f1p0/4959 |
| i1_LQ_HNbud_c138479/f1p27/941 | i2_LQ_HNbud_c28673/f1p0/2694 | i1_LQ_HNbud_c253785/f1p9/1429 | i1_HQ_HNbud_c34492/f3p2/1485 |
| i0_HQ_HNbud_c98183/f33p0/953 | i1_HQ_HNbud_c917/f57p11/1654 | i1_HQ_HNbud_c154058/f2p5/1882 | i1_HQ_HNbud_c108059/f2p5/1349 |
| i2_HQ_HNbud_c38483/f2p2/2254 | i1_LQ_HNbud_c42105/f1p3/1494 | i1_LQ_HNbud_c200170/f1p5/1470 | i1_LQ_HNbud_c50271/f2p18/1312 |
| i1_LQ_HNbud_c120619/f1p0/1083 | i0_LQ_HNbud_c82263/f1p0/842 | i1_HQ_HNbud_c167288/f3p3/1161 | i1_HQ_HNbud_c25819/f5p1/1782 |
| i2_LQ_HNbud_c26355/f1p14/2890 | i1_HQ_HNbud_c166146/f22p1/1299 | i2_HQ_HNbud_c70433/f12p0/2303 | i3_LQ_HNbud_c18012/f1p2/3132 |
| i0_LQ_HNbud_c27694/f1p0/986 | i1_LQ_HNbud_c39858/f1p2/1408 | i1_HQ_HNbud_c216494/f11p1/1583 | i1_HQ_HNbud_c265793/f13p8/1770 |
| i1_HQ_HNbud_c108215/f2p1/1180 | i1_LQ_HNbud_c22907/f1p1/1237 | i3_HQ_HNbud_c1583/f2p1/3738 | i0_HQ_HNbud_c6798/f3p2/890 |
| i1_LQ_HNbud_c109642/f1p4/1363 | i0_HQ_HNbud_c7868/f3p0/944 | i3_LQ_HNbud_c12652/f1p2/3254 | i1_LQ_HNbud_c229974/f1p1/1794 |
| i1_HQ_HNbud_c26397/f5p1/1450 | i4_HQ_HNbud_c225/f2p0/4291 | i1_HQ_HNbud_c265086/f7p4/1367 | i1_LQ_HNbud_c174010/f1p11/1691 |
| i1_HQ_HNbud_c33671/f2p17/1670 | i1_LQ_HNbud_c172939/f1p4/1483 | i1_HQ_HNbud_c198894/f2p6/1337 | i1_HQ_HNbud_c17668/f3p4/1606 |
| i0_LQ_HNbud_c46358/f1p0/442 | i1_LQ_HNbud_c49769/f1p11/1306 | i1_LQ_HNbud_c130756/f1p0/1550 | i2_LQ_HNbud_c63908/f1p5/2021 |
| i2_LQ_HNbud_c53363/f1p2/2778 | i1_HQ_HNbud_c73796/f3p5/1545 | i2_LQ_HNbud_c72626/f1p11/2775 | i0_LQ_HNbud_c49766/f1p2/843 |
| i1_LQ_HNbud_c68951/f1p1/1678 | i0_LQ_HNbud_c28321/f2p0/515 | i2_HQ_HNbud_c1628/f2p3/2554 | i5_LQ_HNbud_c178/f1p0/5348 |
| i1_HQ_HNbud_c120840/f24p10/1494 | i2_LQ_HNbud_c27948/f1p11/2579 | i1_HQ_HNbud_c167275/f22p3/1121 | i1_LQ_HNbud_c223243/f1p0/1583 |
| i2_HQ_HNbud_c10837/f2p1/2640 | i2_LQ_HNbud_c20049/f1p0/2155 | i1_LQ_HNbud_c223091/f1p1/1324 | i1_HQ_HNbud_c66853/f2p4/1405 |
| i0_LQ_HNbud_c22176/f1p2/441 | i1_LQ_HNbud_c12938/f1p5/2093 | i2_LQ_HNbud_c69442/f1p17/2124 | i1_LQ_HNbud_c22059/f1p2/1419 |
| i1_LQ_HNbud_c44363/f1p0/1031 | i0_LQ_HNbud_c1965/f2p0/741 | i3_LQ_HNbud_c4159/f1p3/3452 | i2_LQ_HNbud_c26288/f1p11/2389 |
| i0_LQ_HNbud_c61030/f1p0/715 | i1_HQ_HNbud_c92062/f2p0/1722 | i1_HQ_HNbud_c264398/f11p2/1580 | i1_LQ_HNbud_c35244/f1p0/1842 |
| i1_LQ_HNbud_c55083/f1p3/1833 | i1_LQ_HNbud_c15644/f1p0/1108 | i1_HQ_HNbud_c257246/f2p3/1091 | i2_LQ_HNbud_c51155/f1p1/2974 |
| i1_LQ_HNbud_c67194/f1p192/1477 | i1_HQ_HNbud_c124066/f7p3/1837 | i1_HQ_HNbud_c68153/f2p1/1664 | i1_HQ_HNbud_c12088/f8p3/1267 |
| i1_LQ_HNbud_c279206/f1p0/1005 | i2_LQ_HNbud_c29092/f1p0/2881 | i1_LQ_HNbud_c155048/f1p8/1317 | i2_LQ_HNbud_c56107/f1p1/2533 |
| i1_LQ_HNbud_c108014/f1p35/1412 | i2_LQ_HNbud_c20225/f1p2/2636 | i1_HQ_HNbud_c23069/f4p2/1675 | i3_LQ_HNbud_c16071/f1p7/3095 |
| i2_LQ_HNbud_c42111/f1p17/2948 | i0_LQ_HNbud_c85867/f1p0/644 | i2_HQ_HNbud_c1282/f5p3/2210 | i1_LQ_HNbud_c226086/f1p17/1230 |
| i2_LQ_HNbud_c22093/f1p0/2722 | i1_LQ_HNbud_c48943/f1p1/1470 | i2_LQ_HNbud_c56683/f1p26/2825 | i1_LQ_HNbud_c226688/f1p2/1846 |
| i2_HQ_HNbud_c655/f22p1/2588 | i1_LQ_HNbud_c4325/f1p2/1891 | i1_HQ_HNbud_c263242/f20p0/1265 | i1_LQ_HNbud_c172321/f1p3/1383 |
| i1_LQ_HNbud_c251113/f1p1/1281 | i1_LQ_HNbud_c105754/f1p0/1931 | i1_LQ_HNbud_c18109/f1p82/1731 | i1_LQ_HNbud_c23064/f1p15/1355 |
| i2_LQ_HNbud_c50605/f1p3/2598 | i0_LQ_HNbud_c2208/f2p6/595 | i1_HQ_HNbud_c86339/f2p6/1605 | i1_HQ_HNbud_c263579/f13p2/1176 |
| i0_LQ_HNbud_c72618/f1p0/888 | i2_LQ_HNbud_c12642/f1p5/2171 | i1_HQ_HNbud_c1076/f58p13/1330 | i1_LQ_HNbud_c105735/f1p0/1459 |
| i1_HQ_HNbud_c24455/f10p3/1617 | i1_LQ_HNbud_c41024/f1p1/1260 | i0_HQ_HNbud_c66786/f2p7/561 | i2_LQ_HNbud_c24843/f1p2/2202 |
| i0_HQ_HNbud_c98374/f2p0/687 | i1_LQ_HNbud_c97610/f1p0/1059 | i1_HQ_HNbud_c24218/f8p3/1522 | i1_HQ_HNbud_c21980/f6p5/1843 |
| i0_LQ_HNbud_c19765/f1p3/913 | i1_LQ_HNbud_c57632/f1p7/1427 | i1_LQ_HNbud_c180417/f1p0/1846 | i0_HQ_HNbud_c98523/f59p0/498 |
| i1_HQ_HNbud_c13103/f3p6/1334 | i2_LQ_HNbud_c23443/f1p4/2504 | i1_LQ_HNbud_c225833/f1p2/1899 | i2_LQ_HNbud_c24011/f1p7/2678 |
| i3_LQ_HNbud_c6619/f1p0/3769 | i0_LQ_HNbud_c6822/f1p0/585 | i0_LQ_HNbud_c5584/f1p5/793 | i2_LQ_HNbud_c52843/f1p3/2456 |
| i2_LQ_HNbud_c54902/f1p1/2505 | i1_LQ_HNbud_c18600/f1p0/1295 | i1_HQ_HNbud_c255876/f3p0/1079 | i1_LQ_HNbud_c95658/f1p7/1213 |
| i1_LQ_HNbud_c104627/f1p1/1894 | i1_LQ_HNbud_c7545/f1p1/1843 | i0_LQ_HNbud_c7813/f2p5/824 | i5_LQ_HNbud_c330/f1p0/5124 |
| i1_HQ_HNbud_c192092/f20p6/1901 | i1_HQ_HNbud_c13740/f5p8/1397 | i2_LQ_HNbud_c33503/f2p5/2534 | i1_LQ_HNbud_c13931/f1p0/1198 |
| i1_LQ_HNbud_c225398/f1p3/1418 | i1_LQ_HNbud_c35868/f1p0/1129 | i1_LQ_HNbud_c91667/f1p15/1789 | i1_LQ_HNbud_c220917/f2p1/1579 |
| i1_LQ_HNbud_c19796/f1p0/1311 | i3_HQ_HNbud_c983/f5p0/3767 | i1_LQ_HNbud_c148968/f1p1/1982 | i1_HQ_HNbud_c104416/f3p3/1124 |
| i2_HQ_HNbud_c1854/f8p2/2446 | i1_LQ_HNbud_c252994/f1p4/1209 | i1_LQ_HNbud_c202106/f1p22/1286 | i1_LQ_HNbud_c29237/f1p29/1049 |
| i1_LQ_HNbud_c93137/f1p4/1692 | i1_LQ_HNbud_c42350/f1p9/1737 | i3_LQ_HNbud_c6611/f1p7/3940 | i1_LQ_HNbud_c130609/f1p1/1728 |
| i2_LQ_HNbud_c43501/f1p5/2570 | i0_HQ_HNbud_c31050/f2p0/843 | i0_LQ_HNbud_c9881/f1p2/734 | i1_LQ_HNbud_c242914/f3p16/1227 |
| i2_HQ_HNbud_c48243/f6p3/2088 | i3_HQ_HNbud_c1549/f2p5/3922 | i1_HQ_HNbud_c243587/f7p5/1561 | i0_HQ_HNbud_c39705/f3p0/829 |
| i1_HQ_HNbud_c102857/f17p17/1382 | i1_LQ_HNbud_c68803/f1p1/1797 | i0_LQ_HNbud_c23645/f1p8/444 | i2_LQ_HNbud_c42724/f1p0/2340 |
| i2_LQ_HNbud_c54426/f1p3/2211 | i1_HQ_HNbud_c263499/f3p1/1167 | i1_HQ_HNbud_c263195/f3p4/1314 | i1_HQ_HNbud_c101439/f27p7/1791 |
| i1_LQ_HNbud_c2913/f1p3/1554 | i0_LQ_HNbud_c98961/f1p4/965 | i0_HQ_HNbud_c98139/f4p0/798 | i1_LQ_HNbud_c19043/f1p3/1530 |
| i1_LQ_HNbud_c224389/f1p1/1281 | i1_LQ_HNbud_c153645/f1p7/1811 | i1_LQ_HNbud_c277482/f1p0/1006 | i1_LQ_HNbud_c29119/f1p2/1010 |
| i1_LQ_HNbud_c249617/f1p6/1696 | i1_LQ_HNbud_c56020/f1p5/1715 | i2_LQ_HNbud_c15421/f1p5/2044 | i1_LQ_HNbud_c34204/f1p6/1381 |
| i0_HQ_HNbud_c98105/f17p0/699 | i2_LQ_HNbud_c63203/f1p2/2036 | i3_LQ_HNbud_c1665/f2p3/3523 | i1_LQ_HNbud_c280062/f1p10/1007 |
| i0_LQ_HNbud_c51932/f1p3/854 | i2_LQ_HNbud_c31295/f1p3/2011 | i1_LQ_HNbud_c41005/f1p0/1730 | i2_HQ_HNbud_c68456/f2p0/2030 |
| i2_LQ_HNbud_c37689/f1p11/2237 | i0_LQ_HNbud_c26104/f1p0/917 | i2_LQ_HNbud_c3440/f1p3/2920 | i2_LQ_HNbud_c34711/f1p4/2991 |
| i2_LQ_HNbud_c39400/f1p4/2254 | i1_LQ_HNbud_c151642/f1p5/1371 | i3_LQ_HNbud_c3586/f1p2/3267 | i1_LQ_HNbud_c108180/f1p2/1337 |
| i1_HQ_HNbud_c263715/f4p1/1280 | i5_LQ_HNbud_c793/f1p0/5361 | i0_LQ_HNbud_c22063/f1p0/822 | i2_LQ_HNbud_c9413/f1p0/2981 |
| i1_HQ_HNbud_c168502/f9p5/1052 | i0_LQ_HNbud_c73542/f1p0/942 | i1_LQ_HNbud_c4563/f1p5/1361 | i1_LQ_HNbud_c20748/f3p22/1664 |
| i0_LQ_HNbud_c77910/f1p0/662 | i3_LQ_HNbud_c7195/f1p1/3619 | i1_LQ_HNbud_c170547/f1p5/1372 | i2_LQ_HNbud_c21569/f1p14/2384 |
| i1_LQ_HNbud_c189374/f1p2/1014 | i1_LQ_HNbud_c60143/f1p1/1045 | i0_HQ_HNbud_c2397/f11p1/733 | i3_LQ_HNbud_c10882/f1p47/3224 |
| i1_LQ_HNbud_c223883/f1p2/1177 | i3_LQ_HNbud_c16002/f1p7/3044 | i2_LQ_HNbud_c34596/f1p7/2315 | i3_LQ_HNbud_c3744/f1p0/3388 |
| i1_LQ_HNbud_c154617/f1p2/1635 | i1_HQ_HNbud_c263236/f8p10/1004 | i0_LQ_HNbud_c77034/f1p0/677 | i1_LQ_HNbud_c194843/f1p8/1275 |
| i1_HQ_HNbud_c125255/f2p1/1586 | i2_HQ_HNbud_c64519/f36p1/2780 | i1_HQ_HNbud_c130362/f2p3/1546 | i2_LQ_HNbud_c24874/f1p7/2854 |
| i1_HQ_HNbud_c66117/f2p3/1852 | i2_LQ_HNbud_c41573/f1p0/2271 | i2_LQ_HNbud_c38496/f1p3/2944 | i0_HQ_HNbud_c37953/f16p3/529 |
| i1_LQ_HNbud_c57443/f1p1/1296 | i1_HQ_HNbud_c23436/f4p0/1285 | i2_LQ_HNbud_c19158/f1p3/2688 | i2_LQ_HNbud_c25586/f1p1/2412 |
| i1_LQ_HNbud_c153529/f1p132/1486 | i3_HQ_HNbud_c1723/f2p0/3402 | i0_LQ_HNbud_c4614/f1p7/995 | i1_LQ_HNbud_c99169/f1p3/1036 |
| i2_LQ_HNbud_c39096/f1p21/2894 | i4_LQ_HNbud_c2713/f1p0/4847 | i1_LQ_HNbud_c205707/f1p0/1860 | i4_LQ_HNbud_c4053/f1p4/4099 |
| i2_HQ_HNbud_c68046/f2p18/2357 | i1_HQ_HNbud_c52193/f2p2/1148 | i1_HQ_HNbud_c4704/f7p2/1455 | i1_LQ_HNbud_c74253/f1p0/1695 |
| i3_LQ_HNbud_c9862/f1p0/3428 | i0_LQ_HNbud_c6580/f2p15/860 | i0_HQ_HNbud_c11274/f2p2/515 | i1_HQ_HNbud_c143589/f16p5/1375 |
| i1_LQ_HNbud_c38139/f1p0/1796 | i2_LQ_HNbud_c20608/f1p0/2297 | i1_HQ_HNbud_c101130/f34p10/1509 | i0_LQ_HNbud_c43938/f1p1/772 |
| i1_LQ_HNbud_c57199/f1p1/1740 | i2_HQ_HNbud_c1664/f4p8/2300 | i1_HQ_HNbud_c6033/f3p2/1115 | i1_LQ_HNbud_c109055/f1p0/1768 |
| i1_HQ_HNbud_c71823/f2p26/2535 | i2_HQ_HNbud_c21619/f2p4/2780 | i1_LQ_HNbud_c54679/f1p10/1681 | i2_LQ_HNbud_c53430/f1p95/2327 |
| i2_HQ_HNbud_c27619/f2p0/2453 | i1_LQ_HNbud_c131565/f1p2/1399 | i1_LQ_HNbud_c20774/f1p3/1209 | i2_LQ_HNbud_c55828/f1p96/2727 |
| i0_LQ_HNbud_c79674/f1p0/786 | i0_LQ_HNbud_c98856/f52p0/967 | i1_HQ_HNbud_c37999/f2p4/1841 | i2_LQ_HNbud_c47220/f1p1/2558 |
| i1_LQ_HNbud_c134006/f1p3/1628 | i1_LQ_HNbud_c3945/f1p0/1520 | i2_HQ_HNbud_c70478/f20p1/2063 | i3_LQ_HNbud_c2688/f1p8/3173 |
| i1_LQ_HNbud_c155762/f1p1/1370 | i1_LQ_HNbud_c108338/f1p1/1263 | i1_LQ_HNbud_c10259/f1p0/1559 | i2_LQ_HNbud_c26140/f1p3/2969 |
| i1_LQ_HNbud_c74750/f1p0/1917 | i1_LQ_HNbud_c40496/f2p2/1313 | i2_HQ_HNbud_c19772/f3p0/2817 | i1_HQ_HNbud_c100666/f23p17/1635 |
| i0_LQ_HNbud_c8205/f2p3/829 | i1_LQ_HNbud_c12202/f1p3/1665 | i2_HQ_HNbud_c25235/f3p7/2926 | i2_HQ_HNbud_c25161/f4p0/2391 |
| i0_HQ_HNbud_c18420/f3p0/551 | i1_HQ_HNbud_c40211/f3p1/1958 | i0_HQ_HNbud_c1206/f7p0/788 | i2_LQ_HNbud_c41575/f1p3/2107 |
| i1_HQ_HNbud_c120722/f27p13/1594 | i1_LQ_HNbud_c69482/f1p7/1906 | i2_LQ_HNbud_c41771/f1p25/2699 | i3_LQ_HNbud_c3477/f1p3/3921 |
| i1_HQ_HNbud_c267494/f2p1/1054 | i2_HQ_HNbud_c61668/f2p2/2036 | i1_LQ_HNbud_c174342/f1p9/1948 | i1_HQ_HNbud_c216332/f13p4/1648 |
| i1_HQ_HNbud_c234650/f3p0/1066 | i0_HQ_HNbud_c38085/f7p17/954 | i1_HQ_HNbud_c23676/f2p2/1645 | i2_LQ_HNbud_c15887/f1p10/2046 |
| i2_HQ_HNbud_c70644/f10p1/2162 | i2_LQ_HNbud_c26868/f1p0/2303 | i1_LQ_HNbud_c229491/f1p20/1663 | i1_HQ_HNbud_c166142/f9p3/1540 |
| i1_HQ_HNbud_c66651/f5p7/1994 | i1_LQ_HNbud_c156063/f1p3/1541 | i1_HQ_HNbud_c13736/f11p8/1730 | i1_LQ_HNbud_c278399/f1p11/967 |
| i1_LQ_HNbud_c115897/f1p1/1051 | i1_HQ_HNbud_c265038/f14p5/1410 | i3_LQ_HNbud_c8223/f1p3/3488 | i1_LQ_HNbud_c56908/f1p5/1347 |
| i2_HQ_HNbud_c27390/f2p1/2678 | i1_LQ_HNbud_c19038/f1p23/1629 | i1_LQ_HNbud_c73746/f1p1/1371 | i1_LQ_HNbud_c74418/f1p5/1520 |
| i1_HQ_HNbud_c55879/f2p0/1122 | i1_LQ_HNbud_c17442/f6p1/1588 | i2_LQ_HNbud_c52532/f1p21/2625 | i1_LQ_HNbud_c104244/f1p3/1896 |
| i0_LQ_HNbud_c77508/f1p0/690 | i0_LQ_HNbud_c29432/f1p0/808 | i1_LQ_HNbud_c112826/f4p4/1562 | i2_HQ_HNbud_c20019/f3p0/2365 |
| i0_LQ_HNbud_c25559/f1p1/983 | i1_LQ_HNbud_c11260/f1p11/1750 | i0_HQ_HNbud_c43811/f2p0/727 | i2_LQ_HNbud_c5557/f1p1/2638 |
| i4_LQ_HNbud_c4297/f1p6/4045 | i1_HQ_HNbud_c55529/f6p0/1642 | i1_LQ_HNbud_c273338/f1p3/1634 | i1_LQ_HNbud_c108801/f1p11/1559 |
| i1_HQ_HNbud_c7543/f2p9/1415 | i1_LQ_HNbud_c12790/f1p22/1485 | i0_HQ_HNbud_c13139/f4p1/734 | i4_LQ_HNbud_c437/f1p0/4276 |
| i0_HQ_HNbud_c30197/f3p3/894 | i1_LQ_HNbud_c23342/f1p20/1685 | i1_HQ_HNbud_c125942/f4p3/1536 | i1_LQ_HNbud_c228495/f1p7/1178 |
| i1_LQ_HNbud_c88162/f1p7/1287 | i0_LQ_HNbud_c4317/f1p0/713 | i2_HQ_HNbud_c11841/f5p1/2432 | i2_LQ_HNbud_c40297/f1p0/2450 |
| i1_HQ_HNbud_c264781/f11p8/1400 | i1_LQ_HNbud_c103995/f1p5/1567 | i1_LQ_HNbud_c199412/f1p2/1284 | i0_HQ_HNbud_c11636/f2p0/685 |
| i2_LQ_HNbud_c20721/f1p1/2588 | i0_LQ_HNbud_c46393/f1p12/690 | i2_LQ_HNbud_c60407/f1p106/2275 | i1_LQ_HNbud_c73300/f1p25/1399 |
| i1_LQ_HNbud_c162181/f1p2/1077 | i3_LQ_HNbud_c12600/f1p12/3485 | i1_LQ_HNbud_c106170/f1p8/1400 | i3_LQ_HNbud_c12891/f1p2/3744 |
| i3_LQ_HNbud_c11651/f1p3/3092 | i1_HQ_HNbud_c135508/f6p0/1074 | i1_HQ_HNbud_c219660/f142p28/1806 | i1_LQ_HNbud_c29656/f1p15/1062 |
| i1_HQ_HNbud_c54371/f4p0/1325 | i0_LQ_HNbud_c73435/f1p3/723 | i1_LQ_HNbud_c175736/f1p3/1169 | i1_HQ_HNbud_c144622/f5p0/1236 |
| i1_LQ_HNbud_c10986/f5p3/1636 | i1_LQ_HNbud_c33559/f1p8/1396 | i1_LQ_HNbud_c34262/f1p6/1348 | i0_LQ_HNbud_c48143/f1p0/759 |
| i0_HQ_HNbud_c2410/f4p0/898 | i0_LQ_HNbud_c75597/f1p17/845 | i1_LQ_HNbud_c205950/f1p10/1811 | i0_LQ_HNbud_c28351/f1p3/921 |
| i3_LQ_HNbud_c2745/f1p4/3855 | i2_LQ_HNbud_c22060/f1p1/2222 | i4_LQ_HNbud_c3586/f1p2/4407 | i0_LQ_HNbud_c43779/f1p2/950 |
| i6_LQ_HNbud_c2556/f1p0/6495 | i2_HQ_HNbud_c65384/f27p5/2479 | i1_HQ_HNbud_c13827/f5p0/1344 | i1_LQ_HNbud_c107494/f1p4/1861 |
| i0_LQ_HNbud_c42906/f1p0/748 | i1_LQ_HNbud_c13045/f2p2/1659 | i2_HQ_HNbud_c31686/f8p1/2605 | i1_HQ_HNbud_c65728/f3p1/1478 |
| i2_LQ_HNbud_c3395/f1p2/2975 | i1_LQ_HNbud_c202307/f1p5/1846 | i3_HQ_HNbud_c15236/f2p2/3063 | i2_LQ_HNbud_c30718/f1p5/2080 |
| i1_LQ_HNbud_c71451/f4p4/1500 | i0_HQ_HNbud_c1157/f6p1/841 | i1_LQ_HNbud_c1489/f17p11/1918 | i2_LQ_HNbud_c65147/f1p3/2231 |
| i1_LQ_HNbud_c112785/f1p1/1878 | i1_LQ_HNbud_c178376/f1p0/1144 | i1_LQ_HNbud_c147740/f1p0/1226 | i2_LQ_HNbud_c21995/f1p1/2271 |
| i2_LQ_HNbud_c7877/f1p1/3020 | i1_HQ_HNbud_c242706/f6p0/1904 | i1_LQ_HNbud_c27041/f1p0/1712 | i2_LQ_HNbud_c27912/f1p1/2763 |
| i3_LQ_HNbud_c11848/f1p0/3486 | i0_LQ_HNbud_c4198/f1p0/600 | i0_LQ_HNbud_c11083/f1p1/935 | i2_LQ_HNbud_c29906/f1p2/2008 |
| i0_HQ_HNbud_c7137/f2p8/933 | i3_LQ_HNbud_c5142/f1p2/3490 | i2_LQ_HNbud_c22659/f1p0/2056 | i1_LQ_HNbud_c127624/f2p3/1761 |
| i1_LQ_HNbud_c90460/f1p4/1716 | i1_LQ_HNbud_c52241/f1p0/1589 | i1_LQ_HNbud_c98090/f1p0/1108 | i3_LQ_HNbud_c3419/f1p5/3145 |
| i1_LQ_HNbud_c263005/f1p1/1066 | i1_LQ_HNbud_c262723/f1p3/1015 | i1_LQ_HNbud_c247382/f1p0/1377 | i1_LQ_HNbud_c50350/f3p9/2010 |
| i2_HQ_HNbud_c45245/f7p1/2386 | i0_HQ_HNbud_c12592/f6p1/817 | i2_LQ_HNbud_c39459/f1p1/2345 | i2_LQ_HNbud_c3955/f1p1/2182 |
| i2_LQ_HNbud_c52911/f1p3/2373 | i1_HQ_HNbud_c17717/f8p0/1260 | i1_LQ_HNbud_c53303/f1p3/1628 | i2_LQ_HNbud_c16234/f1p4/2047 |
| i2_LQ_HNbud_c19825/f1p0/2395 | i1_HQ_HNbud_c110594/f2p0/1942 | i1_HQ_HNbud_c245041/f5p1/1200 | i3_LQ_HNbud_c13376/f1p10/3261 |
| i2_LQ_HNbud_c16163/f1p1/2040 | i3_HQ_HNbud_c16999/f6p0/3049 | i1_LQ_HNbud_c84627/f1p3/1566 | i1_HQ_HNbud_c264403/f4p0/1370 |
| i3_LQ_HNbud_c6891/f1p4/3412 | i2_LQ_HNbud_c1619/f1p0/2926 | i3_LQ_HNbud_c16003/f1p2/3056 | i0_HQ_HNbud_c27365/f2p0/937 |
| i2_HQ_HNbud_c29070/f2p25/2906 | i1_LQ_HNbud_c74817/f1p1/1782 | i0_LQ_HNbud_c72703/f1p43/892 | i2_LQ_HNbud_c52526/f1p4/2118 |
| i1_HQ_HNbud_c263518/f3p7/1369 | i2_LQ_HNbud_c41705/f1p6/2613 | i3_LQ_HNbud_c11279/f1p13/3598 | i3_HQ_HNbud_c1855/f2p0/3738 |
| i1_LQ_HNbud_c8177/f1p1/1875 | i0_LQ_HNbud_c5071/f1p2/725 | i1_LQ_HNbud_c24776/f7p28/1855 | i1_HQ_HNbud_c18598/f2p4/1305 |
| i1_HQ_HNbud_c19049/f4p5/1259 | i0_LQ_HNbud_c6927/f2p1/785 | i1_LQ_HNbud_c39225/f1p0/1340 | i2_LQ_HNbud_c9807/f1p0/2751 |
| i2_LQ_HNbud_c50766/f1p0/2107 | i0_HQ_HNbud_c18464/f4p0/524 | i1_HQ_HNbud_c64676/f38p25/1329 | i0_HQ_HNbud_c48082/f2p0/931 |
| i3_LQ_HNbud_c15566/f1p0/3030 | i0_HQ_HNbud_c2356/f5p7/818 | i1_LQ_HNbud_c17628/f3p0/1194 | i2_LQ_HNbud_c61834/f1p0/2049 |
| i3_LQ_HNbud_c10067/f1p1/3451 | i0_LQ_HNbud_c24647/f2p0/921 | i3_HQ_HNbud_c1100/f4p0/3352 | i0_HQ_HNbud_c10839/f2p0/824 |
| i1_LQ_HNbud_c224971/f1p7/1768 | i1_LQ_HNbud_c53312/f1p0/1497 | i1_HQ_HNbud_c9830/f3p1/1620 | i1_LQ_HNbud_c153712/f1p2/1614 |
| i2_LQ_HNbud_c7758/f1p1/2798 | i2_LQ_HNbud_c22674/f1p0/2687 | i0_LQ_HNbud_c23805/f1p7/745 | i5_LQ_HNbud_c788/f1p0/5415 |
| i1_LQ_HNbud_c154146/f1p4/1870 | i1_LQ_HNbud_c130576/f1p8/1313 | i1_LQ_HNbud_c178817/f1p1/1539 | i0_HQ_HNbud_c17201/f11p0/786 |
| i2_LQ_HNbud_c30523/f1p0/2041 | i1_LQ_HNbud_c90968/f2p0/1741 | i1_LQ_HNbud_c25126/f1p4/1418 | i1_LQ_HNbud_c55124/f1p3/1826 |
| i3_LQ_HNbud_c10619/f1p4/3536 | i0_LQ_HNbud_c46273/f1p0/781 | i2_LQ_HNbud_c18257/f2p18/2348 | i2_LQ_HNbud_c28188/f1p0/2496 |
| i0_LQ_HNbud_c30589/f2p0/806 | i0_HQ_HNbud_c909/f15p4/811 | i1_LQ_HNbud_c13928/f1p0/1307 | i1_LQ_HNbud_c231937/f1p1/1791 |
| i2_LQ_HNbud_c59982/f1p2/2714 | i1_HQ_HNbud_c219182/f3p10/1789 | i1_HQ_HNbud_c36872/f5p0/1553 | i1_LQ_HNbud_c228604/f1p0/1369 |
| i0_LQ_HNbud_c72648/f1p0/470 | i2_HQ_HNbud_c65203/f26p5/2530 | i0_LQ_HNbud_c83938/f1p0/930 | i1_LQ_HNbud_c28048/f1p1/1341 |
| i1_LQ_HNbud_c72870/f1p0/1729 | i2_LQ_HNbud_c43127/f1p6/2584 | i2_LQ_HNbud_c51671/f1p5/2156 | i1_LQ_HNbud_c107750/f1p2/1422 |
| i1_HQ_HNbud_c270372/f4p2/1123 | i0_LQ_HNbud_c114921/f1p0/990 | i1_HQ_HNbud_c73305/f2p2/1737 | i1_LQ_HNbud_c15242/f1p2/1020 |
| i1_LQ_HNbud_c204418/f1p3/1724 | i2_HQ_HNbud_c33473/f2p12/2548 | i2_LQ_HNbud_c21612/f1p8/2944 | i0_LQ_HNbud_c67228/f1p0/959 |
| i2_LQ_HNbud_c61746/f2p0/2093 | i1_HQ_HNbud_c66788/f2p11/1801 | i1_HQ_HNbud_c81085/f30p5/1377 | i1_LQ_HNbud_c151158/f1p2/1308 |
| i3_LQ_HNbud_c3178/f1p12/3733 | i1_LQ_HNbud_c195958/f1p3/1290 | i1_HQ_HNbud_c245369/f5p1/1090 | i1_LQ_HNbud_c198919/f1p3/1532 |
| i2_LQ_HNbud_c21171/f1p16/2619 | i2_LQ_HNbud_c8155/f1p17/2390 | i2_LQ_HNbud_c23543/f1p4/2232 | i3_LQ_HNbud_c4707/f1p3/3794 |
| i1_HQ_HNbud_c51789/f3p9/1311 | i0_LQ_HNbud_c9295/f1p0/790 | i2_LQ_HNbud_c30279/f1p13/1975 | i1_LQ_HNbud_c61034/f1p1/1037 |
| i1_LQ_HNbud_c203842/f1p1/1226 | i1_LQ_HNbud_c177509/f1p1/1701 | i1_LQ_HNbud_c70912/f1p0/1392 | i2_LQ_HNbud_c40561/f1p12/2072 |
| i5_LQ_HNbud_c1862/f1p0/5054 | i0_LQ_HNbud_c30122/f1p2/928 | i2_LQ_HNbud_c15244/f1p2/2041 | i1_HQ_HNbud_c144841/f13p2/1640 |
| i0_LQ_HNbud_c41152/f1p3/877 | i1_LQ_HNbud_c108713/f1p1/1888 | i2_LQ_HNbud_c70898/f2p0/2071 | i2_LQ_HNbud_c19467/f1p4/2632 |
| i0_HQ_HNbud_c18393/f2p1/710 | i4_LQ_HNbud_c1919/f1p2/4599 | i2_HQ_HNbud_c64915/f7p11/2184 | i2_LQ_HNbud_c7256/f1p3/2413 |
| i2_LQ_HNbud_c12903/f1p1/2699 | i1_LQ_HNbud_c41617/f1p0/1385 | i3_HQ_HNbud_c1198/f3p3/3677 | i1_LQ_HNbud_c174828/f1p0/1579 |
| i1_LQ_HNbud_c26281/f1p0/1906 | i1_HQ_HNbud_c243338/f10p11/1157 | i1_HQ_HNbud_c18128/f2p1/1817 | i1_HQ_HNbud_c128608/f2p0/1181 |
| i1_HQ_HNbud_c216859/f6p0/1640 | i1_LQ_HNbud_c105131/f1p2/1465 | i1_HQ_HNbud_c131247/f2p0/1294 | i1_LQ_HNbud_c278022/f1p0/1001 |
| i0_LQ_HNbud_c18382/f4p2/841 | i1_LQ_HNbud_c42039/f2p18/1340 | i0_LQ_HNbud_c30106/f1p0/922 | i1_LQ_HNbud_c72804/f2p0/1797 |
| i2_HQ_HNbud_c23070/f2p2/2786 | i2_LQ_HNbud_c26486/f1p3/2850 | i0_LQ_HNbud_c80597/f1p0/709 | i0_HQ_HNbud_c13014/f4p0/881 |
| i1_LQ_HNbud_c91503/f1p3/1837 | i2_LQ_HNbud_c59542/f1p0/2502 | i2_HQ_HNbud_c1839/f6p5/2194 | i3_LQ_HNbud_c9750/f1p22/3681 |
| i3_HQ_HNbud_c17030/f2p0/3062 | i1_LQ_HNbud_c52287/f1p1/1889 | i1_HQ_HNbud_c182299/f7p2/1172 | i3_HQ_HNbud_c15171/f3p0/3044 |
| i3_LQ_HNbud_c2109/f1p2/3624 | i1_LQ_HNbud_c118395/f1p2/1098 | i1_LQ_HNbud_c151487/f1p9/1973 | i0_HQ_HNbud_c13858/f5p1/847 |
| i2_LQ_HNbud_c50244/f1p10/2373 | i1_HQ_HNbud_c2434/f24p7/1227 | i0_HQ_HNbud_c64673/f4p0/715 | i1_LQ_HNbud_c187724/f1p1/1018 |
| i1_LQ_HNbud_c195875/f1p3/1705 | i0_LQ_HNbud_c9713/f1p2/996 | i1_LQ_HNbud_c92940/f1p1/1978 | i2_LQ_HNbud_c11260/f1p10/2300 |
| i1_LQ_HNbud_c257232/f1p8/1652 | i0_LQ_HNbud_c53964/f1p4/794 | i1_LQ_HNbud_c56047/f1p0/1177 | i1_LQ_HNbud_c74173/f1p18/1400 |
| i2_LQ_HNbud_c38639/f1p5/2841 | i0_LQ_HNbud_c49067/f1p4/994 | i1_HQ_HNbud_c9638/f4p8/1832 | i1_LQ_HNbud_c203487/f1p13/1671 |
| i2_LQ_HNbud_c51892/f1p4/2550 | i1_LQ_HNbud_c149298/f1p2/1219 | i0_LQ_HNbud_c72215/f1p0/722 | i1_HQ_HNbud_c47286/f11p0/1426 |
| i4_HQ_HNbud_c4367/f6p1/4832 | i1_LQ_HNbud_c126921/f1p32/1741 | i3_LQ_HNbud_c9379/f1p0/3137 | i1_HQ_HNbud_c54691/f3p4/1473 |
| i1_HQ_HNbud_c25275/f5p4/1292 | i1_LQ_HNbud_c278370/f1p26/1014 | i0_LQ_HNbud_c67316/f1p5/937 | i1_LQ_HNbud_c7172/f1p5/1171 |
| i1_LQ_HNbud_c136674/f2p6/1045 | i1_LQ_HNbud_c15838/f1p2/1031 | i2_LQ_HNbud_c28644/f1p0/2456 | i0_HQ_HNbud_c2282/f5p1/956 |
| i2_LQ_HNbud_c11835/f1p15/2748 | i2_LQ_HNbud_c35564/f1p0/2844 | i2_LQ_HNbud_c73537/f1p2/2005 | i2_LQ_HNbud_c2920/f1p1/2095 |
| i0_HQ_HNbud_c97819/f11p0/803 | i2_LQ_HNbud_c3753/f1p8/2356 | i2_LQ_HNbud_c20895/f1p1/2383 | i1_LQ_HNbud_c55165/f1p1/1122 |
| i1_LQ_HNbud_c85101/f1p6/1376 | i1_HQ_HNbud_c25966/f4p9/1428 | i1_LQ_HNbud_c148006/f1p8/1660 | i0_LQ_HNbud_c4830/f1p1/586 |
| i1_LQ_HNbud_c163071/f1p3/1062 | i2_HQ_HNbud_c70880/f3p3/2105 | i1_LQ_HNbud_c230388/f1p2/1875 | i1_LQ_HNbud_c229537/f1p19/1209 |
| i3_LQ_HNbud_c6711/f1p0/3715 | i1_HQ_HNbud_c264722/f11p9/1244 | i1_LQ_HNbud_c40217/f1p2/1937 | i4_LQ_HNbud_c1707/f1p0/4361 |
| i2_LQ_HNbud_c73484/f1p1/2005 | i4_LQ_HNbud_c3986/f1p8/4057 | i2_HQ_HNbud_c33460/f2p9/2130 | i1_LQ_HNbud_c5996/f1p26/2165 |
| i1_HQ_HNbud_c77939/f2p5/1354 | i1_LQ_HNbud_c208615/f2p0/1113 | i2_LQ_HNbud_c13462/f1p10/2976 | i0_LQ_HNbud_c45481/f1p77/1001 |
| i1_HQ_HNbud_c70352/f2p0/1959 | i1_LQ_HNbud_c110063/f1p1/1882 | i1_LQ_HNbud_c68033/f1p1/1294 | i1_HQ_HNbud_c72722/f3p2/1528 |
| i5_LQ_HNbud_c1103/f1p0/5426 | i2_LQ_HNbud_c2263/f3p12/2517 | i2_LQ_HNbud_c27270/f1p5/2557 | i1_LQ_HNbud_c99737/f1p0/1086 |
| i0_HQ_HNbud_c7338/f3p3/789 | i2_LQ_HNbud_c24465/f1p1/2160 | i1_HQ_HNbud_c40481/f3p4/1378 | i2_LQ_HNbud_c14031/f1p12/2077 |
| i1_HQ_HNbud_c50865/f2p0/1218 | i1_HQ_HNbud_c33414/f4p4/1544 | i1_LQ_HNbud_c27515/f1p7/1392 | i3_LQ_HNbud_c13763/f1p7/3418 |
| i1_LQ_HNbud_c12935/f2p1/1275 | i2_HQ_HNbud_c21399/f3p2/2368 | i0_LQ_HNbud_c18760/f8p3/1010 | i1_LQ_HNbud_c133862/f1p0/1420 |
| i3_LQ_HNbud_c7733/f1p2/3361 | i1_HQ_HNbud_c106679/f4p5/1570 | i1_LQ_HNbud_c154658/f1p17/1392 | i2_LQ_HNbud_c2977/f1p1/2972 |
| i1_LQ_HNbud_c1530/f10p2/1706 | i3_LQ_HNbud_c4886/f1p3/3427 | i1_LQ_HNbud_c49862/f1p4/1474 | i3_LQ_HNbud_c18623/f1p0/3008 |
| i2_LQ_HNbud_c10147/f1p8/2795 | i2_LQ_HNbud_c39768/f1p41/2513 | i1_LQ_HNbud_c6464/f1p0/1892 | i3_LQ_HNbud_c10235/f1p0/3579 |
| i1_HQ_HNbud_c53467/f8p2/1719 | i1_LQ_HNbud_c230366/f1p4/1734 | i1_LQ_HNbud_c21700/f1p3/1444 | i2_HQ_HNbud_c20820/f2p1/2313 |
| i1_HQ_HNbud_c82864/f17p0/1268 | i1_LQ_HNbud_c125754/f1p6/1903 | i2_LQ_HNbud_c27827/f1p1/3025 | i2_HQ_HNbud_c64698/f4p0/2124 |
| i1_LQ_HNbud_c52012/f1p1/1099 | i4_LQ_HNbud_c1571/f1p0/4710 | i0_LQ_HNbud_c8064/f1p0/846 | i0_LQ_HNbud_c49299/f1p5/929 |
| i4_LQ_HNbud_c3161/f1p7/4677 | i2_HQ_HNbud_c47550/f13p2/2328 | i0_LQ_HNbud_c19539/f1p1/578 | i2_LQ_HNbud_c51938/f1p0/2127 |
| i2_LQ_HNbud_c59251/f1p2/2300 | i0_LQ_HNbud_c42017/f1p0/614 | i2_LQ_HNbud_c25840/f1p1/2235 | i2_HQ_HNbud_c33376/f4p4/2564 |
| i2_LQ_HNbud_c55664/f1p9/2112 | i0_LQ_HNbud_c81870/f1p0/893 | i1_HQ_HNbud_c54614/f2p8/1830 | i1_LQ_HNbud_c90192/f1p2/1570 |
| i2_LQ_HNbud_c38608/f1p0/2144 | i1_HQ_HNbud_c17448/f3p1/1519 | i1_HQ_HNbud_c38024/f2p8/1875 | i0_LQ_HNbud_c26802/f1p3/810 |
| i2_LQ_HNbud_c59962/f1p0/2369 | i1_LQ_HNbud_c148691/f1p4/1165 | i1_LQ_HNbud_c174076/f1p0/1363 | i2_HQ_HNbud_c26708/f3p2/2180 |
| i1_HQ_HNbud_c243578/f5p1/1099 | i1_LQ_HNbud_c21581/f1p21/1708 | i2_LQ_HNbud_c35096/f1p9/2920 | i0_LQ_HNbud_c76425/f1p0/798 |
| i1_LQ_HNbud_c25165/f1p9/1228 | i1_LQ_HNbud_c260946/f1p1/1063 | i1_HQ_HNbud_c264086/f14p2/1315 | i1_HQ_HNbud_c29177/f4p9/1330 |
| i1_HQ_HNbud_c38093/f2p2/1759 | i0_LQ_HNbud_c12806/f1p0/749 | i3_LQ_HNbud_c15911/f1p0/3031 | i0_LQ_HNbud_c29015/f1p1/985 |
| i5_LQ_HNbud_c1656/f1p3/5408 | i2_LQ_HNbud_c15530/f1p35/1912 | i0_LQ_HNbud_c9161/f1p1/638 | i2_LQ_HNbud_c60113/f1p1/2305 |
| i0_LQ_HNbud_c23790/f1p13/927 | i0_LQ_HNbud_c43724/f1p2/907 | i1_HQ_HNbud_c3860/f5p1/1394 | i1_LQ_HNbud_c33917/f1p4/1250 |
| i1_LQ_HNbud_c7459/f1p23/1154 | i1_LQ_HNbud_c264020/f1p2/1499 | i1_HQ_HNbud_c25395/f4p2/1285 | i1_LQ_HNbud_c153316/f1p0/1157 |
| i1_LQ_HNbud_c71752/f1p39/1920 | i1_LQ_HNbud_c73633/f1p1/1853 | i1_HQ_HNbud_c234560/f3p2/1073 | i1_LQ_HNbud_c196991/f1p6/1545 |
| i1_LQ_HNbud_c8531/f1p10/1385 | i2_LQ_HNbud_c51419/f1p7/2832 | i2_HQ_HNbud_c17879/f3p0/2121 | i1_LQ_HNbud_c98718/f1p1/1061 |
| i0_LQ_HNbud_c6391/f2p1/971 | i2_HQ_HNbud_c1354/f5p2/2349 | i0_LQ_HNbud_c26411/f1p0/844 | i1_LQ_HNbud_c25196/f1p4/1728 |
| i4_LQ_HNbud_c2720/f1p0/4117 | i2_LQ_HNbud_c53564/f1p1/2955 | i2_LQ_HNbud_c19930/f1p4/2711 | i3_LQ_HNbud_c1852/f2p5/3542 |
| i0_LQ_HNbud_c7648/f1p2/756 | i1_LQ_HNbud_c205917/f1p4/1323 | i1_LQ_HNbud_c9839/f1p12/1485 | i2_LQ_HNbud_c10649/f1p0/2565 |
| i1_HQ_HNbud_c53829/f2p9/1288 | i1_HQ_HNbud_c23822/f2p3/1861 | i2_HQ_HNbud_c66247/f21p8/2621 | i2_LQ_HNbud_c40389/f1p1/2287 |
| i4_LQ_HNbud_c1485/f1p1/4537 | i0_HQ_HNbud_c25205/f2p8/651 | i1_HQ_HNbud_c3361/f5p0/1876 | i1_LQ_HNbud_c132373/f1p5/1157 |
| i2_LQ_HNbud_c37847/f1p12/2081 | i1_LQ_HNbud_c221631/f1p5/1293 | i1_HQ_HNbud_c5438/f3p5/1290 | i1_LQ_HNbud_c214726/f1p5/1025 |
| i1_HQ_HNbud_c12901/f4p2/1139 | i1_HQ_HNbud_c151701/f3p3/1816 | i2_LQ_HNbud_c68336/f1p25/2062 | i3_LQ_HNbud_c10817/f1p7/3504 |
| i1_HQ_HNbud_c11736/f3p6/1142 | i1_HQ_HNbud_c193842/f56p12/1671 | i1_HQ_HNbud_c12558/f4p1/1916 | i1_LQ_HNbud_c226850/f1p1/1621 |
| i2_HQ_HNbud_c34757/f3p5/2617 | i5_LQ_HNbud_c130/f2p0/5229 | i0_LQ_HNbud_c30008/f1p3/832 | i4_LQ_HNbud_c2535/f1p0/4636 |
| i2_HQ_HNbud_c7894/f2p3/2757 | i3_LQ_HNbud_c16518/f1p0/3044 | i3_LQ_HNbud_c4647/f1p2/3122 | i0_HQ_HNbud_c2019/f8p0/886 |
| i3_LQ_HNbud_c9121/f1p1/3205 | i2_LQ_HNbud_c15874/f1p2/2174 | i0_LQ_HNbud_c26797/f1p0/905 | i0_LQ_HNbud_c28725/f1p240/732 |
| i1_LQ_HNbud_c156865/f1p2/1164 | i3_HQ_HNbud_c683/f8p0/3225 | i1_LQ_HNbud_c86262/f1p6/1185 | i1_LQ_HNbud_c50054/f1p5/1621 |
| i0_LQ_HNbud_c53612/f1p0/510 | i1_LQ_HNbud_c11404/f1p4/1555 | i2_LQ_HNbud_c26507/f1p0/2082 | i2_HQ_HNbud_c3272/f4p4/2239 |
| i2_LQ_HNbud_c54097/f1p7/2821 | i2_LQ_HNbud_c36028/f1p4/2078 | i0_LQ_HNbud_c6622/f1p0/612 | i1_LQ_HNbud_c109624/f1p2/1727 |
| i1_LQ_HNbud_c35057/f1p2/1632 | i3_LQ_HNbud_c3881/f1p0/3471 | i0_LQ_HNbud_c8970/f2p4/933 | i1_LQ_HNbud_c172063/f1p3/1429 |
| i6_LQ_HNbud_c436/f1p0/6819 | i1_HQ_HNbud_c3228/f12p4/1218 | i1_LQ_HNbud_c116153/f1p5/1032 | i2_HQ_HNbud_c18063/f4p8/2286 |
| i1_LQ_HNbud_c19141/f1p2/1959 | i1_HQ_HNbud_c74896/f8p3/1288 | i1_HQ_HNbud_c102230/f25p17/1388 | i0_LQ_HNbud_c46934/f1p0/900 |
| i0_HQ_HNbud_c8597/f4p3/998 | i0_LQ_HNbud_c31896/f2p0/873 | i4_LQ_HNbud_c1479/f1p0/4443 | i0_LQ_HNbud_c13018/f1p0/298 |
| i0_HQ_HNbud_c20467/f2p1/536 | i1_LQ_HNbud_c89797/f2p1/1721 | i1_LQ_HNbud_c279971/f1p0/1011 | i0_LQ_HNbud_c3329/f2p0/915 |
| i1_LQ_HNbud_c75561/f1p0/1203 | i3_LQ_HNbud_c13134/f1p3/3938 | i2_LQ_HNbud_c28461/f1p0/2062 | i3_HQ_HNbud_c1879/f2p4/3456 |
| i1_LQ_HNbud_c176291/f1p8/1213 | i1_LQ_HNbud_c90829/f1p16/1724 | i2_HQ_HNbud_c11986/f4p1/2885 | i0_LQ_HNbud_c85835/f2p8/645 |
| i1_LQ_HNbud_c103987/f1p3/1246 | i1_HQ_HNbud_c233370/f7p2/1069 | i1_LQ_HNbud_c38929/f1p5/1754 | i1_LQ_HNbud_c112640/f1p7/1679 |
| i2_LQ_HNbud_c47090/f1p0/2005 | i1_LQ_HNbud_c75369/f1p0/1284 | i1_HQ_HNbud_c218499/f11p5/1180 | i1_LQ_HNbud_c17792/f3p2/1749 |
| i3_LQ_HNbud_c11762/f1p2/3339 | i0_HQ_HNbud_c98889/f33p0/668 | i1_LQ_HNbud_c21982/f1p0/1144 | i1_LQ_HNbud_c23325/f1p10/1693 |
| i1_HQ_HNbud_c130868/f2p5/1726 | i1_HQ_HNbud_c143363/f6p3/1042 | i1_HQ_HNbud_c195250/f3p3/1930 | i1_LQ_HNbud_c67957/f1p2/1305 |
| i3_LQ_HNbud_c16364/f1p8/3033 | i1_LQ_HNbud_c152223/f1p3/1810 | i1_LQ_HNbud_c108341/f1p15/1923 | i1_LQ_HNbud_c149633/f1p2/1285 |
| i2_LQ_HNbud_c53618/f1p2/2145 | i2_LQ_HNbud_c73684/f1p21/1931 | i1_LQ_HNbud_c203094/f1p141/1905 | i2_LQ_HNbud_c37377/f1p1/2160 |
| i1_LQ_HNbud_c84344/f1p0/1105 | i2_LQ_HNbud_c34571/f1p6/2524 | i2_LQ_HNbud_c12685/f1p1/2463 | i1_HQ_HNbud_c121787/f3p4/1104 |
| i1_LQ_HNbud_c198586/f1p3/1669 | i1_LQ_HNbud_c228435/f1p1/1939 | i1_LQ_HNbud_c51215/f1p4/1405 | i1_LQ_HNbud_c90524/f2p1/1984 |
| i2_LQ_HNbud_c24507/f1p7/2560 | i1_LQ_HNbud_c153220/f1p0/1615 | i0_LQ_HNbud_c53772/f1p7/823 | i3_LQ_HNbud_c14989/f1p0/3181 |
| i1_LQ_HNbud_c119289/f1p0/1044 | i1_HQ_HNbud_c208891/f2p3/1076 | i4_LQ_HNbud_c4141/f1p0/4053 | i2_LQ_HNbud_c51257/f1p4/2106 |
| i3_LQ_HNbud_c4726/f1p0/3167 | i0_LQ_HNbud_c9667/f1p0/879 | i1_LQ_HNbud_c53356/f1p5/1748 | i1_HQ_HNbud_c34378/f2p2/1296 |
| i1_LQ_HNbud_c36252/f1p0/1116 | i1_LQ_HNbud_c98562/f1p45/894 | i1_HQ_HNbud_c33776/f2p2/1810 | i0_HQ_HNbud_c812/f20p0/764 |
| i1_LQ_HNbud_c18543/f1p2/1523 | i1_LQ_HNbud_c15266/f1p3/1108 | i1_LQ_HNbud_c44717/f1p0/1087 | i1_HQ_HNbud_c75053/f7p6/1768 |
| i1_LQ_HNbud_c89457/f1p10/1163 | i1_LQ_HNbud_c153883/f1p4/1795 | i4_LQ_HNbud_c4291/f1p2/4076 | i1_LQ_HNbud_c110413/f1p4/1398 |
| i2_LQ_HNbud_c23050/f1p0/2355 | i1_HQ_HNbud_c17495/f3p2/1841 | i2_HQ_HNbud_c33340/f3p3/2744 | i0_LQ_HNbud_c39452/f3p2/907 |
| i1_LQ_HNbud_c152395/f1p6/1172 | i4_LQ_HNbud_c1283/f1p3/4800 | i3_LQ_HNbud_c3740/f1p7/3743 | i3_LQ_HNbud_c5715/f1p0/3205 |
| i1_HQ_HNbud_c75020/f3p1/1117 | i0_HQ_HNbud_c42337/f2p0/963 | i1_LQ_HNbud_c162103/f1p3/1097 | i1_HQ_HNbud_c242759/f2p1/1431 |
| i2_LQ_HNbud_c57338/f1p0/2291 | i3_LQ_HNbud_c12757/f1p0/3208 | i2_LQ_HNbud_c54029/f1p11/2352 | i1_LQ_HNbud_c26519/f1p2/1691 |
| i3_LQ_HNbud_c6970/f1p5/3676 | i3_LQ_HNbud_c7552/f1p0/3894 | i2_LQ_HNbud_c63085/f1p0/2049 | i1_LQ_HNbud_c223863/f1p1/1497 |
| i0_LQ_HNbud_c21561/f1p3/363 | i2_HQ_HNbud_c41932/f2p6/2137 | i1_HQ_HNbud_c21335/f7p2/1827 | i1_HQ_HNbud_c66959/f3p7/1208 |
| i1_HQ_HNbud_c267527/f2p6/1434 | i0_HQ_HNbud_c46973/f2p0/646 | i3_LQ_HNbud_c2361/f1p3/3238 | i1_LQ_HNbud_c204916/f1p9/1743 |
| i1_LQ_HNbud_c24838/f10p6/1741 | i3_LQ_HNbud_c8428/f1p1/4020 | i1_LQ_HNbud_c146752/f1p5/1616 | i1_LQ_HNbud_c128329/f1p0/1130 |
| i2_LQ_HNbud_c57231/f1p3/2948 | i1_LQ_HNbud_c108326/f1p2/1672 | i2_LQ_HNbud_c26028/f1p0/2937 | i1_LQ_HNbud_c68069/f1p1/1994 |
| i2_LQ_HNbud_c50474/f1p1/2152 | i1_LQ_HNbud_c222671/f1p11/1659 | i0_HQ_HNbud_c5478/f3p1/723 | i0_LQ_HNbud_c41530/f1p5/811 |
| i0_HQ_HNbud_c1213/f10p12/853 | i1_LQ_HNbud_c280702/f1p3/1006 | i4_LQ_HNbud_c485/f1p0/4123 | i0_HQ_HNbud_c64737/f5p0/614 |
| i1_LQ_HNbud_c18591/f1p2/1736 | i1_HQ_HNbud_c35477/f2p0/1286 | i0_HQ_HNbud_c2116/f5p3/905 | i1_LQ_HNbud_c170791/f1p6/1224 |
| i1_LQ_HNbud_c74455/f1p29/1978 | i4_LQ_HNbud_c2837/f1p2/4124 | i2_HQ_HNbud_c50393/f3p4/2638 | i2_LQ_HNbud_c21856/f1p2/2657 |
| i2_LQ_HNbud_c3457/f1p0/2401 | i0_LQ_HNbud_c74235/f1p1/929 | i2_LQ_HNbud_c56037/f1p1/2472 | i0_LQ_HNbud_c78713/f1p5/979 |
| i1_LQ_HNbud_c226383/f1p0/1316 | i2_LQ_HNbud_c50870/f1p0/2549 | i1_LQ_HNbud_c90849/f1p5/1585 | i3_LQ_HNbud_c12803/f1p15/3159 |
| i1_HQ_HNbud_c265122/f127p32/1731 | i1_LQ_HNbud_c55041/f1p0/1505 | i0_HQ_HNbud_c97759/f32p0/849 | i1_LQ_HNbud_c60523/f1p30/1020 |
| i1_HQ_HNbud_c25202/f6p17/1185 | i1_LQ_HNbud_c133377/f1p4/1258 | i1_HQ_HNbud_c63015/f3p0/1391 | i1_LQ_HNbud_c106945/f1p2/1263 |
| i0_LQ_HNbud_c48738/f1p3/1000 | i1_LQ_HNbud_c130482/f1p1/1211 | i0_LQ_HNbud_c13067/f3p2/903 | i1_HQ_HNbud_c70475/f2p2/1774 |
| i1_LQ_HNbud_c226630/f1p9/1731 | i3_HQ_HNbud_c16985/f3p0/3143 | i2_HQ_HNbud_c14321/f2p1/2235 | i1_LQ_HNbud_c104361/f1p5/977 |
| i2_LQ_HNbud_c14995/f2p0/2067 | i0_LQ_HNbud_c50129/f2p7/681 | i2_LQ_HNbud_c15742/f1p3/2035 | i2_LQ_HNbud_c5570/f1p261/2793 |
| i2_LQ_HNbud_c14993/f1p3/2020 | i1_LQ_HNbud_c91203/f1p6/1464 | i1_HQ_HNbud_c17452/f19p77/1990 | i1_LQ_HNbud_c74545/f1p3/1534 |
| i2_HQ_HNbud_c19745/f2p1/2121 | i3_LQ_HNbud_c12033/f1p0/3300 | i2_HQ_HNbud_c70150/f3p0/2165 | i2_LQ_HNbud_c59130/f1p0/2120 |
| i1_LQ_HNbud_c38336/f2p1/1907 | i1_LQ_HNbud_c74007/f1p3/1815 | i3_HQ_HNbud_c16874/f4p0/3546 | i1_HQ_HNbud_c17401/f2p0/1598 |
| i1_LQ_HNbud_c90558/f1p3/1554 | i2_LQ_HNbud_c27242/f2p1/2426 | i2_LQ_HNbud_c40397/f1p4/2130 | i1_LQ_HNbud_c106180/f1p6/1202 |
| i1_HQ_HNbud_c131963/f4p7/1798 | i1_LQ_HNbud_c132268/f1p0/1724 | i1_HQ_HNbud_c254844/f8p31/1016 | i2_HQ_HNbud_c4963/f2p0/2474 |
| i1_LQ_HNbud_c26332/f4p2/1890 | i3_LQ_HNbud_c8300/f1p0/3105 | i0_HQ_HNbud_c121096/f6p13/881 | i1_LQ_HNbud_c252041/f1p0/1291 |
| i1_LQ_HNbud_c24219/f1p1/1366 | i1_LQ_HNbud_c56795/f1p8/1503 | i1_LQ_HNbud_c179716/f1p1/1147 | i2_LQ_HNbud_c13629/f1p2/2287 |
| i2_LQ_HNbud_c11347/f1p0/2114 | i2_HQ_HNbud_c16390/f22p0/2827 | i4_LQ_HNbud_c3913/f1p5/4855 | i0_HQ_HNbud_c25124/f3p2/905 |
| i1_HQ_HNbud_c201730/f2p0/1614 | i2_HQ_HNbud_c47959/f12p4/2242 | i1_LQ_HNbud_c7187/f1p2/1452 | i1_LQ_HNbud_c149426/f1p0/1203 |
| i1_LQ_HNbud_c223397/f1p1/1324 | i1_LQ_HNbud_c279112/f1p4/1019 | i0_LQ_HNbud_c75136/f1p2/450 | i1_HQ_HNbud_c2654/f5p2/1735 |
| i1_LQ_HNbud_c188542/f1p0/1024 | i1_LQ_HNbud_c34840/f3p2/1799 | i0_LQ_HNbud_c23457/f1p0/974 | i1_HQ_HNbud_c65705/f2p5/2009 |
| i0_HQ_HNbud_c18431/f3p0/992 | i1_LQ_HNbud_c250656/f1p1/1481 | i1_LQ_HNbud_c10957/f1p5/1306 | i1_HQ_HNbud_c71915/f3p1/1160 |
| i4_LQ_HNbud_c3910/f1p81/4151 | i1_LQ_HNbud_c180083/f1p1/1978 | i2_HQ_HNbud_c28184/f2p10/2480 | i2_HQ_HNbud_c17882/f2p3/2167 |
| i3_LQ_HNbud_c7490/f1p8/3869 | i3_LQ_HNbud_c11187/f1p0/3140 | i1_LQ_HNbud_c51745/f1p6/1604 | i1_LQ_HNbud_c177273/f1p5/1170 |
| i1_LQ_HNbud_c6524/f1p3/1271 | i1_LQ_HNbud_c223344/f1p0/1162 | i0_HQ_HNbud_c98794/f15p0/739 | i1_LQ_HNbud_c116266/f1p3/1051 |
| i2_LQ_HNbud_c39645/f1p3/2776 | i1_LQ_HNbud_c151258/f1p0/1127 | i1_LQ_HNbud_c180307/f1p16/1106 | i1_LQ_HNbud_c131543/f1p0/1515 |
| i1_LQ_HNbud_c73611/f1p4/1364 | i2_LQ_HNbud_c56346/f1p6/2257 | i2_HQ_HNbud_c5761/f4p0/2413 | i3_LQ_HNbud_c13737/f1p1/3148 |
| i2_LQ_HNbud_c8979/f1p9/2181 | i1_HQ_HNbud_c170427/f7p1/1497 | i1_LQ_HNbud_c103730/f1p1/1384 | i2_LQ_HNbud_c59118/f1p1/2541 |
| i2_HQ_HNbud_c66167/f26p1/2411 | i1_LQ_HNbud_c61140/f1p0/1068 | i1_LQ_HNbud_c204146/f1p87/1162 | i3_LQ_HNbud_c10197/f1p6/3167 |
| i2_LQ_HNbud_c73772/f1p0/2010 | i1_HQ_HNbud_c216802/f5p1/1841 | i0_LQ_HNbud_c107503/f1p0/476 | i1_LQ_HNbud_c85765/f1p0/1982 |
| i2_LQ_HNbud_c25081/f2p12/2366 | i0_HQ_HNbud_c41229/f3p0/587 | i1_LQ_HNbud_c128719/f1p3/1233 | i1_LQ_HNbud_c213762/f1p4/1015 |
| i2_HQ_HNbud_c42568/f2p14/2573 | i0_LQ_HNbud_c83349/f1p0/921 | i1_HQ_HNbud_c10663/f2p13/1397 | i1_LQ_HNbud_c241444/f1p14/1072 |
| i0_LQ_HNbud_c40254/f1p25/412 | i0_LQ_HNbud_c29266/f1p0/880 | i3_LQ_HNbud_c7907/f1p4/3141 | i0_LQ_HNbud_c9018/f1p0/648 |
| i2_LQ_HNbud_c46378/f1p9/2027 | i2_LQ_HNbud_c7394/f1p2/2559 | i0_LQ_HNbud_c46533/f1p0/677 | i0_LQ_HNbud_c53924/f1p8/700 |
| i2_HQ_HNbud_c8051/f2p4/2794 | i2_HQ_HNbud_c40432/f2p0/2095 | i1_LQ_HNbud_c214751/f1p2/1026 | i1_LQ_HNbud_c178003/f1p2/1279 |
| i2_LQ_HNbud_c34541/f1p1/2482 | i2_LQ_HNbud_c46217/f1p2/1970 | i1_HQ_HNbud_c73349/f3p0/1178 | i1_LQ_HNbud_c33281/f1p11/1764 |
| i1_LQ_HNbud_c180122/f1p17/1342 | i0_LQ_HNbud_c41629/f1p2/932 | i1_LQ_HNbud_c125088/f1p6/1313 | i1_HQ_HNbud_c101990/f11p4/1364 |
| i1_HQ_HNbud_c264213/f2p3/1251 | i1_LQ_HNbud_c27336/f1p25/1445 | i2_LQ_HNbud_c18427/f1p3/2785 | i3_LQ_HNbud_c15005/f1p5/3103 |
| i0_LQ_HNbud_c5150/f1p9/992 | i2_LQ_HNbud_c2901/f1p2/2350 | i1_LQ_HNbud_c253406/f1p1/1553 | i1_LQ_HNbud_c108481/f1p0/1202 |
| i1_HQ_HNbud_c221848/f2p4/1910 | i2_LQ_HNbud_c42329/f1p0/2867 | i1_HQ_HNbud_c130376/f2p1/1432 | i1_LQ_HNbud_c3974/f1p4/1268 |
| i5_LQ_HNbud_c1490/f1p5/5355 | i1_HQ_HNbud_c17930/f2p0/1469 | i1_LQ_HNbud_c91247/f1p4/1388 | i2_LQ_HNbud_c42786/f1p1/2437 |
| i0_HQ_HNbud_c2371/f4p3/654 | i0_LQ_HNbud_c80556/f1p0/581 | i2_LQ_HNbud_c13145/f1p1/2153 | i5_LQ_HNbud_c1099/f1p9/5307 |
| i0_LQ_HNbud_c7689/f1p0/752 | i1_HQ_HNbud_c37435/f20p8/1759 | i1_LQ_HNbud_c155489/f1p8/1274 | i0_LQ_HNbud_c11193/f2p5/902 |
| i1_LQ_HNbud_c89498/f1p1/1237 | i0_LQ_HNbud_c28549/f1p1/796 | i1_LQ_HNbud_c109408/f1p2/1869 | i2_LQ_HNbud_c5670/f1p5/2347 |
| i0_LQ_HNbud_c98051/f2p0/558 | i0_LQ_HNbud_c20114/f1p1/913 | i1_LQ_HNbud_c112381/f2p4/1129 | i1_LQ_HNbud_c35545/f1p0/1528 |
| i1_HQ_HNbud_c263370/f31p5/1656 | i1_HQ_HNbud_c53305/f2p2/1937 | i1_LQ_HNbud_c65901/f1p7/1827 | i1_LQ_HNbud_c280573/f1p1/1043 |
| i3_LQ_HNbud_c7937/f1p2/3571 | i0_LQ_HNbud_c9345/f1p4/925 | i1_LQ_HNbud_c228294/f1p0/1658 | i2_LQ_HNbud_c56879/f1p10/2443 |
| i0_LQ_HNbud_c69199/f1p0/830 | i1_HQ_HNbud_c143113/f10p6/1366 | i0_LQ_HNbud_c53989/f1p28/808 | i1_LQ_HNbud_c56258/f1p0/1420 |
| i1_LQ_HNbud_c224763/f1p3/1764 | i2_HQ_HNbud_c14417/f2p0/2322 | i1_HQ_HNbud_c20950/f2p0/1877 | i1_LQ_HNbud_c170892/f1p0/1224 |
| i1_LQ_HNbud_c103975/f1p6/1923 | i1_HQ_HNbud_c243906/f11p1/1682 | i1_HQ_HNbud_c264518/f7p4/1099 | i1_LQ_HNbud_c88370/f1p0/1457 |
| i1_LQ_HNbud_c21191/f1p1/1210 | i1_HQ_HNbud_c33329/f2p4/1636 | i0_HQ_HNbud_c98531/f46p0/542 | i0_HQ_HNbud_c2438/f5p2/650 |
| i4_LQ_HNbud_c1160/f1p0/4176 | i1_LQ_HNbud_c6124/f1p0/1813 | i3_LQ_HNbud_c4940/f1p0/3547 | i1_LQ_HNbud_c85528/f1p4/1789 |
| i0_LQ_HNbud_c22602/f1p7/981 | i1_LQ_HNbud_c198546/f1p0/1235 | i0_LQ_HNbud_c67195/f1p0/649 | i0_LQ_HNbud_c84288/f1p0/802 |
| i1_LQ_HNbud_c198151/f1p4/1692 | i1_HQ_HNbud_c121424/f20p9/1646 | i0_LQ_HNbud_c8110/f1p4/935 | i1_LQ_HNbud_c162806/f1p6/1013 |
| i0_LQ_HNbud_c25957/f1p1/795 | i4_HQ_HNbud_c243/f2p0/4181 | i2_HQ_HNbud_c8091/f2p3/2112 | i3_LQ_HNbud_c17505/f1p0/3381 |
| i3_HQ_HNbud_c1943/f2p0/3130 | i1_HQ_HNbud_c195099/f2p0/1634 | i1_HQ_HNbud_c88053/f2p0/1470 | i2_HQ_HNbud_c18045/f3p5/2562 |
| i2_LQ_HNbud_c7158/f1p0/2631 | i1_LQ_HNbud_c177219/f1p7/1351 | i2_LQ_HNbud_c23772/f1p5/2950 | i2_LQ_HNbud_c37482/f1p2/2517 |
| i2_LQ_HNbud_c62723/f1p5/2052 | i1_LQ_HNbud_c226373/f1p14/1431 | i4_LQ_HNbud_c2769/f1p1/4641 | i1_LQ_HNbud_c21776/f1p1/1779 |
| i1_LQ_HNbud_c34101/f1p4/1331 | i2_HQ_HNbud_c45126/f3p3/2209 | i1_HQ_HNbud_c126336/f2p3/1117 | i1_LQ_HNbud_c33629/f1p3/1451 |
| i2_LQ_HNbud_c18599/f1p3/2587 | i1_HQ_HNbud_c265719/f21p4/1081 | i2_LQ_HNbud_c50197/f1p8/2372 | i1_LQ_HNbud_c37551/f1p7/1531 |
| i0_LQ_HNbud_c49273/f1p1/946 | i2_LQ_HNbud_c25513/f1p5/2519 | i3_LQ_HNbud_c12807/f1p0/3633 | i1_LQ_HNbud_c124498/f1p2/1744 |
| i2_LQ_HNbud_c51648/f1p15/2528 | i2_LQ_HNbud_c2464/f1p2/2294 | i1_LQ_HNbud_c129977/f1p2/1269 | i1_LQ_HNbud_c26495/f1p3/1605 |
| i1_LQ_HNbud_c35898/f1p1/1316 | i1_LQ_HNbud_c36151/f1p0/1370 | i2_LQ_HNbud_c52615/f1p0/2225 | i1_HQ_HNbud_c49771/f2p0/1159 |
| i1_LQ_HNbud_c40616/f1p6/1221 | i1_HQ_HNbud_c24438/f2p3/1697 | i2_LQ_HNbud_c6018/f1p0/2322 | i0_LQ_HNbud_c30027/f1p0/703 |
| i1_LQ_HNbud_c149772/f1p3/1720 | i1_HQ_HNbud_c30857/f12p1/1473 | i1_LQ_HNbud_c148323/f1p7/2012 | i2_HQ_HNbud_c18108/f2p0/2257 |
| i1_HQ_HNbud_c167861/f12p9/1437 | i2_LQ_HNbud_c29066/f1p2/2402 | i0_HQ_HNbud_c41787/f2p2/826 | i1_LQ_HNbud_c176114/f1p8/1383 |
| i2_LQ_HNbud_c34463/f1p20/2106 | i1_LQ_HNbud_c133053/f1p5/1345 | i0_HQ_HNbud_c13817/f2p27/778 | i2_LQ_HNbud_c52145/f1p0/2460 |
| i3_LQ_HNbud_c6094/f1p366/3398 | i1_LQ_HNbud_c173445/f1p3/1213 | i1_LQ_HNbud_c228400/f1p8/1647 | i1_LQ_HNbud_c222880/f1p6/1434 |
| i2_LQ_HNbud_c27385/f1p0/2340 | i2_LQ_HNbud_c37140/f1p0/2157 | i0_LQ_HNbud_c75751/f1p2/1001 | i1_LQ_HNbud_c106251/f1p2/1645 |
| i1_HQ_HNbud_c132276/f2p15/1321 | i1_HQ_HNbud_c152342/f2p1/1310 | i2_LQ_HNbud_c15578/f1p0/2054 | i0_LQ_HNbud_c85107/f1p2/813 |
| i1_HQ_HNbud_c50212/f2p3/1261 | i1_LQ_HNbud_c224995/f1p1/1617 | i1_LQ_HNbud_c74503/f1p1/1928 | i2_LQ_HNbud_c54442/f1p0/2276 |
| i1_LQ_HNbud_c75532/f1p2/1596 | i1_LQ_HNbud_c23618/f1p3/1696 | i1_HQ_HNbud_c216306/f23p16/1766 | i1_HQ_HNbud_c81227/f14p9/1594 |
| i1_HQ_HNbud_c100247/f7p2/1637 | i1_HQ_HNbud_c2595/f35p10/1892 | i2_LQ_HNbud_c40669/f1p2/2359 | i3_LQ_HNbud_c4253/f1p0/3088 |
| i1_HQ_HNbud_c216989/f2p2/1103 | i1_LQ_HNbud_c69143/f1p3/1720 | i0_HQ_HNbud_c2028/f4p0/888 | i0_LQ_HNbud_c97839/f18p0/749 |
| i1_LQ_HNbud_c172338/f1p4/1391 | i1_LQ_HNbud_c197340/f1p78/1066 | i1_LQ_HNbud_c151510/f1p6/1252 | i1_LQ_HNbud_c58491/f1p1/1717 |
| i0_HQ_HNbud_c18762/f10p0/642 | i2_LQ_HNbud_c23633/f1p0/2291 | i2_HQ_HNbud_c31821/f3p1/2081 | i0_LQ_HNbud_c41046/f1p6/842 |
| i0_LQ_HNbud_c103833/f1p0/654 | i2_LQ_HNbud_c42600/f1p0/2521 | i1_HQ_HNbud_c166137/f36p12/1654 | i2_HQ_HNbud_c70489/f12p3/2273 |
| i1_LQ_HNbud_c10430/f1p5/1305 | i0_LQ_HNbud_c71400/f1p0/717 | i1_LQ_HNbud_c35958/f1p6/1456 | i2_LQ_HNbud_c19743/f1p7/2248 |
| i4_LQ_HNbud_c4276/f1p2/4040 | i2_LQ_HNbud_c28794/f1p1/2850 | i2_LQ_HNbud_c53006/f1p0/2108 | i4_LQ_HNbud_c4043/f1p0/4047 |
| i1_HQ_HNbud_c90064/f2p2/1733 | i3_LQ_HNbud_c15679/f1p0/3074 | i0_LQ_HNbud_c52041/f1p7/604 | i0_HQ_HNbud_c64648/f29p0/482 |
| i2_HQ_HNbud_c49769/f2p5/2483 | i1_LQ_HNbud_c74719/f1p1/1351 | i1_HQ_HNbud_c14112/f14p4/1322 | i1_LQ_HNbud_c55574/f1p1/1425 |
| i2_LQ_HNbud_c53106/f1p6/2670 | i1_LQ_HNbud_c104774/f1p13/1511 | i2_LQ_HNbud_c43768/f1p9/2149 | i2_HQ_HNbud_c1360/f11p1/2693 |
| i1_HQ_HNbud_c28027/f2p4/1527 | i2_LQ_HNbud_c41271/f1p11/2562 | i2_HQ_HNbud_c1168/f6p1/2713 | i0_HQ_HNbud_c66788/f4p14/624 |
| i2_LQ_HNbud_c7667/f1p1/2718 | i0_LQ_HNbud_c22838/f1p8/692 | i1_LQ_HNbud_c69357/f1p1/1424 | i1_LQ_HNbud_c266723/f1p11/1157 |
| i1_LQ_HNbud_c195304/f1p5/1135 | i0_LQ_HNbud_c21182/f1p2/702 | i1_LQ_HNbud_c242514/f1p0/1083 | i1_LQ_HNbud_c107398/f1p0/1555 |
| i0_LQ_HNbud_c41074/f1p2/508 | i3_LQ_HNbud_c11647/f1p4/3509 | i3_LQ_HNbud_c17281/f1p11/3062 | i2_LQ_HNbud_c14376/f1p1/2692 |
| i2_LQ_HNbud_c8614/f1p3/2351 | i1_LQ_HNbud_c107095/f1p1/1896 | i3_LQ_HNbud_c3026/f1p1/3238 | i1_LQ_HNbud_c104148/f1p10/1691 |
| i2_LQ_HNbud_c37638/f1p3/2694 | i3_LQ_HNbud_c11999/f1p2/3376 | i2_LQ_HNbud_c20194/f1p1/2658 | i3_HQ_HNbud_c1687/f2p1/3794 |
| i1_HQ_HNbud_c171032/f3p3/1563 | i2_LQ_HNbud_c25605/f1p11/2374 | i2_LQ_HNbud_c46508/f1p0/2043 | i1_HQ_HNbud_c23869/f2p3/1240 |
| i1_LQ_HNbud_c256683/f1p0/1027 | i1_LQ_HNbud_c38904/f1p4/1447 | i1_HQ_HNbud_c72168/f2p2/1822 | i1_LQ_HNbud_c215721/f1p1/1058 |
| i1_LQ_HNbud_c91035/f1p1/1218 | i1_LQ_HNbud_c149865/f1p3/1073 | i2_LQ_HNbud_c41221/f1p35/2503 | i0_LQ_HNbud_c77439/f1p0/905 |
| i3_HQ_HNbud_c866/f5p0/3290 | i1_HQ_HNbud_c265689/f15p0/1288 | i0_LQ_HNbud_c72917/f1p1/737 | i1_LQ_HNbud_c270811/f1p2/1233 |
| i1_LQ_HNbud_c67489/f1p0/1639 | i1_LQ_HNbud_c151427/f1p1/1587 | i1_HQ_HNbud_c151058/f2p6/1154 | i1_LQ_HNbud_c214982/f1p1/1031 |
| i1_HQ_HNbud_c264055/f6p4/1124 | i2_LQ_HNbud_c37628/f1p6/2552 | i1_LQ_HNbud_c52992/f1p0/1372 | i2_LQ_HNbud_c22216/f1p19/2669 |
| i0_HQ_HNbud_c18596/f2p2/968 | i1_LQ_HNbud_c29939/f1p23/898 | i1_LQ_HNbud_c110684/f1p17/1495 | i1_LQ_HNbud_c23111/f3p11/1998 |
| i3_LQ_HNbud_c2457/f1p0/3669 | i2_LQ_HNbud_c5899/f1p4/2182 | i1_HQ_HNbud_c84067/f2p0/1535 | i3_LQ_HNbud_c16383/f1p0/3058 |
| i2_LQ_HNbud_c26465/f1p1/2345 | i2_HQ_HNbud_c65352/f7p1/2133 | i3_LQ_HNbud_c11169/f1p5/3216 | i0_HQ_HNbud_c97905/f17p0/839 |
| i1_LQ_HNbud_c88039/f1p9/1832 | i1_LQ_HNbud_c74990/f1p4/1541 | i0_LQ_HNbud_c21096/f1p2/954 | i0_HQ_HNbud_c1532/f10p0/661 |
| i1_LQ_HNbud_c201824/f1p1/1516 | i2_LQ_HNbud_c24273/f1p10/2134 | i1_LQ_HNbud_c73121/f1p2/1360 | i0_LQ_HNbud_c77407/f1p0/504 |
| i0_HQ_HNbud_c26961/f2p0/819 | i1_HQ_HNbud_c34831/f9p3/1588 | i1_LQ_HNbud_c73081/f1p4/1660 | i2_LQ_HNbud_c64074/f1p4/2069 |
| i1_LQ_HNbud_c170707/f1p1/1272 | i1_HQ_HNbud_c17865/f8p2/1634 | i5_LQ_HNbud_c2011/f1p0/5002 | i1_LQ_HNbud_c163378/f1p53/1349 |
| i2_HQ_HNbud_c1968/f12p0/2449 | i3_LQ_HNbud_c12160/f1p1/3667 | i2_LQ_HNbud_c38566/f1p0/2432 | i3_LQ_HNbud_c8224/f1p0/3773 |
| i1_LQ_HNbud_c72418/f1p1/1993 | i0_HQ_HNbud_c64813/f38p0/674 | i1_LQ_HNbud_c23752/f1p16/1561 | i2_HQ_HNbud_c1124/f22p5/2649 |
| i2_LQ_HNbud_c54070/f1p3/2097 | i2_LQ_HNbud_c11287/f1p13/2758 | i1_LQ_HNbud_c38367/f1p12/1662 | i2_LQ_HNbud_c62306/f1p1/2340 |
| i0_LQ_HNbud_c25594/f1p0/452 | i3_LQ_HNbud_c10344/f1p2/3415 | i2_HQ_HNbud_c11750/f3p7/2395 | i3_LQ_HNbud_c9478/f1p2/3542 |
| i0_LQ_HNbud_c45171/f1p0/559 | i1_LQ_HNbud_c93624/f1p7/1591 | i1_LQ_HNbud_c68399/f1p2/1432 | i3_HQ_HNbud_c16817/f6p0/3750 |
| i1_LQ_HNbud_c51995/f1p3/1681 | i1_LQ_HNbud_c66046/f1p0/1471 | i0_LQ_HNbud_c31148/f1p0/753 | i0_LQ_HNbud_c4651/f1p0/729 |
| i0_LQ_HNbud_c10679/f1p2/846 | i1_LQ_HNbud_c132992/f1p2/1797 | i0_HQ_HNbud_c2247/f8p0/568 | i1_LQ_HNbud_c11950/f1p3/1728 |
| i2_LQ_HNbud_c52904/f1p3/2645 | i0_LQ_HNbud_c71296/f1p25/954 | i2_LQ_HNbud_c57710/f1p12/2520 | i1_LQ_HNbud_c155803/f1p1/1571 |
| i2_HQ_HNbud_c3097/f2p10/2214 | i1_LQ_HNbud_c241612/f1p10/1577 | i1_LQ_HNbud_c187751/f1p11/1017 | i3_LQ_HNbud_c6884/f1p5/3405 |
| i2_LQ_HNbud_c1788/f2p5/2640 | i1_LQ_HNbud_c256431/f1p29/1527 | i3_LQ_HNbud_c12849/f1p1/3582 | i0_HQ_HNbud_c24916/f5p5/514 |
| i3_LQ_HNbud_c7557/f1p2/3595 | i5_LQ_HNbud_c688/f1p0/5442 | i2_LQ_HNbud_c13711/f2p0/2480 | i1_HQ_HNbud_c264960/f12p15/1489 |
| i2_HQ_HNbud_c70575/f13p7/2713 | i1_LQ_HNbud_c229242/f1p1/1615 | i3_LQ_HNbud_c13674/f1p14/3168 | i0_HQ_HNbud_c99564/f2p0/816 |
| i0_LQ_HNbud_c5154/f1p3/853 | i1_HQ_HNbud_c18085/f3p1/1542 | i1_LQ_HNbud_c128678/f1p0/1225 | i1_LQ_HNbud_c38380/f1p0/1931 |
| i1_LQ_HNbud_c174365/f1p3/1293 | i2_LQ_HNbud_c55117/f1p2/2078 | i4_LQ_HNbud_c1825/f1p4/4331 | i1_HQ_HNbud_c264833/f5p1/1770 |
| i1_LQ_HNbud_c103733/f2p4/1404 | i1_LQ_HNbud_c152660/f1p5/1539 | i2_LQ_HNbud_c10113/f1p4/2253 | i1_LQ_HNbud_c132880/f1p6/1420 |
| i0_LQ_HNbud_c7931/f1p14/538 | i1_HQ_HNbud_c33637/f3p3/1675 | i0_HQ_HNbud_c17162/f26p0/801 | i1_LQ_HNbud_c93453/f1p3/1143 |
| i0_LQ_HNbud_c40236/f1p18/926 | i1_HQ_HNbud_c265070/f15p9/1262 | i3_HQ_HNbud_c1509/f2p14/3272 | i1_HQ_HNbud_c168008/f20p9/1487 |
| i1_LQ_HNbud_c142033/f1p1/1055 | i1_HQ_HNbud_c183813/f2p3/1091 | i1_HQ_HNbud_c13276/f2p3/1699 | i1_LQ_HNbud_c26515/f2p0/1568 |
| i0_HQ_HNbud_c18447/f5p9/817 | i0_LQ_HNbud_c74832/f1p0/988 | i0_LQ_HNbud_c49222/f1p1/885 | i1_LQ_HNbud_c10654/f1p23/1280 |
| i2_HQ_HNbud_c13107/f3p0/2668 | i1_LQ_HNbud_c75648/f1p10/1698 | i0_LQ_HNbud_c50590/f1p2/744 | i1_LQ_HNbud_c112653/f1p2/1943 |
| i2_HQ_HNbud_c7425/f7p0/2856 | i0_LQ_HNbud_c74350/f1p0/856 | i2_LQ_HNbud_c8465/f1p2/2073 | i5_LQ_HNbud_c1406/f1p0/5205 |
| i5_LQ_HNbud_c1786/f1p6/5139 | i1_LQ_HNbud_c104177/f2p2/1276 | i1_LQ_HNbud_c176020/f1p4/1312 | i1_LQ_HNbud_c152465/f1p6/1915 |
| i2_LQ_HNbud_c51877/f1p0/2345 | i1_HQ_HNbud_c2389/f7p2/1229 | i1_HQ_HNbud_c84053/f3p1/1282 | i0_LQ_HNbud_c25399/f1p3/634 |
| i1_LQ_HNbud_c51289/f1p6/1695 | i2_HQ_HNbud_c18092/f2p4/2897 | i1_HQ_HNbud_c24360/f2p3/1364 | i1_HQ_HNbud_c13664/f2p1/1822 |
| i0_HQ_HNbud_c38077/f13p0/615 | i2_LQ_HNbud_c63140/f1p2/2027 | i1_HQ_HNbud_c104675/f2p39/1858 | i1_HQ_HNbud_c132399/f2p42/1160 |
| i3_LQ_HNbud_c13856/f1p17/3293 | i3_LQ_HNbud_c4550/f1p1/3819 | i2_LQ_HNbud_c22029/f1p1/2963 | i0_LQ_HNbud_c40388/f1p1/984 |
| i1_HQ_HNbud_c73917/f2p0/1452 | i0_LQ_HNbud_c40499/f1p13/790 | i1_HQ_HNbud_c49847/f2p1/1595 | i1_HQ_HNbud_c235330/f2p9/1491 |
| i1_LQ_HNbud_c199566/f1p33/1879 | i0_LQ_HNbud_c13854/f1p2/685 | i1_LQ_HNbud_c126808/f1p3/1273 | i2_LQ_HNbud_c10757/f1p0/2718 |
| i1_LQ_HNbud_c56523/f1p1/1694 | i1_LQ_HNbud_c127426/f1p1/1516 | i2_HQ_HNbud_c13065/f5p2/2613 | i1_LQ_HNbud_c170821/f1p6/1843 |
| i0_HQ_HNbud_c29028/f3p0/844 | i4_HQ_HNbud_c135/f2p0/4488 | i0_LQ_HNbud_c30094/f1p10/620 | i1_LQ_HNbud_c37492/f1p1/1659 |
| i2_LQ_HNbud_c39244/f1p0/2119 | i2_HQ_HNbud_c61363/f4p2/2053 | i1_LQ_HNbud_c57090/f1p2/1614 | i2_LQ_HNbud_c10111/f1p2/2595 |
| i1_LQ_HNbud_c223192/f1p3/1090 | i2_LQ_HNbud_c54297/f1p6/2164 | i1_LQ_HNbud_c132472/f1p3/1892 | i2_LQ_HNbud_c36049/f1p18/2079 |
| i2_LQ_HNbud_c54382/f1p3/2214 | i1_HQ_HNbud_c183836/f2p3/1029 | i4_LQ_HNbud_c1235/f1p0/4437 | i2_LQ_HNbud_c51092/f1p13/2266 |
| i4_LQ_HNbud_c3657/f1p7/4163 | i0_LQ_HNbud_c72468/f1p0/931 | i1_HQ_HNbud_c26689/f2p4/1388 | i2_LQ_HNbud_c35004/f1p7/2525 |
| i1_HQ_HNbud_c48461/f4p2/1472 | i2_HQ_HNbud_c47568/f5p0/2245 | i3_LQ_HNbud_c3802/f1p1/3574 | i2_HQ_HNbud_c65153/f5p2/2080 |
| i1_HQ_HNbud_c20323/f3p1/1346 | i1_LQ_HNbud_c13712/f1p5/1199 | i0_HQ_HNbud_c98570/f15p0/981 | i1_LQ_HNbud_c30491/f1p1/1034 |
| i1_LQ_HNbud_c252864/f1p1/1229 | i2_HQ_HNbud_c47761/f2p2/2097 | i2_LQ_HNbud_c12771/f1p2/2336 | i3_LQ_HNbud_c5085/f1p0/3502 |
| i1_LQ_HNbud_c204133/f1p2/1798 | i1_LQ_HNbud_c260634/f1p46/1085 | i3_LQ_HNbud_c15943/f1p1/3056 | i1_HQ_HNbud_c242974/f13p6/1561 |
| i2_LQ_HNbud_c57733/f1p1/2491 | i0_LQ_HNbud_c75113/f1p6/813 | i1_LQ_HNbud_c250879/f1p0/1308 | i2_LQ_HNbud_c53369/f1p11/2426 |
| i1_LQ_HNbud_c93482/f1p5/1311 | i2_HQ_HNbud_c5281/f2p0/2201 | i0_LQ_HNbud_c70584/f1p3/625 | i2_LQ_HNbud_c2702/f1p3/2096 |
| i1_LQ_HNbud_c13345/f1p1/1806 | i3_LQ_HNbud_c9469/f1p11/3124 | i2_LQ_HNbud_c30044/f1p0/2049 | i2_HQ_HNbud_c61390/f4p8/2052 |
| i1_LQ_HNbud_c170808/f1p1/1467 | i3_LQ_HNbud_c5688/f1p4/3260 | i1_LQ_HNbud_c69251/f1p0/1677 | i0_LQ_HNbud_c41336/f1p336/809 |
| i3_LQ_HNbud_c11474/f1p0/3088 | i1_LQ_HNbud_c129473/f1p2/1341 | i0_HQ_HNbud_c2396/f2p6/698 | i1_LQ_HNbud_c153815/f1p4/1499 |
| i2_LQ_HNbud_c55092/f1p9/2843 | i1_HQ_HNbud_c244123/f43p8/1116 | i1_LQ_HNbud_c196820/f1p1/1593 | i2_LQ_HNbud_c38611/f1p2/2104 |
| i1_LQ_HNbud_c56619/f1p10/1562 | i1_LQ_HNbud_c107994/f1p1/1408 | i2_LQ_HNbud_c6827/f1p25/2329 | i2_LQ_HNbud_c28196/f1p1/2636 |
| i1_LQ_HNbud_c103859/f1p10/1918 | i2_LQ_HNbud_c59422/f1p1/2589 | i1_LQ_HNbud_c205056/f1p7/1401 | i0_LQ_HNbud_c9773/f1p27/859 |
| i1_LQ_HNbud_c106192/f1p9/1428 | i1_LQ_HNbud_c66339/f1p7/1686 | i1_HQ_HNbud_c93107/f3p2/1941 | i1_LQ_HNbud_c222414/f1p3/1416 |
| i2_LQ_HNbud_c14435/f1p0/2980 | i1_HQ_HNbud_c18293/f8p6/1878 | i1_LQ_HNbud_c130274/f1p7/2075 | i0_LQ_HNbud_c3121/f1p10/988 |
| i1_HQ_HNbud_c255415/f4p1/1097 | i3_HQ_HNbud_c16990/f2p1/3050 | i2_LQ_HNbud_c41039/f1p1/2678 | i0_LQ_HNbud_c40149/f1p7/876 |
| i2_LQ_HNbud_c42646/f1p1/2303 | i2_LQ_HNbud_c55715/f1p3/2247 | i1_HQ_HNbud_c256090/f2p10/1057 | i1_LQ_HNbud_c222955/f1p1/1410 |
| i1_LQ_HNbud_c132116/f1p6/1194 | i1_LQ_HNbud_c113266/f1p4/1810 | i1_HQ_HNbud_c22199/f2p7/1339 | i2_LQ_HNbud_c57969/f1p2/2085 |
| i1_HQ_HNbud_c23412/f2p3/1814 | i1_LQ_HNbud_c280655/f1p2/1015 | i1_LQ_HNbud_c126948/f1p3/1175 | i1_HQ_HNbud_c55251/f3p4/1497 |
| i2_HQ_HNbud_c12641/f3p3/2256 | i0_HQ_HNbud_c7719/f2p2/841 | i1_LQ_HNbud_c107711/f1p20/1799 | i0_LQ_HNbud_c26130/f1p0/852 |
| i1_LQ_HNbud_c228631/f1p7/1590 | i1_LQ_HNbud_c165161/f1p24/1529 | i1_LQ_HNbud_c105208/f1p3/1603 | i1_HQ_HNbud_c2398/f23p4/1286 |
| i2_LQ_HNbud_c41279/f1p8/2688 | i0_LQ_HNbud_c10305/f2p10/966 | i1_LQ_HNbud_c108124/f1p3/1922 | i1_HQ_HNbud_c208395/f3p2/1081 |
| i2_LQ_HNbud_c37272/f1p12/2085 | i0_LQ_HNbud_c23368/f1p1/857 | i1_LQ_HNbud_c49213/f1p10/1794 | i1_LQ_HNbud_c163156/f1p11/1357 |
| i1_LQ_HNbud_c73079/f1p8/1581 | i1_LQ_HNbud_c68249/f1p3/1467 | i1_HQ_HNbud_c1353/f26p3/1438 | i2_LQ_HNbud_c28352/f1p0/2215 |
| i2_LQ_HNbud_c53814/f1p11/2086 | i0_LQ_HNbud_c44356/f1p0/782 | i1_LQ_HNbud_c51747/f1p2/1370 | i1_HQ_HNbud_c7903/f4p1/1833 |
| i1_HQ_HNbud_c192349/f3p2/1726 | i2_LQ_HNbud_c36843/f1p1/2955 | i1_HQ_HNbud_c42083/f2p0/1245 | i2_HQ_HNbud_c21132/f2p1/2055 |
| i1_HQ_HNbud_c148547/f2p0/1978 | i1_HQ_HNbud_c42284/f7p10/1860 | i1_LQ_HNbud_c75563/f1p0/1658 | i1_LQ_HNbud_c55762/f1p1/1608 |
| i1_HQ_HNbud_c87950/f2p1/1672 | i1_HQ_HNbud_c244641/f4p2/1644 | i2_LQ_HNbud_c50888/f1p1/2441 | i2_LQ_HNbud_c19541/f1p0/2241 |
| i1_LQ_HNbud_c183941/f2p4/1046 | i1_LQ_HNbud_c11182/f1p7/1644 | i1_LQ_HNbud_c210983/f1p2/1098 | i0_LQ_HNbud_c7875/f1p2/956 |
| i1_HQ_HNbud_c123175/f14p7/1253 | i1_LQ_HNbud_c172244/f1p14/1662 | i2_HQ_HNbud_c11081/f2p3/2597 | i1_LQ_HNbud_c203664/f1p0/1404 |
| i0_HQ_HNbud_c1820/f5p12/975 | i3_LQ_HNbud_c6858/f1p13/3452 | i1_HQ_HNbud_c36188/f3p4/1267 | i2_LQ_HNbud_c39707/f1p3/2318 |
| i0_HQ_HNbud_c1487/f9p0/769 | i1_LQ_HNbud_c149147/f1p0/1558 | i2_HQ_HNbud_c1429/f3p2/2633 | i1_LQ_HNbud_c73852/f1p2/1375 |
| i0_LQ_HNbud_c21619/f1p1/895 | i2_LQ_HNbud_c2350/f1p5/2827 | i1_HQ_HNbud_c265730/f16p2/1856 | i2_LQ_HNbud_c5365/f1p7/2888 |
| i1_HQ_HNbud_c14057/f6p6/1439 | i1_HQ_HNbud_c16124/f104p13/1315 | i1_HQ_HNbud_c68354/f3p8/1831 | i1_LQ_HNbud_c67935/f1p8/1960 |
| i1_LQ_HNbud_c132829/f1p1/1589 | i2_LQ_HNbud_c43805/f1p3/2394 | i1_LQ_HNbud_c37338/f1p1/1126 | i1_LQ_HNbud_c90397/f1p10/1618 |
| i0_LQ_HNbud_c49088/f1p1/962 | i4_LQ_HNbud_c2536/f1p1/4570 | i1_HQ_HNbud_c10480/f3p13/1376 | i1_LQ_HNbud_c29891/f1p0/1046 |
| i1_LQ_HNbud_c151123/f1p3/1669 | i1_LQ_HNbud_c116085/f1p4/1052 | i1_LQ_HNbud_c10922/f1p3/1336 | i2_HQ_HNbud_c70873/f5p6/2044 |
| i1_LQ_HNbud_c163861/f1p3/1359 | i2_LQ_HNbud_c26825/f1p9/2334 | i1_LQ_HNbud_c24599/f1p6/1320 | i1_LQ_HNbud_c132985/f1p4/1197 |
| i2_HQ_HNbud_c25591/f3p2/2250 | i2_LQ_HNbud_c5863/f1p1/2324 | i3_LQ_HNbud_c12694/f1p7/3281 | i0_HQ_HNbud_c16127/f3p0/887 |
| i0_LQ_HNbud_c10302/f2p1/487 | i0_HQ_HNbud_c18438/f4p3/749 | i0_LQ_HNbud_c24503/f1p0/955 | i1_LQ_HNbud_c117602/f1p10/1280 |
| i1_LQ_HNbud_c226474/f1p4/1239 | i1_LQ_HNbud_c99446/f1p225/1037 | i1_LQ_HNbud_c215783/f1p3/1064 | i1_HQ_HNbud_c34707/f2p7/1628 |
| i1_LQ_HNbud_c19526/f1p1/1787 | i1_HQ_HNbud_c86937/f2p4/1764 | i1_HQ_HNbud_c2822/f27p6/1553 | i2_LQ_HNbud_c63173/f1p2/2036 |
| i3_LQ_HNbud_c9675/f1p0/3754 | i1_LQ_HNbud_c25561/f1p3/1659 | i0_HQ_HNbud_c66531/f4p1/511 | i2_LQ_HNbud_c25345/f1p1/2458 |
| i0_HQ_HNbud_c98357/f3p4/967 | i0_LQ_HNbud_c5254/f1p0/675 | i1_LQ_HNbud_c21310/f1p4/1642 | i1_LQ_HNbud_c228084/f1p22/1389 |
| i1_LQ_HNbud_c140385/f1p1/1041 | i1_LQ_HNbud_c150017/f1p0/1414 | i1_HQ_HNbud_c143640/f4p2/1429 | i1_LQ_HNbud_c22858/f1p4/1285 |
| i1_HQ_HNbud_c267210/f2p10/1053 | i1_LQ_HNbud_c133601/f1p11/1545 | i1_LQ_HNbud_c224705/f1p1/1527 | i1_LQ_HNbud_c9606/f1p4/1157 |
| i1_HQ_HNbud_c130601/f2p5/1720 | i1_LQ_HNbud_c277956/f1p0/1008 | i0_HQ_HNbud_c12393/f2p0/670 | i1_LQ_HNbud_c10818/f1p0/1903 |
| i1_HQ_HNbud_c144463/f8p4/1506 | i1_LQ_HNbud_c150807/f1p23/1399 | i1_LQ_HNbud_c106817/f1p1/1786 | i0_HQ_HNbud_c50945/f2p0/706 |
| i2_LQ_HNbud_c28409/f1p5/2128 | i6_LQ_HNbud_c2786/f1p5/6367 | i2_HQ_HNbud_c70503/f4p0/2261 | i0_LQ_HNbud_c776/f14p0/767 |
| i1_LQ_HNbud_c108907/f1p20/1268 | i1_LQ_HNbud_c85495/f1p9/1948 | i0_LQ_HNbud_c3025/f1p5/972 | i1_LQ_HNbud_c140870/f1p3/1024 |
| i1_LQ_HNbud_c155187/f1p14/1982 | i1_HQ_HNbud_c103513/f3p0/1297 | i1_LQ_HNbud_c87730/f1p9/1239 | i1_LQ_HNbud_c224090/f1p1/1191 |
| i1_HQ_HNbud_c265046/f3p19/1873 | i1_LQ_HNbud_c205382/f1p8/1358 | i4_LQ_HNbud_c2474/f1p1/4126 | i0_LQ_HNbud_c27230/f1p0/947 |
| i2_HQ_HNbud_c65499/f4p3/2108 | i2_LQ_HNbud_c21845/f1p4/2694 | i2_LQ_HNbud_c25180/f1p3/3046 | i1_HQ_HNbud_c132959/f2p7/1775 |
| i1_LQ_HNbud_c37318/f1p5/1379 | i2_HQ_HNbud_c14556/f2p3/2582 | i2_HQ_HNbud_c26570/f2p3/2168 | i4_LQ_HNbud_c3663/f1p4/4714 |
| i0_LQ_HNbud_c80314/f1p0/695 | i4_LQ_HNbud_c1572/f1p0/4394 | i1_LQ_HNbud_c279249/f1p2/1006 | i2_LQ_HNbud_c34732/f1p2/1823 |
| i1_LQ_HNbud_c37735/f1p11/1524 | i1_LQ_HNbud_c4191/f1p1/1683 | i2_HQ_HNbud_c882/f17p10/2305 | i2_LQ_HNbud_c57400/f1p12/2226 |
| i0_HQ_HNbud_c44681/f2p0/806 | i1_LQ_HNbud_c129645/f1p1/1312 | i2_LQ_HNbud_c64186/f1p1/2066 | i1_LQ_HNbud_c112791/f1p1/1504 |
| i2_HQ_HNbud_c28894/f2p2/2739 | i1_LQ_HNbud_c152724/f1p0/1352 | i1_LQ_HNbud_c13474/f1p3/2008 | i1_LQ_HNbud_c21792/f1p0/1209 |
| i1_HQ_HNbud_c267254/f3p0/1822 | i2_LQ_HNbud_c13259/f1p2/2051 | i0_HQ_HNbud_c54747/f2p150/506 | i0_LQ_HNbud_c5618/f1p4/978 |
| i2_LQ_HNbud_c72527/f1p1/2611 | i1_LQ_HNbud_c126947/f1p2/1790 | i2_LQ_HNbud_c45281/f2p2/2057 | i1_LQ_HNbud_c116421/f1p8/1115 |
| i1_HQ_HNbud_c82756/f3p4/1130 | i1_LQ_HNbud_c109016/f1p4/1275 | i1_HQ_HNbud_c81407/f8p9/1723 | i3_LQ_HNbud_c17657/f1p0/3673 |
| i0_HQ_HNbud_c98244/f3p0/746 | i1_LQ_HNbud_c232054/f1p2/1719 | i1_LQ_HNbud_c177068/f1p7/1700 | i1_HQ_HNbud_c66955/f2p18/1131 |
| i2_HQ_HNbud_c32185/f6p4/2746 | i1_LQ_HNbud_c38893/f1p15/1823 | i3_LQ_HNbud_c9201/f1p2/3206 | i1_LQ_HNbud_c280003/f1p0/1003 |
| i2_LQ_HNbud_c11795/f1p0/2197 | i0_LQ_HNbud_c42476/f1p0/681 | i1_LQ_HNbud_c38923/f2p0/1282 | i2_HQ_HNbud_c34634/f2p4/2911 |
| i2_LQ_HNbud_c33953/f1p0/2456 | i1_HQ_HNbud_c2575/f8p0/1738 | i1_LQ_HNbud_c170377/f1p32/1820 | i1_LQ_HNbud_c22353/f1p1/1102 |
| i0_HQ_HNbud_c12728/f2p11/979 | i1_LQ_HNbud_c6893/f1p4/1679 | i2_LQ_HNbud_c14372/f1p3/2424 | i1_LQ_HNbud_c252886/f1p6/1645 |
| i1_HQ_HNbud_c166236/f16p8/1503 | i0_HQ_HNbud_c97769/f12p1/789 | i1_LQ_HNbud_c18895/f2p0/1808 | i1_LQ_HNbud_c45758/f1p0/1015 |
| i1_LQ_HNbud_c265119/f11p4/1145 | i1_LQ_HNbud_c22135/f1p3/1730 | i1_LQ_HNbud_c224805/f1p2/1098 | i0_HQ_HNbud_c39453/f5p11/914 |
| i0_LQ_HNbud_c49986/f1p5/939 | i1_HQ_HNbud_c39715/f3p1/1470 | i1_LQ_HNbud_c139921/f1p1/1033 | i1_LQ_HNbud_c54798/f1p1/1707 |
| i2_LQ_HNbud_c38142/f1p3/2097 | i0_HQ_HNbud_c9919/f2p1/833 | i1_HQ_HNbud_c264402/f4p2/1239 | i1_LQ_HNbud_c214082/f1p3/1609 |
| i1_LQ_HNbud_c88413/f1p3/1139 | i2_LQ_HNbud_c15133/f1p2/2048 | i3_HQ_HNbud_c1898/f2p1/3123 | i3_LQ_HNbud_c1774/f2p8/3137 |
| i0_LQ_HNbud_c31515/f1p0/945 | i1_HQ_HNbud_c234215/f4p5/1065 | i4_LQ_HNbud_c512/f1p6/4595 | i1_LQ_HNbud_c5649/f1p1/1550 |
| i1_LQ_HNbud_c224585/f1p0/1960 | i2_LQ_HNbud_c30197/f1p0/2016 | i1_HQ_HNbud_c129524/f2p7/1824 | i2_HQ_HNbud_c28829/f2p0/2560 |
| i1_HQ_HNbud_c13598/f7p1/1536 | i2_HQ_HNbud_c70754/f17p2/2407 | i1_LQ_HNbud_c261308/f1p0/1024 | i2_LQ_HNbud_c36039/f1p6/2936 |
| i0_HQ_HNbud_c23636/f2p4/557 | i1_LQ_HNbud_c200839/f1p3/1710 | i1_HQ_HNbud_c9861/f16p4/1386 | i1_LQ_HNbud_c10607/f1p0/1139 |
| i2_LQ_HNbud_c39930/f1p0/2125 | i2_LQ_HNbud_c52566/f1p0/2518 | i1_LQ_HNbud_c40717/f1p1/1586 | i1_LQ_HNbud_c152936/f1p0/1197 |
| i1_LQ_HNbud_c3473/f1p9/1937 | i0_LQ_HNbud_c12643/f1p15/946 | i1_LQ_HNbud_c176159/f1p14/1628 | i1_LQ_HNbud_c35503/f1p1/1651 |
| i1_LQ_HNbud_c35042/f1p10/1665 | i0_HQ_HNbud_c97811/f6p0/967 | i1_HQ_HNbud_c145216/f43p1/1732 | i1_HQ_HNbud_c243715/f3p0/1112 |
| i1_LQ_HNbud_c107025/f1p0/1573 | i1_LQ_HNbud_c70477/f1p4/1145 | i1_LQ_HNbud_c7052/f2p0/1666 | i2_LQ_HNbud_c22522/f1p0/2218 |
| i1_LQ_HNbud_c107002/f1p0/1468 | i2_LQ_HNbud_c30795/f1p0/2022 | i1_HQ_HNbud_c151229/f2p10/1741 | i1_LQ_HNbud_c4279/f1p0/1345 |
| i1_LQ_HNbud_c249918/f1p7/1202 | i0_LQ_HNbud_c71775/f1p0/784 | i2_LQ_HNbud_c3587/f1p11/3008 | i1_LQ_HNbud_c171340/f1p6/1910 |
| i3_LQ_HNbud_c1688/f2p2/3281 | i2_LQ_HNbud_c41760/f1p2/2383 | i1_LQ_HNbud_c107023/f1p3/1443 | i2_LQ_HNbud_c10246/f1p3/2146 |
| i1_LQ_HNbud_c119973/f1p0/1019 | i3_LQ_HNbud_c1731/f2p0/3825 | i2_HQ_HNbud_c61698/f2p0/2088 | i1_LQ_HNbud_c205431/f1p0/1372 |
| i1_LQ_HNbud_c73884/f1p1/1356 | i0_LQ_HNbud_c4675/f1p0/867 | i0_HQ_HNbud_c99510/f3p0/943 | i0_LQ_HNbud_c12990/f1p0/667 |
| i2_HQ_HNbud_c47678/f5p1/2425 | i0_LQ_HNbud_c20203/f1p0/639 | i1_LQ_HNbud_c24231/f2p4/1836 | i1_LQ_HNbud_c93667/f1p0/1268 |
| i2_LQ_HNbud_c31347/f1p3/2140 | i1_LQ_HNbud_c44218/f1p0/1086 | i1_HQ_HNbud_c144857/f35p17/1386 | i0_LQ_HNbud_c29301/f1p0/670 |
| i1_LQ_HNbud_c48837/f1p1/1816 | i2_LQ_HNbud_c63673/f1p0/2057 | i1_HQ_HNbud_c89056/f4p8/1759 | i1_LQ_HNbud_c129690/f1p16/1470 |
| i1_LQ_HNbud_c68792/f1p10/1601 | i1_LQ_HNbud_c155797/f1p9/1809 | i0_LQ_HNbud_c110278/f1p3/749 | i0_LQ_HNbud_c27618/f1p6/911 |
| i0_HQ_HNbud_c11261/f2p16/487 | i2_LQ_HNbud_c23873/f1p3/2890 | i1_LQ_HNbud_c38900/f1p15/1538 | i0_LQ_HNbud_c5177/f1p0/957 |
| i1_LQ_HNbud_c174576/f1p2/1485 | i2_LQ_HNbud_c7391/f1p0/2382 | i0_HQ_HNbud_c4998/f2p1/963 | i2_LQ_HNbud_c37364/f1p10/2455 |
| i2_HQ_HNbud_c20858/f2p2/3024 | i2_LQ_HNbud_c6720/f1p7/2563 | i1_LQ_HNbud_c128635/f1p1/1686 | i1_LQ_HNbud_c17893/f1p18/1916 |
| i1_LQ_HNbud_c224507/f1p1/1124 | i3_LQ_HNbud_c11600/f1p0/3687 | i1_LQ_HNbud_c34692/f1p3/1561 | i2_HQ_HNbud_c48028/f5p4/2104 |
| i1_LQ_HNbud_c68150/f1p7/1761 | i0_HQ_HNbud_c22820/f3p2/485 | i1_LQ_HNbud_c198082/f1p13/1542 | i1_LQ_HNbud_c90309/f1p19/1473 |
| i2_LQ_HNbud_c57074/f1p0/2099 | i0_LQ_HNbud_c24909/f1p0/876 | i1_LQ_HNbud_c41134/f1p12/1510 | i1_LQ_HNbud_c175139/f1p5/1338 |
| i2_LQ_HNbud_c2848/f1p56/2157 | i1_LQ_HNbud_c61965/f1p0/1487 | i0_LQ_HNbud_c25316/f1p5/741 | i0_HQ_HNbud_c8555/f7p0/776 |
| i0_HQ_HNbud_c818/f24p0/878 | i1_LQ_HNbud_c52574/f3p0/1582 | i1_LQ_HNbud_c280324/f1p0/1403 | i1_LQ_HNbud_c105186/f1p13/1905 |
| i1_LQ_HNbud_c105214/f1p5/1103 | i2_HQ_HNbud_c4062/f9p2/2207 | i0_LQ_HNbud_c55297/f1p14/760 | i1_LQ_HNbud_c133964/f1p7/1559 |
| i1_LQ_HNbud_c155572/f1p1/1852 | i0_LQ_HNbud_c41681/f2p4/775 | i1_HQ_HNbud_c159217/f3p1/1248 | i1_LQ_HNbud_c34047/f1p0/1535 |
| i0_LQ_HNbud_c85885/f2p0/957 | i2_HQ_HNbud_c68353/f2p0/2078 | i1_HQ_HNbud_c124254/f2p7/1277 | i2_LQ_HNbud_c54552/f1p5/1993 |
| i2_LQ_HNbud_c13133/f1p2/2546 | i1_HQ_HNbud_c2420/f6p0/1432 | i0_LQ_HNbud_c22044/f1p1/959 | i2_LQ_HNbud_c42991/f1p0/2356 |
| i2_LQ_HNbud_c73608/f1p0/2018 | i1_LQ_HNbud_c180258/f1p1/1392 | i1_LQ_HNbud_c249543/f1p0/1132 | i1_HQ_HNbud_c263253/f18p1/1111 |
| i1_LQ_HNbud_c139361/f1p2/1583 | i0_LQ_HNbud_c38733/f1p0/315 | i0_HQ_HNbud_c98430/f5p0/934 | i0_LQ_HNbud_c66829/f2p0/888 |
| i0_LQ_HNbud_c3759/f2p2/672 | i1_LQ_HNbud_c268627/f1p9/1914 | i1_LQ_HNbud_c3622/f1p0/1491 | i0_HQ_HNbud_c98090/f32p0/732 |
| i1_LQ_HNbud_c6591/f1p10/1423 | i1_HQ_HNbud_c67954/f2p2/1721 | i1_LQ_HNbud_c99987/f1p16/1651 | i1_LQ_HNbud_c131022/f1p2/1117 |
| i0_HQ_HNbud_c39478/f3p7/787 | i0_LQ_HNbud_c29016/f1p0/408 | i0_LQ_HNbud_c49974/f1p0/417 | i0_HQ_HNbud_c64768/f3p2/836 |
| i3_LQ_HNbud_c9757/f1p5/3117 | i1_HQ_HNbud_c150727/f2p5/2004 | i1_LQ_HNbud_c149028/f1p1/1943 | i0_LQ_HNbud_c55218/f1p8/735 |
| i1_LQ_HNbud_c139628/f1p1/1057 | i0_LQ_HNbud_c74196/f1p4/681 | i2_HQ_HNbud_c1916/f4p10/2258 | i1_HQ_HNbud_c170317/f2p3/1342 |
| i1_LQ_HNbud_c26538/f1p0/1417 | i1_LQ_HNbud_c149224/f1p1/1626 | i1_LQ_HNbud_c150466/f1p11/1210 | i6_LQ_HNbud_c2059/f1p0/6637 |
| i1_LQ_HNbud_c156532/f1p0/1266 | i1_LQ_HNbud_c55133/f1p16/1410 | i2_LQ_HNbud_c5248/f1p9/2233 | i1_LQ_HNbud_c278588/f1p0/1373 |
| i0_LQ_HNbud_c13714/f1p0/800 | i1_LQ_HNbud_c11478/f1p0/1380 | i0_LQ_HNbud_c82202/f1p5/812 | i0_HQ_HNbud_c2377/f6p0/864 |
| i1_LQ_HNbud_c166921/f2p0/1563 | i1_LQ_HNbud_c151065/f1p10/1254 | i0_LQ_HNbud_c75709/f2p5/788 | i0_HQ_HNbud_c2958/f2p6/652 |
| i1_HQ_HNbud_c272034/f3p10/1126 | i1_LQ_HNbud_c60600/f1p0/1048 | i2_LQ_HNbud_c20169/f1p9/2185 | i1_LQ_HNbud_c49140/f3p5/1314 |
| i1_LQ_HNbud_c8162/f1p1/1138 | i1_LQ_HNbud_c42000/f1p25/1482 | i2_LQ_HNbud_c18412/f1p8/3004 | i0_LQ_HNbud_c72184/f1p10/804 |
| i1_LQ_HNbud_c180632/f1p1/1513 | i0_HQ_HNbud_c9787/f2p4/869 | i0_HQ_HNbud_c37987/f27p2/714 | i1_LQ_HNbud_c277970/f1p15/1046 |
| i0_LQ_HNbud_c81947/f1p0/671 | i1_LQ_HNbud_c106223/f1p15/1565 | i1_LQ_HNbud_c63865/f1p26/1870 | i1_LQ_HNbud_c210474/f1p7/1106 |
| i1_LQ_HNbud_c44636/f1p1/1020 | i0_HQ_HNbud_c9856/f5p16/890 | i1_LQ_HNbud_c74179/f1p73/1594 | i0_LQ_HNbud_c109414/f1p10/695 |
| i0_LQ_HNbud_c31723/f1p0/862 | i1_HQ_HNbud_c56955/f4p2/1919 | i1_LQ_HNbud_c171870/f1p1/1396 | i0_LQ_HNbud_c42964/f1p0/955 |
| i1_LQ_HNbud_c188736/f1p0/1071 | i2_LQ_HNbud_c41566/f1p33/2392 | i1_LQ_HNbud_c162516/f1p0/1055 | i2_LQ_HNbud_c11686/f1p0/2721 |
| i1_LQ_HNbud_c171729/f1p3/1486 | i1_LQ_HNbud_c141100/f1p3/1147 | i1_LQ_HNbud_c68983/f3p4/1755 | i1_LQ_HNbud_c92557/f1p10/1702 |
| i0_LQ_HNbud_c81259/f1p5/669 | i1_LQ_HNbud_c203637/f1p0/1118 | i1_LQ_HNbud_c52169/f1p0/1257 | i1_HQ_HNbud_c87373/f4p10/1375 |
| i1_LQ_HNbud_c85861/f1p0/1204 | i1_HQ_HNbud_c36851/f5p3/1541 | i0_HQ_HNbud_c25827/f2p6/851 | i1_LQ_HNbud_c109501/f1p16/1545 |
| i0_HQ_HNbud_c23854/f3p7/712 | i0_LQ_HNbud_c67287/f1p3/696 | i2_HQ_HNbud_c7802/f3p0/2218 | i2_HQ_HNbud_c64702/f23p0/2046 |
| i0_LQ_HNbud_c85778/f1p8/714 | i0_HQ_HNbud_c26717/f2p0/719 | i2_LQ_HNbud_c16013/f1p0/2021 | i1_LQ_HNbud_c42016/f1p6/1885 |
| i1_LQ_HNbud_c34873/f1p2/1552 | i2_HQ_HNbud_c61373/f3p4/2045 | i0_LQ_HNbud_c98551/f6p0/558 | i4_LQ_HNbud_c395/f1p0/5032 |
| i1_LQ_HNbud_c50138/f1p6/1448 | i1_LQ_HNbud_c136032/f4p0/1081 | i0_LQ_HNbud_c29510/f1p0/995 | i1_LQ_HNbud_c250560/f1p7/1849 |
| i1_LQ_HNbud_c176967/f1p3/1971 | i1_LQ_HNbud_c7716/f1p5/1590 | i2_LQ_HNbud_c73476/f1p1/2013 | i0_LQ_HNbud_c6654/f1p0/712 |
| i1_LQ_HNbud_c106924/f1p1/1937 | i3_LQ_HNbud_c12351/f1p0/3317 | i1_LQ_HNbud_c56846/f1p0/1135 | i1_LQ_HNbud_c143010/f1p1/1587 |
| i1_LQ_HNbud_c206315/f2p11/1472 | i1_HQ_HNbud_c109792/f3p0/1588 | i1_HQ_HNbud_c264990/f3p9/1892 | i1_LQ_HNbud_c37337/f1p6/1333 |
| i2_LQ_HNbud_c60339/f1p0/2739 | i2_LQ_HNbud_c59627/f1p2/2578 | i1_LQ_HNbud_c72294/f1p9/1684 | i0_HQ_HNbud_c38308/f10p4/862 |
| i1_HQ_HNbud_c11896/f9p12/1313 | i0_HQ_HNbud_c30300/f2p3/810 | i1_LQ_HNbud_c18234/f1p43/1763 | i1_LQ_HNbud_c176806/f1p0/1806 |
| i1_LQ_HNbud_c105700/f1p2/1293 | i1_LQ_HNbud_c251048/f1p2/1945 | i0_HQ_HNbud_c714/f13p0/823 | i0_LQ_HNbud_c26602/f1p1/891 |
| i0_LQ_HNbud_c55304/f1p4/676 | i1_LQ_HNbud_c231904/f1p0/1102 | i1_LQ_HNbud_c196213/f1p24/1171 | i1_LQ_HNbud_c33573/f1p9/1496 |
| i2_HQ_HNbud_c18176/f3p5/2845 | i0_LQ_HNbud_c41277/f1p0/927 | i1_LQ_HNbud_c10732/f1p16/1341 | i1_LQ_HNbud_c130266/f1p4/1578 |
| i1_LQ_HNbud_c151267/f1p1/1616 | i1_LQ_HNbud_c154806/f1p1/1206 | i2_LQ_HNbud_c50454/f1p0/2224 | i3_LQ_HNbud_c4176/f1p3/3180 |
| i1_LQ_HNbud_c40715/f1p0/1271 | i0_HQ_HNbud_c7838/f9p1/668 | i0_LQ_HNbud_c78456/f1p0/745 | i3_LQ_HNbud_c5379/f1p4/3523 |
| i1_LQ_HNbud_c91521/f1p3/1246 | i2_HQ_HNbud_c10866/f3p5/2786 | i1_LQ_HNbud_c105240/f1p1/1186 | i1_LQ_HNbud_c53359/f1p4/1265 |
| i0_LQ_HNbud_c51125/f1p3/846 | i1_HQ_HNbud_c47236/f3p2/1696 | i2_LQ_HNbud_c54662/f1p0/2203 | i1_HQ_HNbud_c36266/f15p1/1568 |
| i1_HQ_HNbud_c193495/f17p3/1402 | i1_LQ_HNbud_c108697/f1p8/1515 | i1_HQ_HNbud_c66531/f2p0/1430 | i1_HQ_HNbud_c244085/f11p1/1559 |
| i0_HQ_HNbud_c39614/f2p2/511 | i2_LQ_HNbud_c4516/f1p0/2150 | i2_LQ_HNbud_c6167/f1p4/2833 | i2_LQ_HNbud_c10759/f1p2/2821 |
| i0_LQ_HNbud_c70302/f1p11/972 | i2_LQ_HNbud_c30974/f1p2/2034 | i0_LQ_HNbud_c2295/f1p9/697 | i2_LQ_HNbud_c3807/f1p13/2450 |
| i0_HQ_HNbud_c1448/f8p0/734 | i0_LQ_HNbud_c45041/f1p0/742 | i1_HQ_HNbud_c7516/f5p1/1519 | i1_LQ_HNbud_c69063/f1p7/1803 |
| i1_LQ_HNbud_c155863/f1p5/1043 | i3_LQ_HNbud_c15986/f1p0/3038 | i1_HQ_HNbud_c2020/f6p7/1447 | i0_LQ_HNbud_c84552/f1p0/862 |
| i1_LQ_HNbud_c266110/f1p2/1584 | i2_LQ_HNbud_c58737/f1p0/2747 | i1_LQ_HNbud_c9939/f1p2/1675 | i1_LQ_HNbud_c199667/f1p0/1192 |
| i1_HQ_HNbud_c12256/f2p1/1719 | i1_LQ_HNbud_c67101/f1p2/1921 | i0_LQ_HNbud_c22763/f2p0/591 | i2_LQ_HNbud_c33834/f1p2/2746 |
| i1_LQ_HNbud_c151617/f1p1/1503 | i1_LQ_HNbud_c272894/f1p6/1550 | i2_LQ_HNbud_c23994/f1p4/2838 | i1_HQ_HNbud_c1932/f9p8/1181 |
| i0_HQ_HNbud_c2291/f11p5/434 | i1_LQ_HNbud_c229163/f1p12/1456 | i0_LQ_HNbud_c79621/f1p2/890 | i1_LQ_HNbud_c49203/f1p0/1933 |
| i1_HQ_HNbud_c5124/f3p14/1144 | i2_LQ_HNbud_c58068/f1p4/2845 | i1_HQ_HNbud_c26105/f3p3/1526 | i1_LQ_HNbud_c4680/f12p3/1666 |
| i0_HQ_HNbud_c3596/f2p8/416 | i1_LQ_HNbud_c180862/f1p6/1388 | i2_LQ_HNbud_c5216/f1p1/2902 | i1_LQ_HNbud_c25944/f1p0/1227 |
| i2_LQ_HNbud_c43087/f1p0/2257 | i2_HQ_HNbud_c3208/f2p20/2217 | i2_HQ_HNbud_c17966/f2p8/2737 | i2_LQ_HNbud_c65169/f4p1/2058 |
| i2_HQ_HNbud_c2165/f4p3/2725 | i2_HQ_HNbud_c61417/f4p2/2050 | i2_HQ_HNbud_c3696/f2p2/2758 | i2_LQ_HNbud_c38165/f1p3/2081 |
| i1_HQ_HNbud_c49663/f6p1/1216 | i1_HQ_HNbud_c190903/f3p2/1273 | i1_LQ_HNbud_c201751/f1p23/1972 | i1_LQ_HNbud_c227971/f1p2/1593 |
| i0_LQ_HNbud_c3699/f1p1/524 | i2_LQ_HNbud_c55562/f1p1/2885 | i1_LQ_HNbud_c109747/f1p1/1892 | i0_LQ_HNbud_c12466/f1p0/718 |
| i1_LQ_HNbud_c107322/f1p24/1437 | i1_LQ_HNbud_c56970/f4p0/1492 | i1_HQ_HNbud_c7433/f3p1/1415 | i1_LQ_HNbud_c81741/f2p11/1077 |
| i1_LQ_HNbud_c253364/f1p4/1657 | i1_LQ_HNbud_c248430/f1p2/1397 | i0_LQ_HNbud_c29904/f1p5/568 | i1_LQ_HNbud_c74184/f1p2/1282 |
| i1_LQ_HNbud_c91368/f1p1/1547 | i0_LQ_HNbud_c24136/f1p0/874 | i2_LQ_HNbud_c52913/f1p2/2454 | i0_LQ_HNbud_c10892/f1p0/792 |
| i1_HQ_HNbud_c12801/f2p0/1708 | i2_HQ_HNbud_c70798/f6p3/2376 | i1_LQ_HNbud_c156337/f1p1/1323 | i1_LQ_HNbud_c266362/f1p11/1500 |
| i1_LQ_HNbud_c91992/f1p1/1333 | i1_LQ_HNbud_c62386/f1p3/1054 | i1_LQ_HNbud_c148347/f1p10/1501 | i2_LQ_HNbud_c28585/f1p1/2203 |
| i1_LQ_HNbud_c200814/f1p5/1292 | i0_LQ_HNbud_c67647/f1p14/544 | i2_LQ_HNbud_c4797/f1p10/2363 | i3_LQ_HNbud_c12861/f1p3/3395 |
| i1_HQ_HNbud_c14329/f17p3/1813 | i1_LQ_HNbud_c110252/f4p6/1234 | i1_LQ_HNbud_c252034/f1p1/1293 | i0_LQ_HNbud_c83628/f1p0/760 |
| i1_LQ_HNbud_c55898/f1p0/1637 | i3_LQ_HNbud_c10068/f1p274/3700 | i0_LQ_HNbud_c40366/f3p7/923 | i1_HQ_HNbud_c52460/f6p9/1688 |
| i1_HQ_HNbud_c265047/f51p11/1603 | i1_HQ_HNbud_c148648/f2p2/1593 | i2_LQ_HNbud_c51878/f1p0/2111 | i1_LQ_HNbud_c18302/f2p1/1429 |
| i1_LQ_HNbud_c159089/f1p1/1543 | i1_LQ_HNbud_c68991/f2p8/1225 | i1_LQ_HNbud_c20595/f1p7/1763 | i0_LQ_HNbud_c83596/f1p0/624 |
| i0_LQ_HNbud_c25693/f1p8/787 | i2_LQ_HNbud_c4464/f1p11/2267 | i1_LQ_HNbud_c251999/f1p12/1473 | i0_LQ_HNbud_c77165/f1p0/787 |
| i6_LQ_HNbud_c778/f1p0/6812 | i0_LQ_HNbud_c11942/f2p1/603 | i1_HQ_HNbud_c1876/f12p6/1691 | i2_LQ_HNbud_c55098/f1p2/2720 |
| i0_LQ_HNbud_c22945/f1p7/847 | i2_HQ_HNbud_c1042/f14p14/2586 | i2_HQ_HNbud_c12776/f2p0/2629 | i3_LQ_HNbud_c1173/f1p0/3896 |
| i1_HQ_HNbud_c41830/f4p10/1573 | i1_HQ_HNbud_c264326/f16p7/1304 | i2_LQ_HNbud_c12877/f1p9/2331 | i1_HQ_HNbud_c225397/f2p1/1802 |
| i0_HQ_HNbud_c11650/f3p0/742 | i3_LQ_HNbud_c11461/f1p1/3820 | i0_LQ_HNbud_c10210/f1p4/588 | i2_LQ_HNbud_c44255/f1p1/2874 |
| i1_LQ_HNbud_c91119/f1p1/1141 | i0_LQ_HNbud_c43086/f1p0/675 | i1_LQ_HNbud_c205710/f1p9/1645 | i2_HQ_HNbud_c44129/f2p4/2443 |
| i0_HQ_HNbud_c1683/f7p3/852 | i0_LQ_HNbud_c30486/f1p3/535 | i1_LQ_HNbud_c106492/f1p1/1301 | i1_LQ_HNbud_c180879/f1p0/1099 |
| i2_LQ_HNbud_c65094/f3p2/2070 | i0_LQ_HNbud_c39999/f1p0/786 | i3_LQ_HNbud_c10713/f1p0/3454 | i1_LQ_HNbud_c96299/f1p4/1490 |
| i0_HQ_HNbud_c66935/f2p5/869 | i1_LQ_HNbud_c197644/f1p1/1556 | i1_HQ_HNbud_c65642/f4p2/1385 | i1_LQ_HNbud_c198388/f1p11/1556 |
| i1_LQ_HNbud_c201752/f1p2/1690 | i1_HQ_HNbud_c222277/f2p14/1656 | i4_HQ_HNbud_c73/f5p0/4663 | i2_HQ_HNbud_c7939/f2p5/2088 |
| i0_LQ_HNbud_c8085/f2p10/1023 | i2_LQ_HNbud_c36239/f1p15/1930 | i1_HQ_HNbud_c2074/f11p3/1975 | i0_HQ_HNbud_c18694/f3p1/860 |
| i1_HQ_HNbud_c102410/f4p0/1195 | i0_LQ_HNbud_c52444/f1p1/788 | i2_HQ_HNbud_c71573/f2p10/2982 | i1_LQ_HNbud_c13279/f1p4/1515 |
| i1_LQ_HNbud_c225362/f1p2/1576 | i1_HQ_HNbud_c218348/f13p6/1127 | i1_LQ_HNbud_c73760/f1p0/1123 | i1_LQ_HNbud_c106093/f1p3/1784 |
| i1_LQ_HNbud_c166326/f16p7/1331 | i1_LQ_HNbud_c173662/f1p1/1662 | i0_LQ_HNbud_c84325/f1p0/741 | i1_LQ_HNbud_c87499/f1p2/1368 |
| i1_HQ_HNbud_c263690/f4p0/1393 | i1_HQ_HNbud_c166463/f5p5/1472 | i0_LQ_HNbud_c99897/f1p0/583 | i1_LQ_HNbud_c195868/f1p0/1441 |
| i1_LQ_HNbud_c214363/f1p4/1012 | i2_LQ_HNbud_c53666/f1p2/2779 | i1_HQ_HNbud_c19190/f2p13/1820 | i1_LQ_HNbud_c36001/f2p2/1652 |
| i1_LQ_HNbud_c228130/f1p1/1313 | i1_HQ_HNbud_c84127/f2p1/1575 | i1_HQ_HNbud_c267512/f2p5/1060 | i3_LQ_HNbud_c14731/f1p4/3117 |
| i2_LQ_HNbud_c27323/f1p3/2372 | i1_LQ_HNbud_c232902/f1p5/1466 | i2_HQ_HNbud_c27607/f5p14/2285 | i1_LQ_HNbud_c75768/f1p2/1323 |
| i2_HQ_HNbud_c2695/f3p4/2355 | i2_LQ_HNbud_c1569/f5p13/2465 | i2_LQ_HNbud_c9836/f1p2/2342 | i1_LQ_HNbud_c150415/f1p2/1731 |
| i1_LQ_HNbud_c196206/f1p2/1677 | i1_HQ_HNbud_c125595/f2p1/1509 | i1_LQ_HNbud_c24387/f1p1/1295 | i1_LQ_HNbud_c224098/f1p1/1248 |
| i1_LQ_HNbud_c9968/f1p8/1302 | i1_LQ_HNbud_c75784/f1p18/2187 | i1_LQ_HNbud_c6243/f1p10/1793 | i1_LQ_HNbud_c34536/f1p2/1445 |
| i1_HQ_HNbud_c192445/f11p10/1960 | i1_HQ_HNbud_c167801/f25p17/1644 | i2_LQ_HNbud_c38865/f1p5/2329 | i2_LQ_HNbud_c57922/f1p2/3077 |
| i1_LQ_HNbud_c231204/f1p267/2069 | i6_LQ_HNbud_c1369/f1p0/6472 | i2_LQ_HNbud_c14374/f2p3/2347 | i1_LQ_HNbud_c201097/f1p1/1275 |
| i1_LQ_HNbud_c96266/f1p2/1494 | i1_LQ_HNbud_c173526/f1p3/1610 | i2_LQ_HNbud_c56506/f1p1/2228 | i2_LQ_HNbud_c53689/f1p2/2065 |
| i1_LQ_HNbud_c90183/f1p1/1463 | i2_LQ_HNbud_c70902/f3p8/2034 | i1_LQ_HNbud_c250472/f1p12/1555 | i1_LQ_HNbud_c200157/f1p0/1249 |
| i1_LQ_HNbud_c106489/f1p5/1168 | i0_LQ_HNbud_c54936/f1p10/811 | i1_LQ_HNbud_c188420/f1p1/1023 | i1_LQ_HNbud_c34561/f1p4/1472 |
| i2_LQ_HNbud_c73800/f1p0/2009 | i4_LQ_HNbud_c1004/f1p0/4178 | i1_LQ_HNbud_c102203/f1p9/1609 | i2_LQ_HNbud_c15293/f1p5/1958 |
| i1_HQ_HNbud_c144206/f3p0/1120 | i0_HQ_HNbud_c12829/f2p9/752 | i1_LQ_HNbud_c7272/f1p1/1653 | i0_HQ_HNbud_c98610/f6p0/681 |
| i1_HQ_HNbud_c89053/f2p0/1287 | i1_LQ_HNbud_c249882/f1p3/1958 | i1_LQ_HNbud_c202417/f1p0/1315 | i6_LQ_HNbud_c2027/f1p19/6460 |
| i1_LQ_HNbud_c223670/f1p0/1735 | i0_HQ_HNbud_c39665/f2p0/810 | i2_LQ_HNbud_c30428/f1p1/2044 | i0_HQ_HNbud_c3001/f4p0/684 |
| i1_LQ_HNbud_c54749/f1p2/1638 | i1_LQ_HNbud_c253662/f1p2/1591 | i1_LQ_HNbud_c55802/f1p4/1561 | i0_LQ_HNbud_c6647/f1p0/966 |
| i1_LQ_HNbud_c149595/f1p18/1887 | i1_LQ_HNbud_c87156/f1p1/1192 | i1_LQ_HNbud_c18441/f1p1/1298 | i0_LQ_HNbud_c70018/f1p0/865 |
| i1_LQ_HNbud_c277978/f1p1/1004 | i1_HQ_HNbud_c195038/f2p14/1637 | i0_LQ_HNbud_c41742/f1p0/750 | i1_LQ_HNbud_c130377/f1p8/1670 |
| i0_LQ_HNbud_c69441/f1p8/614 | i0_LQ_HNbud_c78402/f1p0/725 | i1_HQ_HNbud_c265393/f15p0/1084 | i5_LQ_HNbud_c359/f1p0/5631 |
| i0_LQ_HNbud_c8079/f1p0/778 | i1_LQ_HNbud_c93627/f1p2/1506 | i2_LQ_HNbud_c62831/f1p0/2020 | i1_LQ_HNbud_c181000/f1p0/1347 |
| i1_LQ_HNbud_c201645/f1p0/1179 | i1_HQ_HNbud_c203488/f2p7/1581 | i2_HQ_HNbud_c36650/f2p0/2844 | i1_HQ_HNbud_c6060/f2p7/1145 |
| i1_LQ_HNbud_c265050/f2p4/1150 | i1_LQ_HNbud_c78589/f1p2/1068 | i0_HQ_HNbud_c37999/f17p1/758 | i1_LQ_HNbud_c229472/f1p2/1489 |
| i1_LQ_HNbud_c53469/f1p1/1654 | i1_LQ_HNbud_c12235/f2p2/1626 | i1_LQ_HNbud_c151002/f1p2/1468 | i1_LQ_HNbud_c229675/f1p11/1503 |
| i2_LQ_HNbud_c39020/f1p0/2666 | i2_LQ_HNbud_c34393/f1p0/2164 | i2_LQ_HNbud_c24976/f1p4/2806 | i1_HQ_HNbud_c192062/f13p1/1310 |
| i1_LQ_HNbud_c125548/f1p1/1967 | i0_HQ_HNbud_c1158/f11p0/996 | i1_LQ_HNbud_c40941/f1p1/1128 | i0_LQ_HNbud_c73860/f1p1/943 |
| i0_LQ_HNbud_c107710/f1p8/848 | i1_LQ_HNbud_c231676/f1p0/1118 | i0_LQ_HNbud_c45581/f1p0/806 | i1_LQ_HNbud_c99813/f1p2/1040 |
| i0_LQ_HNbud_c17292/f1p4/717 | i1_LQ_HNbud_c21432/f1p1/1470 | i1_LQ_HNbud_c37800/f1p1/1367 | i0_HQ_HNbud_c65123/f37p0/951 |
| i1_LQ_HNbud_c252778/f1p4/1983 | i1_LQ_HNbud_c10324/f1p17/1064 | i3_LQ_HNbud_c9180/f1p17/3937 | i1_LQ_HNbud_c67455/f1p0/1650 |
| i0_HQ_HNbud_c1438/f8p0/790 | i1_HQ_HNbud_c206047/f2p2/1717 | i0_HQ_HNbud_c18024/f4p0/569 | i1_LQ_HNbud_c49800/f1p6/1532 |
| i1_HQ_HNbud_c2474/f14p2/1922 | i1_LQ_HNbud_c155403/f1p6/1468 | i1_HQ_HNbud_c90123/f2p7/1392 | i2_LQ_HNbud_c24764/f1p0/2054 |
| i1_HQ_HNbud_c36488/f7p1/1279 | i4_LQ_HNbud_c2971/f1p8/4269 | i0_LQ_HNbud_c74896/f1p3/828 | i1_HQ_HNbud_c34156/f2p0/1278 |
| i1_LQ_HNbud_c110479/f1p4/1534 | i3_LQ_HNbud_c13803/f1p15/3372 | i1_LQ_HNbud_c35013/f1p3/1749 | i1_LQ_HNbud_c249449/f1p2/1237 |
| i0_LQ_HNbud_c9109/f1p2/902 | i2_HQ_HNbud_c29688/f4p1/2229 | i0_HQ_HNbud_c97924/f4p0/695 | i2_HQ_HNbud_c47724/f4p1/2140 |
| i1_LQ_HNbud_c86284/f1p2/1501 | i1_LQ_HNbud_c228372/f1p21/1751 | i1_LQ_HNbud_c75683/f1p5/1244 | i2_LQ_HNbud_c21075/f1p47/2084 |
| i1_LQ_HNbud_c10748/f1p3/1261 | i3_HQ_HNbud_c16767/f7p1/3542 | i1_LQ_HNbud_c7761/f1p1/1363 | i1_HQ_HNbud_c167022/f6p0/1114 |
| i2_LQ_HNbud_c4886/f1p3/2133 | i2_LQ_HNbud_c37983/f1p1/2593 | i1_HQ_HNbud_c5345/f10p7/1750 | i1_LQ_HNbud_c228392/f1p0/1990 |
| i2_LQ_HNbud_c15767/f1p0/2099 | i3_HQ_HNbud_c939/f5p0/3447 | i2_HQ_HNbud_c49946/f2p3/2589 | i1_HQ_HNbud_c120862/f10p1/1692 |
| i1_LQ_HNbud_c187660/f1p106/1057 | i1_LQ_HNbud_c44982/f1p0/1031 | i0_LQ_HNbud_c11320/f1p1/857 | i2_LQ_HNbud_c43273/f1p4/2465 |
| i1_LQ_HNbud_c172459/f1p1/1420 | i3_LQ_HNbud_c13085/f1p7/3147 | i1_HQ_HNbud_c129513/f2p7/1517 | i1_LQ_HNbud_c198548/f1p5/1316 |
| i2_LQ_HNbud_c68253/f1p2/2494 | i0_LQ_HNbud_c64684/f1p0/828 | i1_LQ_HNbud_c6900/f1p2/1932 | i1_HQ_HNbud_c121095/f26p5/1023 |
| i1_HQ_HNbud_c72495/f3p1/1358 | i1_LQ_HNbud_c149047/f1p3/1562 | i1_HQ_HNbud_c2432/f5p1/1675 | i1_LQ_HNbud_c224411/f1p0/1394 |
| i1_LQ_HNbud_c139714/f1p0/1034 | i0_LQ_HNbud_c27426/f1p3/495 | i3_LQ_HNbud_c7280/f1p0/3297 | i2_HQ_HNbud_c70020/f3p4/2293 |
| i2_HQ_HNbud_c17949/f2p7/2688 | i2_HQ_HNbud_c18287/f2p7/2662 | i1_LQ_HNbud_c230049/f1p0/1202 | i1_LQ_HNbud_c106975/f1p3/1340 |
| i0_LQ_HNbud_c12152/f1p5/585 | i1_LQ_HNbud_c57286/f1p7/1696 | i1_HQ_HNbud_c264849/f3p14/1833 | i1_HQ_HNbud_c41995/f2p0/1760 |
| i2_LQ_HNbud_c59995/f1p4/2701 | i1_LQ_HNbud_c65980/f1p63/1302 | i1_LQ_HNbud_c67609/f1p4/1510 | i2_LQ_HNbud_c33438/f4p11/2267 |
| i2_LQ_HNbud_c11065/f1p1/2161 | i1_HQ_HNbud_c77310/f2p1/1071 | i1_LQ_HNbud_c200801/f1p2/1981 | i1_LQ_HNbud_c174503/f1p1/1201 |
| i1_LQ_HNbud_c48558/f2p5/1211 | i1_LQ_HNbud_c273066/f1p13/1521 | i1_HQ_HNbud_c195199/f2p3/1488 | i1_LQ_HNbud_c163289/f1p5/897 |
| i1_LQ_HNbud_c12166/f1p7/1303 | i2_LQ_HNbud_c30015/f1p0/2035 | i0_HQ_HNbud_c121135/f8p0/701 | i1_LQ_HNbud_c3824/f1p1/1454 |
| i1_LQ_HNbud_c150565/f1p1/1376 | i2_HQ_HNbud_c12831/f4p7/2442 | i1_LQ_HNbud_c70249/f1p4/1261 | i0_LQ_HNbud_c79714/f1p7/713 |
| i2_LQ_HNbud_c5167/f1p34/2287 | i2_LQ_HNbud_c20090/f1p0/2806 | i2_HQ_HNbud_c65100/f74p5/2523 | i1_LQ_HNbud_c35929/f1p3/1675 |
| i3_LQ_HNbud_c2351/f1p17/3575 | i4_LQ_HNbud_c3651/f1p2/4239 | i0_LQ_HNbud_c54889/f2p2/995 | i1_LQ_HNbud_c14163/f1p4/1531 |
| i0_HQ_HNbud_c1653/f2p5/885 | i2_LQ_HNbud_c6409/f1p1/2779 | i1_LQ_HNbud_c3733/f1p1/1211 | i1_LQ_HNbud_c91432/f1p16/1677 |
| i2_LQ_HNbud_c39556/f1p1/2898 | i3_LQ_HNbud_c7853/f1p4/3217 | i0_HQ_HNbud_c5347/f2p1/964 | i2_HQ_HNbud_c47762/f3p4/2211 |
| i2_LQ_HNbud_c18691/f1p1/2329 | i1_LQ_HNbud_c130570/f2p0/1699 | i1_LQ_HNbud_c25319/f1p2/1129 | i0_HQ_HNbud_c2156/f6p8/953 |
| i0_LQ_HNbud_c41624/f1p2/924 | i1_HQ_HNbud_c217963/f6p4/1345 | i2_LQ_HNbud_c54245/f1p21/2361 | i1_LQ_HNbud_c200601/f1p13/1752 |
| i1_LQ_HNbud_c37891/f1p2/1160 | i0_LQ_HNbud_c20707/f1p9/445 | i4_LQ_HNbud_c1198/f1p4/4766 | i0_LQ_HNbud_c18219/f1p0/945 |
| i1_LQ_HNbud_c85314/f1p5/1316 | i1_LQ_HNbud_c129793/f1p11/1832 | i1_LQ_HNbud_c251214/f1p1/1698 | i1_HQ_HNbud_c10381/f5p3/1160 |
| i3_LQ_HNbud_c14615/f1p1/3103 | i2_LQ_HNbud_c8877/f1p33/2607 | i1_HQ_HNbud_c49690/f5p1/1671 | i2_LQ_HNbud_c14168/f1p29/2331 |
| i0_LQ_HNbud_c24842/f1p4/759 | i1_HQ_HNbud_c24333/f3p1/1701 | i2_LQ_HNbud_c58485/f1p21/2482 | i1_LQ_HNbud_c2982/f3p2/1168 |
| i2_LQ_HNbud_c34626/f1p3/2104 | i1_LQ_HNbud_c66201/f1p9/1214 | i3_LQ_HNbud_c6363/f1p0/3758 | i2_LQ_HNbud_c41373/f1p2/2646 |
| i1_HQ_HNbud_c208775/f2p0/1067 | i1_HQ_HNbud_c42089/f3p36/1302 | i3_HQ_HNbud_c1863/f2p7/3357 | i1_LQ_HNbud_c112354/f1p3/1655 |
| i1_HQ_HNbud_c20339/f13p4/1257 | i1_LQ_HNbud_c86560/f1p4/1783 | i1_LQ_HNbud_c176878/f1p5/1718 | i0_LQ_HNbud_c43684/f1p2/855 |
| i1_LQ_HNbud_c161201/f1p2/1096 | i2_LQ_HNbud_c25854/f1p2/2688 | i1_HQ_HNbud_c120738/f17p5/1425 | i2_LQ_HNbud_c50313/f1p9/2589 |
| i1_HQ_HNbud_c17402/f2p1/1591 | i1_LQ_HNbud_c201935/f1p4/1179 | i2_LQ_HNbud_c11850/f1p0/2720 | i3_LQ_HNbud_c9354/f1p0/3638 |
| i0_LQ_HNbud_c46659/f1p2/992 | i1_HQ_HNbud_c144269/f2p1/1070 | i2_LQ_HNbud_c51711/f1p1/2065 | i0_HQ_HNbud_c52331/f2p1/785 |
| i1_HQ_HNbud_c22470/f8p1/1207 | i1_LQ_HNbud_c37966/f1p2/1226 | i2_LQ_HNbud_c1978/f2p0/2308 | i3_LQ_HNbud_c6906/f1p0/3505 |
| i1_LQ_HNbud_c91346/f1p2/1919 | i2_LQ_HNbud_c53001/f1p1/2491 | i1_HQ_HNbud_c31305/f59p9/1103 | i1_HQ_HNbud_c3481/f3p0/1265 |
| i1_LQ_HNbud_c161827/f1p3/1102 | i1_HQ_HNbud_c25127/f5p1/1939 | i1_HQ_HNbud_c120826/f8p5/1845 | i0_HQ_HNbud_c39416/f3p14/858 |
| i3_LQ_HNbud_c4076/f1p3/3839 | i1_LQ_HNbud_c92373/f1p4/1539 | i1_LQ_HNbud_c52234/f1p4/1414 | i1_HQ_HNbud_c67744/f4p0/1842 |
| i1_HQ_HNbud_c52189/f3p0/1752 | i1_HQ_HNbud_c244206/f7p3/1145 | i1_HQ_HNbud_c1383/f30p5/1252 | i1_LQ_HNbud_c25756/f1p0/1494 |
| i2_LQ_HNbud_c18424/f1p2/2106 | i0_LQ_HNbud_c57928/f1p1/665 | i2_LQ_HNbud_c23150/f1p2/2093 | i1_HQ_HNbud_c156223/f2p14/1638 |
| i3_LQ_HNbud_c2637/f1p12/3483 | i1_LQ_HNbud_c133446/f1p1/1575 | i2_LQ_HNbud_c26062/f1p6/2320 | i1_LQ_HNbud_c140472/f1p0/1097 |
| i2_LQ_HNbud_c40355/f1p1/2535 | i3_LQ_HNbud_c9793/f1p11/3290 | i3_LQ_HNbud_c5488/f1p0/4064 | i4_LQ_HNbud_c1863/f1p4/4582 |
| i2_HQ_HNbud_c9846/f2p22/2348 | i3_LQ_HNbud_c5625/f1p2/3407 | i3_LQ_HNbud_c14600/f1p2/3707 | i1_LQ_HNbud_c175395/f4p4/1894 |
| i2_LQ_HNbud_c18747/f1p2/2105 | i1_HQ_HNbud_c166937/f5p3/1117 | i1_HQ_HNbud_c128230/f2p3/1448 | i1_HQ_HNbud_c264853/f17p12/1622 |
| i0_HQ_HNbud_c30133/f2p0/833 | i2_LQ_HNbud_c50325/f1p2/2134 | i1_LQ_HNbud_c139119/f1p13/1028 | i0_LQ_HNbud_c75506/f1p0/969 |
| i1_LQ_HNbud_c75573/f1p7/1821 | i1_LQ_HNbud_c72785/f1p0/1251 | i1_HQ_HNbud_c41690/f2p18/1359 | i1_LQ_HNbud_c250646/f1p0/1218 |
| i1_LQ_HNbud_c204745/f1p2/1663 | i2_LQ_HNbud_c28640/f1p0/2096 | i0_LQ_HNbud_c73100/f1p1/823 | i3_LQ_HNbud_c5558/f1p1/3273 |
| i1_LQ_HNbud_c24160/f1p2/1751 | i1_LQ_HNbud_c52351/f1p1/1670 | i1_LQ_HNbud_c38448/f1p0/1413 | i1_LQ_HNbud_c223719/f1p4/1359 |
| i2_LQ_HNbud_c16144/f1p9/2025 | i1_LQ_HNbud_c37438/f1p0/1228 | i1_LQ_HNbud_c89145/f1p25/1773 | i1_LQ_HNbud_c278092/f1p0/1007 |
| i0_HQ_HNbud_c3794/f5p3/573 | i1_LQ_HNbud_c51788/f1p4/1574 | i0_HQ_HNbud_c38004/f10p0/792 | i1_LQ_HNbud_c251340/f1p0/1255 |
| i1_LQ_HNbud_c130822/f1p6/1142 | i1_HQ_HNbud_c115466/f2p0/1100 | i1_LQ_HNbud_c35014/f1p1/1325 | i0_LQ_HNbud_c47628/f1p0/745 |
| i1_HQ_HNbud_c150603/f2p2/1336 | i1_LQ_HNbud_c230105/f1p2/1843 | i0_LQ_HNbud_c75339/f1p0/727 | i0_HQ_HNbud_c2103/f3p8/930 |
| i0_LQ_HNbud_c53826/f1p5/889 | i2_HQ_HNbud_c11141/f2p0/2226 | i2_HQ_HNbud_c49903/f2p7/2293 | i1_LQ_HNbud_c49476/f1p1/1262 |
| i2_LQ_HNbud_c41869/f1p0/2299 | i1_LQ_HNbud_c147855/f1p1/1901 | i0_LQ_HNbud_c5596/f1p0/594 | i1_LQ_HNbud_c8659/f1p0/1787 |
| i3_HQ_HNbud_c1281/f3p2/3603 | i3_LQ_HNbud_c8178/f1p2/3175 | i2_LQ_HNbud_c4634/f1p1/2190 | i0_HQ_HNbud_c97963/f14p0/1002 |
| i2_LQ_HNbud_c27686/f1p6/2370 | i2_LQ_HNbud_c20572/f1p2/2916 | i1_LQ_HNbud_c52017/f1p2/1976 | i1_LQ_HNbud_c224035/f1p16/1673 |
| i1_HQ_HNbud_c55469/f2p0/1752 | i0_LQ_HNbud_c45896/f1p1/482 | i2_LQ_HNbud_c47006/f1p2/1981 | i0_LQ_HNbud_c28674/f1p0/551 |
| i2_LQ_HNbud_c50862/f1p3/2432 | i1_LQ_HNbud_c280409/f1p0/1018 | i0_LQ_HNbud_c70137/f1p0/992 | i0_LQ_HNbud_c71824/f1p2/574 |
| i1_HQ_HNbud_c221922/f2p27/1689 | i1_LQ_HNbud_c9888/f1p4/1167 | i3_LQ_HNbud_c17365/f1p4/3454 | i1_LQ_HNbud_c15113/f1p27/1095 |
| i1_LQ_HNbud_c91575/f1p1/1483 | i2_HQ_HNbud_c48482/f2p0/2082 | i0_LQ_HNbud_c4617/f1p1/916 | i0_LQ_HNbud_c3358/f1p7/729 |
| i0_LQ_HNbud_c13339/f1p2/780 | i1_HQ_HNbud_c217674/f17p4/1651 | i2_LQ_HNbud_c28493/f1p2/2516 | i3_LQ_HNbud_c13404/f1p8/3446 |
| i2_LQ_HNbud_c38530/f1p1/2428 | i1_LQ_HNbud_c69216/f1p0/1900 | i1_LQ_HNbud_c43596/f2p24/1057 | i0_LQ_HNbud_c31528/f2p0/648 |
| i1_LQ_HNbud_c20513/f3p3/1354 | i0_LQ_HNbud_c31718/f1p0/953 | i1_LQ_HNbud_c22924/f1p6/1758 | i0_LQ_HNbud_c80885/f1p0/831 |
| i0_LQ_HNbud_c74715/f1p0/570 | i1_LQ_HNbud_c12342/f1p1/2224 | i0_HQ_HNbud_c8908/f6p1/757 | i1_HQ_HNbud_c38444/f3p25/1706 |
| i1_LQ_HNbud_c65882/f1p5/1726 | i1_LQ_HNbud_c228486/f1p3/1592 | i0_LQ_HNbud_c70344/f1p4/703 | i1_LQ_HNbud_c202471/f1p2/1191 |
| i2_LQ_HNbud_c8319/f1p4/2199 | i1_LQ_HNbud_c17877/f1p1/1511 | i1_LQ_HNbud_c243209/f3p7/1163 | i0_LQ_HNbud_c41248/f1p3/513 |
| i0_LQ_HNbud_c22828/f1p0/851 | i2_LQ_HNbud_c23301/f1p3/2500 | i1_LQ_HNbud_c265419/f7p19/1119 | i3_LQ_HNbud_c5464/f1p3/3831 |
| i1_LQ_HNbud_c128097/f1p0/1539 | i0_LQ_HNbud_c55107/f1p1/809 | i1_LQ_HNbud_c155382/f1p10/2020 | i2_LQ_HNbud_c40430/f1p0/2386 |
| i1_LQ_HNbud_c155034/f1p0/1405 | i1_LQ_HNbud_c19575/f1p0/1238 | i1_LQ_HNbud_c152764/f1p0/1206 | i3_LQ_HNbud_c7788/f1p1/3200 |
| i1_HQ_HNbud_c190827/f4p2/1804 | i4_LQ_HNbud_c3919/f1p1/4173 | i1_LQ_HNbud_c172794/f1p0/1580 | i1_HQ_HNbud_c264150/f13p14/1302 |
| i1_LQ_HNbud_c21267/f1p4/1796 | i0_LQ_HNbud_c7229/f1p0/766 | i1_LQ_HNbud_c60233/f1p15/1050 | i1_HQ_HNbud_c120740/f7p2/1186 |
| i1_LQ_HNbud_c72450/f1p1/1717 | i1_LQ_HNbud_c105107/f1p0/1497 | i1_HQ_HNbud_c11267/f4p0/1581 | i0_LQ_HNbud_c5848/f1p0/490 |
| i1_HQ_HNbud_c27304/f2p0/1112 | i1_LQ_HNbud_c89494/f1p6/1835 | i2_LQ_HNbud_c21911/f1p0/2703 | i1_LQ_HNbud_c242063/f1p6/1027 |
| i0_LQ_HNbud_c80681/f1p0/705 | i1_LQ_HNbud_c171734/f1p16/1888 | i1_LQ_HNbud_c176511/f1p1/1724 | i2_HQ_HNbud_c45089/f2p1/2032 |
| i1_LQ_HNbud_c35983/f1p0/1694 | i0_HQ_HNbud_c19829/f3p1/883 | i1_LQ_HNbud_c202218/f1p4/1460 | i2_HQ_HNbud_c70443/f2p1/2646 |
| i2_HQ_HNbud_c11305/f2p5/2831 | i1_LQ_HNbud_c148275/f1p1/1162 | i0_HQ_HNbud_c1565/f7p0/613 | i3_LQ_HNbud_c8563/f1p7/3533 |
| i1_LQ_HNbud_c175734/f1p4/2000 | i1_HQ_HNbud_c15662/f12p3/1259 | i1_HQ_HNbud_c72961/f3p0/1474 | i1_LQ_HNbud_c51083/f1p2/1545 |
| i1_LQ_HNbud_c130108/f1p5/1371 | i2_LQ_HNbud_c42412/f1p1/2212 | i2_HQ_HNbud_c4271/f4p1/2498 | i3_LQ_HNbud_c3310/f1p2/3202 |
| i1_LQ_HNbud_c152747/f1p2/1098 | i1_LQ_HNbud_c234956/f2p2/1121 | i1_LQ_HNbud_c125248/f1p1/1182 | i3_LQ_HNbud_c15030/f1p4/3523 |
| i0_LQ_HNbud_c78214/f1p0/575 | i3_LQ_HNbud_c10362/f1p1/3113 | i1_LQ_HNbud_c197267/f1p4/1594 | i0_LQ_HNbud_c5501/f1p0/795 |
| i1_HQ_HNbud_c51244/f2p2/1957 | i1_LQ_HNbud_c131871/f1p4/1507 | i0_LQ_HNbud_c27916/f1p5/479 | i1_LQ_HNbud_c45597/f1p0/1076 |
| i2_HQ_HNbud_c16801/f2p7/2152 | i1_LQ_HNbud_c52388/f1p2/1468 | i1_LQ_HNbud_c80170/f1p1/1018 | i1_LQ_HNbud_c39496/f1p205/1989 |
| i1_LQ_HNbud_c106504/f1p2/1490 | i1_LQ_HNbud_c129702/f1p18/1836 | i1_LQ_HNbud_c52141/f1p5/1821 | i3_LQ_HNbud_c5905/f1p2/3232 |
| i3_LQ_HNbud_c3817/f1p6/3707 | i2_LQ_HNbud_c33684/f1p10/2754 | i4_LQ_HNbud_c1441/f1p6/4279 | i1_LQ_HNbud_c178896/f1p17/1543 |
| i2_LQ_HNbud_c40093/f1p4/2208 | i1_HQ_HNbud_c92033/f2p9/1546 | i1_HQ_HNbud_c1772/f8p1/1749 | i0_LQ_HNbud_c29800/f1p4/982 |
| i1_LQ_HNbud_c280192/f1p2/1010 | i3_LQ_HNbud_c11431/f1p0/3429 | i1_LQ_HNbud_c187620/f1p0/1062 | i2_LQ_HNbud_c25499/f1p9/2162 |
| i1_HQ_HNbud_c216568/f17p3/1201 | i1_LQ_HNbud_c41246/f1p3/1243 | i1_HQ_HNbud_c267371/f4p2/1340 | i1_HQ_HNbud_c4291/f4p3/1976 |
| i1_LQ_HNbud_c55524/f1p2/1400 | i0_LQ_HNbud_c46650/f1p0/688 | i3_LQ_HNbud_c14425/f1p0/3252 | i0_HQ_HNbud_c7301/f5p0/958 |
| i2_LQ_HNbud_c37735/f1p9/2177 | i1_HQ_HNbud_c85788/f2p4/1670 | i1_LQ_HNbud_c170683/f1p25/1766 | i1_LQ_HNbud_c133882/f1p13/1959 |
| i1_LQ_HNbud_c89426/f1p8/1659 | i1_LQ_HNbud_c200579/f1p1/1603 | i1_HQ_HNbud_c265048/f6p3/1201 | i1_LQ_HNbud_c204429/f1p1/1267 |
| i2_LQ_HNbud_c60444/f1p5/2278 | i2_HQ_HNbud_c6244/f2p3/2949 | i2_LQ_HNbud_c21925/f1p5/2444 | i1_LQ_HNbud_c208348/f3p1/1051 |
| i0_LQ_HNbud_c25839/f1p4/865 | i2_LQ_HNbud_c909/f17p1/2207 | i3_LQ_HNbud_c4383/f1p0/3461 | i0_HQ_HNbud_c1805/f6p3/584 |
| i2_LQ_HNbud_c8560/f1p7/2530 | i1_HQ_HNbud_c86428/f2p2/1870 | i2_LQ_HNbud_c51696/f1p2/2106 | i1_HQ_HNbud_c255157/f4p0/1046 |
| i0_LQ_HNbud_c8406/f1p0/821 | i2_LQ_HNbud_c34802/f1p4/2275 | i2_LQ_HNbud_c9613/f1p6/2474 | i0_LQ_HNbud_c81545/f1p0/954 |
| i1_LQ_HNbud_c41623/f1p2/1507 | i2_LQ_HNbud_c23570/f1p2/2607 | i1_LQ_HNbud_c104469/f1p3/1381 | i2_LQ_HNbud_c10176/f1p1/2082 |
| i1_LQ_HNbud_c162913/f1p6/1148 | i1_LQ_HNbud_c269575/f1p8/1017 | i2_LQ_HNbud_c40751/f1p25/2953 | i1_LQ_HNbud_c126927/f1p0/1337 |
| i3_LQ_HNbud_c5577/f1p6/3422 | i1_LQ_HNbud_c6592/f2p1/1924 | i2_LQ_HNbud_c28833/f1p2/2279 | i1_LQ_HNbud_c129637/f1p5/1827 |
| i0_LQ_HNbud_c22610/f1p0/991 | i4_LQ_HNbud_c71/f5p0/4688 | i1_HQ_HNbud_c35727/f5p1/1437 | i1_LQ_HNbud_c6784/f1p0/1391 |
| i0_HQ_HNbud_c17047/f26p0/771 | i4_LQ_HNbud_c501/f1p0/4460 | i1_HQ_HNbud_c39670/f3p2/1918 | i1_HQ_HNbud_c90251/f4p4/1893 |
| i1_LQ_HNbud_c222584/f1p6/1287 | i1_LQ_HNbud_c131285/f1p0/1567 | i1_HQ_HNbud_c88477/f3p1/1319 | i2_HQ_HNbud_c49970/f2p0/2132 |
| i2_LQ_HNbud_c8785/f1p3/2691 | i1_LQ_HNbud_c263349/f1p2/1156 | i2_LQ_HNbud_c57242/f1p23/2217 | i1_LQ_HNbud_c190389/f1p2/979 |
| i1_LQ_HNbud_c75675/f1p1/1323 | i1_HQ_HNbud_c1858/f3p0/1183 | i1_LQ_HNbud_c235305/f1p1/1041 | i0_LQ_HNbud_c9792/f1p0/892 |
| i1_HQ_HNbud_c4375/f3p1/1519 | i1_LQ_HNbud_c10318/f1p3/1320 | i1_HQ_HNbud_c181742/f3p2/1016 | i0_LQ_HNbud_c8252/f1p9/938 |
| i1_LQ_HNbud_c149658/f1p2/1727 | i3_HQ_HNbud_c1008/f4p0/3804 | i1_LQ_HNbud_c26344/f1p1/1504 | i1_LQ_HNbud_c188248/f2p8/1515 |
| i1_LQ_HNbud_c77990/f1p1/1531 | i1_LQ_HNbud_c73383/f1p1/1142 | i1_HQ_HNbud_c1416/f9p0/1283 | i0_HQ_HNbud_c2323/f8p4/863 |
| i1_HQ_HNbud_c49776/f6p0/1762 | i3_LQ_HNbud_c16089/f1p0/3023 | i2_LQ_HNbud_c58585/f1p12/2378 | i2_LQ_HNbud_c25658/f1p7/2228 |
| i0_LQ_HNbud_c68588/f1p0/885 | i1_LQ_HNbud_c202127/f1p10/1022 | i0_LQ_HNbud_c41469/f1p0/618 | i1_HQ_HNbud_c246227/f7p8/1415 |
| i3_HQ_HNbud_c16690/f14p7/3197 | i1_HQ_HNbud_c95416/f3p1/1074 | i1_HQ_HNbud_c92444/f2p1/1949 | i0_LQ_HNbud_c8658/f1p27/755 |
| i1_HQ_HNbud_c263386/f6p5/1215 | i0_LQ_HNbud_c84679/f2p0/963 | i0_HQ_HNbud_c18664/f2p5/578 | i1_HQ_HNbud_c101295/f25p29/1402 |
| i1_HQ_HNbud_c17433/f3p1/1363 | i2_LQ_HNbud_c56664/f1p5/1905 | i2_LQ_HNbud_c24677/f1p1/2201 | i1_HQ_HNbud_c264488/f2p2/1939 |
| i2_LQ_HNbud_c37085/f1p3/2241 | i1_LQ_HNbud_c227344/f1p1/1730 | i0_HQ_HNbud_c13959/f2p0/851 | i1_LQ_HNbud_c229003/f1p5/1466 |
| i1_LQ_HNbud_c152340/f1p1/1716 | i2_LQ_HNbud_c37311/f1p7/2861 | i1_LQ_HNbud_c9435/f1p13/1489 | i1_LQ_HNbud_c202139/f1p4/1966 |
| i1_LQ_HNbud_c65982/f1p0/1277 | i3_LQ_HNbud_c14956/f1p0/3445 | i0_HQ_HNbud_c1706/f5p0/753 | i3_LQ_HNbud_c4404/f1p2/3762 |
| i3_LQ_HNbud_c5107/f1p0/3895 | i0_LQ_HNbud_c27067/f1p4/778 | i3_LQ_HNbud_c9088/f1p0/3191 | i2_HQ_HNbud_c10897/f2p4/2417 |
| i1_LQ_HNbud_c9283/f1p1/1404 | i0_LQ_HNbud_c21641/f1p6/986 | i1_LQ_HNbud_c155511/f1p26/1403 | i2_HQ_HNbud_c24447/f2p2/2188 |
| i2_LQ_HNbud_c46368/f1p1/2052 | i1_LQ_HNbud_c149995/f1p13/1351 | i1_HQ_HNbud_c4634/f8p1/1752 | i1_HQ_HNbud_c222766/f3p3/1277 |
| i1_HQ_HNbud_c8063/f9p1/1487 | i3_LQ_HNbud_c11001/f1p4/3085 | i1_LQ_HNbud_c44127/f1p4/1082 | i1_LQ_HNbud_c7969/f1p0/1769 |
| i1_LQ_HNbud_c162987/f1p0/1063 | i1_HQ_HNbud_c208838/f2p1/1096 | i1_HQ_HNbud_c36032/f6p3/1129 | i1_LQ_HNbud_c147879/f1p8/1466 |
| i3_LQ_HNbud_c9586/f1p3/3931 | i1_HQ_HNbud_c24453/f7p18/1474 | i1_LQ_HNbud_c9397/f1p0/1543 | i1_LQ_HNbud_c236299/f1p1/1070 |
| i1_HQ_HNbud_c192226/f98p20/1453 | i0_LQ_HNbud_c73301/f1p1/601 | i2_LQ_HNbud_c26376/f1p2/2075 | i2_LQ_HNbud_c45562/f1p1/2013 |
| i1_LQ_HNbud_c34908/f2p0/1724 | i1_HQ_HNbud_c100408/f12p2/1560 | i1_LQ_HNbud_c196301/f1p3/1323 | i2_LQ_HNbud_c26712/f1p2/2277 |
| i3_HQ_HNbud_c18150/f15p1/3093 | i4_HQ_HNbud_c280/f2p0/4348 | i1_LQ_HNbud_c279608/f1p3/1022 | i1_LQ_HNbud_c3412/f1p1/1128 |
| i1_LQ_HNbud_c72997/f1p13/1481 | i1_HQ_HNbud_c10848/f8p2/1436 | i2_LQ_HNbud_c29878/f1p1/2017 | i0_LQ_HNbud_c23827/f1p6/740 |
| i2_LQ_HNbud_c37411/f1p11/2894 | i1_HQ_HNbud_c183777/f2p0/1068 | i1_HQ_HNbud_c192748/f14p3/1503 | i0_HQ_HNbud_c97932/f13p0/937 |
| i3_LQ_HNbud_c15296/f1p8/3034 | i0_LQ_HNbud_c1730/f3p1/810 | i1_LQ_HNbud_c125411/f1p2/1323 | i1_LQ_HNbud_c108001/f1p0/1894 |
| i1_LQ_HNbud_c130098/f1p4/1339 | i1_LQ_HNbud_c10138/f1p7/1423 | i0_LQ_HNbud_c40674/f1p0/732 | i4_LQ_HNbud_c4257/f1p0/4026 |
| i1_LQ_HNbud_c103946/f1p2/1411 | i3_LQ_HNbud_c9853/f1p6/3452 | i1_LQ_HNbud_c222643/f1p4/1357 | i5_LQ_HNbud_c1167/f1p0/5233 |
| i1_LQ_HNbud_c32952/f1p0/1428 | i2_HQ_HNbud_c12779/f2p10/2242 | i1_LQ_HNbud_c129839/f1p7/1290 | i1_LQ_HNbud_c71192/f1p2/1748 |
| i2_LQ_HNbud_c43931/f1p1/2666 | i1_HQ_HNbud_c88355/f4p7/1748 | i2_LQ_HNbud_c12872/f1p1/2335 | i0_LQ_HNbud_c20107/f1p3/864 |
| i2_LQ_HNbud_c42253/f1p14/2888 | i3_HQ_HNbud_c1535/f2p0/3765 | i1_LQ_HNbud_c146122/f1p0/1566 | i1_LQ_HNbud_c131838/f1p3/1666 |
| i1_HQ_HNbud_c106734/f3p1/1700 | i2_LQ_HNbud_c13007/f1p1/2950 | i1_LQ_HNbud_c241018/f1p12/1064 | i0_HQ_HNbud_c4289/f5p2/862 |
| i0_LQ_HNbud_c82426/f1p0/947 | i3_LQ_HNbud_c8835/f1p2/3570 | i1_LQ_HNbud_c52743/f1p2/1536 | i1_HQ_HNbud_c43970/f3p14/1293 |
| i3_LQ_HNbud_c12594/f1p2/3401 | i1_LQ_HNbud_c20567/f1p5/1277 | i1_LQ_HNbud_c28585/f2p1/1080 | i0_LQ_HNbud_c48044/f1p11/752 |
| i2_LQ_HNbud_c1060/f15p1/2387 | i2_LQ_HNbud_c52388/f1p7/2344 | i4_LQ_HNbud_c2066/f1p0/4270 | i1_LQ_HNbud_c203468/f1p3/1867 |
| i3_LQ_HNbud_c15860/f1p1/3038 | i1_LQ_HNbud_c149517/f1p4/2003 | i0_HQ_HNbud_c9934/f2p1/966 | i1_LQ_HNbud_c49555/f1p16/1851 |
| i1_HQ_HNbud_c67684/f6p1/1317 | i1_LQ_HNbud_c201797/f1p6/1462 | i1_HQ_HNbud_c104872/f4p6/1766 | i1_HQ_HNbud_c24265/f3p3/1379 |
| i2_LQ_HNbud_c25660/f1p8/2622 | i2_LQ_HNbud_c37577/f1p6/2166 | i1_LQ_HNbud_c154086/f1p3/1961 | i1_LQ_HNbud_c85190/f1p5/1967 |
| i0_LQ_HNbud_c53838/f1p3/767 | i1_LQ_HNbud_c58499/f1p5/1686 | i0_LQ_HNbud_c4111/f2p11/515 | i0_LQ_HNbud_c18603/f2p1/773 |
| i1_LQ_HNbud_c124384/f3p0/1554 | i1_LQ_HNbud_c51221/f1p2/1625 | i2_HQ_HNbud_c7039/f2p4/2565 | i1_LQ_HNbud_c10155/f1p5/1646 |
| i1_LQ_HNbud_c170526/f1p11/1400 | i0_LQ_HNbud_c45309/f1p0/685 | i0_LQ_HNbud_c79127/f1p0/734 | i0_LQ_HNbud_c84486/f1p0/796 |
| i1_LQ_HNbud_c178813/f1p1/1103 | i2_LQ_HNbud_c37295/f1p13/2230 | i1_LQ_HNbud_c37997/f1p3/1464 | i1_LQ_HNbud_c75750/f1p1/1551 |
| i1_HQ_HNbud_c59710/f2p3/1038 | i0_LQ_HNbud_c19279/f2p0/857 | i1_LQ_HNbud_c103798/f1p0/1113 | i2_LQ_HNbud_c19676/f1p3/2761 |
| i1_HQ_HNbud_c81986/f3p0/1419 | i1_LQ_HNbud_c126547/f1p6/1697 | i2_LQ_HNbud_c26196/f1p11/2236 | i1_LQ_HNbud_c75390/f1p1/1162 |
| i0_HQ_HNbud_c97853/f5p0/763 | i2_LQ_HNbud_c53316/f1p5/2615 | i1_LQ_HNbud_c15159/f1p0/1089 | i0_LQ_HNbud_c70266/f1p2/671 |
| i1_LQ_HNbud_c200919/f1p5/1609 | i2_LQ_HNbud_c7186/f1p9/2833 | i1_LQ_HNbud_c228250/f1p3/1655 | i1_LQ_HNbud_c74505/f1p1/1344 |
| i4_LQ_HNbud_c3343/f1p0/4731 | i0_LQ_HNbud_c43898/f1p0/878 | i1_LQ_HNbud_c129928/f1p0/1489 | i1_LQ_HNbud_c230244/f2p2/1906 |
| i1_LQ_HNbud_c74832/f1p0/1638 | i0_LQ_HNbud_c82682/f1p0/760 | i2_HQ_HNbud_c2139/f4p1/2304 | i1_LQ_HNbud_c167535/f12p5/1124 |
| i2_LQ_HNbud_c12744/f1p0/2626 | i1_LQ_HNbud_c20313/f1p0/1294 | i2_LQ_HNbud_c51070/f1p3/2407 | i2_LQ_HNbud_c56606/f1p4/2068 |
| i0_LQ_HNbud_c80353/f1p0/686 | i1_HQ_HNbud_c168660/f3p1/1102 | i1_HQ_HNbud_c5148/f2p3/1137 | i0_LQ_HNbud_c44427/f1p0/689 |
| i2_LQ_HNbud_c55186/f1p7/2029 | i1_HQ_HNbud_c265399/f5p8/1022 | i1_LQ_HNbud_c248317/f1p5/1728 | i0_LQ_HNbud_c2850/f2p0/900 |
| i1_LQ_HNbud_c6760/f1p11/1574 | i2_LQ_HNbud_c38175/f1p3/2204 | i0_LQ_HNbud_c7594/f1p0/832 | i1_LQ_HNbud_c162923/f1p0/1089 |
| i2_LQ_HNbud_c36266/f1p1/2933 | i1_HQ_HNbud_c92656/f4p3/1291 | i1_LQ_HNbud_c132708/f1p2/1611 | i1_LQ_HNbud_c90831/f1p114/1213 |
| i1_LQ_HNbud_c132913/f1p2/1270 | i1_HQ_HNbud_c112017/f4p1/1453 | i1_LQ_HNbud_c36592/f1p3/1359 | i1_LQ_HNbud_c87948/f1p0/1183 |
| i1_HQ_HNbud_c5213/f15p6/1355 | i1_LQ_HNbud_c129333/f1p3/1487 | i1_LQ_HNbud_c96769/f1p1/1041 | i1_LQ_HNbud_c163409/f1p1/1023 |
| i1_LQ_HNbud_c161924/f1p0/1087 | i1_HQ_HNbud_c144884/f3p5/1830 | i1_HQ_HNbud_c12193/f4p2/1595 | i6_LQ_HNbud_c2003/f1p0/6644 |
| i1_HQ_HNbud_c5496/f2p0/1274 | i4_LQ_HNbud_c2147/f1p5/4336 | i0_LQ_HNbud_c72168/f1p1/736 | i1_HQ_HNbud_c192291/f4p9/1100 |
| i1_HQ_HNbud_c167511/f26p1/1092 | i0_HQ_HNbud_c66767/f2p0/635 | i1_LQ_HNbud_c13502/f1p2/1133 | i1_HQ_HNbud_c35934/f4p1/1752 |
| i1_LQ_HNbud_c248789/f1p9/1623 | i1_HQ_HNbud_c2353/f10p9/1283 | i1_HQ_HNbud_c95719/f2p14/1018 | i1_LQ_HNbud_c259351/f1p3/1070 |
| i1_HQ_HNbud_c4886/f4p2/1460 | i1_LQ_HNbud_c129960/f1p2/1840 | i2_LQ_HNbud_c31264/f1p5/2129 | i2_LQ_HNbud_c22583/f1p12/2056 |
| i0_LQ_HNbud_c52007/f1p0/719 | i0_LQ_HNbud_c68860/f1p0/750 | i1_LQ_HNbud_c20846/f1p2/1710 | i2_LQ_HNbud_c35157/f1p6/2395 |
| i1_LQ_HNbud_c21779/f1p1/1584 | i3_LQ_HNbud_c4947/f1p1/3315 | i2_LQ_HNbud_c21485/f1p4/2485 | i0_LQ_HNbud_c27903/f1p4/576 |
| i1_LQ_HNbud_c4142/f1p10/1753 | i1_LQ_HNbud_c7581/f1p10/1535 | i0_LQ_HNbud_c19570/f1p15/831 | i1_LQ_HNbud_c128811/f1p0/1524 |
| i1_HQ_HNbud_c131230/f2p4/1613 | i1_LQ_HNbud_c19420/f1p5/1890 | i2_LQ_HNbud_c12002/f2p3/2952 | i1_HQ_HNbud_c226961/f4p5/1703 |
| i2_LQ_HNbud_c50518/f1p16/2729 | i2_HQ_HNbud_c6015/f2p2/2851 | i1_LQ_HNbud_c49093/f1p3/1162 | i1_LQ_HNbud_c89835/f1p3/1517 |
| i2_LQ_HNbud_c57851/f1p16/2453 | i2_LQ_HNbud_c14565/f1p7/2917 | i1_LQ_HNbud_c39771/f1p1/1223 | i1_LQ_HNbud_c20736/f1p6/1218 |
| i1_LQ_HNbud_c103800/f1p1/1422 | i0_LQ_HNbud_c79984/f1p0/734 | i1_LQ_HNbud_c201467/f1p1/1354 | i0_LQ_HNbud_c7114/f1p2/996 |
| i1_HQ_HNbud_c183800/f2p2/1039 | i1_LQ_HNbud_c278171/f1p5/1013 | i1_HQ_HNbud_c136043/f4p5/1052 | i1_HQ_HNbud_c67735/f2p2/1437 |
| i4_LQ_HNbud_c1506/f1p0/4222 | i1_LQ_HNbud_c58464/f1p20/1185 | i5_LQ_HNbud_c622/f1p0/5559 | i1_HQ_HNbud_c1957/f3p4/1594 |
| i2_LQ_HNbud_c4760/f1p4/2396 | i0_LQ_HNbud_c44503/f1p0/941 | i2_HQ_HNbud_c70530/f3p1/2949 | i1_HQ_HNbud_c219662/f21p7/1625 |
| i1_LQ_HNbud_c231130/f2p1/1380 | i2_LQ_HNbud_c53347/f1p1/2081 | i1_LQ_HNbud_c8608/f1p0/1250 | i1_LQ_HNbud_c71958/f2p0/1222 |
| i0_LQ_HNbud_c2310/f2p12/823 | i0_LQ_HNbud_c13673/f2p2/816 | i3_LQ_HNbud_c9508/f1p3/3457 | i1_LQ_HNbud_c200629/f1p7/1585 |
| i1_HQ_HNbud_c67015/f2p1/1539 | i1_LQ_HNbud_c163888/f1p4/1172 | i1_LQ_HNbud_c249303/f1p10/1883 | i1_LQ_HNbud_c40151/f1p3/1225 |
| i1_HQ_HNbud_c145254/f4p1/1259 | i0_LQ_HNbud_c2494/f1p2/569 | i2_LQ_HNbud_c43694/f1p11/2682 | i2_LQ_HNbud_c22848/f1p2/2133 |
| i1_HQ_HNbud_c16289/f5p5/1431 | i1_LQ_HNbud_c53857/f1p2/1365 | i2_LQ_HNbud_c6694/f1p3/2127 | i1_LQ_HNbud_c91757/f1p2/1188 |
| i1_HQ_HNbud_c87213/f2p1/1177 | i1_LQ_HNbud_c24498/f2p1/2024 | i1_LQ_HNbud_c98257/f1p3/1071 | i1_HQ_HNbud_c208686/f2p8/1042 |
| i1_HQ_HNbud_c145257/f3p0/1378 | i1_LQ_HNbud_c152837/f1p14/1652 | i1_LQ_HNbud_c173735/f1p3/1414 | i2_LQ_HNbud_c53889/f1p8/2928 |
| i2_HQ_HNbud_c20160/f2p2/2455 | i1_LQ_HNbud_c241324/f1p2/972 | i0_LQ_HNbud_c26487/f1p0/966 | i1_HQ_HNbud_c2390/f6p3/1496 |
| i1_LQ_HNbud_c267508/f2p0/2012 | i2_LQ_HNbud_c53811/f1p8/2895 | i1_HQ_HNbud_c7546/f15p5/1738 | i1_HQ_HNbud_c200231/f4p4/1345 |
| i1_LQ_HNbud_c116538/f1p1/1033 | i2_LQ_HNbud_c43910/f1p6/2493 | i0_LQ_HNbud_c73984/f1p3/913 | i1_LQ_HNbud_c87235/f1p0/1409 |
| i1_LQ_HNbud_c41660/f1p3/1320 | i3_LQ_HNbud_c13447/f1p0/3579 | i1_LQ_HNbud_c3549/f1p1/1289 | i1_LQ_HNbud_c33467/f1p1/1485 |
| i1_LQ_HNbud_c132641/f1p16/1706 | i2_LQ_HNbud_c23410/f1p1/2219 | i0_HQ_HNbud_c4709/f4p1/932 | i1_HQ_HNbud_c71859/f2p6/1336 |
| i4_LQ_HNbud_c3210/f1p3/4616 | i1_LQ_HNbud_c171551/f1p9/1687 | i0_LQ_HNbud_c11430/f3p9/521 | i1_LQ_HNbud_c35881/f1p3/1418 |
| i2_HQ_HNbud_c65772/f2p9/2455 | i1_LQ_HNbud_c110216/f1p5/1445 | i0_LQ_HNbud_c3573/f1p6/677 | i1_HQ_HNbud_c74440/f3p1/1498 |
| i1_LQ_HNbud_c54572/f1p1/1579 | i2_LQ_HNbud_c50260/f1p11/2289 | i1_LQ_HNbud_c263006/f1p0/1092 | i2_LQ_HNbud_c35003/f1p3/2825 |
| i1_LQ_HNbud_c130587/f1p4/1875 | i1_LQ_HNbud_c50118/f1p0/1802 | i1_HQ_HNbud_c63014/f7p6/1862 | i1_LQ_HNbud_c106029/f1p7/1588 |
| i1_LQ_HNbud_c19588/f1p2/1928 | i1_LQ_HNbud_c58387/f2p1/1190 | i1_LQ_HNbud_c56925/f1p0/1217 | i1_LQ_HNbud_c86607/f1p5/1838 |
| i2_LQ_HNbud_c37354/f1p5/2731 | i2_LQ_HNbud_c10338/f1p0/2377 | i1_LQ_HNbud_c253467/f1p6/1542 | i1_LQ_HNbud_c54025/f1p0/1488 |
| i2_HQ_HNbud_c33647/f2p7/2345 | i1_LQ_HNbud_c55330/f1p13/1447 | i1_LQ_HNbud_c3170/f1p27/1428 | i2_LQ_HNbud_c25944/f1p2/2882 |
| i2_LQ_HNbud_c6863/f1p16/2370 | i1_HQ_HNbud_c18274/f5p11/1771 | i1_LQ_HNbud_c55021/f1p0/1372 | i5_HQ_HNbud_c84/f3p0/5466 |
| i2_LQ_HNbud_c38311/f1p105/2646 | i2_LQ_HNbud_c37192/f1p0/2238 | i0_LQ_HNbud_c52951/f1p1/852 | i1_HQ_HNbud_c264929/f8p3/1211 |
| i2_LQ_HNbud_c58255/f1p0/2084 | i2_LQ_HNbud_c41580/f1p8/2778 | i1_LQ_HNbud_c250342/f1p0/1388 | i0_LQ_HNbud_c46005/f1p0/836 |
| i1_LQ_HNbud_c51511/f1p12/1679 | i1_LQ_HNbud_c93266/f1p3/1739 | i1_HQ_HNbud_c55389/f2p0/1519 | i1_LQ_HNbud_c88644/f1p3/1803 |
| i0_HQ_HNbud_c57610/f3p33/712 | i3_LQ_HNbud_c18652/f1p0/3002 | i1_LQ_HNbud_c171545/f1p1/1749 | i1_LQ_HNbud_c171929/f1p2/1580 |
| i2_LQ_HNbud_c35278/f1p13/2513 | i4_LQ_HNbud_c2404/f1p1/4665 | i5_LQ_HNbud_c1907/f1p0/5042 | i1_LQ_HNbud_c17914/f1p6/1876 |
| i1_LQ_HNbud_c24256/f1p3/1398 | i1_LQ_HNbud_c174321/f1p5/1361 | i1_LQ_HNbud_c4540/f1p0/1643 | i1_LQ_HNbud_c6922/f1p0/1920 |
| i1_LQ_HNbud_c81636/f3p4/1114 | i1_HQ_HNbud_c23541/f10p6/1420 | i1_LQ_HNbud_c231398/f1p11/1099 | i0_LQ_HNbud_c80760/f1p0/799 |
| i1_LQ_HNbud_c126439/f1p0/1912 | i0_LQ_HNbud_c27396/f1p15/635 | i2_HQ_HNbud_c9693/f4p1/2711 | i2_LQ_HNbud_c34618/f1p8/2134 |
| i4_LQ_HNbud_c1121/f1p0/4852 | i2_LQ_HNbud_c68841/f1p1/2168 | i1_LQ_HNbud_c127710/f1p0/1447 | i1_LQ_HNbud_c204014/f1p1/1975 |
| i3_LQ_HNbud_c14381/f1p1/3143 | i2_LQ_HNbud_c34812/f1p2/2448 | i0_LQ_HNbud_c77080/f1p0/919 | i1_HQ_HNbud_c145351/f6p3/1289 |
| i1_HQ_HNbud_c17556/f3p0/1824 | i1_HQ_HNbud_c32619/f8p2/1123 | i1_LQ_HNbud_c151937/f1p9/1261 | i0_HQ_HNbud_c9875/f6p2/951 |
| i0_LQ_HNbud_c4917/f1p2/929 | i1_LQ_HNbud_c152028/f1p1/1844 | i0_LQ_HNbud_c24481/f1p3/917 | i1_LQ_HNbud_c130451/f1p206/1719 |
| i0_LQ_HNbud_c9262/f9p0/730 | i0_HQ_HNbud_c21145/f2p0/946 | i2_LQ_HNbud_c61822/f2p0/2235 | i2_LQ_HNbud_c13912/f1p6/3028 |
| i0_LQ_HNbud_c83267/f1p0/870 | i0_LQ_HNbud_c54136/f1p1/608 | i1_LQ_HNbud_c251912/f1p3/1377 | i1_LQ_HNbud_c150696/f1p1/1825 |
| i1_HQ_HNbud_c95787/f2p2/1098 | i1_LQ_HNbud_c14297/f1p1/1395 | i3_HQ_HNbud_c1423/f2p1/3359 | i2_HQ_HNbud_c1307/f7p1/2814 |
| i2_LQ_HNbud_c46146/f1p2/2055 | i1_LQ_HNbud_c90244/f1p11/1160 | i0_LQ_HNbud_c19606/f1p4/775 | i2_LQ_HNbud_c64042/f1p4/2061 |
| i3_LQ_HNbud_c2292/f1p0/3177 | i0_LQ_HNbud_c30233/f1p0/911 | i2_LQ_HNbud_c46045/f1p1/2016 | i2_LQ_HNbud_c53980/f1p2/2164 |
| i0_LQ_HNbud_c46455/f1p0/793 | i1_HQ_HNbud_c25485/f2p9/1709 | i1_HQ_HNbud_c53507/f2p1/1236 | i2_HQ_HNbud_c19085/f3p2/2429 |
| i0_LQ_HNbud_c5581/f1p1/778 | i1_HQ_HNbud_c12739/f13p3/1208 | i3_LQ_HNbud_c4184/f1p4/3104 | i1_HQ_HNbud_c23889/f3p3/1634 |
| i1_HQ_HNbud_c271175/f2p3/1384 | i2_LQ_HNbud_c52962/f1p1/2433 | i1_LQ_HNbud_c154149/f1p3/1120 | i3_HQ_HNbud_c1290/f3p7/3574 |
| i1_LQ_HNbud_c278086/f1p0/998 | i1_HQ_HNbud_c57107/f5p4/1156 | i2_LQ_HNbud_c11461/f1p0/2120 | i3_LQ_HNbud_c15599/f1p5/3053 |
| i1_LQ_HNbud_c199874/f1p1/1571 | i0_LQ_HNbud_c69550/f1p0/692 | i2_HQ_HNbud_c64578/f5p1/2126 | i1_LQ_HNbud_c211072/f1p2/1568 |
| i0_LQ_HNbud_c77498/f1p0/568 | i2_LQ_HNbud_c9067/f1p2/2507 | i0_LQ_HNbud_c6289/f1p2/792 | i1_LQ_HNbud_c48343/f7p8/1982 |
| i0_LQ_HNbud_c13523/f2p3/557 | i1_HQ_HNbud_c18933/f2p3/1966 | i1_HQ_HNbud_c6254/f4p0/1349 | i1_HQ_HNbud_c219380/f23p1/1276 |
| i1_LQ_HNbud_c4430/f1p5/1508 | i1_LQ_HNbud_c196711/f1p0/1726 | i1_LQ_HNbud_c21323/f1p1/1488 | i2_LQ_HNbud_c35097/f1p5/2814 |
| i2_LQ_HNbud_c59407/f1p6/2780 | i2_LQ_HNbud_c41787/f1p3/2506 | i1_LQ_HNbud_c150212/f1p0/1757 | i1_LQ_HNbud_c125026/f1p0/1657 |
| i0_LQ_HNbud_c76109/f1p0/832 | i1_LQ_HNbud_c171647/f1p2/1789 | i1_LQ_HNbud_c58377/f1p9/1522 | i0_LQ_HNbud_c47945/f1p0/926 |
| i1_HQ_HNbud_c115534/f2p2/1037 | i0_LQ_HNbud_c29704/f1p0/713 | i2_LQ_HNbud_c29719/f2p0/2110 | i3_LQ_HNbud_c4722/f1p3/3252 |
| i0_LQ_HNbud_c27083/f1p1/969 | i0_LQ_HNbud_c47692/f1p1/591 | i2_LQ_HNbud_c11059/f1p1/2187 | i1_LQ_HNbud_c266029/f3p36/1731 |
| i2_LQ_HNbud_c63053/f1p2/2027 | i2_LQ_HNbud_c13898/f1p4/2562 | i4_LQ_HNbud_c368/f1p0/4373 | i1_LQ_HNbud_c133734/f1p1/1880 |
| i2_LQ_HNbud_c55818/f1p13/2420 | i1_LQ_HNbud_c173752/f1p4/1972 | i1_LQ_HNbud_c55972/f1p0/1930 | i1_HQ_HNbud_c121675/f2p1/1440 |
| i1_LQ_HNbud_c154756/f1p1/1642 | i1_LQ_HNbud_c214053/f1p4/1023 | i2_HQ_HNbud_c1803/f6p14/2309 | i4_LQ_HNbud_c4056/f1p2/4059 |
| i2_LQ_HNbud_c3188/f1p0/2391 | i2_LQ_HNbud_c37254/f1p9/2880 | i2_LQ_HNbud_c9938/f1p2/2691 | i1_LQ_HNbud_c130140/f1p7/1790 |
| i1_LQ_HNbud_c5731/f1p3/1350 | i1_LQ_HNbud_c38745/f1p7/1771 | i3_LQ_HNbud_c8053/f1p3/3671 | i0_LQ_HNbud_c9497/f2p36/889 |
| i1_LQ_HNbud_c15435/f1p26/1084 | i2_LQ_HNbud_c24242/f1p5/2769 | i1_LQ_HNbud_c106836/f1p4/1719 | i1_LQ_HNbud_c29924/f1p5/1102 |
| i3_LQ_HNbud_c16475/f1p0/3046 | i2_LQ_HNbud_c43118/f1p3/2423 | i2_LQ_HNbud_c6705/f1p1/2264 | i2_LQ_HNbud_c63512/f1p4/2040 |
| i1_HQ_HNbud_c264878/f5p5/1477 | i0_LQ_HNbud_c78750/f1p0/782 | i0_LQ_HNbud_c19968/f1p5/608 | i1_LQ_HNbud_c93642/f1p0/1389 |
| i1_HQ_HNbud_c132801/f2p1/1487 | i1_LQ_HNbud_c242175/f1p32/1017 | i1_HQ_HNbud_c23202/f3p8/1151 | i1_LQ_HNbud_c56716/f1p11/1574 |
| i1_LQ_HNbud_c205902/f1p6/1435 | i2_LQ_HNbud_c24583/f1p2/2599 | i2_LQ_HNbud_c5803/f1p5/2684 | i2_LQ_HNbud_c18548/f1p4/2450 |
| i0_LQ_HNbud_c11200/f1p2/660 | i0_LQ_HNbud_c7567/f1p0/689 | i0_LQ_HNbud_c71258/f1p2/760 | i3_LQ_HNbud_c3183/f1p4/3530 |
| i2_LQ_HNbud_c14113/f1p2/2765 | i1_HQ_HNbud_c53223/f5p2/1594 | i2_LQ_HNbud_c15745/f1p30/2030 | i2_LQ_HNbud_c22259/f1p0/2294 |
| i4_LQ_HNbud_c3940/f1p1/4987 | i1_LQ_HNbud_c85769/f3p4/1693 | i1_LQ_HNbud_c147662/f1p19/1395 | i1_LQ_HNbud_c69178/f1p2/1698 |
| i3_HQ_HNbud_c17037/f2p0/3049 | i0_LQ_HNbud_c69967/f1p0/732 | i1_HQ_HNbud_c85813/f3p5/1581 | i3_LQ_HNbud_c6244/f1p1/3243 |
| i1_LQ_HNbud_c125219/f1p0/1182 | i1_LQ_HNbud_c279087/f1p9/1002 | i3_LQ_HNbud_c11590/f1p0/3907 | i4_LQ_HNbud_c1914/f1p4/4101 |
| i1_LQ_HNbud_c85807/f1p0/1358 | i0_HQ_HNbud_c10670/f2p1/898 | i1_LQ_HNbud_c66510/f1p5/1377 | i1_LQ_HNbud_c74930/f1p6/1245 |
| i1_HQ_HNbud_c190899/f5p2/1990 | i2_HQ_HNbud_c65894/f6p3/2047 | i1_LQ_HNbud_c25234/f1p13/1730 | i2_LQ_HNbud_c53879/f1p4/2504 |
| i1_HQ_HNbud_c27379/f2p8/1575 | i1_HQ_HNbud_c8706/f12p2/1310 | i1_LQ_HNbud_c179170/f1p2/1127 | i1_LQ_HNbud_c252204/f1p3/1483 |
| i1_LQ_HNbud_c227916/f1p0/1197 | i1_LQ_HNbud_c49485/f1p0/1294 | i1_LQ_HNbud_c21085/f1p1/1570 | i0_LQ_HNbud_c26116/f2p3/926 |
| i1_HQ_HNbud_c34914/f3p7/1222 | i1_LQ_HNbud_c6511/f1p3/1663 | i1_HQ_HNbud_c36286/f14p4/1209 | i2_LQ_HNbud_c54733/f1p8/2091 |
| i1_LQ_HNbud_c200175/f1p0/1202 | i2_LQ_HNbud_c2372/f1p0/2347 | i1_HQ_HNbud_c148352/f2p3/1708 | i4_LQ_HNbud_c2728/f1p1/4089 |
| i0_LQ_HNbud_c46453/f1p59/994 | i2_HQ_HNbud_c1922/f9p4/2227 | i1_LQ_HNbud_c70291/f1p0/1679 | i2_LQ_HNbud_c18512/f1p6/2337 |
| i1_LQ_HNbud_c92277/f1p5/1537 | i0_LQ_HNbud_c19113/f1p3/629 | i1_HQ_HNbud_c36565/f7p3/1793 | i1_LQ_HNbud_c19834/f1p9/1542 |
| i0_HQ_HNbud_c28788/f3p0/776 | i4_LQ_HNbud_c3027/f1p0/4584 | i0_LQ_HNbud_c29638/f1p0/856 | i0_LQ_HNbud_c5844/f1p3/738 |
| i1_HQ_HNbud_c75726/f2p1/2001 | i2_LQ_HNbud_c19378/f1p3/2195 | i0_LQ_HNbud_c74443/f1p0/752 | i0_LQ_HNbud_c75553/f1p0/638 |
| i7_LQ_HNbud_c133/f1p0/7417 | i1_LQ_HNbud_c180442/f1p5/1436 | i0_LQ_HNbud_c45898/f1p0/630 | i1_HQ_HNbud_c84136/f4p11/1999 |
| i1_HQ_HNbud_c53234/f4p8/1560 | i0_LQ_HNbud_c81591/f1p0/765 | i4_LQ_HNbud_c2495/f1p0/4147 | i2_LQ_HNbud_c33889/f1p2/2151 |
| i1_HQ_HNbud_c132864/f2p7/1713 | i1_HQ_HNbud_c53791/f3p3/1378 | i5_LQ_HNbud_c843/f1p2/5324 | i2_HQ_HNbud_c65871/f10p4/2043 |
| i0_LQ_HNbud_c83218/f1p0/906 | i1_LQ_HNbud_c57511/f1p4/2020 | i1_LQ_HNbud_c187954/f1p4/1039 | i1_LQ_HNbud_c226407/f1p28/1233 |
| i1_LQ_HNbud_c191216/f35p10/1222 | i2_LQ_HNbud_c38363/f1p24/2751 | i2_HQ_HNbud_c18579/f2p3/2307 | i1_LQ_HNbud_c39445/f6p24/2000 |
| i1_LQ_HNbud_c151860/f1p6/1935 | i2_HQ_HNbud_c1215/f4p4/2695 | i1_LQ_HNbud_c205668/f1p4/1327 | i1_HQ_HNbud_c29661/f6p4/1500 |
| i0_HQ_HNbud_c97825/f13p0/879 | i0_LQ_HNbud_c49559/f1p0/841 | i2_HQ_HNbud_c2137/f5p0/2881 | i1_HQ_HNbud_c217125/f17p5/1158 |
| i0_HQ_HNbud_c1852/f5p3/921 | i1_LQ_HNbud_c217355/f13p3/1162 | i2_HQ_HNbud_c70620/f5p18/2148 | i1_LQ_HNbud_c253129/f1p6/1507 |
| i0_HQ_HNbud_c1800/f3p0/792 | i1_HQ_HNbud_c88827/f4p6/1538 | i1_LQ_HNbud_c269319/f3p10/1041 | i1_LQ_HNbud_c171011/f1p2/1997 |
| i1_LQ_HNbud_c263140/f1p2/1026 | i2_LQ_HNbud_c45520/f1p3/2044 | i1_LQ_HNbud_c18751/f1p5/1606 | i0_LQ_HNbud_c53152/f1p10/788 |
| i1_HQ_HNbud_c6929/f2p1/1624 | i2_LQ_HNbud_c27798/f1p14/2318 | i1_LQ_HNbud_c106245/f1p3/1990 | i2_LQ_HNbud_c27186/f1p0/2178 |
| i1_LQ_HNbud_c177399/f1p2/1556 | i1_HQ_HNbud_c121650/f4p2/1131 | i1_HQ_HNbud_c37636/f2p0/1803 | i1_LQ_HNbud_c231623/f1p1/1757 |
| i4_LQ_HNbud_c2895/f1p0/4803 | i2_LQ_HNbud_c51590/f1p0/2102 | i0_LQ_HNbud_c46656/f1p0/728 | i1_LQ_HNbud_c203972/f1p7/1370 |
| i2_HQ_HNbud_c17908/f5p2/2195 | i0_HQ_HNbud_c11006/f2p5/796 | i1_HQ_HNbud_c126201/f3p4/1633 | i0_HQ_HNbud_c849/f10p0/888 |
| i3_LQ_HNbud_c9017/f1p1/3703 | i1_LQ_HNbud_c78640/f1p0/1069 | i3_LQ_HNbud_c16530/f1p0/3067 | i2_LQ_HNbud_c18477/f1p2/2312 |
| i1_LQ_HNbud_c5687/f1p5/1620 | i1_HQ_HNbud_c264885/f3p1/1599 | i0_HQ_HNbud_c1638/f12p0/647 | i0_LQ_HNbud_c30805/f1p1/933 |
| i1_HQ_HNbud_c245513/f10p9/1763 | i2_LQ_HNbud_c59048/f1p0/2900 | i0_LQ_HNbud_c18776/f2p0/775 | i2_LQ_HNbud_c4666/f1p9/2770 |
| i1_LQ_HNbud_c253597/f1p9/1253 | i1_LQ_HNbud_c55139/f1p8/1727 | i2_LQ_HNbud_c51283/f1p0/2423 | i1_LQ_HNbud_c68306/f1p14/1742 |
| i4_LQ_HNbud_c2949/f1p1/4479 | i0_LQ_HNbud_c5517/f4p0/624 | i0_HQ_HNbud_c18740/f4p0/628 | i0_HQ_HNbud_c64640/f13p0/988 |
| i2_HQ_HNbud_c16607/f15p1/2294 | i2_LQ_HNbud_c14308/f1p2/2272 | i1_LQ_HNbud_c67106/f1p5/1146 | i1_LQ_HNbud_c131687/f1p5/1313 |
| i1_LQ_HNbud_c200309/f1p1/1724 | i1_LQ_HNbud_c171657/f1p5/1297 | i2_HQ_HNbud_c70446/f4p4/2076 | i1_LQ_HNbud_c51214/f1p0/1256 |
| i1_LQ_HNbud_c53339/f1p6/1461 | i1_LQ_HNbud_c3408/f1p3/1137 | i2_LQ_HNbud_c26822/f1p7/2679 | i0_HQ_HNbud_c1956/f5p8/749 |
| i1_HQ_HNbud_c195039/f5p6/1485 | i2_LQ_HNbud_c53236/f1p191/2318 | i1_LQ_HNbud_c130085/f1p3/1528 | i1_LQ_HNbud_c22606/f1p6/1141 |
| i1_HQ_HNbud_c48962/f2p0/1654 | i0_HQ_HNbud_c13774/f2p2/727 | i1_LQ_HNbud_c221269/f1p3/1909 | i3_LQ_HNbud_c9915/f1p11/3306 |
| i0_LQ_HNbud_c13417/f2p4/615 | i1_HQ_HNbud_c49328/f2p3/1317 | i2_HQ_HNbud_c51925/f2p10/2719 | i1_LQ_HNbud_c21144/f1p2/1458 |
| i1_HQ_HNbud_c243070/f4p11/1548 | i1_LQ_HNbud_c107775/f1p2/1937 | i1_HQ_HNbud_c37260/f18p38/1791 | i1_HQ_HNbud_c55810/f3p0/1286 |
| i2_LQ_HNbud_c26827/f1p2/2376 | i1_HQ_HNbud_c22662/f3p1/1746 | i3_LQ_HNbud_c7456/f1p0/3865 | i1_LQ_HNbud_c202284/f1p15/1246 |
| i2_HQ_HNbud_c14437/f4p3/2273 | i1_LQ_HNbud_c36695/f1p2/1811 | i1_HQ_HNbud_c20101/f5p3/1801 | i1_LQ_HNbud_c199991/f1p1/1495 |
| i1_LQ_HNbud_c159492/f2p2/1012 | i0_LQ_HNbud_c54437/f1p8/906 | i1_LQ_HNbud_c180784/f1p1/1876 | i1_LQ_HNbud_c68729/f1p1/1264 |
| i1_LQ_HNbud_c213843/f1p9/1816 | i0_LQ_HNbud_c77749/f1p0/934 | i0_HQ_HNbud_c65115/f18p0/894 | i2_LQ_HNbud_c5793/f1p3/2970 |
| i1_LQ_HNbud_c229022/f1p0/1408 | i2_LQ_HNbud_c53244/f1p5/2872 | i1_LQ_HNbud_c125530/f1p1/1798 | i1_LQ_HNbud_c73922/f1p1/1892 |
| i0_LQ_HNbud_c50054/f1p4/686 | i0_LQ_HNbud_c42578/f1p7/463 | i1_LQ_HNbud_c91928/f1p1/1394 | i0_LQ_HNbud_c27917/f1p1/641 |
| i1_LQ_HNbud_c69017/f1p7/1794 | i1_LQ_HNbud_c44356/f1p0/1072 | i4_LQ_HNbud_c4286/f1p0/4042 | i2_LQ_HNbud_c12775/f1p16/2640 |
| i1_LQ_HNbud_c9340/f1p6/1759 | i1_LQ_HNbud_c49411/f1p8/1531 | i2_LQ_HNbud_c62026/f1p39/2048 | i2_LQ_HNbud_c2800/f1p2/2338 |
| i2_LQ_HNbud_c34068/f1p0/2244 | i0_LQ_HNbud_c54565/f1p0/701 | i0_LQ_HNbud_c21047/f1p1/896 | i1_LQ_HNbud_c130675/f1p5/1699 |
| i1_LQ_HNbud_c172590/f1p2/1534 | i1_HQ_HNbud_c34301/f4p1/1834 | i3_HQ_HNbud_c1322/f3p3/3237 | i2_LQ_HNbud_c63449/f1p3/1772 |
| i2_LQ_HNbud_c62144/f1p1/2016 | i1_LQ_HNbud_c225598/f1p3/1252 | i1_LQ_HNbud_c57241/f1p4/1831 | i1_LQ_HNbud_c231872/f1p6/1256 |
| i1_HQ_HNbud_c244553/f59p18/1844 | i0_LQ_HNbud_c78540/f1p0/649 | i1_LQ_HNbud_c56529/f1p2/1539 | i1_HQ_HNbud_c2106/f19p5/1439 |
| i1_LQ_HNbud_c37909/f1p3/1207 | i1_LQ_HNbud_c89832/f1p3/1568 | i1_LQ_HNbud_c35101/f2p0/1795 | i1_LQ_HNbud_c173367/f1p4/1294 |
| i1_HQ_HNbud_c27951/f4p0/1290 | i2_LQ_HNbud_c22715/f1p0/2369 | i3_LQ_HNbud_c17656/f1p3/3103 | i3_LQ_HNbud_c13537/f1p1/3407 |
| i3_LQ_HNbud_c13594/f1p1/3070 | i1_LQ_HNbud_c174656/f1p2/1931 | i1_LQ_HNbud_c226369/f1p5/1280 | i1_LQ_HNbud_c132192/f1p3/1933 |
| i1_LQ_HNbud_c200885/f1p1/1924 | i1_LQ_HNbud_c38599/f1p1/1740 | i2_LQ_HNbud_c60309/f1p1/2759 | i1_HQ_HNbud_c33273/f2p2/1228 |
| i2_HQ_HNbud_c3950/f2p0/2133 | i3_LQ_HNbud_c15071/f1p0/3314 | i1_LQ_HNbud_c38924/f2p3/1252 | i2_LQ_HNbud_c15180/f1p2/2057 |
| i2_HQ_HNbud_c1145/f11p0/2635 | i0_HQ_HNbud_c30912/f3p1/540 | i1_HQ_HNbud_c40766/f3p3/1359 | i1_HQ_HNbud_c41450/f4p1/1138 |
| i2_LQ_HNbud_c55620/f1p3/2906 | i0_LQ_HNbud_c54474/f1p11/773 | i1_LQ_HNbud_c104751/f1p0/1122 | i5_LQ_HNbud_c1393/f1p0/5243 |
| i0_LQ_HNbud_c83718/f1p0/999 | i1_HQ_HNbud_c104589/f2p0/1788 | i1_LQ_HNbud_c110826/f1p1/1257 | i2_LQ_HNbud_c52061/f1p2/2620 |
| i1_LQ_HNbud_c2078/f14p6/1910 | i1_LQ_HNbud_c264079/f10p10/1359 | i1_HQ_HNbud_c69467/f3p4/1723 | i1_LQ_HNbud_c203636/f1p2/1674 |
| i0_HQ_HNbud_c26243/f3p6/798 | i4_LQ_HNbud_c3659/f1p4/4362 | i0_HQ_HNbud_c11308/f3p2/882 | i1_LQ_HNbud_c127840/f1p12/1359 |
| i2_LQ_HNbud_c5825/f1p2/2617 | i2_LQ_HNbud_c50110/f1p10/2931 | i2_LQ_HNbud_c59246/f1p0/2859 | i3_LQ_HNbud_c15881/f1p1/3053 |
| i1_LQ_HNbud_c152708/f1p3/1582 | i1_LQ_HNbud_c75603/f2p3/2077 | i1_HQ_HNbud_c41599/f2p28/2022 | i1_LQ_HNbud_c223507/f1p0/1121 |
| i1_LQ_HNbud_c175296/f1p1/1267 | i3_LQ_HNbud_c7179/f1p2/3849 | i2_LQ_HNbud_c51643/f1p7/2585 | i1_LQ_HNbud_c248127/f1p3/1408 |
| i2_LQ_HNbud_c60165/f1p6/2919 | i1_HQ_HNbud_c18562/f5p2/1231 | i1_LQ_HNbud_c68494/f1p1/1438 | i1_LQ_HNbud_c58489/f1p11/1524 |
| i2_LQ_HNbud_c6748/f1p0/2340 | i1_HQ_HNbud_c144356/f19p11/1729 | i1_LQ_HNbud_c51966/f1p2/1437 | i1_LQ_HNbud_c61365/f1p2/1080 |
| i0_HQ_HNbud_c12037/f3p0/620 | i1_LQ_HNbud_c87716/f1p2/1499 | i1_HQ_HNbud_c218401/f8p8/1542 | i1_HQ_HNbud_c93145/f2p4/1503 |
| i2_LQ_HNbud_c28846/f1p1/2648 | i1_LQ_HNbud_c18905/f1p3/1551 | i0_LQ_HNbud_c23524/f1p2/710 | i2_LQ_HNbud_c8210/f1p1/2319 |
| i1_LQ_HNbud_c74875/f1p3/1749 | i0_HQ_HNbud_c27679/f4p11/769 | i1_LQ_HNbud_c56043/f1p1/1220 | i2_LQ_HNbud_c39159/f1p6/2932 |
| i1_LQ_HNbud_c177194/f1p5/1296 | i1_LQ_HNbud_c250160/f1p3/1303 | i2_HQ_HNbud_c1805/f4p5/2652 | i0_HQ_HNbud_c9757/f2p3/859 |
| i1_LQ_HNbud_c78027/f1p1/1067 | i2_LQ_HNbud_c11844/f1p0/2186 | i1_LQ_HNbud_c204575/f1p1/1399 | i1_LQ_HNbud_c173248/f1p1/1956 |
| i1_LQ_HNbud_c227258/f1p2/1408 | i1_LQ_HNbud_c171985/f1p1/1224 | i1_HQ_HNbud_c56379/f3p1/1525 | i2_LQ_HNbud_c6633/f1p1/2699 |
| i1_LQ_HNbud_c42063/f1p10/1511 | i3_LQ_HNbud_c13823/f1p4/3454 | i1_LQ_HNbud_c130397/f2p0/1251 | i2_LQ_HNbud_c62594/f1p1/2015 |
| i3_LQ_HNbud_c11393/f1p0/3465 | i0_LQ_HNbud_c20426/f1p2/893 | i1_LQ_HNbud_c131956/f1p3/1959 | i1_HQ_HNbud_c267309/f3p4/1272 |
| i1_LQ_HNbud_c18074/f1p1/1592 | i1_LQ_HNbud_c156827/f1p7/1356 | i1_HQ_HNbud_c11137/f5p3/1387 | i1_LQ_HNbud_c18994/f1p0/1234 |
| i1_LQ_HNbud_c40764/f1p3/1725 | i1_LQ_HNbud_c15796/f1p3/1060 | i1_LQ_HNbud_c280421/f1p2/1023 | i1_LQ_HNbud_c253627/f1p37/1792 |
| i1_LQ_HNbud_c36854/f1p0/1797 | i0_HQ_HNbud_c12967/f10p0/513 | i1_LQ_HNbud_c131010/f1p2/1684 | i2_LQ_HNbud_c36018/f1p3/2768 |
| i4_LQ_HNbud_c2480/f1p0/4450 | i2_HQ_HNbud_c66889/f2p5/2211 | i1_HQ_HNbud_c49875/f4p5/1824 | i3_LQ_HNbud_c6744/f1p0/3612 |
| i2_LQ_HNbud_c42722/f1p7/2882 | i0_LQ_HNbud_c31539/f2p0/932 | i2_HQ_HNbud_c47494/f10p2/2187 | i2_LQ_HNbud_c55091/f1p6/2983 |
| i3_HQ_HNbud_c18085/f13p3/3992 | i1_LQ_HNbud_c25131/f1p3/1703 | i1_HQ_HNbud_c230164/f2p3/1614 | i2_LQ_HNbud_c54387/f1p23/2708 |
| i6_LQ_HNbud_c543/f1p0/6620 | i0_LQ_HNbud_c12365/f1p5/735 | i1_LQ_HNbud_c117682/f1p9/1065 | i1_LQ_HNbud_c67984/f2p1/1439 |
| i2_LQ_HNbud_c59720/f1p2/2137 | i0_LQ_HNbud_c71474/f1p5/438 | i1_LQ_HNbud_c149868/f1p6/1437 | i1_LQ_HNbud_c1851/f2p0/1882 |
| i0_LQ_HNbud_c53751/f1p8/956 | i1_LQ_HNbud_c149578/f1p3/1958 | i2_HQ_HNbud_c70291/f23p9/2261 | i0_LQ_HNbud_c40858/f1p0/854 |
| i1_HQ_HNbud_c1914/f3p3/1360 | i0_LQ_HNbud_c7037/f1p4/527 | i1_HQ_HNbud_c149849/f3p2/1292 | i1_LQ_HNbud_c133701/f1p3/1889 |
| i2_LQ_HNbud_c53915/f1p3/2940 | i0_LQ_HNbud_c23762/f1p1/667 | i6_LQ_HNbud_c2809/f1p0/6905 | i2_LQ_HNbud_c34587/f1p0/2630 |
| i1_LQ_HNbud_c92697/f1p8/1827 | i1_HQ_HNbud_c191316/f9p0/1263 | i0_HQ_HNbud_c2245/f4p1/917 | i1_LQ_HNbud_c230337/f1p1/1293 |
| i4_LQ_HNbud_c4234/f1p0/4076 | i2_LQ_HNbud_c43510/f1p1/2411 | i2_HQ_HNbud_c22285/f3p0/2336 | i4_LQ_HNbud_c4090/f1p5/4071 |
| i1_LQ_HNbud_c148590/f1p1/1259 | i2_LQ_HNbud_c12782/f2p4/2171 | i1_LQ_HNbud_c264715/f92p17/1732 | i2_LQ_HNbud_c63820/f1p16/1902 |
| i3_LQ_HNbud_c10807/f1p5/3086 | i1_HQ_HNbud_c2381/f23p8/1388 | i2_HQ_HNbud_c7406/f3p0/2318 | i0_LQ_HNbud_c50787/f1p0/558 |
| i1_HQ_HNbud_c41376/f2p4/1402 | i1_HQ_HNbud_c32469/f4p0/1598 | i1_LQ_HNbud_c226607/f1p1/1630 | i1_LQ_HNbud_c195494/f1p2/1337 |
| i1_LQ_HNbud_c202448/f1p2/1801 | i1_LQ_HNbud_c196371/f1p0/1166 | i1_LQ_HNbud_c267373/f1p7/1039 | i2_HQ_HNbud_c45258/f2p3/2036 |
| i1_LQ_HNbud_c5262/f1p8/1131 | i3_LQ_HNbud_c10350/f1p0/3261 | i5_LQ_HNbud_c1770/f1p2/5077 | i0_LQ_HNbud_c29100/f1p0/839 |
| i1_LQ_HNbud_c58036/f1p16/1849 | i1_HQ_HNbud_c192613/f22p6/1114 | i1_LQ_HNbud_c227477/f1p1/1415 | i2_LQ_HNbud_c9921/f1p0/2667 |
| i1_HQ_HNbud_c49971/f4p6/1724 | i0_LQ_HNbud_c77155/f1p0/829 | i1_HQ_HNbud_c1856/f5p3/1490 | i1_LQ_HNbud_c229359/f1p1/1838 |
| i0_HQ_HNbud_c6531/f2p6/752 | i2_LQ_HNbud_c64038/f1p2/2033 | i2_LQ_HNbud_c56874/f1p10/2095 | i0_LQ_HNbud_c67521/f1p0/911 |
| i1_LQ_HNbud_c67237/f1p1/1891 | i2_LQ_HNbud_c52762/f1p3/2700 | i4_LQ_HNbud_c1696/f1p0/4681 | i2_LQ_HNbud_c59427/f1p0/2146 |
| i1_LQ_HNbud_c34746/f2p2/1369 | i1_LQ_HNbud_c196644/f1p0/1340 | i1_HQ_HNbud_c37846/f8p3/1825 | i2_LQ_HNbud_c61628/f2p0/2077 |
| i1_LQ_HNbud_c67666/f1p3/1556 | i1_LQ_HNbud_c196223/f1p0/1837 | i1_HQ_HNbud_c10551/f4p1/1379 | i1_LQ_HNbud_c90026/f1p18/1259 |
| i1_HQ_HNbud_c65992/f2p2/1411 | i1_HQ_HNbud_c49623/f2p6/1173 | i1_HQ_HNbud_c65957/f2p7/1720 | i1_HQ_HNbud_c81331/f5p3/1838 |
| i2_LQ_HNbud_c21288/f1p4/2139 | i2_LQ_HNbud_c24431/f1p2/2802 | i1_HQ_HNbud_c176377/f2p5/1602 | i1_HQ_HNbud_c9287/f3p6/1273 |
| i1_LQ_HNbud_c109722/f1p15/1629 | i0_LQ_HNbud_c41504/f1p12/555 | i0_LQ_HNbud_c40226/f1p0/767 | i0_LQ_HNbud_c97195/f1p0/721 |
| i1_HQ_HNbud_c26875/f3p0/1997 | i0_HQ_HNbud_c98265/f22p0/766 | i1_LQ_HNbud_c27267/f1p5/919 | i1_LQ_HNbud_c72187/f1p2/1181 |
| i1_LQ_HNbud_c279670/f1p2/1039 | i2_LQ_HNbud_c19724/f1p7/2901 | i0_LQ_HNbud_c74170/f1p0/497 | i0_LQ_HNbud_c23057/f1p0/582 |
| i1_HQ_HNbud_c22548/f2p1/1142 | i1_HQ_HNbud_c26293/f9p2/1258 | i2_LQ_HNbud_c16084/f1p0/2025 | i2_LQ_HNbud_c50999/f1p0/2200 |
| i2_LQ_HNbud_c35883/f1p31/2475 | i0_LQ_HNbud_c43003/f1p1/634 | i1_HQ_HNbud_c1501/f17p3/1449 | i1_HQ_HNbud_c49495/f5p1/1565 |
| i4_LQ_HNbud_c2418/f1p12/4997 | i1_LQ_HNbud_c95835/f2p0/1041 | i2_HQ_HNbud_c70897/f2p1/2012 | i1_LQ_HNbud_c3704/f1p12/1926 |
| i1_LQ_HNbud_c74177/f1p132/1844 | i1_HQ_HNbud_c92031/f3p0/1536 | i2_LQ_HNbud_c53231/f1p2/2243 | i2_LQ_HNbud_c36054/f1p0/2835 |
| i4_LQ_HNbud_c1593/f1p5/4623 | i1_HQ_HNbud_c13569/f5p7/1190 | i1_LQ_HNbud_c128223/f1p2/1993 | i1_LQ_HNbud_c105854/f1p1/1115 |
| i0_LQ_HNbud_c46211/f1p8/697 | i1_LQ_HNbud_c253169/f1p11/1584 | i1_HQ_HNbud_c115440/f2p1/1053 | i1_HQ_HNbud_c94604/f4p5/1014 |
| i1_LQ_HNbud_c146462/f1p1/1510 | i1_LQ_HNbud_c51895/f1p15/1702 | i1_HQ_HNbud_c265109/f7p0/1160 | i1_LQ_HNbud_c151834/f1p0/1722 |
| i1_LQ_HNbud_c86794/f1p10/1305 | i2_LQ_HNbud_c54172/f1p4/2096 | i1_HQ_HNbud_c191983/f19p3/1466 | i1_LQ_HNbud_c151092/f1p2/1269 |
| i1_LQ_HNbud_c104681/f1p8/1909 | i1_LQ_HNbud_c125555/f1p4/1783 | i1_LQ_HNbud_c196053/f1p0/1285 | i3_LQ_HNbud_c12735/f1p1/3059 |
| i1_LQ_HNbud_c54601/f1p2/1650 | i0_LQ_HNbud_c19358/f1p4/771 | i1_HQ_HNbud_c170393/f3p4/1304 | i1_LQ_HNbud_c29271/f1p26/1058 |
| i0_LQ_HNbud_c32035/f1p0/828 | i2_HQ_HNbud_c13589/f2p19/2762 | i0_LQ_HNbud_c8111/f1p4/624 | i1_LQ_HNbud_c115887/f1p0/1039 |
| i0_LQ_HNbud_c8158/f1p0/973 | i1_LQ_HNbud_c74991/f1p2/1811 | i2_LQ_HNbud_c21669/f1p18/2807 | i2_LQ_HNbud_c50912/f1p1/2165 |
| i3_LQ_HNbud_c17578/f1p0/3192 | i1_LQ_HNbud_c73668/f1p1/1919 | i2_HQ_HNbud_c13640/f8p4/2713 | i2_HQ_HNbud_c40835/f4p1/2468 |
| i1_LQ_HNbud_c159512/f3p1/1104 | i1_HQ_HNbud_c167661/f2p0/1548 | i0_LQ_HNbud_c75224/f1p0/959 | i1_HQ_HNbud_c32598/f5p3/1154 |
| i1_LQ_HNbud_c14350/f1p0/1264 | i1_LQ_HNbud_c58164/f1p2/1488 | i5_LQ_HNbud_c604/f1p0/5628 | i0_LQ_HNbud_c39854/f2p2/796 |
| i0_LQ_HNbud_c48528/f1p1/710 | i2_LQ_HNbud_c41438/f1p11/2487 | i1_HQ_HNbud_c77433/f2p2/1028 | i1_LQ_HNbud_c160272/f1p0/1797 |
| i1_LQ_HNbud_c153168/f1p1/1528 | i1_LQ_HNbud_c155979/f1p3/1393 | i1_LQ_HNbud_c89548/f2p3/1940 | i1_LQ_HNbud_c120556/f1p3/1108 |
| i1_LQ_HNbud_c150753/f1p7/1877 | i1_HQ_HNbud_c254370/f10p5/1078 | i1_LQ_HNbud_c17978/f1p0/1235 | i0_LQ_HNbud_c29606/f1p0/747 |
| i1_LQ_HNbud_c187541/f1p0/1023 | i2_LQ_HNbud_c59523/f1p6/2055 | i0_LQ_HNbud_c79832/f1p0/1000 | i4_LQ_HNbud_c548/f1p0/4702 |
| i1_LQ_HNbud_c5585/f1p3/1686 | i2_LQ_HNbud_c24038/f1p1/2092 | i3_HQ_HNbud_c16843/f5p0/3160 | i1_HQ_HNbud_c17438/f3p4/1704 |
| i3_LQ_HNbud_c12893/f1p3/3810 | i1_LQ_HNbud_c174291/f1p1/1564 | i3_LQ_HNbud_c15303/f1p3/3019 | i0_LQ_HNbud_c13958/f1p0/857 |
| i1_HQ_HNbud_c151955/f3p2/1736 | i1_HQ_HNbud_c75552/f2p1/1508 | i1_LQ_HNbud_c163287/f1p9/1029 | i2_LQ_HNbud_c15282/f1p2/2067 |
| i1_LQ_HNbud_c57533/f1p8/1216 | i1_HQ_HNbud_c3511/f11p6/1312 | i2_LQ_HNbud_c43655/f1p23/2256 | i1_LQ_HNbud_c216360/f17p5/1560 |
| i2_LQ_HNbud_c62172/f1p1/2036 | i1_HQ_HNbud_c44246/f3p5/1290 | i1_HQ_HNbud_c216463/f7p2/1367 | i1_LQ_HNbud_c177354/f1p0/1731 |
| i1_LQ_HNbud_c5014/f1p6/1603 | i1_LQ_HNbud_c116615/f1p2/1014 | i1_LQ_HNbud_c104766/f1p3/1563 | i0_LQ_HNbud_c23089/f1p0/894 |
| i1_LQ_HNbud_c229564/f1p0/1476 | i2_LQ_HNbud_c3982/f1p5/2566 | i1_HQ_HNbud_c242615/f10p4/1792 | i0_LQ_HNbud_c31659/f1p0/716 |
| i1_LQ_HNbud_c17127/f33p5/1580 | i1_HQ_HNbud_c17606/f7p1/1294 | i1_LQ_HNbud_c50243/f1p5/1259 | i1_LQ_HNbud_c72649/f1p1/1496 |
| i0_LQ_HNbud_c10183/f2p5/748 | i1_HQ_HNbud_c51800/f2p0/1513 | i1_HQ_HNbud_c244438/f14p12/1829 | i1_LQ_HNbud_c90792/f1p6/1886 |
| i0_LQ_HNbud_c3684/f1p3/851 | i1_HQ_HNbud_c167885/f4p3/1576 | i0_HQ_HNbud_c1533/f4p0/551 | i1_LQ_HNbud_c202606/f1p0/1350 |
| i1_LQ_HNbud_c85604/f1p3/1639 | i2_LQ_HNbud_c9845/f1p0/2271 | i3_LQ_HNbud_c11865/f1p7/3328 | i1_LQ_HNbud_c37022/f1p3/1470 |
| i1_LQ_HNbud_c196255/f1p11/1595 | i3_LQ_HNbud_c16433/f1p0/3028 | i0_HQ_HNbud_c8486/f3p2/989 | i4_LQ_HNbud_c1973/f1p0/5103 |
| i1_LQ_HNbud_c126487/f1p7/1814 | i1_HQ_HNbud_c25706/f7p2/1290 | i3_HQ_HNbud_c1614/f2p20/3305 | i1_HQ_HNbud_c49290/f2p4/1779 |
| i2_LQ_HNbud_c16001/f1p7/2049 | i2_LQ_HNbud_c24168/f1p50/2506 | i2_LQ_HNbud_c10684/f1p5/2227 | i0_HQ_HNbud_c50458/f2p0/445 |
| i0_LQ_HNbud_c31751/f1p0/667 | i2_LQ_HNbud_c54551/f1p0/2356 | i0_HQ_HNbud_c98094/f37p0/860 | i0_LQ_HNbud_c78356/f1p0/891 |
| i1_LQ_HNbud_c106821/f1p3/1553 | i1_HQ_HNbud_c100663/f57p15/1654 | i1_LQ_HNbud_c106867/f1p2/1988 | i0_LQ_HNbud_c70954/f1p9/837 |
| i0_LQ_HNbud_c55093/f1p2/786 | i2_LQ_HNbud_c22973/f1p2/2717 | i2_HQ_HNbud_c2005/f4p0/2403 | i1_HQ_HNbud_c19960/f4p0/1875 |
| i1_LQ_HNbud_c130879/f1p3/1675 | i2_LQ_HNbud_c4872/f1p16/2181 | i1_LQ_HNbud_c20264/f1p0/1966 | i0_LQ_HNbud_c11639/f1p0/740 |
| i2_LQ_HNbud_c26242/f1p2/2101 | i1_LQ_HNbud_c148871/f1p0/1414 | i1_HQ_HNbud_c193299/f7p0/1076 | i1_LQ_HNbud_c179698/f1p0/1386 |
| i0_LQ_HNbud_c20955/f1p0/779 | i1_LQ_HNbud_c22513/f1p3/1606 | i1_LQ_HNbud_c4783/f1p2/2045 | i2_LQ_HNbud_c15446/f1p12/2033 |
| i1_LQ_HNbud_c126016/f1p0/1805 | i1_LQ_HNbud_c175135/f1p0/1121 | i1_LQ_HNbud_c6820/f1p0/1562 | i2_LQ_HNbud_c14152/f1p5/2644 |
| i2_LQ_HNbud_c2615/f1p1/2617 | i2_LQ_HNbud_c38625/f1p7/2594 | i5_LQ_HNbud_c479/f1p0/5235 | i1_HQ_HNbud_c191148/f6p6/1320 |
| i0_LQ_HNbud_c83517/f1p0/972 | i1_LQ_HNbud_c25641/f2p0/1429 | i2_LQ_HNbud_c6273/f1p2/2242 | i1_LQ_HNbud_c88999/f1p5/1623 |
| i1_LQ_HNbud_c60122/f1p1/1030 | i1_LQ_HNbud_c112745/f1p1/1489 | i1_LQ_HNbud_c173693/f1p0/1254 | i1_LQ_HNbud_c235336/f1p3/1087 |
| i1_LQ_HNbud_c41253/f1p2/1402 | i1_HQ_HNbud_c142754/f19p9/1217 | i1_LQ_HNbud_c74012/f1p3/1270 | i1_LQ_HNbud_c175419/f1p0/1768 |
| i2_LQ_HNbud_c4456/f1p0/2641 | i1_LQ_HNbud_c57076/f1p5/1631 | i1_LQ_HNbud_c106443/f1p2/1335 | i1_LQ_HNbud_c21941/f1p3/1777 |
| i2_LQ_HNbud_c58010/f1p0/2445 | i1_LQ_HNbud_c65620/f2p4/1881 | i1_HQ_HNbud_c18364/f8p4/1656 | i1_HQ_HNbud_c263207/f6p2/1209 |
| i1_LQ_HNbud_c171236/f1p0/1485 | i2_LQ_HNbud_c43872/f1p3/2195 | i2_LQ_HNbud_c60079/f1p1/2620 | i3_LQ_HNbud_c10083/f1p10/3727 |
| i1_HQ_HNbud_c32435/f5p0/1386 | i1_LQ_HNbud_c228515/f1p8/1530 | i1_HQ_HNbud_c180480/f2p14/1650 | i0_LQ_HNbud_c27748/f1p7/545 |
| i2_LQ_HNbud_c13997/f1p0/2215 | i0_LQ_HNbud_c81145/f1p0/993 | i1_LQ_HNbud_c54869/f1p3/1525 | i1_LQ_HNbud_c70357/f1p4/1898 |
| i1_LQ_HNbud_c111682/f1p9/1643 | i1_LQ_HNbud_c279307/f1p17/922 | i2_LQ_HNbud_c10835/f2p1/2918 | i1_LQ_HNbud_c37781/f1p2/1143 |
| i1_LQ_HNbud_c66245/f1p3/1629 | i1_LQ_HNbud_c10598/f1p2/1144 | i1_HQ_HNbud_c242846/f5p0/1426 | i1_LQ_HNbud_c89225/f1p0/1144 |
| i1_LQ_HNbud_c278384/f1p0/1004 | i0_LQ_HNbud_c5529/f1p3/658 | i1_LQ_HNbud_c20277/f1p1/1368 | i1_LQ_HNbud_c103996/f1p1/1560 |
| i4_LQ_HNbud_c813/f1p0/4794 | i1_LQ_HNbud_c56957/f1p2/1416 | i1_HQ_HNbud_c150490/f4p6/1608 | i3_LQ_HNbud_c14656/f1p0/3548 |
| i2_LQ_HNbud_c35129/f1p2/2803 | i1_HQ_HNbud_c1612/f21p9/1607 | i1_LQ_HNbud_c68057/f1p48/2060 | i1_LQ_HNbud_c5490/f1p10/1958 |
| i1_LQ_HNbud_c136670/f1p1/1561 | i2_LQ_HNbud_c34981/f1p6/2439 | i2_HQ_HNbud_c1684/f5p0/2443 | i3_LQ_HNbud_c6879/f1p154/3320 |
| i2_LQ_HNbud_c3674/f1p10/2758 | i1_HQ_HNbud_c55108/f2p2/1287 | i1_LQ_HNbud_c153534/f1p8/1484 | i1_LQ_HNbud_c191636/f142p29/1183 |
| i1_LQ_HNbud_c103790/f1p3/1513 | i2_LQ_HNbud_c25459/f1p1/2935 | i0_HQ_HNbud_c39585/f2p6/462 | i1_LQ_HNbud_c107948/f1p0/1953 |
| i0_LQ_HNbud_c53643/f1p0/748 | i1_LQ_HNbud_c67466/f1p7/1807 | i2_HQ_HNbud_c17661/f6p5/2438 | i1_LQ_HNbud_c231297/f1p3/1584 |
| i2_LQ_HNbud_c23820/f1p3/2103 | i4_LQ_HNbud_c3887/f1p3/4667 | i0_LQ_HNbud_c68386/f1p2/926 | i1_LQ_HNbud_c90226/f1p5/1246 |
| i1_LQ_HNbud_c87680/f1p2/1370 | i1_LQ_HNbud_c51719/f1p0/1540 | i0_LQ_HNbud_c45574/f1p0/875 | i1_LQ_HNbud_c34072/f1p1/1505 |
| i0_HQ_HNbud_c1844/f8p2/903 | i0_LQ_HNbud_c81216/f1p0/749 | i1_HQ_HNbud_c225081/f4p10/1819 | i2_LQ_HNbud_c20539/f1p1/2425 |
| i1_LQ_HNbud_c241688/f1p0/1080 | i1_LQ_HNbud_c68396/f1p3/1802 | i0_LQ_HNbud_c71855/f1p20/875 | i1_LQ_HNbud_c277774/f1p4/1020 |
| i1_HQ_HNbud_c183118/f4p2/1083 | i1_LQ_HNbud_c253047/f1p0/1457 | i1_HQ_HNbud_c265690/f5p1/1186 | i4_LQ_HNbud_c360/f1p0/4973 |
| i0_LQ_HNbud_c26762/f1p2/522 | i1_LQ_HNbud_c171307/f1p9/1403 | i2_LQ_HNbud_c24849/f1p1/2083 | i1_HQ_HNbud_c4617/f2p3/1143 |
| i2_LQ_HNbud_c31510/f1p5/2033 | i2_LQ_HNbud_c34392/f1p7/2248 | i1_HQ_HNbud_c516/f38p4/1792 | i1_LQ_HNbud_c40190/f1p0/1300 |
| i0_LQ_HNbud_c43725/f1p10/919 | i1_HQ_HNbud_c217106/f4p2/1458 | i1_LQ_HNbud_c224369/f1p0/1554 | i0_LQ_HNbud_c26955/f1p703/699 |
| i3_LQ_HNbud_c9109/f1p1/3737 | i0_LQ_HNbud_c25229/f1p4/983 | i1_HQ_HNbud_c58025/f3p3/1689 | i2_LQ_HNbud_c21330/f1p2/2807 |
| i1_LQ_HNbud_c89686/f1p10/1634 | i1_LQ_HNbud_c9644/f1p1/1286 | i2_LQ_HNbud_c19306/f1p1/2497 | i2_LQ_HNbud_c34355/f1p0/2411 |
| i3_HQ_HNbud_c760/f7p0/3337 | i1_LQ_HNbud_c200834/f1p10/1437 | i0_LQ_HNbud_c21769/f1p3/631 | i1_LQ_HNbud_c252072/f1p12/1415 |
| i1_LQ_HNbud_c271122/f1p0/1230 | i2_LQ_HNbud_c51752/f1p6/2481 | i0_LQ_HNbud_c21876/f1p2/489 | i2_HQ_HNbud_c71549/f3p7/2130 |
| i0_LQ_HNbud_c78165/f1p1/526 | i1_LQ_HNbud_c131796/f1p2/1650 | i1_LQ_HNbud_c201906/f1p1/1419 | i1_LQ_HNbud_c7336/f1p10/1160 |
| i0_LQ_HNbud_c73604/f1p6/589 | i1_LQ_HNbud_c230155/f1p6/1358 | i0_LQ_HNbud_c80840/f1p0/449 | i2_LQ_HNbud_c54705/f1p9/2204 |
| i1_HQ_HNbud_c166615/f4p7/1402 | i4_LQ_HNbud_c2387/f1p1/4603 | i1_LQ_HNbud_c216798/f3p4/1092 | i3_LQ_HNbud_c5264/f1p14/3885 |
| i2_LQ_HNbud_c52539/f1p1/2098 | i1_LQ_HNbud_c9574/f1p0/1275 | i2_LQ_HNbud_c39635/f1p23/2432 | i1_LQ_HNbud_c28859/f1p7/1055 |
| i5_LQ_HNbud_c1084/f1p0/5415 | i1_HQ_HNbud_c138614/f3p10/1113 | i2_LQ_HNbud_c58518/f1p1/2686 | i1_LQ_HNbud_c230331/f1p11/1607 |
| i2_LQ_HNbud_c12741/f1p7/2771 | i1_LQ_HNbud_c80440/f1p8/1030 | i1_LQ_HNbud_c203305/f1p3/1312 | i1_HQ_HNbud_c24501/f2p3/1540 |
| i2_LQ_HNbud_c2499/f1p9/2705 | i2_LQ_HNbud_c59692/f1p2/2098 | i0_LQ_HNbud_c21181/f1p10/781 | i2_LQ_HNbud_c30984/f1p17/2040 |
| i1_LQ_HNbud_c35588/f2p0/1221 | i1_LQ_HNbud_c277756/f1p1/1039 | i1_LQ_HNbud_c204002/f1p1/1185 | i1_LQ_HNbud_c278884/f1p0/1051 |
| i1_LQ_HNbud_c156147/f1p5/1679 | i0_LQ_HNbud_c28097/f1p3/947 | i0_HQ_HNbud_c25634/f3p0/745 | i2_HQ_HNbud_c49191/f3p1/2333 |
| i1_LQ_HNbud_c225841/f1p1/1470 | i1_HQ_HNbud_c4597/f14p3/1165 | i2_LQ_HNbud_c33576/f2p2/2348 | i1_LQ_HNbud_c18261/f3p9/1654 |
| i1_HQ_HNbud_c10359/f6p3/1574 | i1_HQ_HNbud_c26764/f2p4/1791 | i1_HQ_HNbud_c48745/f2p5/1764 | i1_HQ_HNbud_c264924/f13p11/1568 |
| i2_LQ_HNbud_c50433/f1p322/2697 | i1_HQ_HNbud_c38778/f2p5/1870 | i1_LQ_HNbud_c5119/f1p2/1971 | i1_HQ_HNbud_c216402/f9p0/1554 |
| i1_HQ_HNbud_c17626/f4p1/1437 | i3_HQ_HNbud_c1612/f2p8/3551 | i2_LQ_HNbud_c53993/f1p5/2569 | i1_LQ_HNbud_c20852/f1p12/1598 |
| i1_LQ_HNbud_c86007/f1p38/1854 | i1_LQ_HNbud_c106920/f1p10/1632 | i1_HQ_HNbud_c109963/f5p5/1669 | i2_LQ_HNbud_c38594/f1p28/1782 |
| i3_LQ_HNbud_c2633/f1p2/3643 | i1_LQ_HNbud_c277566/f1p0/1000 | i1_LQ_HNbud_c197658/f1p1/1547 | i1_LQ_HNbud_c34212/f2p2/1579 |
| i1_LQ_HNbud_c162044/f1p0/1086 | i1_LQ_HNbud_c177356/f1p4/2158 | i1_HQ_HNbud_c109172/f2p1/1388 | i1_LQ_HNbud_c53683/f1p5/1438 |
| i0_HQ_HNbud_c64663/f15p5/827 | i1_LQ_HNbud_c278064/f1p0/1001 | i2_LQ_HNbud_c56771/f1p5/2824 | i1_LQ_HNbud_c163358/f1p8/1060 |
| i1_LQ_HNbud_c21725/f1p1/1227 | i1_LQ_HNbud_c155944/f1p6/1483 | i1_HQ_HNbud_c224957/f2p1/1953 | i1_LQ_HNbud_c37116/f11p5/1923 |
| i0_HQ_HNbud_c1787/f9p1/767 | i1_LQ_HNbud_c228629/f1p7/1692 | i1_LQ_HNbud_c9341/f1p2/1148 | i5_LQ_HNbud_c1825/f1p2/5024 |
| i1_LQ_HNbud_c175399/f1p1/1190 | i7_HQ_HNbud_c83/f2p0/7417 | i1_LQ_HNbud_c9475/f2p1/1384 | i1_LQ_HNbud_c190866/f3p2/1307 |
| i1_LQ_HNbud_c227772/f1p12/1730 | i1_LQ_HNbud_c39766/f1p16/1968 | i1_LQ_HNbud_c54875/f1p12/1850 | i2_LQ_HNbud_c4142/f1p12/2532 |
| i1_HQ_HNbud_c245409/f3p6/1065 | i1_LQ_HNbud_c66736/f1p4/1233 | i1_HQ_HNbud_c2709/f3p1/1201 | i1_LQ_HNbud_c25036/f1p1/1638 |
| i1_LQ_HNbud_c147765/f1p9/1507 | i2_HQ_HNbud_c65776/f2p10/2101 | i1_LQ_HNbud_c71688/f1p4/1544 | i1_LQ_HNbud_c279950/f1p3/1044 |
| i1_LQ_HNbud_c4120/f1p3/1501 | i1_LQ_HNbud_c10407/f1p18/1435 | i1_LQ_HNbud_c147781/f1p1/1611 | i1_LQ_HNbud_c133287/f1p3/1906 |
| i1_LQ_HNbud_c148855/f1p10/1899 | i1_LQ_HNbud_c152400/f1p1/1254 | i1_LQ_HNbud_c34231/f1p2/1631 | i0_LQ_HNbud_c24559/f1p8/778 |
| i0_LQ_HNbud_c22491/f1p5/959 | i0_HQ_HNbud_c64638/f21p3/710 | i0_LQ_HNbud_c22085/f1p124/752 | i2_LQ_HNbud_c30782/f1p0/2027 |
| i1_HQ_HNbud_c6179/f7p2/1438 | i2_LQ_HNbud_c13166/f1p3/2485 | i0_HQ_HNbud_c8076/f2p6/852 | i1_LQ_HNbud_c54643/f1p1/1530 |
| i1_LQ_HNbud_c174706/f1p0/1849 | i1_LQ_HNbud_c170962/f1p3/1911 | i2_LQ_HNbud_c41583/f1p0/2726 | i4_LQ_HNbud_c1535/f1p0/4469 |
| i0_LQ_HNbud_c26982/f1p0/455 | i1_HQ_HNbud_c246048/f8p10/1202 | i1_LQ_HNbud_c55833/f1p3/1182 | i1_HQ_HNbud_c256039/f2p6/1068 |
| i1_HQ_HNbud_c111866/f2p3/1926 | i1_LQ_HNbud_c105280/f1p1/1397 | i3_LQ_HNbud_c11054/f1p0/3324 | i1_LQ_HNbud_c130991/f1p13/1174 |
| i1_HQ_HNbud_c103259/f6p1/1567 | i0_HQ_HNbud_c85748/f6p3/653 | i2_LQ_HNbud_c62366/f1p1/2014 | i1_LQ_HNbud_c5653/f1p1/1699 |
| i1_LQ_HNbud_c229671/f1p8/1264 | i1_HQ_HNbud_c66279/f5p1/2000 | i1_LQ_HNbud_c187347/f1p5/1075 | i3_LQ_HNbud_c11309/f1p3/3751 |
| i2_LQ_HNbud_c23693/f1p1133/2840 | i0_HQ_HNbud_c10217/f3p0/915 | i2_HQ_HNbud_c17708/f4p2/2170 | i1_LQ_HNbud_c89810/f1p9/1313 |
| i4_LQ_HNbud_c1761/f1p12/4647 | i1_HQ_HNbud_c247097/f2p12/1974 | i1_LQ_HNbud_c174181/f1p4/1877 | i1_LQ_HNbud_c249576/f1p8/1934 |
| i2_HQ_HNbud_c18140/f3p1/2354 | i1_HQ_HNbud_c85723/f3p6/1623 | i1_HQ_HNbud_c107794/f3p4/1588 | i1_LQ_HNbud_c2821/f1p1/1309 |
| i1_HQ_HNbud_c265231/f50p10/1596 | i1_LQ_HNbud_c108665/f1p0/1572 | i2_LQ_HNbud_c36212/f1p8/2399 | i1_LQ_HNbud_c19042/f1p6/1136 |
| i1_HQ_HNbud_c265411/f10p2/1073 | i0_LQ_HNbud_c80255/f1p0/766 | i2_LQ_HNbud_c19559/f1p8/2743 | i1_HQ_HNbud_c38990/f2p11/1485 |
| i1_HQ_HNbud_c62874/f19p5/1516 | i1_LQ_HNbud_c83323/f37p21/1280 | i1_LQ_HNbud_c279492/f1p7/1018 | i1_LQ_HNbud_c278528/f1p20/1002 |
| i2_LQ_HNbud_c15292/f1p5/2094 | i1_LQ_HNbud_c132418/f1p0/1108 | i1_LQ_HNbud_c109042/f1p11/1653 | i1_LQ_HNbud_c9134/f8p5/1905 |
| i2_LQ_HNbud_c43401/f1p1/2178 | i1_HQ_HNbud_c183105/f4p4/1027 | i1_LQ_HNbud_c222930/f1p4/1656 | i2_HQ_HNbud_c16636/f14p4/2576 |
| i2_LQ_HNbud_c50542/f1p3/2903 | i1_HQ_HNbud_c2599/f12p3/1725 | i1_HQ_HNbud_c264980/f6p6/1106 | i1_LQ_HNbud_c133447/f1p14/1124 |
| i1_LQ_HNbud_c249037/f1p0/1700 | i1_HQ_HNbud_c264293/f5p0/1195 | i0_HQ_HNbud_c19311/f2p1/697 | i1_LQ_HNbud_c153650/f1p2/1791 |
| i1_LQ_HNbud_c10857/f1p11/1776 | i1_LQ_HNbud_c224954/f1p4/1263 | i2_LQ_HNbud_c39625/f1p1/2086 | i0_LQ_HNbud_c72328/f1p156/923 |
| i1_HQ_HNbud_c35430/f2p3/1552 | i1_LQ_HNbud_c21504/f1p0/1520 | i0_LQ_HNbud_c11440/f1p1/957 | i0_HQ_HNbud_c2309/f5p5/788 |
| i2_LQ_HNbud_c7325/f1p2/2373 | i1_LQ_HNbud_c116574/f1p19/1300 | i0_LQ_HNbud_c26277/f1p6/656 | i1_LQ_HNbud_c55013/f1p17/1231 |
| i1_HQ_HNbud_c5233/f3p1/1155 | i2_LQ_HNbud_c19907/f1p6/2622 | i1_LQ_HNbud_c100423/f1p1/1216 | i2_LQ_HNbud_c23371/f1p2/2953 |
| i2_HQ_HNbud_c65572/f27p6/2470 | i2_LQ_HNbud_c6928/f1p7/2702 | i2_LQ_HNbud_c58717/f1p27/2149 | i1_HQ_HNbud_c34018/f3p3/1424 |
| i1_LQ_HNbud_c108386/f1p0/1634 | i1_LQ_HNbud_c152337/f1p4/1618 | i2_LQ_HNbud_c35778/f1p0/2755 | i0_HQ_HNbud_c46834/f2p6/475 |
| i1_LQ_HNbud_c108954/f1p23/1862 | i2_HQ_HNbud_c66392/f5p1/2356 | i1_LQ_HNbud_c57624/f1p2/1663 | i1_LQ_HNbud_c200447/f1p5/1162 |

**TableS2. KEGG pathway enrichment of DEGs in floral formation of tree peony.**

| Term | #KEGG pathway | ID | Input number | Background number | P-Value | Corrected P-Value | | | Enrichment Factor | Input gene |
| --- | --- | --- | --- | --- | --- | --- | --- | --- | --- | --- |
| S1_S2_up | DNA replication | ko03030 | 64 | 322 | 4.60E-35 | 4.88E-33 | 1.99E+01 | i2_LQ_HNbud_c56488/f1p20/3105\|i2_HQ_HNbud_c1181/f31p18/2198\|i2_HQ_HNbud_c64519/f36p1/2780\|i2_LQ_HNbud_c7224/f1p7/2795\|i1_LQ_HNbud_c222540/f1p2/1625\|i2_LQ_HNbud_c42090/f1p2/2517\|i0_HQ_HNbud_c12393/f2p0/670\|i3_HQ_HNbud_c1735/f2p1/3229\|i1_LQ_HNbud_c129292/f1p2/1189\|i1_LQ_HNbud_c20054/f1p2/1132\|i2_LQ_HNbud_c52550/f1p1/2900\|i2_LQ_HNbud_c66904/f1p1/2775\|i3_LQ_HNbud_c16568/f1p58/3022\|i2_HQ_HNbud_c65838/f12p10/2556\|i1_LQ_HNbud_c39169/f1p1/1684\|i6_LQ_HNbud_c436/f1p0/6819\|i3_LQ_HNbud_c10626/f1p5/3487\|i0_LQ_HNbud_c41401/f1p2/720\|i2_LQ_HNbud_c47151/f1p4/2062\|i1_LQ_HNbud_c105775/f1p16/1408\|i2_HQ_HNbud_c70509/f2p2/2528\|i1_HQ_HNbud_c159627/f2p1/1510\|i1_LQ_HNbud_c204117/f1p16/1274\|i3_LQ_HNbud_c10197/f1p6/3167\|i1_HQ_HNbud_c88501/f3p2/1574\|i4_HQ_HNbud_c4367/f6p1/4832\|i2_HQ_HNbud_c19772/f3p0/2817\|i2_HQ_HNbud_c66463/f2p2/2849\|i2_LQ_HNbud_c4487/f1p3/2920\|i2_HQ_HNbud_c1673/f3p3/2271\|i2_LQ_HNbud_c71566/f1p0/2011\|i2_LQ_HNbud_c39590/f1p2/2616\|i2_HQ_HNbud_c70893/f4p0/2051\|i2_LQ_HNbud_c27141/f1p1/2978\|i1_LQ_HNbud_c203685/f1p17/1773\|i1_HQ_HNbud_c65705/f2p5/2009\|i3_LQ_HNbud_c6619/f1p0/3769\|i1_LQ_HNbud_c134006/f1p3/1628\|i3_LQ_HNbud_c18604/f1p0/3002\|i2_HQ_HNbud_c64526/f14p1/2827\|i1_LQ_HNbud_c106324/f1p11/1134\|i1_LQ_HNbud_c74007/f1p3/1815\|i1_HQ_HNbud_c23225/f2p2/1823\|i1_LQ_HNbud_c34692/f1p3/1561\|i1_LQ_HNbud_c109815/f2p2/1152\|i2_LQ_HNbud_c8614/f1p3/2351\|i2_LQ_HNbud_c3349/f1p1/2567\|i3_LQ_HNbud_c12803/f1p15/3159\|i2_HQ_HNbud_c70396/f3p3/2576\|i2_LQ_HNbud_c28503/f1p5/2860\|i4_LQ_HNbud_c708/f1p0/4482\|i1_LQ_HNbud_c108368/f1p4/1609\|i1_LQ_HNbud_c264776/f119p16/1206\|i1_LQ_HNbud_c241444/f1p14/1072\|i2_HQ_HNbud_c8051/f2p4/2794\|i1_LQ_HNbud_c93344/f1p3/1655\|i1_LQ_HNbud_c12422/f1p2/1711\|i1_HQ_HNbud_c7251/f4p1/1564\|i0_HQ_HNbud_c2396/f2p6/698\|i1_LQ_HNbud_c269024/f1p16/1184\|i3_LQ_HNbud_c10344/f1p2/3415\|i2_LQ_HNbud_c43501/f1p5/2570\|i4_LQ_HNbud_c2989/f1p9/4673\|i2_HQ_HNbud_c65037/f36p7/2570 | | |
| S1_S2_up | Mismatch repair | ko03430 | 27 | 189 | 1.67E-12 | 8.87E-11 | 1.43E+01 | i2_HQ_HNbud_c1181/f31p18/2198\|i0_HQ_HNbud_c12393/f2p0/670\|i1_LQ_HNbud_c129292/f1p2/1189\|i1_LQ_HNbud_c105775/f1p16/1408\|i0_LQ_HNbud_c41401/f1p2/720\|i2_HQ_HNbud_c1673/f3p3/2271\|i1_LQ_HNbud_c204117/f1p16/1274\|i2_HQ_HNbud_c19772/f3p0/2817\|i3_LQ_HNbud_c9469/f1p11/3124\|i2_LQ_HNbud_c73580/f1p1/1966\|i1_HQ_HNbud_c65705/f2p5/2009\|i1_LQ_HNbud_c134006/f1p3/1628\|i3_LQ_HNbud_c11999/f1p2/3376\|i1_LQ_HNbud_c176120/f1p2/1941\|i1_LQ_HNbud_c74007/f1p3/1815\|i1_LQ_HNbud_c34692/f1p3/1561\|i1_LQ_HNbud_c109815/f2p2/1152\|i2_LQ_HNbud_c3349/f1p1/2567\|i1_LQ_HNbud_c106324/f1p11/1134\|i1_LQ_HNbud_c264776/f119p16/1206\|i2_HQ_HNbud_c8051/f2p4/2794\|i1_LQ_HNbud_c93344/f1p3/1655\|i2_LQ_HNbud_c39590/f1p2/2616\|i1_LQ_HNbud_c269024/f1p16/1184\|i4_LQ_HNbud_c2989/f1p9/4673\|i1_LQ_HNbud_c52241/f1p0/1589\|i3_LQ_HNbud_c10797/f1p0/3318 | | |
| S1_S2_up | Pyrimidine metabolism | ko00240 | 49 | 730 | 5.57E-10 | 1.97E-08 | 6.71E+00 | i1_HQ_HNbud_c146570/f3p2/1398\|i1_LQ_HNbud_c222540/f1p2/1625\|i0_LQ_HNbud_c42845/f1p0/898\|i0_HQ_HNbud_c6933/f6p2/989\|i2_LQ_HNbud_c26046/f1p7/2858\|i0_HQ_HNbud_c99580/f2p0/901\|i1_LQ_HNbud_c203443/f1p5/1278\|i0_LQ_HNbud_c79785/f1p0/679\|i1_HQ_HNbud_c88501/f3p2/1574\|i1_HQ_HNbud_c220457/f4p8/1280\|i0_HQ_HNbud_c66677/f2p0/797\|i1_HQ_HNbud_c7884/f3p2/1194\|i1_LQ_HNbud_c39169/f1p1/1684\|i6_LQ_HNbud_c436/f1p0/6819\|i3_LQ_HNbud_c10626/f1p5/3487\|i0_LQ_HNbud_c41401/f1p2/720\|i1_HQ_HNbud_c23225/f2p2/1823\|i1_HQ_HNbud_c159627/f2p1/1510\|i0_LQ_HNbud_c99631/f1p0/839\|i2_LQ_HNbud_c12346/f1p7/2438\|i1_HQ_HNbud_c192055/f32p8/1376\|i1_LQ_HNbud_c71451/f4p4/1500\|i2_LQ_HNbud_c71566/f1p0/2011\|i2_LQ_HNbud_c70898/f2p0/2071\|i2_HQ_HNbud_c70893/f4p0/2051\|i3_LQ_HNbud_c10344/f1p2/3415\|i1_HQ_HNbud_c65705/f2p5/2009\|i2_LQ_HNbud_c57277/f1p7/2731\|i3_LQ_HNbud_c6619/f1p0/3769\|i1_LQ_HNbud_c134006/f1p3/1628\|i3_LQ_HNbud_c4159/f1p3/3452\|i0_HQ_HNbud_c39475/f3p0/580\|i1_LQ_HNbud_c74007/f1p3/1815\|i1_LQ_HNbud_c111603/f1p4/1629\|i1_LQ_HNbud_c34692/f1p3/1561\|i4_HQ_HNbud_c4367/f6p1/4832\|i2_LQ_HNbud_c8614/f1p3/2351\|i1_LQ_HNbud_c27646/f1p2/1824\|i1_LQ_HNbud_c140401/f1p2/1082\|i0_HQ_HNbud_c97833/f7p0/791\|i2_LQ_HNbud_c50888/f1p1/2441\|i1_LQ_HNbud_c108677/f1p2/1601\|i1_LQ_HNbud_c93344/f1p3/1655\|i1_LQ_HNbud_c89498/f1p1/1237\|i1_LQ_HNbud_c12422/f1p2/1711\|i1_HQ_HNbud_c7251/f4p1/1564\|i0_HQ_HNbud_c2396/f2p6/698\|i1_HQ_HNbud_c74681/f4p5/1322\|i2_LQ_HNbud_c6394/f1p7/2641 | | |
| S1_S2_up | Base excision repair | ko03410 | 22 | 231 | 1.57E-07 | 4.16E-06 | 9.52E+00 | i1_HQ_HNbud_c18128/f2p1/1817\|i6_LQ_HNbud_c436/f1p0/6819\|i0_LQ_HNbud_c41401/f1p2/720\|i1_LQ_HNbud_c204117/f1p16/1274\|i2_HQ_HNbud_c19772/f3p0/2817\|i2_HQ_HNbud_c65153/f5p2/2080\|i1_HQ_HNbud_c65705/f2p5/2009\|i3_LQ_HNbud_c6619/f1p0/3769\|i1_LQ_HNbud_c134006/f1p3/1628\|i1_LQ_HNbud_c74007/f1p3/1815\|i1_HQ_HNbud_c23225/f2p2/1823\|i1_LQ_HNbud_c34692/f1p3/1561\|i2_LQ_HNbud_c8614/f1p3/2351\|i1_LQ_HNbud_c106324/f1p11/1134\|i1_LQ_HNbud_c108368/f1p4/1609\|i1_LQ_HNbud_c264776/f119p16/1206\|i1_LQ_HNbud_c105775/f1p16/1408\|i1_LQ_HNbud_c93344/f1p3/1655\|i1_LQ_HNbud_c12422/f1p2/1711\|i0_HQ_HNbud_c2396/f2p6/698\|i1_LQ_HNbud_c269024/f1p16/1184\|i4_LQ_HNbud_c2989/f1p9/4673 | | |
| S1_S2_up | Nucleotide excision repair | ko03420 | 27 | 362 | 6.30E-07 | 1.34E-05 | 7.46E+00 | i2_HQ_HNbud_c1181/f31p18/2198\|i0_HQ_HNbud_c12393/f2p0/670\|i1_LQ_HNbud_c129292/f1p2/1189\|i6_LQ_HNbud_c436/f1p0/6819\|i0_LQ_HNbud_c41401/f1p2/720\|i2_HQ_HNbud_c1673/f3p3/2271\|i1_LQ_HNbud_c204117/f1p16/1274\|i2_HQ_HNbud_c19772/f3p0/2817\|i1_HQ_HNbud_c65705/f2p5/2009\|i3_LQ_HNbud_c6619/f1p0/3769\|i1_LQ_HNbud_c134006/f1p3/1628\|i1_LQ_HNbud_c74007/f1p3/1815\|i1_HQ_HNbud_c23225/f2p2/1823\|i1_LQ_HNbud_c34692/f1p3/1561\|i1_LQ_HNbud_c109815/f2p2/1152\|i2_LQ_HNbud_c8614/f1p3/2351\|i2_LQ_HNbud_c3349/f1p1/2567\|i1_LQ_HNbud_c106324/f1p11/1134\|i1_LQ_HNbud_c264776/f119p16/1206\|i2_HQ_HNbud_c8051/f2p4/2794\|i1_LQ_HNbud_c93344/f1p3/1655\|i1_LQ_HNbud_c12422/f1p2/1711\|i2_LQ_HNbud_c39590/f1p2/2616\|i0_HQ_HNbud_c2396/f2p6/698\|i1_LQ_HNbud_c269024/f1p16/1184\|i1_LQ_HNbud_c105775/f1p16/1408\|i4_LQ_HNbud_c2989/f1p9/4673 | | |
| S1_S2_up | Homologous recombination | ko03440 | 19 | 197 | 9.08E-07 | 1.60E-05 | 9.64E+00 | i1_LQ_HNbud_c109815/f2p2/1152\|i2_HQ_HNbud_c1181/f31p18/2198\|i2_LQ_HNbud_c3349/f1p1/2567\|i0_LQ_HNbud_c41401/f1p2/720\|i4_HQ_HNbud_c231/f2p0/4130\|i0_HQ_HNbud_c12393/f2p0/670\|i1_HQ_HNbud_c65705/f2p5/2009\|i1_LQ_HNbud_c67192/f1p1/1312\|i2_HQ_HNbud_c8051/f2p4/2794\|i2_HQ_HNbud_c1673/f3p3/2271\|i1_LQ_HNbud_c134006/f1p3/1628\|i1_LQ_HNbud_c54170/f1p0/1937\|i2_LQ_HNbud_c39590/f1p2/2616\|i1_LQ_HNbud_c129292/f1p2/1189\|i2_LQ_HNbud_c22659/f1p0/2056\|i4_LQ_HNbud_c2769/f1p1/4641\|i1_LQ_HNbud_c74007/f1p3/1815\|i1_LQ_HNbud_c93344/f1p3/1655\|i1_LQ_HNbud_c34692/f1p3/1561 | | |
| S1_S2_up | Purine metabolism | ko00230 | 44 | 975 | 8.53E-05 | 0.001291512 | 4.51E+00 | i1_LQ_HNbud_c222540/f1p2/1625\|i1_LQ_HNbud_c155762/f1p1/1370\|i2_LQ_HNbud_c26046/f1p7/2858\|i0_HQ_HNbud_c99580/f2p0/901\|i1_LQ_HNbud_c150807/f1p23/1399\|i1_LQ_HNbud_c203443/f1p5/1278\|i0_LQ_HNbud_c79785/f1p0/679\|i1_HQ_HNbud_c220457/f4p8/1280\|i1_LQ_HNbud_c42039/f2p18/1340\|i1_LQ_HNbud_c39169/f1p1/1684\|i6_LQ_HNbud_c436/f1p0/6819\|i3_LQ_HNbud_c10626/f1p5/3487\|i0_LQ_HNbud_c41401/f1p2/720\|i1_HQ_HNbud_c23225/f2p2/1823\|i1_HQ_HNbud_c159627/f2p1/1510\|i1_LQ_HNbud_c174365/f1p3/1293\|i2_HQ_HNbud_c19518/f5p1/2204\|i2_HQ_HNbud_c61668/f2p2/2036\|i1_HQ_HNbud_c88501/f3p2/1574\|i1_HQ_HNbud_c192055/f32p8/1376\|i2_LQ_HNbud_c38142/f1p3/2097\|i2_LQ_HNbud_c71566/f1p0/2011\|i2_LQ_HNbud_c70898/f2p0/2071\|i2_HQ_HNbud_c70893/f4p0/2051\|i1_LQ_HNbud_c50271/f2p18/1312\|i3_LQ_HNbud_c10344/f1p2/3415\|i1_HQ_HNbud_c65705/f2p5/2009\|i2_LQ_HNbud_c57277/f1p7/2731\|i3_LQ_HNbud_c6619/f1p0/3769\|i1_LQ_HNbud_c134006/f1p3/1628\|i1_LQ_HNbud_c67903/f1p1/1751\|i3_LQ_HNbud_c4159/f1p3/3452\|i1_LQ_HNbud_c98700/f1p1/1060\|i1_LQ_HNbud_c74007/f1p3/1815\|i1_LQ_HNbud_c111603/f1p4/1629\|i1_LQ_HNbud_c34692/f1p3/1561\|i4_HQ_HNbud_c4367/f6p1/4832\|i2_LQ_HNbud_c8614/f1p3/2351\|i1_LQ_HNbud_c93344/f1p3/1655\|i1_LQ_HNbud_c12422/f1p2/1711\|i1_HQ_HNbud_c7251/f4p1/1564\|i0_HQ_HNbud_c2396/f2p6/698\|i1_HQ_HNbud_c74681/f4p5/1322\|i2_LQ_HNbud_c6394/f1p7/2641 | | |
| S1_S2_up | Pentose and glucuronate interconversions | ko00040 | 21 | 338 | 0.000128 | 0.001698142 | 6.21E+00 | i1_LQ_HNbud_c152465/f1p6/1915\|i1_HQ_HNbud_c48286/f3p4/1191\|i2_LQ_HNbud_c27385/f1p0/2340\|i2_LQ_HNbud_c40561/f1p12/2072\|i2_LQ_HNbud_c30279/f1p13/1975\|i1_LQ_HNbud_c231937/f1p1/1791\|i2_HQ_HNbud_c41932/f2p6/2137\|i1_HQ_HNbud_c25375/f8p0/1491\|i1_HQ_HNbud_c151229/f2p10/1741\|i2_LQ_HNbud_c62913/f1p2/2020\|i1_HQ_HNbud_c217358/f13p18/1935\|i1_LQ_HNbud_c251113/f1p1/1281\|i1_LQ_HNbud_c7500/f1p0/1768\|i1_LQ_HNbud_c108999/f1p0/1349\|i1_LQ_HNbud_c17893/f1p18/1916\|i2_LQ_HNbud_c62944/f1p2/2044\|i2_LQ_HNbud_c15874/f1p2/2174\|i2_HQ_HNbud_c34080/f2p0/2201\|i1_LQ_HNbud_c249088/f1p0/1753\|i2_HQ_HNbud_c47568/f5p0/2245\|i1_HQ_HNbud_c100247/f7p2/1637 | | |
| S1_S2_up | Cysteine and methionine metabolism | ko00270 | 35 | 747 | 0.000226 | 0.002661248 | 4.69E+00 | i0_LQ_HNbud_c41629/f1p2/932\|i2_LQ_HNbud_c34594/f1p5/2457\|i3_LQ_HNbud_c8499/f1p1/3085\|i1_LQ_HNbud_c23342/f1p20/1685\|i4_LQ_HNbud_c3322/f1p0/4959\|i3_LQ_HNbud_c2361/f1p3/3238\|i2_HQ_HNbud_c18272/f4p2/2844\|i1_HQ_HNbud_c25966/f4p9/1428\|i4_HQ_HNbud_c51/f6p0/5036\|i1_LQ_HNbud_c197155/f1p5/1913\|i1_HQ_HNbud_c65613/f7p0/1292\|i1_LQ_HNbud_c196991/f1p6/1545\|i1_LQ_HNbud_c205936/f1p0/1609\|i1_LQ_HNbud_c247382/f1p0/1377\|i1_LQ_HNbud_c9645/f1p34/1581\|i1_HQ_HNbud_c1881/f42p27/1838\|i1_HQ_HNbud_c627/f34p27/1706\|i1_LQ_HNbud_c130686/f1p27/1699\|i3_LQ_HNbud_c13324/f1p0/3983\|i2_HQ_HNbud_c831/f10p3/2956\|i1_LQ_HNbud_c199566/f1p33/1879\|i3_LQ_HNbud_c2683/f1p0/3326\|i4_LQ_HNbud_c1987/f1p3/4213\|i1_HQ_HNbud_c93301/f2p9/1475\|i1_HQ_HNbud_c132399/f2p42/1160\|i3_LQ_HNbud_c11737/f1p2/3884\|i1_HQ_HNbud_c81227/f14p9/1594\|i1_LQ_HNbud_c170377/f1p32/1820\|i1_LQ_HNbud_c228084/f1p22/1389\|i1_LQ_HNbud_c177068/f1p7/1700\|i1_HQ_HNbud_c242706/f6p0/1904\|i2_LQ_HNbud_c53814/f1p11/2086\|i1_HQ_HNbud_c187229/f2p0/1484\|i1_LQ_HNbud_c18746/f1p18/1784\|i1_LQ_HNbud_c129690/f1p16/1470 | | |
| S1_S2_up | Phagosome | ko04145 | 28 | 554 | 0.0003 | 0.003180827 | 5.05E+00 | i0_LQ_HNbud_c82095/f1p0/942\|i1_LQ_HNbud_c204278/f1p22/1721\|i1_HQ_HNbud_c265122/f127p32/1731\|i1_LQ_HNbud_c38367/f1p12/1662\|i1_HQ_HNbud_c267789/f2p18/1674\|i1_LQ_HNbud_c242130/f1p0/1094\|i1_LQ_HNbud_c227827/f1p6/1826\|i1_LQ_HNbud_c116421/f1p8/1115\|i1_HQ_HNbud_c53984/f4p9/1871\|i1_LQ_HNbud_c23193/f1p15/1671\|i1_LQ_HNbud_c133141/f1p23/1803\|i1_HQ_HNbud_c3253/f14p0/1742\|i1_HQ_HNbud_c265134/f5p8/1238\|i1_LQ_HNbud_c25196/f1p4/1728\|i1_LQ_HNbud_c133180/f1p110/1693\|i1_LQ_HNbud_c20256/f1p8/1698\|i1_LQ_HNbud_c40083/f1p7/1663\|i1_LQ_HNbud_c109371/f1p23/1806\|i1_LQ_HNbud_c204418/f1p3/1724\|i1_LQ_HNbud_c198922/f1p31/1689\|i1_HQ_HNbud_c264754/f104p24/1739\|i1_LQ_HNbud_c175250/f1p8/1697\|i1_LQ_HNbud_c23754/f1p17/1735\|i1_HQ_HNbud_c17835/f20p10/1761\|i1_LQ_HNbud_c14681/f1p1/1085\|i1_HQ_HNbud_c1309/f36p9/1643\|i0_LQ_HNbud_c11193/f2p5/902\|i1_LQ_HNbud_c172723/f1p0/1472 | | |
| S1_S2_up | Terpenoid backbone biosynthesis | ko00900 | 22 | 393 | 0.000356 | 0.003428078 | 5.60E+00 | i2_HQ_HNbud_c34757/f3p5/2617\|i1_LQ_HNbud_c227058/f1p22/1948\|i1_LQ_HNbud_c225398/f1p3/1418\|i2_LQ_HNbud_c51155/f1p1/2974\|i0_LQ_HNbud_c26411/f1p0/844\|i1_LQ_HNbud_c213751/f1p6/1011\|i1_LQ_HNbud_c151123/f1p3/1669\|i1_LQ_HNbud_c91506/f1p24/1990\|i2_HQ_HNbud_c1664/f4p8/2300\|i1_LQ_HNbud_c226688/f1p2/1846\|i2_LQ_HNbud_c46529/f1p0/2047\|i2_HQ_HNbud_c66247/f21p8/2621\|i0_LQ_HNbud_c30122/f1p2/928\|i1_LQ_HNbud_c19043/f1p3/1530\|i2_LQ_HNbud_c59656/f1p5/2594\|i2_LQ_HNbud_c56649/f1p4/2763\|i2_HQ_HNbud_c19242/f3p1/2263\|i1_LQ_HNbud_c87346/f1p24/1534\|i1_LQ_HNbud_c178036/f1p15/1897\|i1_LQ_HNbud_c88413/f1p3/1139\|i1_HQ_HNbud_c219658/f172p4/1672\|i1_LQ_HNbud_c10654/f1p23/1280 | | |
| S1_S2_up | Non-homologous end-joining | ko03450 | 6 | 49 | 0.001738 | 0.015348167 | 1.22E+01 | i1_LQ_HNbud_c119289/f1p0/1044\|i2_LQ_HNbud_c12642/f1p5/2171\|i1_LQ_HNbud_c108368/f1p4/1609\|i1_HQ_HNbud_c17679/f4p1/1687\|i1_LQ_HNbud_c226850/f1p1/1621\|i2_LQ_HNbud_c67537/f1p0/2213 | | |
| S1_S2_up | Lysine degradation | ko00310 | 12 | 199 | 0.004215 | 0.034370898 | 6.03E+00 | i1_LQ_HNbud_c90460/f1p4/1716\|i2_LQ_HNbud_c7877/f1p1/3020\|i1_LQ_HNbud_c231937/f1p1/1791\|i1_HQ_HNbud_c151229/f2p10/1741\|i2_LQ_HNbud_c40561/f1p12/2072\|i2_LQ_HNbud_c25823/f1p1/2388\|i1_LQ_HNbud_c251113/f1p1/1281\|i2_LQ_HNbud_c30279/f1p13/1975\|i1_LQ_HNbud_c50888/f1p0/1519\|i2_LQ_HNbud_c7758/f1p1/2798\|i2_LQ_HNbud_c37628/f1p6/2552\|i2_HQ_HNbud_c34080/f2p0/2201 | | |
| S1_S2_up | Circadian rhythm - plant | ko04712 | 14 | 264 | 0.006134 | 0.043349425 | 5.30E+00 | i2_LQ_HNbud_c26920/f1p17/2901\|i1_LQ_HNbud_c249768/f1p6/1518\|i2_LQ_HNbud_c53369/f1p11/2426\|i1_HQ_HNbud_c24406/f3p6/1370\|i1_LQ_HNbud_c112913/f1p5/1244\|i2_LQ_HNbud_c35178/f2p3/2529\|i3_LQ_HNbud_c15287/f1p1/3031\|i2_HQ_HNbud_c27866/f2p1/3004\|i2_LQ_HNbud_c72626/f1p11/2775\|i3_LQ_HNbud_c8223/f1p3/3488\|i2_LQ_HNbud_c41573/f1p0/2271\|i2_LQ_HNbud_c28794/f1p1/2850\|i2_LQ_HNbud_c23772/f1p5/2950\|i2_LQ_HNbud_c1725/f3p0/2269 | | |
| S1_S2_up | Arginine and proline metabolism | ko00330 | 17 | 348 | 0.005871 | 0.043349425 | 4.89E+00 | i1_LQ_HNbud_c130686/f1p27/1699\|i1_LQ_HNbud_c170377/f1p32/1820\|i1_LQ_HNbud_c18746/f1p18/1784\|i2_LQ_HNbud_c30279/f1p13/1975\|i1_LQ_HNbud_c231937/f1p1/1791\|i1_LQ_HNbud_c177068/f1p7/1700\|i1_HQ_HNbud_c151229/f2p10/1741\|i2_LQ_HNbud_c40561/f1p12/2072\|i1_LQ_HNbud_c199566/f1p33/1879\|i1_LQ_HNbud_c23342/f1p20/1685\|i1_LQ_HNbud_c251113/f1p1/1281\|i2_LQ_HNbud_c53814/f1p11/2086\|i1_HQ_HNbud_c38319/f7p6/1866\|i1_HQ_HNbud_c1881/f42p27/1838\|i2_HQ_HNbud_c34080/f2p0/2201\|i3_LQ_HNbud_c5688/f1p4/3260\|i1_HQ_HNbud_c627/f34p27/1706 | | |
| S1_S2_up | Limonene and pinene degradation | ko00903 | 6 | 66 | 0.006716 | 0.044492682 | 9.09E+00 | i1_LQ_HNbud_c231937/f1p1/1791\|i2_LQ_HNbud_c30279/f1p13/1975\|i1_HQ_HNbud_c151229/f2p10/1741\|i2_LQ_HNbud_c40561/f1p12/2072\|i1_LQ_HNbud_c251113/f1p1/1281\|i2_HQ_HNbud_c34080/f2p0/2201 | | |
| S1_S2_up | Flavonoid biosynthesis | ko00941 | 7 | 90 | 0.007701 | 0.048017386 | 7.78E+00 | i1_LQ_HNbud_c249768/f1p6/1518\|i1_LQ_HNbud_c89704/f1p13/1357\|i2_HQ_HNbud_c18063/f4p8/2286\|i1_HQ_HNbud_c191018/f23p19/1607\|i1_LQ_HNbud_c149147/f1p0/1558\|i1_HQ_HNbud_c25202/f6p17/1185\|i1_HQ_HNbud_c24406/f3p6/1370 | | |
| S1_S2_down | Protein processing in endoplasmic reticulum | ko04141 | 67 | 1811 | 1.31E-31 | 7.33E-30 | 3.70E+00 | i1_HQ_HNbud_c220992/f2p30/1744\|i0_LQ_HNbud_c22602/f1p7/981\|i1_HQ_HNbud_c16322/f2p11/1127\|i2_HQ_HNbud_c20344/f3p12/2478\|i1_HQ_HNbud_c16210/f27p26/1793\|i1_LQ_HNbud_c155489/f1p8/1274\|i1_LQ_HNbud_c278370/f1p26/1014\|i1_LQ_HNbud_c214019/f1p19/901\|i0_LQ_HNbud_c28549/f1p1/796\|i1_LQ_HNbud_c11182/f1p7/1644\|i0_LQ_HNbud_c9667/f1p0/879\|i0_LQ_HNbud_c74044/f1p0/810\|i0_LQ_HNbud_c41102/f1p196/957\|i0_LQ_HNbud_c40254/f1p25/412\|i2_LQ_HNbud_c51353/f1p16/2438\|i1_LQ_HNbud_c29237/f1p29/1049\|i2_LQ_HNbud_c3962/f1p24/2378\|i1_LQ_HNbud_c85759/f1p27/1634\|i0_HQ_HNbud_c66939/f2p13/774\|i2_LQ_HNbud_c31295/f1p3/2011\|i0_LQ_HNbud_c7875/f1p2/956\|i0_HQ_HNbud_c18431/f3p0/992\|i2_HQ_HNbud_c21399/f3p2/2368\|i0_LQ_HNbud_c50590/f1p2/744\|i1_LQ_HNbud_c89457/f1p10/1163\|i0_LQ_HNbud_c23790/f1p13/927\|i0_LQ_HNbud_c53964/f1p4/794\|i1_LQ_HNbud_c74264/f1p31/1752\|i0_HQ_HNbud_c98794/f15p0/739\|i0_LQ_HNbud_c9713/f1p2/996\|i2_HQ_HNbud_c3272/f4p4/2239\|i1_LQ_HNbud_c133377/f1p4/1258\|i2_HQ_HNbud_c1058/f19p16/2485\|i1_LQ_HNbud_c203384/f1p13/1171\|i2_LQ_HNbud_c50244/f1p10/2373\|i0_LQ_HNbud_c20152/f1p0/813\|i0_LQ_HNbud_c5154/f1p3/853\|i2_HQ_HNbud_c1863/f6p4/2318\|i0_LQ_HNbud_c66472/f1p3/932\|i0_HQ_HNbud_c17162/f26p0/801\|i2_LQ_HNbud_c22216/f1p19/2669\|i2_LQ_HNbud_c3753/f1p8/2356\|i2_LQ_HNbud_c14460/f1p13/2528\|i0_LQ_HNbud_c96561/f1p0/846\|i0_HQ_HNbud_c98374/f2p0/687\|i2_HQ_HNbud_c23078/f2p3/2594\|i1_LQ_HNbud_c5075/f1p65/1606\|i0_LQ_HNbud_c69199/f1p0/830\|i2_LQ_HNbud_c56190/f1p3/2427\|i1_HQ_HNbud_c265461/f3p13/1054\|i2_LQ_HNbud_c26355/f1p14/2890\|i0_HQ_HNbud_c2145/f17p2/799\|i2_HQ_HNbud_c66198/f56p8/2364\|i0_HQ_HNbud_c98348/f22p0/712\|i0_HQ_HNbud_c4082/f3p7/630\|i0_LQ_HNbud_c43057/f1p0/760\|i2_LQ_HNbud_c50200/f1p12/2500\|i0_HQ_HNbud_c812/f20p0/764\|i0_HQ_HNbud_c2972/f3p4/830\|i1_LQ_HNbud_c98492/f1p7/926\|i0_LQ_HNbud_c72215/f1p0/722\|i0_LQ_HNbud_c54294/f1p7/639\|i0_HQ_HNbud_c13858/f5p1/847\|i1_LQ_HNbud_c98910/f1p14/1059\|i0_LQ_HNbud_c21619/f1p1/895\|i2_LQ_HNbud_c21845/f1p4/2694\|i1_HQ_HNbud_c264056/f4p3/1135 | | |
| S1_S2_down | Plant-pathogen interaction | ko04626 | 12 | 624 | 0.000771 | 0.02159422 | 1.92E+00 | i1_HQ_HNbud_c220992/f2p30/1744\|i0_LQ_HNbud_c22602/f1p7/981\|i2_LQ_HNbud_c22216/f1p19/2669\|i2_HQ_HNbud_c20344/f3p12/2478\|i2_LQ_HNbud_c14460/f1p13/2528\|i1_HQ_HNbud_c16210/f27p26/1793\|i2_LQ_HNbud_c50200/f1p12/2500\|i1_LQ_HNbud_c74264/f1p31/1752\|i2_LQ_HNbud_c51353/f1p16/2438\|i1_LQ_HNbud_c85759/f1p27/1634\|i2_HQ_HNbud_c1058/f19p16/2485\|i1_LQ_HNbud_c11182/f1p7/1644 | | |
| S1_S3_up | DNA replication | ko03030 | 60 | 322 | 1.12E-25 | 1.24E-23 | 1.86E+01 | i2_LQ_HNbud_c56488/f1p20/3105\|i2_HQ_HNbud_c1181/f31p18/2198\|i2_HQ_HNbud_c64526/f14p1/2827\|i2_LQ_HNbud_c7224/f1p7/2795\|i1_LQ_HNbud_c222540/f1p2/1625\|i2_LQ_HNbud_c42090/f1p2/2517\|i3_HQ_HNbud_c1735/f2p1/3229\|i1_LQ_HNbud_c129292/f1p2/1189\|i1_LQ_HNbud_c20054/f1p2/1132\|i2_LQ_HNbud_c52550/f1p1/2900\|i2_LQ_HNbud_c66904/f1p1/2775\|i3_LQ_HNbud_c16568/f1p58/3022\|i2_HQ_HNbud_c65838/f12p10/2556\|i1_LQ_HNbud_c39169/f1p1/1684\|i6_LQ_HNbud_c436/f1p0/6819\|i3_LQ_HNbud_c13447/f1p0/3579\|i1_LQ_HNbud_c109815/f2p2/1152\|i1_LQ_HNbud_c105775/f1p16/1408\|i2_HQ_HNbud_c70509/f2p2/2528\|i1_HQ_HNbud_c159627/f2p1/1510\|i3_LQ_HNbud_c11393/f1p0/3465\|i1_LQ_HNbud_c204117/f1p16/1274\|i2_LQ_HNbud_c43910/f1p6/2493\|i1_HQ_HNbud_c88501/f3p2/1574\|i2_HQ_HNbud_c19772/f3p0/2817\|i2_HQ_HNbud_c66463/f2p2/2849\|i2_LQ_HNbud_c4487/f1p3/2920\|i2_HQ_HNbud_c1673/f3p3/2271\|i2_LQ_HNbud_c71566/f1p0/2011\|i2_LQ_HNbud_c39590/f1p2/2616\|i2_HQ_HNbud_c70893/f4p0/2051\|i4_LQ_HNbud_c2480/f1p0/4450\|i1_LQ_HNbud_c203685/f1p17/1773\|i1_HQ_HNbud_c65705/f2p5/2009\|i3_LQ_HNbud_c6619/f1p0/3769\|i1_LQ_HNbud_c134006/f1p3/1628\|i3_LQ_HNbud_c18604/f1p0/3002\|i2_HQ_HNbud_c64519/f36p1/2780\|i3_LQ_HNbud_c10626/f1p5/3487\|i1_HQ_HNbud_c23225/f2p2/1823\|i1_LQ_HNbud_c34692/f1p3/1561\|i2_LQ_HNbud_c47151/f1p4/2062\|i2_LQ_HNbud_c8614/f1p3/2351\|i2_LQ_HNbud_c3349/f1p1/2567\|i1_LQ_HNbud_c106324/f1p11/1134\|i2_HQ_HNbud_c70396/f3p3/2576\|i2_LQ_HNbud_c28503/f1p5/2860\|i4_LQ_HNbud_c708/f1p0/4482\|i2_LQ_HNbud_c27141/f1p1/2978\|i1_LQ_HNbud_c264776/f119p16/1206\|i1_LQ_HNbud_c241444/f1p14/1072\|i2_HQ_HNbud_c8051/f2p4/2794\|i1_LQ_HNbud_c93344/f1p3/1655\|i1_LQ_HNbud_c12422/f1p2/1711\|i1_HQ_HNbud_c7251/f4p1/1564\|i1_LQ_HNbud_c269024/f1p16/1184\|i3_LQ_HNbud_c10344/f1p2/3415\|i2_LQ_HNbud_c43501/f1p5/2570\|i4_LQ_HNbud_c2989/f1p9/4673\|i2_HQ_HNbud_c65037/f36p7/2570 | | |
| S1_S3_up | Mismatch repair | ko03430 | 24 | 189 | 3.66E-08 | 2.01E-06 | 1.27E+01 | i2_HQ_HNbud_c1181/f31p18/2198\|i1_LQ_HNbud_c129292/f1p2/1189\|i1_LQ_HNbud_c105775/f1p16/1408\|i3_LQ_HNbud_c13447/f1p0/3579\|i2_HQ_HNbud_c1673/f3p3/2271\|i3_LQ_HNbud_c11393/f1p0/3465\|i1_LQ_HNbud_c204117/f1p16/1274\|i2_HQ_HNbud_c19772/f3p0/2817\|i3_LQ_HNbud_c9469/f1p11/3124\|i2_LQ_HNbud_c73580/f1p1/1966\|i1_HQ_HNbud_c65705/f2p5/2009\|i1_LQ_HNbud_c134006/f1p3/1628\|i1_LQ_HNbud_c176120/f1p2/1941\|i1_LQ_HNbud_c34692/f1p3/1561\|i1_LQ_HNbud_c109815/f2p2/1152\|i2_LQ_HNbud_c3349/f1p1/2567\|i1_LQ_HNbud_c106324/f1p11/1134\|i1_LQ_HNbud_c264776/f119p16/1206\|i2_HQ_HNbud_c8051/f2p4/2794\|i1_LQ_HNbud_c93344/f1p3/1655\|i2_LQ_HNbud_c39590/f1p2/2616\|i1_LQ_HNbud_c269024/f1p16/1184\|i4_LQ_HNbud_c2989/f1p9/4673\|i1_LQ_HNbud_c52241/f1p0/1589 | | |
| S1_S3_up | Flavonoid biosynthesis | ko00941 | 15 | 90 | 6.34E-07 | 2.32E-05 | 1.67E+01 | i1_LQ_HNbud_c249768/f1p6/1518\|i1_LQ_HNbud_c89704/f1p13/1357\|i2_LQ_HNbud_c10759/f1p2/2821\|i3_LQ_HNbud_c15986/f1p0/3038\|i1_LQ_HNbud_c25756/f1p0/1494\|i2_HQ_HNbud_c18063/f4p8/2286\|i3_LQ_HNbud_c6906/f1p0/3505\|i1_HQ_HNbud_c191018/f23p19/1607\|i1_LQ_HNbud_c149147/f1p0/1558\|i0_LQ_HNbud_c19606/f1p4/775\|i1_HQ_HNbud_c25202/f6p17/1185\|i1_HQ_HNbud_c24406/f3p6/1370\|i1_HQ_HNbud_c19190/f2p13/1820\|i2_LQ_HNbud_c5793/f1p3/2970\|i2_HQ_HNbud_c18176/f3p5/2845 | | |
| S1_S3_up | Nucleotide excision repair | ko03420 | 29 | 362 | 1.01E-05 | 0.000279035 | 8.01E+00 | i2_HQ_HNbud_c1181/f31p18/2198\|i1_LQ_HNbud_c129292/f1p2/1189\|i6_LQ_HNbud_c436/f1p0/6819\|i3_LQ_HNbud_c13447/f1p0/3579\|i2_HQ_HNbud_c1673/f3p3/2271\|i3_LQ_HNbud_c11393/f1p0/3465\|i1_LQ_HNbud_c204117/f1p16/1274\|i2_HQ_HNbud_c19772/f3p0/2817\|i4_LQ_HNbud_c2480/f1p0/4450\|i1_HQ_HNbud_c65705/f2p5/2009\|i1_LQ_HNbud_c74875/f1p3/1749\|i3_LQ_HNbud_c6619/f1p0/3769\|i1_LQ_HNbud_c134006/f1p3/1628\|i1_LQ_HNbud_c17127/f33p5/1580\|i1_LQ_HNbud_c132708/f1p2/1611\|i1_HQ_HNbud_c23225/f2p2/1823\|i1_LQ_HNbud_c34692/f1p3/1561\|i1_LQ_HNbud_c109815/f2p2/1152\|i2_LQ_HNbud_c8614/f1p3/2351\|i2_LQ_HNbud_c3349/f1p1/2567\|i1_LQ_HNbud_c106324/f1p11/1134\|i1_LQ_HNbud_c264776/f119p16/1206\|i2_HQ_HNbud_c8051/f2p4/2794\|i1_LQ_HNbud_c93344/f1p3/1655\|i1_LQ_HNbud_c12422/f1p2/1711\|i2_LQ_HNbud_c39590/f1p2/2616\|i1_LQ_HNbud_c269024/f1p16/1184\|i1_LQ_HNbud_c105775/f1p16/1408\|i4_LQ_HNbud_c2989/f1p9/4673 | | |
| S1_S3_up | Pyrimidine metabolism | ko00240 | 45 | 730 | 2.83E-05 | 0.000622119 | 6.16E+00 | i1_HQ_HNbud_c146570/f3p2/1398\|i1_LQ_HNbud_c222540/f1p2/1625\|i0_LQ_HNbud_c42845/f1p0/898\|i0_HQ_HNbud_c99580/f2p0/901\|i1_LQ_HNbud_c203443/f1p5/1278\|i1_LQ_HNbud_c81636/f3p4/1114\|i0_LQ_HNbud_c79785/f1p0/679\|i1_HQ_HNbud_c220457/f4p8/1280\|i0_HQ_HNbud_c66677/f2p0/797\|i0_LQ_HNbud_c54474/f1p11/773\|i1_HQ_HNbud_c7884/f3p2/1194\|i1_LQ_HNbud_c39169/f1p1/1684\|i6_LQ_HNbud_c436/f1p0/6819\|i1_HQ_HNbud_c23225/f2p2/1823\|i1_HQ_HNbud_c159627/f2p1/1510\|i0_LQ_HNbud_c99631/f1p0/839\|i1_HQ_HNbud_c88501/f3p2/1574\|i4_LQ_HNbud_c2387/f1p1/4603\|i2_LQ_HNbud_c12346/f1p7/2438\|i3_LQ_HNbud_c10344/f1p2/3415\|i2_LQ_HNbud_c71566/f1p0/2011\|i2_LQ_HNbud_c70898/f2p0/2071\|i2_HQ_HNbud_c70893/f4p0/2051\|i4_LQ_HNbud_c2480/f1p0/4450\|i1_HQ_HNbud_c65705/f2p5/2009\|i2_LQ_HNbud_c57277/f1p7/2731\|i3_LQ_HNbud_c6619/f1p0/3769\|i1_LQ_HNbud_c134006/f1p3/1628\|i0_HQ_HNbud_c39475/f3p0/580\|i3_LQ_HNbud_c10626/f1p5/3487\|i1_LQ_HNbud_c111603/f1p4/1629\|i1_LQ_HNbud_c34692/f1p3/1561\|i2_LQ_HNbud_c8614/f1p3/2351\|i1_LQ_HNbud_c27646/f1p2/1824\|i1_HQ_HNbud_c192055/f32p8/1376\|i0_HQ_HNbud_c97833/f7p0/791\|i2_LQ_HNbud_c50888/f1p1/2441\|i1_LQ_HNbud_c108677/f1p2/1601\|i1_LQ_HNbud_c93344/f1p3/1655\|i1_LQ_HNbud_c89498/f1p1/1237\|i1_LQ_HNbud_c12422/f1p2/1711\|i1_HQ_HNbud_c7251/f4p1/1564\|i1_LQ_HNbud_c277774/f1p4/1020\|i1_HQ_HNbud_c74681/f4p5/1322\|i2_LQ_HNbud_c6394/f1p7/2641 | | |
| S1_S3_up | Terpenoid backbone biosynthesis | ko00900 | 28 | 393 | 9.73E-05 | 0.001784413 | 7.12E+00 | i2_LQ_HNbud_c909/f17p1/2207\|i2_HQ_HNbud_c34757/f3p5/2617\|i1_LQ_HNbud_c105700/f1p2/1293\|i2_HQ_HNbud_c65100/f74p5/2523\|i1_LQ_HNbud_c227058/f1p22/1948\|i1_LQ_HNbud_c225398/f1p3/1418\|i1_HQ_HNbud_c170393/f3p4/1304\|i2_LQ_HNbud_c51155/f1p1/2974\|i0_LQ_HNbud_c26411/f1p0/844\|i1_LQ_HNbud_c213751/f1p6/1011\|i1_LQ_HNbud_c151123/f1p3/1669\|i1_HQ_HNbud_c49776/f6p0/1762\|i1_LQ_HNbud_c91506/f1p24/1990\|i2_HQ_HNbud_c1664/f4p8/2300\|i2_LQ_HNbud_c46529/f1p0/2047\|i2_HQ_HNbud_c66247/f21p8/2621\|i0_LQ_HNbud_c30122/f1p2/928\|i1_LQ_HNbud_c19043/f1p3/1530\|i0_LQ_HNbud_c19279/f2p0/857\|i2_LQ_HNbud_c59656/f1p5/2594\|i2_LQ_HNbud_c56649/f1p4/2763\|i2_HQ_HNbud_c19242/f3p1/2263\|i1_LQ_HNbud_c87346/f1p24/1534\|i2_HQ_HNbud_c33647/f2p7/2345\|i1_LQ_HNbud_c178036/f1p15/1897\|i1_LQ_HNbud_c88413/f1p3/1139\|i1_HQ_HNbud_c219658/f172p4/1672\|i0_LQ_HNbud_c26487/f1p0/966 | | |
| S1_S3_up | Base excision repair | ko03410 | 19 | 231 | 0.000238 | 0.003316322 | 8.23E+00 | i6_LQ_HNbud_c436/f1p0/6819\|i3_LQ_HNbud_c13447/f1p0/3579\|i1_LQ_HNbud_c106324/f1p11/1134\|i1_LQ_HNbud_c93344/f1p3/1655\|i4_LQ_HNbud_c2480/f1p0/4450\|i2_HQ_HNbud_c65153/f5p2/2080\|i1_HQ_HNbud_c65705/f2p5/2009\|i1_LQ_HNbud_c264776/f119p16/1206\|i1_LQ_HNbud_c105775/f1p16/1408\|i3_LQ_HNbud_c6619/f1p0/3769\|i1_LQ_HNbud_c134006/f1p3/1628\|i1_LQ_HNbud_c12422/f1p2/1711\|i1_LQ_HNbud_c269024/f1p16/1184\|i2_LQ_HNbud_c8614/f1p3/2351\|i2_HQ_HNbud_c19772/f3p0/2817\|i1_LQ_HNbud_c204117/f1p16/1274\|i1_HQ_HNbud_c23225/f2p2/1823\|i4_LQ_HNbud_c2989/f1p9/4673\|i1_LQ_HNbud_c34692/f1p3/1561 | | |
| S1_S3_up | Glycolysis / Gluconeogenesis | ko00010 | 57 | 1100 | 0.000241 | 0.003316322 | 5.18E+00 | i0_LQ_HNbud_c4917/f1p2/929\|i2_LQ_HNbud_c5167/f1p34/2287\|i1_LQ_HNbud_c65882/f1p5/1726\|i1_HQ_HNbud_c13740/f5p8/1397\|i1_HQ_HNbud_c243070/f4p11/1548\|i2_LQ_HNbud_c30279/f1p13/1975\|i1_HQ_HNbud_c237239/f4p1/1269\|i1_LQ_HNbud_c248170/f1p2/1412\|i1_LQ_HNbud_c251999/f1p12/1473\|i2_LQ_HNbud_c31726/f2p4/2046\|i1_HQ_HNbud_c53223/f5p2/1594\|i2_LQ_HNbud_c68841/f1p1/2168\|i1_LQ_HNbud_c107750/f1p2/1422\|i1_HQ_HNbud_c4704/f7p2/1455\|i1_LQ_HNbud_c72294/f1p9/1684\|i1_HQ_HNbud_c2263/f19p6/1342\|i1_HQ_HNbud_c255347/f2p16/1964\|i1_HQ_HNbud_c65613/f7p0/1292\|i2_LQ_HNbud_c40561/f1p12/2072\|i1_HQ_HNbud_c2020/f6p7/1447\|i1_LQ_HNbud_c201906/f1p1/1419\|i2_HQ_HNbud_c19518/f5p1/2204\|i1_LQ_HNbud_c205936/f1p0/1609\|i2_HQ_HNbud_c61668/f2p2/2036\|i1_LQ_HNbud_c34561/f1p4/1472\|i1_LQ_HNbud_c231937/f1p1/1791\|i2_LQ_HNbud_c54552/f1p5/1993\|i1_LQ_HNbud_c33573/f1p9/1496\|i1_LQ_HNbud_c49555/f1p16/1851\|i1_LQ_HNbud_c38900/f1p15/1538\|i1_HQ_HNbud_c242846/f5p0/1426\|i1_LQ_HNbud_c205431/f1p0/1372\|i1_LQ_HNbud_c154658/f1p17/1392\|i1_HQ_HNbud_c151229/f2p10/1741\|i2_LQ_HNbud_c65094/f3p2/2070\|i1_HQ_HNbud_c35727/f5p1/1437\|i1_LQ_HNbud_c37735/f1p11/1524\|i1_LQ_HNbud_c93266/f1p3/1739\|i1_LQ_HNbud_c22059/f1p2/1419\|i2_HQ_HNbud_c34080/f2p0/2201\|i1_LQ_HNbud_c24160/f1p2/1751\|i2_LQ_HNbud_c38142/f1p3/2097\|i1_LQ_HNbud_c251113/f1p1/1281\|i2_LQ_HNbud_c38611/f1p2/2104\|i1_HQ_HNbud_c101990/f11p4/1364\|i1_LQ_HNbud_c51745/f1p6/1604\|i1_LQ_HNbud_c133964/f1p7/1559\|i1_LQ_HNbud_c171307/f1p9/1403\|i2_HQ_HNbud_c14088/f2p5/2407\|i1_LQ_HNbud_c85495/f1p9/1948\|i1_LQ_HNbud_c129793/f1p11/1832\|i1_LQ_HNbud_c5731/f1p3/1350\|i1_HQ_HNbud_c81331/f5p3/1838\|i1_LQ_HNbud_c112751/f1p8/1557\|i1_HQ_HNbud_c27379/f2p8/1575\|i1_HQ_HNbud_c85788/f2p4/1670\|i1_HQ_HNbud_c77939/f2p5/1354 | | |
| S1_S3_up | Circadian rhythm - plant | ko04712 | 20 | 264 | 0.000445 | 0.005441776 | 7.58E+00 | i2_LQ_HNbud_c26920/f1p17/2901\|i1_LQ_HNbud_c249768/f1p6/1518\|i2_LQ_HNbud_c72626/f1p11/2775\|i1_LQ_HNbud_c112913/f1p5/1244\|i2_LQ_HNbud_c55562/f1p1/2885\|i2_LQ_HNbud_c35178/f2p3/2529\|i3_LQ_HNbud_c15287/f1p1/3031\|i3_LQ_HNbud_c12735/f1p1/3059\|i3_LQ_HNbud_c6906/f1p0/3505\|i2_LQ_HNbud_c24976/f1p4/2806\|i2_LQ_HNbud_c53889/f1p8/2928\|i2_LQ_HNbud_c53369/f1p11/2426\|i2_HQ_HNbud_c27866/f2p1/3004\|i1_HQ_HNbud_c24406/f3p6/1370\|i2_HQ_HNbud_c70530/f3p1/2949\|i0_LQ_HNbud_c28674/f1p0/551\|i2_LQ_HNbud_c1725/f3p0/2269\|i2_LQ_HNbud_c28794/f1p1/2850\|i2_LQ_HNbud_c23772/f1p5/2950\|i0_LQ_HNbud_c79621/f1p2/890 | | |
| S1_S3_up | Homologous recombination | ko03440 | 16 | 197 | 0.000808 | 0.008884614 | 8.12E+00 | i1_LQ_HNbud_c109815/f2p2/1152\|i2_HQ_HNbud_c1181/f31p18/2198\|i2_LQ_HNbud_c3349/f1p1/2567\|i4_HQ_HNbud_c231/f2p0/4130\|i2_HQ_HNbud_c1673/f3p3/2271\|i1_HQ_HNbud_c65705/f2p5/2009\|i1_LQ_HNbud_c67192/f1p1/1312\|i2_HQ_HNbud_c8051/f2p4/2794\|i1_LQ_HNbud_c134006/f1p3/1628\|i1_LQ_HNbud_c54170/f1p0/1937\|i1_LQ_HNbud_c129292/f1p2/1189\|i2_LQ_HNbud_c39590/f1p2/2616\|i2_HQ_HNbud_c70897/f2p1/2012\|i2_LQ_HNbud_c22659/f1p0/2056\|i1_LQ_HNbud_c93344/f1p3/1655\|i1_LQ_HNbud_c34692/f1p3/1561 | | |
| S1_S3_up | Amino sugar and nucleotide sugar metabolism | ko00520 | 37 | 670 | 0.000983 | 0.009832678 | 5.52E+00 | i1_HQ_HNbud_c167861/f12p9/1437\|i1_LQ_HNbud_c103730/f1p1/1384\|i2_LQ_HNbud_c21925/f1p5/2444\|i1_HQ_HNbud_c3860/f5p1/1394\|i1_LQ_HNbud_c105795/f1p13/1196\|i2_LQ_HNbud_c24849/f1p1/2083\|i1_HQ_HNbud_c53223/f5p2/1594\|i1_HQ_HNbud_c95416/f3p1/1074\|i3_LQ_HNbud_c15303/f1p3/3019\|i1_LQ_HNbud_c6511/f1p3/1663\|i1_LQ_HNbud_c253859/f1p11/1234\|i2_HQ_HNbud_c1093/f4p0/2353\|i1_LQ_HNbud_c17893/f1p18/1916\|i2_LQ_HNbud_c9436/f1p7/2221\|i1_LQ_HNbud_c89335/f1p13/1277\|i1_LQ_HNbud_c51966/f1p2/1437\|i2_LQ_HNbud_c30523/f1p0/2041\|i3_LQ_HNbud_c7409/f1p3/3663\|i2_LQ_HNbud_c65094/f3p2/2070\|i2_LQ_HNbud_c43273/f1p4/2465\|i2_HQ_HNbud_c47568/f5p0/2245\|i2_LQ_HNbud_c38865/f1p5/2329\|i2_LQ_HNbud_c59995/f1p4/2701\|i2_HQ_HNbud_c5375/f5p2/2522\|i3_LQ_HNbud_c13159/f1p0/3705\|i3_HQ_HNbud_c1208/f3p0/3855\|i2_LQ_HNbud_c38142/f1p3/2097\|i2_HQ_HNbud_c50393/f3p4/2638\|i2_LQ_HNbud_c50605/f1p3/2598\|i1_LQ_HNbud_c221631/f1p5/1293\|i3_HQ_HNbud_c16812/f6p0/3360\|i1_HQ_HNbud_c217358/f13p18/1935\|i1_LQ_HNbud_c13544/f1p13/1221\|i1_LQ_HNbud_c51511/f1p12/1679\|i1_HQ_HNbud_c226180/f4p8/1506\|i2_LQ_HNbud_c27385/f1p0/2340\|i2_LQ_HNbud_c22715/f1p0/2369 | | |
| S1_S3_up | Plant hormone signal transduction | ko04075 | 49 | 988 | 0.001527 | 0.013994088 | 4.96E+00 | i2_LQ_HNbud_c59627/f1p2/2578\|i0_LQ_HNbud_c85885/f2p0/957\|i1_LQ_HNbud_c203842/f1p1/1226\|i0_LQ_HNbud_c49766/f1p2/843\|i1_LQ_HNbud_c180558/f1p1/1786\|i1_HQ_HNbud_c218401/f8p8/1542\|i1_LQ_HNbud_c224971/f1p7/1768\|i1_LQ_HNbud_c175395/f4p4/1894\|i1_LQ_HNbud_c103859/f1p10/1918\|i1_LQ_HNbud_c132373/f1p5/1157\|i3_LQ_HNbud_c14656/f1p0/3548\|i1_LQ_HNbud_c208348/f3p1/1051\|i0_HQ_HNbud_c29827/f2p0/987\|i0_LQ_HNbud_c107503/f1p0/476\|i1_LQ_HNbud_c8162/f1p1/1138\|i2_LQ_HNbud_c22583/f1p12/2056\|i1_LQ_HNbud_c253406/f1p1/1553\|i1_LQ_HNbud_c49140/f3p5/1314\|i2_LQ_HNbud_c15767/f1p0/2099\|i1_LQ_HNbud_c130203/f1p6/1585\|i0_LQ_HNbud_c11320/f1p1/857\|i0_HQ_HNbud_c26374/f3p3/625\|i2_LQ_HNbud_c18424/f1p2/2106\|i2_LQ_HNbud_c16022/f1p2/2042\|i1_HQ_HNbud_c1913/f19p8/1657\|i1_LQ_HNbud_c200814/f1p5/1292\|i1_LQ_HNbud_c68983/f3p4/1755\|i0_LQ_HNbud_c70302/f1p11/972\|i1_HQ_HNbud_c245513/f10p9/1763\|i1_HQ_HNbud_c106733/f4p6/1482\|i2_LQ_HNbud_c46045/f1p1/2016\|i1_HQ_HNbud_c255876/f3p0/1079\|i1_HQ_HNbud_c8186/f2p9/1615\|i0_LQ_HNbud_c73604/f1p6/589\|i2_LQ_HNbud_c33772/f1p0/2533\|i4_LQ_HNbud_c2971/f1p8/4269\|i1_HQ_HNbud_c168502/f9p5/1052\|i0_LQ_HNbud_c11391/f1p0/987\|i2_LQ_HNbud_c62348/f1p0/2014\|i2_LQ_HNbud_c28640/f1p0/2096\|i1_LQ_HNbud_c140385/f1p1/1041\|i2_LQ_HNbud_c10007/f1p2/2225\|i2_LQ_HNbud_c12744/f1p0/2626\|i1_HQ_HNbud_c264929/f8p3/1211\|i1_LQ_HNbud_c203972/f1p7/1370\|i1_LQ_HNbud_c116615/f1p2/1014\|i1_HQ_HNbud_c62811/f46p9/1570\|i0_LQ_HNbud_c81259/f1p5/669\|i1_LQ_HNbud_c25126/f1p4/1418 | | |
| S1_S3_up | Arginine and proline metabolism | ko00330 | 22 | 348 | 0.002178 | 0.018432986 | 6.32E+00 | i0_LQ_HNbud_c4917/f1p2/929\|i1_LQ_HNbud_c34873/f1p2/1552\|i1_LQ_HNbud_c23342/f1p20/1685\|i1_LQ_HNbud_c251113/f1p1/1281\|i1_HQ_HNbud_c38319/f7p6/1866\|i3_LQ_HNbud_c5688/f1p4/3260\|i1_LQ_HNbud_c266029/f3p36/1731\|i2_LQ_HNbud_c40561/f1p12/2072\|i1_HQ_HNbud_c1881/f42p27/1838\|i1_HQ_HNbud_c627/f34p27/1706\|i1_LQ_HNbud_c130686/f1p27/1699\|i1_HQ_HNbud_c151229/f2p10/1741\|i1_LQ_HNbud_c199566/f1p33/1879\|i1_HQ_HNbud_c37260/f18p38/1791\|i1_LQ_HNbud_c253627/f1p37/1792\|i2_HQ_HNbud_c34080/f2p0/2201\|i1_LQ_HNbud_c170377/f1p32/1820\|i1_LQ_HNbud_c231937/f1p1/1791\|i2_LQ_HNbud_c30279/f1p13/1975\|i1_LQ_HNbud_c177068/f1p7/1700\|i2_LQ_HNbud_c53814/f1p11/2086\|i1_LQ_HNbud_c56957/f1p2/1416 | | |
| S1_S3_up | Flavone and flavonol biosynthesis | ko00944 | 5 | 27 | 0.002536 | 0.019928847 | 1.85E+01 | i1_LQ_HNbud_c14930/f1p3/1010\|i1_LQ_HNbud_c214363/f1p4/1012\|i1_HQ_HNbud_c95464/f3p4/1045\|i1_HQ_HNbud_c19190/f2p13/1820\|i2_HQ_HNbud_c18063/f4p8/2286 | | |
| S1_S3_up | Purine metabolism | ko00230 | 47 | 975 | 0.003158 | 0.023159992 | 4.82E+00 | i1_LQ_HNbud_c222540/f1p2/1625\|i1_LQ_HNbud_c155762/f1p1/1370\|i0_HQ_HNbud_c64640/f13p0/988\|i1_LQ_HNbud_c24256/f1p3/1398\|i0_HQ_HNbud_c99580/f2p0/901\|i1_LQ_HNbud_c150807/f1p23/1399\|i1_LQ_HNbud_c203443/f1p5/1278\|i0_LQ_HNbud_c79785/f1p0/679\|i1_HQ_HNbud_c220457/f4p8/1280\|i1_HQ_HNbud_c101295/f25p29/1402\|i0_LQ_HNbud_c54474/f1p11/773\|i2_LQ_HNbud_c68841/f1p1/2168\|i1_LQ_HNbud_c42039/f2p18/1340\|i1_LQ_HNbud_c39169/f1p1/1684\|i6_LQ_HNbud_c436/f1p0/6819\|i1_LQ_HNbud_c98700/f1p1/1060\|i1_HQ_HNbud_c23225/f2p2/1823\|i1_HQ_HNbud_c159627/f2p1/1510\|i1_LQ_HNbud_c174365/f1p3/1293\|i2_HQ_HNbud_c19518/f5p1/2204\|i1_HQ_HNbud_c32598/f5p3/1154\|i2_HQ_HNbud_c61668/f2p2/2036\|i1_HQ_HNbud_c88501/f3p2/1574\|i4_LQ_HNbud_c2387/f1p1/4603\|i1_HQ_HNbud_c192055/f32p8/1376\|i1_LQ_HNbud_c34692/f1p3/1561\|i3_LQ_HNbud_c10344/f1p2/3415\|i2_LQ_HNbud_c71566/f1p0/2011\|i2_LQ_HNbud_c70898/f2p0/2071\|i2_HQ_HNbud_c70893/f4p0/2051\|i1_LQ_HNbud_c50271/f2p18/1312\|i4_LQ_HNbud_c2480/f1p0/4450\|i1_HQ_HNbud_c65705/f2p5/2009\|i2_LQ_HNbud_c65094/f3p2/2070\|i2_LQ_HNbud_c57277/f1p7/2731\|i3_LQ_HNbud_c6619/f1p0/3769\|i1_LQ_HNbud_c134006/f1p3/1628\|i1_LQ_HNbud_c67903/f1p1/1751\|i3_LQ_HNbud_c10626/f1p5/3487\|i1_LQ_HNbud_c111603/f1p4/1629\|i2_LQ_HNbud_c38142/f1p3/2097\|i2_LQ_HNbud_c8614/f1p3/2351\|i1_LQ_HNbud_c93344/f1p3/1655\|i1_LQ_HNbud_c12422/f1p2/1711\|i1_HQ_HNbud_c7251/f4p1/1564\|i1_HQ_HNbud_c74681/f4p5/1322\|i2_LQ_HNbud_c6394/f1p7/2641 | | |
| S1_S3_up | Caffeine metabolism | ko00232 | 3 | 9 | 0.004915 | 0.03180312 | 3.33E+01 | i1_LQ_HNbud_c24256/f1p3/1398\|i1_HQ_HNbud_c32598/f5p3/1154\|i1_LQ_HNbud_c174365/f1p3/1293 | | |
| S1_S3_up | Starch and sucrose metabolism | ko00500 | 49 | 1049 | 0.004634 | 0.03180312 | 4.67E+00 | i2_LQ_HNbud_c45520/f1p3/2044\|i2_LQ_HNbud_c51752/f1p6/2481\|i2_LQ_HNbud_c21925/f1p5/2444\|i1_HQ_HNbud_c264990/f3p9/1892\|i1_LQ_HNbud_c105795/f1p13/1196\|i1_HQ_HNbud_c53223/f5p2/1594\|i1_HQ_HNbud_c3813/f4p2/1840\|i2_HQ_HNbud_c70880/f3p3/2105\|i1_LQ_HNbud_c222584/f1p6/1287\|i1_LQ_HNbud_c268627/f1p9/1914\|i1_HQ_HNbud_c35477/f2p0/1286\|i2_LQ_HNbud_c39159/f1p6/2932\|i1_LQ_HNbud_c6511/f1p3/1663\|i1_LQ_HNbud_c202139/f1p4/1966\|i1_HQ_HNbud_c49690/f5p1/1671\|i1_LQ_HNbud_c17893/f1p18/1916\|i2_LQ_HNbud_c41447/f1p9/2963\|i2_LQ_HNbud_c39350/f1p3/2274\|i2_LQ_HNbud_c59995/f1p4/2701\|i2_LQ_HNbud_c9436/f1p7/2221\|i2_LQ_HNbud_c35719/f1p8/2873\|i1_LQ_HNbud_c204745/f1p2/1663\|i1_LQ_HNbud_c89335/f1p13/1277\|i1_LQ_HNbud_c51966/f1p2/1437\|i3_LQ_HNbud_c7409/f1p3/3663\|i1_LQ_HNbud_c228392/f1p0/1990\|i2_LQ_HNbud_c65094/f3p2/2070\|i2_LQ_HNbud_c43273/f1p4/2465\|i2_HQ_HNbud_c47568/f5p0/2245\|i2_LQ_HNbud_c38865/f1p5/2329\|i1_LQ_HNbud_c126439/f1p0/1912\|i1_LQ_HNbud_c67903/f1p1/1751\|i2_HQ_HNbud_c5375/f5p2/2522\|i3_LQ_HNbud_c13159/f1p0/3705\|i3_HQ_HNbud_c1208/f3p0/3855\|i2_LQ_HNbud_c38142/f1p3/2097\|i2_LQ_HNbud_c27385/f1p0/2340\|i2_LQ_HNbud_c50605/f1p3/2598\|i1_LQ_HNbud_c92697/f1p8/1827\|i1_LQ_HNbud_c55083/f1p3/1833\|i1_LQ_HNbud_c260206/f2p3/1833\|i2_LQ_HNbud_c50325/f1p2/2134\|i1_HQ_HNbud_c217358/f13p18/1935\|i1_LQ_HNbud_c13544/f1p13/1221\|i1_HQ_HNbud_c17556/f3p0/1824\|i1_LQ_HNbud_c253859/f1p11/1234\|i1_HQ_HNbud_c226180/f4p8/1506\|i1_HQ_HNbud_c86339/f2p6/1605\|i2_LQ_HNbud_c22715/f1p0/2369 | | |
| S1_S3_up | Cysteine and methionine metabolism | ko00270 | 37 | 747 | 0.005483 | 0.033505768 | 4.95E+00 | i1_HQ_HNbud_c192226/f98p20/1453\|i1_HQ_HNbud_c167661/f2p0/1548\|i1_LQ_HNbud_c34873/f1p2/1552\|i3_LQ_HNbud_c8499/f1p1/3085\|i2_LQ_HNbud_c53814/f1p11/2086\|i1_LQ_HNbud_c23342/f1p20/1685\|i4_LQ_HNbud_c3322/f1p0/4959\|i1_HQ_HNbud_c25966/f4p9/1428\|i1_LQ_HNbud_c266029/f3p36/1731\|i4_HQ_HNbud_c51/f6p0/5036\|i1_HQ_HNbud_c115466/f2p0/1100\|i1_LQ_HNbud_c197155/f1p5/1913\|i1_HQ_HNbud_c65613/f7p0/1292\|i1_LQ_HNbud_c205936/f1p0/1609\|i1_LQ_HNbud_c57076/f1p5/1631\|i1_LQ_HNbud_c247382/f1p0/1377\|i1_LQ_HNbud_c9645/f1p34/1581\|i1_HQ_HNbud_c1881/f42p27/1838\|i1_HQ_HNbud_c627/f34p27/1706\|i1_LQ_HNbud_c130686/f1p27/1699\|i3_LQ_HNbud_c13324/f1p0/3983\|i2_HQ_HNbud_c831/f10p3/2956\|i1_LQ_HNbud_c129928/f1p0/1489\|i1_LQ_HNbud_c199566/f1p33/1879\|i3_LQ_HNbud_c2683/f1p0/3326\|i4_LQ_HNbud_c1987/f1p3/4213\|i1_HQ_HNbud_c37260/f18p38/1791\|i1_HQ_HNbud_c93301/f2p9/1475\|i1_LQ_HNbud_c129690/f1p16/1470\|i1_HQ_HNbud_c81227/f14p9/1594\|i1_LQ_HNbud_c170377/f1p32/1820\|i1_LQ_HNbud_c228084/f1p22/1389\|i1_LQ_HNbud_c177068/f1p7/1700\|i1_HQ_HNbud_c242706/f6p0/1904\|i3_LQ_HNbud_c11737/f1p2/3884\|i1_HQ_HNbud_c187229/f2p0/1484\|i1_LQ_HNbud_c253627/f1p37/1792 | | |
| S1_S3_up | Limonene and pinene degradation | ko00903 | 7 | 66 | 0.006471 | 0.036529712 | 1.06E+01 | i0_LQ_HNbud_c4917/f1p2/929\|i2_LQ_HNbud_c30279/f1p13/1975\|i1_LQ_HNbud_c231937/f1p1/1791\|i1_HQ_HNbud_c151229/f2p10/1741\|i2_LQ_HNbud_c40561/f1p12/2072\|i1_LQ_HNbud_c251113/f1p1/1281\|i2_HQ_HNbud_c34080/f2p0/2201 | | |
| S1_S3_up | Pentose and glucuronate interconversions | ko00040 | 20 | 338 | 0.006642 | 0.036529712 | 5.92E+00 | i1_LQ_HNbud_c130570/f2p0/1699\|i0_LQ_HNbud_c4917/f1p2/929\|i2_LQ_HNbud_c62913/f1p2/2020\|i2_LQ_HNbud_c30279/f1p13/1975\|i1_LQ_HNbud_c231937/f1p1/1791\|i1_HQ_HNbud_c48286/f3p4/1191\|i1_HQ_HNbud_c25375/f8p0/1491\|i1_LQ_HNbud_c228392/f1p0/1990\|i1_HQ_HNbud_c151229/f2p10/1741\|i2_LQ_HNbud_c40561/f1p12/2072\|i1_HQ_HNbud_c217358/f13p18/1935\|i1_LQ_HNbud_c251113/f1p1/1281\|i1_LQ_HNbud_c7500/f1p0/1768\|i2_HQ_HNbud_c47568/f5p0/2245\|i1_LQ_HNbud_c17893/f1p18/1916\|i2_LQ_HNbud_c62944/f1p2/2044\|i1_LQ_HNbud_c249088/f1p0/1753\|i2_HQ_HNbud_c34080/f2p0/2201\|i2_LQ_HNbud_c27385/f1p0/2340\|i2_LQ_HNbud_c22715/f1p0/2369 | | |
| S1_S3_up | Galactose metabolism | ko00052 | 17 | 276 | 0.008234 | 0.043128609 | 6.16E+00 | i1_LQ_HNbud_c91119/f1p1/1141\|i2_HQ_HNbud_c16390/f22p0/2827\|i2_LQ_HNbud_c18412/f1p8/3004\|i1_LQ_HNbud_c58036/f1p16/1849\|i2_LQ_HNbud_c65094/f3p2/2070\|i1_LQ_HNbud_c88755/f1p5/1574\|i1_HQ_HNbud_c1949/f12p6/1479\|i1_LQ_HNbud_c204429/f1p1/1267\|i2_LQ_HNbud_c22715/f1p0/2369\|i1_HQ_HNbud_c81331/f5p3/1838\|i1_LQ_HNbud_c202606/f1p0/1350\|i2_HQ_HNbud_c17966/f2p8/2737\|i1_HQ_HNbud_c53223/f5p2/1594\|i2_LQ_HNbud_c27385/f1p0/2340\|i2_LQ_HNbud_c35719/f1p8/2873\|i2_HQ_HNbud_c47568/f5p0/2245\|i2_LQ_HNbud_c38142/f1p3/2097 | | |
| S1_S3_up | Fructose and mannose metabolism | ko00051 | 20 | 351 | 0.009624 | 0.048118324 | 5.70E+00 | i1_LQ_HNbud_c89335/f1p13/1277\|i1_HQ_HNbud_c4704/f7p2/1455\|i2_LQ_HNbud_c9436/f1p7/2221\|i1_LQ_HNbud_c205431/f1p0/1372\|i1_LQ_HNbud_c154658/f1p17/1392\|i1_LQ_HNbud_c51511/f1p12/1679\|i1_HQ_HNbud_c2020/f6p7/1447\|i1_LQ_HNbud_c103730/f1p1/1384\|i1_LQ_HNbud_c248170/f1p2/1412\|i1_LQ_HNbud_c253859/f1p11/1234\|i1_LQ_HNbud_c13544/f1p13/1221\|i1_HQ_HNbud_c3860/f5p1/1394\|i1_HQ_HNbud_c81331/f5p3/1838\|i1_LQ_HNbud_c105795/f1p13/1196\|i1_LQ_HNbud_c51966/f1p2/1437\|i1_LQ_HNbud_c216360/f17p5/1560\|i1_HQ_HNbud_c95416/f3p1/1074\|i1_HQ_HNbud_c35727/f5p1/1437\|i1_HQ_HNbud_c53223/f5p2/1594\|i1_LQ_HNbud_c34561/f1p4/1472 | | |
| S1_S3_down | Protein processing in endoplasmic reticulum | ko04141 | 113 | 1811 | 3.93E-47 | 2.95E-45 | 6.24E+00 | i0_LQ_HNbud_c22602/f1p7/981\|i1_HQ_HNbud_c16322/f2p11/1127\|i1_LQ_HNbud_c214019/f1p19/901\|i0_LQ_HNbud_c9667/f1p0/879\|i1_HQ_HNbud_c218348/f13p6/1127\|i0_LQ_HNbud_c55218/f1p8/735\|i4_LQ_HNbud_c1121/f1p0/4852\|i2_LQ_HNbud_c52388/f1p7/2344\|i0_HQ_HNbud_c2145/f17p2/799\|i3_LQ_HNbud_c16433/f1p0/3028\|i2_HQ_HNbud_c21399/f3p2/2368\|i0_LQ_HNbud_c50590/f1p2/744\|i0_HQ_HNbud_c98794/f15p0/739\|i2_HQ_HNbud_c1058/f19p16/2485\|i1_LQ_HNbud_c11182/f1p7/1644\|i0_LQ_HNbud_c40254/f1p25/412\|i2_LQ_HNbud_c14460/f1p13/2528\|i0_HQ_HNbud_c7301/f5p0/958\|i0_HQ_HNbud_c17162/f26p0/801\|i1_LQ_HNbud_c5075/f1p65/1606\|i2_LQ_HNbud_c23994/f1p4/2838\|i1_HQ_HNbud_c49290/f2p4/1779\|i1_HQ_HNbud_c265461/f3p13/1054\|i2_LQ_HNbud_c3807/f1p13/2450\|i0_LQ_HNbud_c43057/f1p0/760\|i1_LQ_HNbud_c98492/f1p7/926\|i2_LQ_HNbud_c1569/f5p13/2465\|i0_LQ_HNbud_c54294/f1p7/639\|i0_HQ_HNbud_c37987/f27p2/714\|i1_LQ_HNbud_c110252/f4p6/1234\|i0_LQ_HNbud_c13339/f1p2/780\|i0_HQ_HNbud_c714/f13p0/823\|i2_HQ_HNbud_c1042/f14p14/2586\|i0_LQ_HNbud_c66472/f1p3/932\|i0_LQ_HNbud_c24842/f1p4/759\|i1_LQ_HNbud_c196213/f1p24/1171\|i2_HQ_HNbud_c3208/f2p20/2217\|i2_LQ_HNbud_c51353/f1p16/2438\|i0_HQ_HNbud_c13858/f5p1/847\|i2_LQ_HNbud_c3962/f1p24/2378\|i1_LQ_HNbud_c85759/f1p27/1634\|i0_LQ_HNbud_c23790/f1p13/927\|i0_LQ_HNbud_c9713/f1p2/996\|i2_LQ_HNbud_c50244/f1p10/2373\|i2_LQ_HNbud_c27323/f1p3/2372\|i2_LQ_HNbud_c22216/f1p19/2669\|i0_HQ_HNbud_c98374/f2p0/687\|i2_HQ_HNbud_c23078/f2p3/2594\|i2_LQ_HNbud_c15446/f1p12/2033\|i2_LQ_HNbud_c56190/f1p3/2427\|i2_LQ_HNbud_c26355/f1p14/2890\|i2_HQ_HNbud_c66198/f56p8/2364\|i0_HQ_HNbud_c98348/f22p0/712\|i2_HQ_HNbud_c3272/f4p4/2239\|i1_HQ_HNbud_c92444/f2p1/1949\|i0_HQ_HNbud_c2972/f3p4/830\|i1_HQ_HNbud_c38444/f3p25/1706\|i2_LQ_HNbud_c21845/f1p4/2694\|i1_HQ_HNbud_c264056/f4p3/1135\|i1_HQ_HNbud_c220992/f2p30/1744\|i2_LQ_HNbud_c26062/f1p6/2320\|i0_LQ_HNbud_c74044/f1p0/810\|i0_LQ_HNbud_c21181/f1p10/781\|i2_LQ_HNbud_c31295/f1p3/2011\|i0_LQ_HNbud_c45581/f1p0/806\|i0_HQ_HNbud_c18431/f3p0/992\|i2_HQ_HNbud_c20344/f3p12/2478\|i1_LQ_HNbud_c104751/f1p0/1122\|i0_HQ_HNbud_c66939/f2p13/774\|i1_LQ_HNbud_c133377/f1p4/1258\|i0_LQ_HNbud_c72168/f1p1/736\|i1_LQ_HNbud_c203384/f1p13/1171\|i0_LQ_HNbud_c49974/f1p0/417\|i0_LQ_HNbud_c53643/f1p0/748\|i0_HQ_HNbud_c38308/f10p4/862\|i0_HQ_HNbud_c39478/f3p7/787\|i0_HQ_HNbud_c4082/f3p7/630\|i0_LQ_HNbud_c69199/f1p0/830\|i0_HQ_HNbud_c812/f20p0/764\|i0_LQ_HNbud_c24909/f1p0/876\|i1_LQ_HNbud_c201751/f1p23/1972\|i1_LQ_HNbud_c278370/f1p26/1014\|i1_LQ_HNbud_c29237/f1p29/1049\|i0_LQ_HNbud_c31723/f1p0/862\|i0_LQ_HNbud_c28549/f1p1/796\|i0_LQ_HNbud_c41102/f1p196/957\|i0_LQ_HNbud_c7875/f1p2/956\|i0_LQ_HNbud_c10183/f2p5/748\|i0_HQ_HNbud_c98265/f22p0/766\|i0_LQ_HNbud_c72215/f1p0/722\|i0_LQ_HNbud_c53964/f1p4/794\|i1_LQ_HNbud_c74264/f1p31/1752\|i0_LQ_HNbud_c20152/f1p0/813\|i0_HQ_HNbud_c1448/f8p0/734\|i0_LQ_HNbud_c5154/f1p3/853\|i2_HQ_HNbud_c1863/f6p4/2318\|i1_HQ_HNbud_c16210/f27p26/1793\|i0_LQ_HNbud_c7037/f1p4/527\|i0_LQ_HNbud_c96561/f1p0/846\|i0_HQ_HNbud_c9856/f5p16/890\|i0_HQ_HNbud_c28788/f3p0/776\|i1_LQ_HNbud_c81741/f2p11/1077\|i0_HQ_HNbud_c25827/f2p6/851\|i0_HQ_HNbud_c98090/f32p0/732\|i2_HQ_HNbud_c9846/f2p22/2348\|i1_LQ_HNbud_c155489/f1p8/1274\|i2_LQ_HNbud_c3753/f1p8/2356\|i2_LQ_HNbud_c50200/f1p12/2500\|i0_LQ_HNbud_c25693/f1p8/787\|i1_LQ_HNbud_c10324/f1p17/1064\|i1_LQ_HNbud_c98910/f1p14/1059\|i0_LQ_HNbud_c21619/f1p1/895\|i0_HQ_HNbud_c39665/f2p0/810 | | |
| S1_S3_down | Spliceosome | ko03040 | 54 | 2169 | 3.12E-07 | 1.17E-05 | 2.49E+00 | i1_LQ_HNbud_c265050/f2p4/1150\|i2_LQ_HNbud_c26062/f1p6/2320\|i1_HQ_HNbud_c193495/f17p3/1402\|i2_HQ_HNbud_c3208/f2p20/2217\|i1_LQ_HNbud_c34204/f1p6/1381\|i2_LQ_HNbud_c3587/f1p11/3008\|i2_LQ_HNbud_c28493/f1p2/2516\|i4_LQ_HNbud_c1121/f1p0/4852\|i2_LQ_HNbud_c52388/f1p7/2344\|i2_LQ_HNbud_c51643/f1p7/2585\|i2_HQ_HNbud_c21399/f3p2/2368\|i1_HQ_HNbud_c51789/f3p9/1311\|i1_LQ_HNbud_c279608/f1p3/1022\|i1_LQ_HNbud_c180862/f1p6/1388\|i1_LQ_HNbud_c104361/f1p5/977\|i2_HQ_HNbud_c9846/f2p22/2348\|i1_LQ_HNbud_c21267/f1p4/1796\|i1_LQ_HNbud_c29924/f1p5/1102\|i2_LQ_HNbud_c26825/f1p9/2334\|i1_HQ_HNbud_c55346/f2p9/1824\|i1_LQ_HNbud_c74990/f1p4/1541\|i2_LQ_HNbud_c50244/f1p10/2373\|i2_LQ_HNbud_c27323/f1p3/2372\|i2_HQ_HNbud_c1863/f6p4/2318\|i1_LQ_HNbud_c257232/f1p8/1652\|i0_LQ_HNbud_c84679/f2p0/963\|i1_LQ_HNbud_c269319/f3p10/1041\|i2_LQ_HNbud_c54387/f1p23/2708\|i1_HQ_HNbud_c3869/f8p4/1411\|i1_LQ_HNbud_c5075/f1p65/1606\|i1_LQ_HNbud_c51995/f1p3/1681\|i1_LQ_HNbud_c27267/f1p5/919\|i1_LQ_HNbud_c155863/f1p5/1043\|i2_LQ_HNbud_c56190/f1p3/2427\|i1_LQ_HNbud_c249303/f1p10/1883\|i2_LQ_HNbud_c26355/f1p14/2890\|i1_LQ_HNbud_c56619/f1p10/1562\|i2_LQ_HNbud_c2263/f3p12/2517\|i2_HQ_HNbud_c66198/f56p8/2364\|i1_LQ_HNbud_c106489/f1p5/1168\|i2_HQ_HNbud_c3272/f4p4/2239\|i1_LQ_HNbud_c62386/f1p3/1054\|i1_LQ_HNbud_c201797/f1p6/1462\|i1_LQ_HNbud_c10324/f1p17/1064\|i2_LQ_HNbud_c3962/f1p24/2378\|i0_LQ_HNbud_c7037/f1p4/527\|i0_HQ_HNbud_c97963/f14p0/1002\|i2_LQ_HNbud_c9613/f1p6/2474\|i1_HQ_HNbud_c183118/f4p2/1083\|i1_LQ_HNbud_c91346/f1p2/1919\|i2_LQ_HNbud_c21845/f1p4/2694\|i0_HQ_HNbud_c39665/f2p0/810\|i7_LQ_HNbud_c133/f1p0/7417\|i1_HQ_HNbud_c18518/f2p5/1756 | | |
| S1_S3_down | Plant-pathogen interaction | ko04626 | 17 | 624 | 0.001555 | 0.038863315 | 2.72E+00 | i1_HQ_HNbud_c16210/f27p26/1793\|i0_LQ_HNbud_c22602/f1p7/981\|i2_LQ_HNbud_c22216/f1p19/2669\|i2_HQ_HNbud_c20344/f3p12/2478\|i2_HQ_HNbud_c1042/f14p14/2586\|i2_LQ_HNbud_c14460/f1p13/2528\|i1_HQ_HNbud_c220992/f2p30/1744\|i1_HQ_HNbud_c38444/f3p25/1706\|i2_LQ_HNbud_c50200/f1p12/2500\|i1_LQ_HNbud_c74264/f1p31/1752\|i1_LQ_HNbud_c11182/f1p7/1644\|i2_LQ_HNbud_c51353/f1p16/2438\|i1_LQ_HNbud_c85759/f1p27/1634\|i0_LQ_HNbud_c72168/f1p1/736\|i2_HQ_HNbud_c1058/f19p16/2485\|i2_LQ_HNbud_c3807/f1p13/2450\|i2_LQ_HNbud_c1569/f5p13/2465 | | |
| S2_S3_up | Flavonoid biosynthesis | ko00941 | 8 | 90 | 1.59E-10 | 7.32E-09 | 8.89E-02 | i1_LQ_HNbud_c249768/f1p6/1518\|i1_LQ_HNbud_c89704/f1p13/1357\|i2_LQ_HNbud_c10759/f1p2/2821\|i3_LQ_HNbud_c15986/f1p0/3038\|i1_HQ_HNbud_c19190/f2p13/1820\|i1_HQ_HNbud_c25202/f6p17/1185\|i1_HQ_HNbud_c24406/f3p6/1370\|i2_HQ_HNbud_c18176/f3p5/2845 | | |
| S2_S3_up | Flavone and flavonol biosynthesis | ko00944 | 3 | 27 | 5.96E-05 | 0.000996925 | 1.11E-01 | i1_LQ_HNbud_c14930/f1p3/1010\|i1_HQ_HNbud_c95464/f3p4/1045\|i1_HQ_HNbud_c19190/f2p13/1820 | | |
| S2_S3_up | Circadian rhythm - plant | ko04712 | 6 | 264 | 6.50E-05 | 0.000996925 | 2.27E-02 | i2_LQ_HNbud_c26920/f1p17/2901\|i1_LQ_HNbud_c249768/f1p6/1518\|i2_LQ_HNbud_c72626/f1p11/2775\|i1_HQ_HNbud_c24406/f3p6/1370\|i2_LQ_HNbud_c53889/f1p8/2928\|i2_LQ_HNbud_c28794/f1p1/2850 | | |
| S2_S3_up | Tyrosine metabolism | ko00350 | 6 | 307 | 0.000146 | 0.001676535 | 1.95E-02 | i1_HQ_HNbud_c101990/f11p4/1364\|i1_LQ_HNbud_c133964/f1p7/1559\|i2_LQ_HNbud_c5167/f1p34/2287\|i1_LQ_HNbud_c251048/f1p2/1945\|i1_LQ_HNbud_c5731/f1p3/1350\|i1_HQ_HNbud_c85788/f2p4/1670 | | |
| S2_S3_up | Plant hormone signal transduction | ko04075 | 9 | 988 | 0.000946 | 0.008701569 | 9.11E-03 | i2_LQ_HNbud_c59627/f1p2/2578\|i1_LQ_HNbud_c54643/f1p1/1530\|i0_LQ_HNbud_c85885/f2p0/957\|i0_LQ_HNbud_c70302/f1p11/972\|i1_LQ_HNbud_c8162/f1p1/1138\|i2_LQ_HNbud_c12744/f1p0/2626\|i1_LQ_HNbud_c68983/f3p4/1755\|i0_LQ_HNbud_c81259/f1p5/669\|i1_LQ_HNbud_c49140/f3p5/1314 | | |
| S2_S3_down | Photosynthesis - antenna proteins | ko00196 | 6 | 287 | 2.17E-05 | 0.000368391 | 2.090592334 | i1_LQ_HNbud_c19042/f1p6/1136\|i1_HQ_HNbud_c256039/f2p6/1068\|i1_LQ_HNbud_c163289/f1p5/897\|i0_LQ_HNbud_c9792/f1p0/892\|i1_HQ_HNbud_c122296/f22p46/1073\|i1_HQ_HNbud_c264980/f6p6/1106 | | |
| S2_S3_down | Phagosome | ko04145 | 8 | 554 | 1.24E-05 | 0.000368391 | 1.444043321 | i1_LQ_HNbud_c227827/f1p6/1826\|i1_LQ_HNbud_c116421/f1p8/1115\|i0_LQ_HNbud_c82095/f1p0/942\|i1_HQ_HNbud_c1309/f36p9/1643\|i1_LQ_HNbud_c23193/f1p15/1671\|i1_LQ_HNbud_c133141/f1p23/1803\|i1_LQ_HNbud_c109371/f1p23/1806\|i1_HQ_HNbud_c264754/f104p24/1739 | | |

**TableS3. Summary of DEGs related to photoperiod pathway.**

| ATH/Rice Gene ID | Gene ID | Identity % | Alignment length (aa) | Gene Symbol | Pathway | FPKM in VS | FPKM in DS | FPKM in DCS |
| --- | --- | --- | --- | --- | --- | --- | --- | --- |
| AT5G19550.1 | i1_HQ_HNbud_c36830/f4p8/1466 | 91.34 | 404 | AAT2 | Photoperiod | 0 | 0 | 0 |
| AT5G19550.1 | i1_LQ_HNbud_c226671/f1p5/1547 | 91.11 | 405 | AAT2 | Photoperiod | 0 | 0 | 0 |
| AT5G19550.1 | i1_LQ_HNbud_c86081/f1p11/1543 | 82.67 | 404 | AAT2 | Photoperiod | 9.056666667 | 16.57 | 25.69333333 |
| AT5G19550.1 | i1_HQ_HNbud_c1780/f6p2/1607 | 81.89 | 403 | AAT2 | Photoperiod | #N/A | #N/A | #N/A |
| AT5G19550.1 | i1_HQ_HNbud_c69282/f2p2/1679 | 81.89 | 403 | AAT2 | Photoperiod | 2.766666667 | 1.773333333 | 4.88 |
| AT5G19550.1 | i1_LQ_HNbud_c125803/f1p2/1735 | 81.89 | 403 | AAT2 | Photoperiod | 2.356666667 | 1.65 | 3.076666667 |
| AT5G19550.1 | i1_LQ_HNbud_c176889/f1p2/1689 | 81.89 | 403 | AAT2 | Photoperiod | 0.013333333 | 0.02 | 0.07 |
| AT5G19550.1 | i1_LQ_HNbud_c32778/f1p2/1789 | 81.89 | 403 | AAT2 | Photoperiod | 0.186666667 | 0.08 | 0.373333333 |
| AT5G19550.1 | i1_LQ_HNbud_c34171/f1p2/1698 | 81.89 | 403 | AAT2 | Photoperiod | 15.98333333 | 17.79 | 18.39666667 |
| AT5G19550.1 | i1_LQ_HNbud_c53253/f1p2/1490 | 81.89 | 403 | AAT2 | Photoperiod | #N/A | #N/A | #N/A |
| AT5G48300.1 | i1_HQ_HNbud_c128399/f2p3/1737 | 90.27 | 524 | ADG1 | Photoperiod | 0 | 0 | 0 |
| AT5G48300.1 | i1_LQ_HNbud_c103734/f1p15/1867 | 86.95 | 521 | ADG1 | Photoperiod | #N/A | #N/A | #N/A |
| AT5G48300.1 | i1_LQ_HNbud_c154640/f1p19/1962 | 86.95 | 521 | ADG1 | Photoperiod | 112.9966667 | 80.12333333 | 102.9666667 |
| AT5G48300.1 | i1_LQ_HNbud_c171131/f1p19/1933 | 86.95 | 521 | ADG1 | Photoperiod | 8.753333333 | 15.21 | 19.07666667 |
| AT5G48300.1 | i1_LQ_HNbud_c38609/f1p18/1783 | 86.95 | 521 | ADG1 | Photoperiod | #N/A | #N/A | #N/A |
| AT5G48300.1 | i1_LQ_HNbud_c68462/f1p17/1809 | 86.95 | 521 | ADG1 | Photoperiod | #N/A | #N/A | #N/A |
| AT5G48300.1 | i1_LQ_HNbud_c68503/f1p8/1807 | 86.95 | 521 | ADG1 | Photoperiod | #N/A | #N/A | #N/A |
| AT5G48300.1 | i1_LQ_HNbud_c73109/f1p16/1842 | 86.95 | 521 | ADG1 | Photoperiod | #N/A | #N/A | #N/A |
| AT5G48300.1 | i1_LQ_HNbud_c88830/f1p14/1823 | 86.95 | 521 | ADG1 | Photoperiod | #N/A | #N/A | #N/A |
| AT5G48300.1 | i2_LQ_HNbud_c36268/f1p99/2291 | 86.95 | 521 | ADG1 | Photoperiod | 0 | 0 | 0.036666667 |
| AT1G18450.1 | i1_LQ_HNbud_c68778/f1p1/1606 | 93.67 | 442 | ARP4 | Photoperiod | 0 | 0 | 0 |
| AT1G18450.1 | i1_LQ_HNbud_c133970/f1p3/1765 | 76.29 | 447 | ARP4 | Photoperiod | 1.36 | 6.276666667 | 3.78 |
| AT1G18450.1 | i1_HQ_HNbud_c26494/f6p3/1790 | 76.06 | 447 | ARP4 | Photoperiod | 10.66333333 | 11.22333333 | 8.386666667 |
| AT1G18450.1 | i1_LQ_HNbud_c68543/f1p3/1656 | 76.06 | 447 | ARP4 | Photoperiod | #N/A | #N/A | #N/A |
| AT1G18450.1 | i1_HQ_HNbud_c2570/f7p3/1739 | 75.73 | 445 | ARP4 | Photoperiod | #N/A | #N/A | #N/A |
| AT1G18450.1 | i1_HQ_HNbud_c83790/f4p3/1863 | 75.73 | 445 | ARP4 | Photoperiod | 3.23 | 9.213333333 | 8.313333333 |
| AT1G18450.1 | i1_LQ_HNbud_c89779/f1p3/1899 | 75.73 | 445 | ARP4 | Photoperiod | 7.913333333 | 11.58 | 10.48 |
| AT1G18450.1 | i1_LQ_HNbud_c54591/f1p1/1794 | 72.04 | 447 | ARP4 | Photoperiod | #N/A | #N/A | #N/A |
| AT1G18450.1 | i1_LQ_HNbud_c36588/f1p3/1734 | 70.92 | 196 | ARP4 | Photoperiod | #N/A | #N/A | #N/A |
| AT1G18450.1 | i2_LQ_HNbud_c22597/f1p2/2218 | 69.21 | 445 | ARP4 | Photoperiod | 0 | 0 | 0 |
| AT1G59940.1 | i1_LQ_HNbud_c116615/f1p2/1014 | 60.87 | 207 | ARR3 | Photoperiod | 0.873333333 | 4.796666667 | 6.086666667 |
| AT5G28450.1 | i0_HQ_HNbud_c12420/f4p8/976 | 91.26 | 103 | AT5G28450 | Photoperiod | #N/A | #N/A | #N/A |
| AT5G28450.1 | i0_LQ_HNbud_c2106/f4p4/1015 | 91.26 | 103 | AT5G28450 | Photoperiod | #N/A | #N/A | #N/A |
| AT5G28450.1 | i1_LQ_HNbud_c277721/f1p7/1014 | 91.26 | 103 | AT5G28450 | Photoperiod | #N/A | #N/A | #N/A |
| AT5G28450.1 | i1_LQ_HNbud_c280307/f1p7/1006 | 91.26 | 103 | AT5G28450 | Photoperiod | #N/A | #N/A | #N/A |
| AT5G28450.1 | i1_LQ_HNbud_c280351/f1p7/1017 | 91.26 | 103 | AT5G28450 | Photoperiod | #N/A | #N/A | #N/A |
| AT5G28450.1 | i1_LQ_HNbud_c28768/f1p6/1068 | 91.26 | 103 | AT5G28450 | Photoperiod | #N/A | #N/A | #N/A |
| AT5G28450.1 | i1_LQ_HNbud_c60645/f1p2/1025 | 91.26 | 103 | AT5G28450 | Photoperiod | 0.023333333 | 0 | 0 |
| AT5G28450.1 | i0_HQ_HNbud_c46239/f2p1/959 | 90.29 | 103 | AT5G28450 | Photoperiod | #N/A | #N/A | #N/A |
| AT5G28450.1 | i1_HQ_HNbud_c101840/f27p7/1056 | 90.29 | 103 | AT5G28450 | Photoperiod | 35.52 | 50.71 | 26.15666667 |
| AT5G28450.1 | i1_LQ_HNbud_c19042/f1p6/1136 | 91.26 | 103 | AT5G28450 | Photoperiod | 40.9 | 47.95 | 22.42666667 |
| AT2G46020.1 | i6_HQ_HNbud_c214/f17p0/6963 | 90.54 | 856 | ATBRM | Photoperiod | #N/A | #N/A | #N/A |
| AT2G46020.1 | i6_HQ_HNbud_c322/f4p0/7080 | 90.54 | 856 | ATBRM | Photoperiod | 0 | 0 | 0 |
| AT2G46020.1 | i6_HQ_HNbud_c332/f2p0/6907 | 89.61 | 77 | ATBRM | Photoperiod | #N/A | #N/A | #N/A |
| AT2G46020.1 | i6_HQ_HNbud_c3420/f5p0/6876 | 89.39 | 858 | ATBRM | Photoperiod | #N/A | #N/A | #N/A |
| AT2G46020.1 | i6_HQ_HNbud_c3456/f17p0/6954 | 89.39 | 858 | ATBRM | Photoperiod | 0 | 0 | 0 |
| AT2G46020.1 | i6_LQ_HNbud_c1812/f1p2/6478 | 83.62 | 763 | ATBRM | Photoperiod | #N/A | #N/A | #N/A |
| AT2G46020.1 | i4_LQ_HNbud_c385/f1p0/4733 | 75.2 | 1343 | ATBRM | Photoperiod | 16.22 | 17.19666667 | 15.13666667 |
| AT2G46020.1 | i7_LQ_HNbud_c552/f1p0/7110 | 74.86 | 1261 | ATBRM | Photoperiod | 0 | 0 | 0 |
| AT2G46020.1 | i6_LQ_HNbud_c1927/f1p1/7053 | 73.66 | 243 | ATBRM | Photoperiod | 0.003333333 | 0 | 0.003333333 |
| AT2G46020.1 | i5_LQ_HNbud_c1091/f1p3/5294 | 71.11 | 1509 | ATBRM | Photoperiod | 17.16333333 | 15.96666667 | 15.33333333 |
| AT5G24930.1 | i1_LQ_HNbud_c107688/f1p0/1380 | 84.66 | 365 | ATCOL4 | Photoperiod | 0 | 0 | 0 |
| AT5G24930.1 | i1_LQ_HNbud_c112244/f1p0/1772 | 83.79 | 364 | ATCOL4 | Photoperiod | 0 | 0 | 0 |
| AT5G24930.1 | i1_LQ_HNbud_c130667/f1p0/1505 | 80.18 | 338 | ATCOL4 | Photoperiod | 0 | 0 | 0 |
| AT5G24930.1 | i1_HQ_HNbud_c107758/f2p14/1419 | 58.47 | 378 | ATCOL4 | Photoperiod | #N/A | #N/A | #N/A |
| AT5G24930.1 | i1_HQ_HNbud_c127026/f2p11/1376 | 58.47 | 378 | ATCOL4 | Photoperiod | #N/A | #N/A | #N/A |
| AT5G24930.1 | i1_HQ_HNbud_c167879/f4p19/1491 | 58.47 | 378 | ATCOL4 | Photoperiod | 34.40333333 | 83.58333333 | 62.68 |
| AT5G24930.1 | i1_HQ_HNbud_c220871/f2p14/1357 | 58.47 | 378 | ATCOL4 | Photoperiod | #N/A | #N/A | #N/A |
| AT5G24930.1 | i1_LQ_HNbud_c103446/f1p10/1416 | 58.47 | 378 | ATCOL4 | Photoperiod | #N/A | #N/A | #N/A |
| AT5G24930.1 | i1_LQ_HNbud_c75179/f1p12/1377 | 58.47 | 378 | ATCOL4 | Photoperiod | #N/A | #N/A | #N/A |
| AT5G24930.1 | i1_LQ_HNbud_c75258/f1p15/1495 | 58.47 | 378 | ATCOL4 | Photoperiod | 0.753333333 | 0.336666667 | 0.186666667 |
| AT2G04030.1 | i2_HQ_HNbud_c24566/f2p11/2267 | 84.77 | 696 | ATHSP90.5 | Photoperiod | #N/A | #N/A | #N/A |
| AT2G04030.1 | i2_HQ_HNbud_c16610/f26p14/2576 | 80.67 | 802 | ATHSP90.5 | Photoperiod | #N/A | #N/A | #N/A |
| AT2G04030.1 | i2_HQ_HNbud_c1745/f8p14/2674 | 80.55 | 802 | ATHSP90.5 | Photoperiod | 130.2033333 | 88.37666667 | 86.85 |
| AT2G04030.1 | i2_HQ_HNbud_c33459/f2p13/2678 | 80.55 | 802 | ATHSP90.5 | Photoperiod | #N/A | #N/A | #N/A |
| AT2G04030.1 | i2_LQ_HNbud_c11498/f1p11/2642 | 80.55 | 802 | ATHSP90.5 | Photoperiod | #N/A | #N/A | #N/A |
| AT2G04030.1 | i2_LQ_HNbud_c23181/f1p17/2572 | 80.55 | 802 | ATHSP90.5 | Photoperiod | #N/A | #N/A | #N/A |
| AT2G04030.1 | i2_LQ_HNbud_c26562/f1p13/2633 | 80.55 | 802 | ATHSP90.5 | Photoperiod | 0 | 0.086666667 | 0 |
| AT2G04030.1 | i2_LQ_HNbud_c58780/f1p14/2966 | 80.55 | 802 | ATHSP90.5 | Photoperiod | 6.52 | 7.73 | 6.853333333 |
| AT2G04030.1 | i2_HQ_HNbud_c48770/f9p2/2764 | 69.65 | 705 | ATHSP90.5 | Photoperiod | 6.34 | 9.186666667 | 5.856666667 |
| AT2G04030.1 | i2_LQ_HNbud_c63716/f1p1/2752 | 69.65 | 705 | ATHSP90.5 | Photoperiod | 56.31 | 32.20666667 | 37.28666667 |
| AT4G31500.1 | i1_LQ_HNbud_c42176/f1p1/1815 | 96.79 | 468 | ATR4 | Photoperiod | 0 | 0 | 0 |
| AT4G31500.1 | i1_HQ_HNbud_c58264/f6p1/1715 | 95.99 | 499 | ATR4 | Photoperiod | #N/A | #N/A | #N/A |
| AT3G30180.1 | i2_LQ_HNbud_c8893/f1p1/2076 | 69.25 | 465 | BR6OX2 | Photoperiod | 8.38 | 1.406666667 | 2.986666667 |
| AT3G30180.1 | i1_LQ_HNbud_c11776/f1p2/1241 | 68.6 | 293 | BR6OX2 | Photoperiod | #N/A | #N/A | #N/A |
| AT3G30180.1 | i1_LQ_HNbud_c130800/f1p2/1497 | 69.25 | 465 | BR6OX2 | Photoperiod | 26.72333333 | 4.596666667 | 6.676666667 |
| AT5G37780.1 | i0_HQ_HNbud_c13325/f7p2/951 | 97.32 | 149 | CAM1 | Photoperiod | 434.4433333 | 202.7566667 | 186.9766667 |
| AT5G37780.1 | i0_HQ_HNbud_c53970/f2p2/960 | 97.32 | 149 | CAM1 | Photoperiod | 51.76 | 101.74 | 110.26 |
| AT5G37780.1 | i0_LQ_HNbud_c121148/f6p0/565 | 97.32 | 149 | CAM1 | Photoperiod | #N/A | #N/A | #N/A |
| AT5G37780.1 | i0_LQ_HNbud_c48849/f1p0/770 | 97.32 | 149 | CAM1 | Photoperiod | #N/A | #N/A | #N/A |
| AT5G37780.1 | i0_LQ_HNbud_c79108/f1p0/716 | 97.32 | 149 | CAM1 | Photoperiod | 66.91333333 | 80.99333333 | 72.12 |
| AT5G37780.1 | i0_LQ_HNbud_c98197/f53p0/804 | 97.32 | 149 | CAM1 | Photoperiod | 407.0566667 | 192.59 | 205.5566667 |
| AT5G37780.1 | i1_LQ_HNbud_c129624/f1p15/1947 | 97.32 | 149 | CAM1 | Photoperiod | 0.096666667 | 0.236666667 | 0.366666667 |
| AT5G37780.1 | i0_LQ_HNbud_c100184/f1p6/720 | 96.64 | 149 | CAM1 | Photoperiod | 357.8233333 | 502.7533333 | 544.97 |
| AT5G37780.1 | i0_LQ_HNbud_c99606/f2p0/732 | 96.64 | 149 | CAM1 | Photoperiod | #N/A | #N/A | #N/A |
| AT5G37780.1 | i3_LQ_HNbud_c12071/f1p36/3207 | 96.64 | 149 | CAM1 | Photoperiod | 1.016666667 | 0.666666667 | 0.806666667 |
| Os08t0157600_01 | i2_LQ_HNbud_c1725/f3p0/2269 | 58.86 | 175 | CCA1/LHY/OsCCA1 | Photoperiod | 0.163333333 | 6.39 | 5.523333333 |
| Os08t0157600_01 | i2_LQ_HNbud_c41573/f1p0/2271 | 58.86 | 175 | CCA1/LHY/OsCCA1 | Photoperiod | 0.59 | 5.25 | 2.916666667 |
| Os08t0157600_01 | i2_LQ_HNbud_c35178/f2p3/2529 | 58.76 | 177 | CCA1/LHY/OsCCA1 | Photoperiod | 0.53 | 7.693333333 | 4.66 |
| Os08t0157600_01 | i2_LQ_HNbud_c53369/f1p11/2426 | 58.76 | 177 | CCA1/LHY/OsCCA1 | Photoperiod | 0.393333333 | 2.683333333 | 2.47 |
| AT3G47500.1 | i1_HQ_HNbud_c49365/f3p1/1430 | 75.28 | 453 | CDF3 | Photoperiod | 0 | 0 | 0 |
| AT2G34140.1 | i2_LQ_HNbud_c50500/f1p0/2066 | 50 | 122 | CDF4 | Photoperiod | 0.086666667 | 0.29 | 0.343333333 |
| AT1G44446.1 | i2_LQ_HNbud_c19282/f1p1/2066 | 92.39 | 473 | CH1 | Photoperiod | 0 | 0 | 0 |
| AT1G44446.1 | i2_LQ_HNbud_c25027/f1p0/2111 | 92.14 | 471 | CH1 | Photoperiod | 0 | 0 | 0 |
| AT1G44446.1 | i1_HQ_HNbud_c91383/f2p2/1950 | 91.88 | 517 | CH1 | Photoperiod | #N/A | #N/A | #N/A |
| AT1G44446.1 | i1_LQ_HNbud_c49243/f1p2/1999 | 91.84 | 515 | CH1 | Photoperiod | 0 | 0 | 0 |
| AT1G44446.1 | i1_HQ_HNbud_c111424/f3p2/1901 | 91.6 | 536 | CH1 | Photoperiod | #N/A | #N/A | #N/A |
| AT1G44446.1 | i2_LQ_HNbud_c15893/f1p0/2038 | 91.6 | 536 | CH1 | Photoperiod | 0 | 0 | 0 |
| AT1G44446.1 | i1_HQ_HNbud_c7817/f2p0/1502 | 74.93 | 375 | CH1 | Photoperiod | 4.026666667 | 5.736666667 | 4.273333333 |
| AT1G44446.1 | i1_HQ_HNbud_c224372/f2p0/1868 | 74.62 | 532 | CH1 | Photoperiod | #N/A | #N/A | #N/A |
| AT1G44446.1 | i1_LQ_HNbud_c52623/f1p0/1885 | 74.62 | 532 | CH1 | Photoperiod | #N/A | #N/A | #N/A |
| AT1G44446.1 | i1_LQ_HNbud_c205444/f2p0/1947 | 74.25 | 532 | CH1 | Photoperiod | 7.483333333 | 13.15333333 | 10.44 |
| AT5G41790.1 | i2_LQ_HNbud_c55326/f1p1/2969 | 61.97 | 476 | CIP1 | Photoperiod | 0 | 0 | 0 |
| AT5G47080.1 | i1_HQ_HNbud_c201992/f2p8/1353 | 79.59 | 294 | CKB1 | Photoperiod | #N/A | #N/A | #N/A |
| AT5G47080.1 | i1_HQ_HNbud_c264498/f25p8/1512 | 79.59 | 294 | CKB1 | Photoperiod | 43.01333333 | 41.55666667 | 41.89666667 |
| AT5G47080.1 | i1_HQ_HNbud_c264952/f18p8/1380 | 79.59 | 294 | CKB1 | Photoperiod | #N/A | #N/A | #N/A |
| AT5G47080.1 | i1_LQ_HNbud_c12441/f1p9/1358 | 79.59 | 294 | CKB1 | Photoperiod | #N/A | #N/A | #N/A |
| AT5G47080.1 | i1_LQ_HNbud_c155435/f1p6/1466 | 79.59 | 294 | CKB1 | Photoperiod | #N/A | #N/A | #N/A |
| AT5G47080.1 | i1_LQ_HNbud_c22399/f1p7/1573 | 79.59 | 294 | CKB1 | Photoperiod | 0.043333333 | 0 | 0 |
| AT5G47080.1 | i1_LQ_HNbud_c248711/f1p7/1350 | 79.59 | 294 | CKB1 | Photoperiod | #N/A | #N/A | #N/A |
| AT5G47080.1 | i1_LQ_HNbud_c35211/f1p8/1470 | 79.59 | 294 | CKB1 | Photoperiod | #N/A | #N/A | #N/A |
| AT5G47080.1 | i1_LQ_HNbud_c50993/f1p7/1467 | 79.59 | 294 | CKB1 | Photoperiod | #N/A | #N/A | #N/A |
| AT5G47080.1 | i1_HQ_HNbud_c106606/f2p9/1560 | 79.59 | 294 | CKB1 | Photoperiod | 41.53666667 | 8.553333333 | 6.123333333 |
| AT3G04680.1 | i2_LQ_HNbud_c20875/f1p0/2103 | 72.12 | 269 | CLPS3 | Photoperiod | 0.036666667 | 0.026666667 | 0.133333333 |
| AT3G04680.1 | i1_HQ_HNbud_c145430/f22p8/1562 | 68.69 | 444 | CLPS3 | Photoperiod | 24.83666667 | 25.87666667 | 24.14666667 |
| AT3G04680.1 | i1_HQ_HNbud_c146333/f7p8/1517 | 68.47 | 444 | CLPS3 | Photoperiod | #N/A | #N/A | #N/A |
| AT3G04680.1 | i1_HQ_HNbud_c263581/f4p8/1636 | 68.47 | 444 | CLPS3 | Photoperiod | #N/A | #N/A | #N/A |
| AT3G04680.1 | i1_LQ_HNbud_c108174/f1p6/1670 | 68.47 | 444 | CLPS3 | Photoperiod | 0 | 0.283333333 | 0 |
| AT3G04680.1 | i1_LQ_HNbud_c150973/f1p7/1663 | 68.47 | 444 | CLPS3 | Photoperiod | 8.503333333 | 18.70333333 | 16.71333333 |
| AT3G04680.1 | i1_LQ_HNbud_c267521/f1p8/1564 | 68.47 | 444 | CLPS3 | Photoperiod | #N/A | #N/A | #N/A |
| AT3G04680.1 | i1_LQ_HNbud_c33945/f1p8/1615 | 68.47 | 444 | CLPS3 | Photoperiod | #N/A | #N/A | #N/A |
| AT3G04680.1 | i1_LQ_HNbud_c34993/f1p8/1775 | 68.02 | 444 | CLPS3 | Photoperiod | 1.223333333 | 1.72 | 1.913333333 |
| AT3G04680.1 | i1_LQ_HNbud_c86447/f1p8/1826 | 68.02 | 444 | CLPS3 | Photoperiod | 0.666666667 | 0.45 | 0.596666667 |
| AT5G37770.1 | i0_LQ_HNbud_c67684/f1p0/748 | 61.07 | 149 | CML24 | Photoperiod | 4.656666667 | 5.773333333 | 6.923333333 |
| AT5G37770.1 | i0_HQ_HNbud_c7193/f3p1/991 | 51.33 | 150 | CML24 | Photoperiod | 17.03 | 30.52666667 | 28.93666667 |
| AT5G37770.1 | i0_HQ_HNbud_c5493/f2p0/938 | 50.99 | 151 | CML24 | Photoperiod | #N/A | #N/A | #N/A |
| AT5G37770.1 | i1_LQ_HNbud_c242379/f1p5/1042 | 50.99 | 151 | CML24 | Photoperiod | 9.086666667 | 21.20333333 | 32.73333333 |
| AT5G15850.1 | i1_LQ_HNbud_c246352/f5p6/1385 | 51.94 | 310 | COL1 | Photoperiod | #N/A | #N/A | #N/A |
| AT3G02380.1 | i1_LQ_HNbud_c75514/f1p4/1347 | 59.01 | 383 | COL2 | Photoperiod | #N/A | #N/A | #N/A |
| AT3G02380.1 | i1_HQ_HNbud_c243873/f12p6/1451 | 58.96 | 385 | COL2 | Photoperiod | 52.25666667 | 24.18333333 | 16.61333333 |
| AT3G02380.1 | i1_LQ_HNbud_c36726/f1p4/1281 | 58.96 | 385 | COL2 | Photoperiod | #N/A | #N/A | #N/A |
| AT3G02380.1 | i1_HQ_HNbud_c37538/f2p4/1333 | 58.7 | 385 | COL2 | Photoperiod | 0.016666667 | 0.04 | 0 |
| AT3G02380.1 | i1_HQ_HNbud_c7853/f9p5/1191 | 57.44 | 390 | COL2 | Photoperiod | 27.62333333 | 15.23333333 | 12.11666667 |
| AT3G02380.1 | i1_LQ_HNbud_c12325/f1p5/1137 | 57.14 | 336 | COL2 | Photoperiod | #N/A | #N/A | #N/A |
| AT5G57660.1 | i1_LQ_HNbud_c180058/f1p3/1235 | 81.75 | 263 | COL5 | Photoperiod | 0 | 0 | 0 |
| AT5G57660.1 | i1_LQ_HNbud_c33880/f1p3/1237 | 81.75 | 263 | COL5 | Photoperiod | 0 | 0 | 0 |
| AT5G57660.1 | i1_LQ_HNbud_c52656/f1p3/1472 | 81.75 | 263 | COL5 | Photoperiod | 0 | 0 | 0 |
| AT5G57660.1 | i1_LQ_HNbud_c91177/f1p3/1430 | 81.75 | 263 | COL5 | Photoperiod | 0 | 0 | 0 |
| AT5G57660.1 | i1_LQ_HNbud_c251312/f1p3/1548 | 81.66 | 338 | COL5 | Photoperiod | 0 | 0 | 0 |
| AT5G57660.1 | i1_LQ_HNbud_c6871/f5p3/1425 | 81.66 | 338 | COL5 | Photoperiod | 0 | 0 | 0 |
| AT5G57660.1 | i1_LQ_HNbud_c19314/f1p3/1533 | 80.85 | 329 | COL5 | Photoperiod | 0 | 0 | 0 |
| AT5G57660.1 | i1_LQ_HNbud_c170194/f2p3/1499 | 79.29 | 338 | COL5 | Photoperiod | 0 | 0 | 0 |
| Os08t0249000_01 | i1_LQ_HNbud_c108459/f1p9/1496 | 60.98 | 41 | COL5 | Photoperiod | 2.68 | 0.786666667 | 0.776666667 |
| AT3G07650.1 | i1_LQ_HNbud_c227363/f1p0/1658 | 67.54 | 382 | COL9 | Photoperiod | 0 | 0 | 0 |
| AT3G07650.1 | i1_LQ_HNbud_c39779/f1p0/1640 | 55.67 | 203 | COL9 | Photoperiod | 0 | 0 | 0 |
| AT3G07650.1 | i1_LQ_HNbud_c103793/f2p1/1942 | 52.31 | 346 | COL9 | Photoperiod | 12.02333333 | 5.35 | 6.47 |
| AT3G07650.1 | i1_LQ_HNbud_c251477/f1p1/2324 | 52.31 | 346 | COL9 | Photoperiod | 0.443333333 | 0.106666667 | 0.08 |
| AT3G07650.1 | i1_HQ_HNbud_c21321/f5p1/1896 | 52.02 | 346 | COL9 | Photoperiod | #N/A | #N/A | #N/A |
| AT4G37580.1 | i1_LQ_HNbud_c131302/f1p0/1804 | 74.32 | 405 | COP3 | Photoperiod | 0.033333333 | 0 | 0.01 |
| AT4G37580.1 | i1_LQ_HNbud_c199533/f1p0/1508 | 74.32 | 405 | COP3 | Photoperiod | 0 | 0 | 0 |
| AT4G14110.1 | i0_HQ_HNbud_c600/f19p0/887 | 65.99 | 197 | COP9 | Photoperiod | 14.11 | 34.86666667 | 40.27666667 |
| AT4G14110.1 | i0_LQ_HNbud_c13492/f1p0/940 | 65.99 | 197 | COP9 | Photoperiod | 0.706666667 | 0.85 | 1.396666667 |
| AT4G14110.1 | i1_LQ_HNbud_c45026/f1p3/1045 | 65.48 | 197 | COP9 | Photoperiod | 3.76 | 3.84 | 4.163333333 |
| AT4G14110.1 | i1_LQ_HNbud_c241219/f1p3/1024 | 65.1 | 149 | COP9 | Photoperiod | 38.1 | 35.57 | 35.34666667 |
| AT4G14110.1 | i0_LQ_HNbud_c2632/f1p3/741 | 64.47 | 197 | COP9 | Photoperiod | 6.853333333 | 9.21 | 10.33 |
| AT4G14110.1 | i0_LQ_HNbud_c53261/f1p16/909 | 63.96 | 197 | COP9 | Photoperiod | 13.73 | 9.21 | 8.086666667 |
| AT1G26830.1 | i2_LQ_HNbud_c5228/f1p3/2529 | 92.62 | 732 | CUL3 | Photoperiod | 0 | 0 | 0 |
| AT1G26830.1 | i2_HQ_HNbud_c7194/f2p2/2774 | 83.38 | 734 | CUL3 | Photoperiod | #N/A | #N/A | #N/A |
| AT1G26830.1 | i2_HQ_HNbud_c13197/f7p2/2758 | 83.08 | 733 | CUL3 | Photoperiod | 4.813333333 | 7.963333333 | 8.333333333 |
| AT1G26830.1 | i2_HQ_HNbud_c43770/f2p2/2769 | 83.08 | 733 | CUL3 | Photoperiod | #N/A | #N/A | #N/A |
| AT1G26830.1 | i2_LQ_HNbud_c54001/f1p4/2743 | 83.08 | 733 | CUL3 | Photoperiod | 5.656666667 | 8.386666667 | 7.25 |
| AT1G26830.1 | i2_LQ_HNbud_c43332/f1p2/2867 | 82.97 | 734 | CUL3 | Photoperiod | 6.583333333 | 4.81 | 4.27 |
| AT1G26830.1 | i2_HQ_HNbud_c4749/f8p6/2821 | 82.83 | 734 | CUL3 | Photoperiod | #N/A | #N/A | #N/A |
| AT1G26830.1 | i2_LQ_HNbud_c40449/f1p2/2380 | 82.83 | 734 | CUL3 | Photoperiod | #N/A | #N/A | #N/A |
| AT1G26830.1 | i2_LQ_HNbud_c9933/f1p2/2899 | 82.83 | 734 | CUL3 | Photoperiod | 7.45 | 7.84 | 7.476666667 |
| AT1G26830.1 | i3_LQ_HNbud_c15405/f1p2/3009 | 82.67 | 733 | CUL3 | Photoperiod | 0.203333333 | 0.483333333 | 0.406666667 |
| AT5G46210.1 | i3_LQ_HNbud_c9451/f1p0/3702 | 83.78 | 746 | CUL4 | Photoperiod | 19.92333333 | 13.61333333 | 14.00333333 |
| AT5G46210.1 | i2_LQ_HNbud_c7496/f1p5/2777 | 83.68 | 766 | CUL4 | Photoperiod | 12.62 | 9.023333333 | 9 |
| AT5G46210.1 | i2_HQ_HNbud_c70553/f18p5/2861 | 81.51 | 795 | CUL4 | Photoperiod | 19.43333333 | 15.96 | 15.15666667 |
| AT5G46210.1 | i2_LQ_HNbud_c10736/f1p5/2749 | 81.51 | 795 | CUL4 | Photoperiod | #N/A | #N/A | #N/A |
| AT5G46210.1 | i2_LQ_HNbud_c22723/f1p8/2818 | 81.51 | 795 | CUL4 | Photoperiod | #N/A | #N/A | #N/A |
| AT5G46210.1 | i2_HQ_HNbud_c70526/f15p5/2737 | 81.38 | 795 | CUL4 | Photoperiod | #N/A | #N/A | #N/A |
| AT5G46210.1 | i2_LQ_HNbud_c39823/f1p6/2709 | 81.38 | 795 | CUL4 | Photoperiod | #N/A | #N/A | #N/A |
| AT5G46210.1 | i2_LQ_HNbud_c42578/f1p5/2693 | 81.38 | 795 | CUL4 | Photoperiod | #N/A | #N/A | #N/A |
| AT5G46210.1 | i2_LQ_HNbud_c27564/f1p5/2639 | 81.26 | 795 | CUL4 | Photoperiod | #N/A | #N/A | #N/A |
| AT5G46210.1 | i2_LQ_HNbud_c36930/f1p9/2822 | 81.26 | 795 | CUL4 | Photoperiod | #N/A | #N/A | #N/A |
| AT2G26710.1 | i2_LQ_HNbud_c23716/f1p0/2058 | 75.92 | 519 | CYP72B1 | Photoperiod | 0.466666667 | 0.746666667 | 0.643333333 |
| AT2G26710.1 | i1_HQ_HNbud_c35255/f7p1/1873 | 75.53 | 519 | CYP72B1 | Photoperiod | 3.383333333 | 5.926666667 | 4.826666667 |
| AT2G26710.1 | i2_LQ_HNbud_c56700/f1p5/2303 | 73.63 | 421 | CYP72B1 | Photoperiod | 0.033333333 | 0.033333333 | 0.036666667 |
| AT4G03400.1 | i1_HQ_HNbud_c7731/f4p2/1926 | 89.78 | 587 | DFL2 | Photoperiod | 0 | 0 | 0 |
| AT4G03400.1 | i1_LQ_HNbud_c88442/f1p0/1926 | 71.23 | 584 | DFL2 | Photoperiod | 0 | 0 | 0.01 |
| AT4G03400.1 | i1_LQ_HNbud_c105168/f1p0/1896 | 70.21 | 584 | DFL2 | Photoperiod | #N/A | #N/A | #N/A |
| AT4G03400.1 | i2_LQ_HNbud_c18961/f1p5/2412 | 67.48 | 409 | DFL2 | Photoperiod | 0 | 0 | 0 |
| AT4G03400.1 | i2_LQ_HNbud_c28640/f1p0/2096 | 71.23 | 584 | DFL2 | Photoperiod | 0.086666667 | 0.736666667 | 1.74 |
| AT4G03400.1 | i2_LQ_HNbud_c62348/f1p0/2014 | 70.21 | 584 | DFL2 | Photoperiod | 0.046666667 | 1.766666667 | 2.246666667 |
| AT4G03400.1 | i1_LQ_HNbud_c110593/f1p1/2009 | 52.97 | 589 | DFL2 | Photoperiod | 0.106666667 | 0.056666667 | 0 |
| AT4G03400.1 | i1_HQ_HNbud_c128268/f3p1/1957 | 52.8 | 589 | DFL2 | Photoperiod | #N/A | #N/A | #N/A |
| AT4G03400.1 | i1_LQ_HNbud_c69864/f1p1/2080 | 52.8 | 589 | DFL2 | Photoperiod | 17.44333333 | 5.116666667 | 4.806666667 |
| AT4G03400.1 | i2_LQ_HNbud_c39708/f1p0/2117 | 52.8 | 589 | DFL2 | Photoperiod | 15.86333333 | 5.77 | 7.093333333 |
| AT4G22140.1 | i0_LQ_HNbud_c3811/f1p0/813 | 81.08 | 111 | EBS | Photoperiod | 6.04 | 17.36 | 11.40666667 |
| AT4G22140.1 | i1_HQ_HNbud_c159458/f2p4/1035 | 80.65 | 217 | EBS | Photoperiod | #N/A | #N/A | #N/A |
| AT4G22140.1 | i1_HQ_HNbud_c144182/f21p11/993 | 80.53 | 113 | EBS | Photoperiod | 26.61 | 55.55333333 | 41.84 |
| AT4G22140.1 | i1_HQ_HNbud_c9465/f12p2/1139 | 80.18 | 217 | EBS | Photoperiod | #N/A | #N/A | #N/A |
| AT4G22140.1 | i1_LQ_HNbud_c92892/f1p2/1176 | 80.18 | 217 | EBS | Photoperiod | 60.59333333 | 47.02 | 41.20666667 |
| AT4G22140.1 | i0_HQ_HNbud_c38060/f11p0/985 | 73.58 | 193 | EBS | Photoperiod | 41.34 | 24.35666667 | 19.03666667 |
| AT4G22140.1 | i1_LQ_HNbud_c105806/f1p0/1168 | 72.14 | 201 | EBS | Photoperiod | 33.72 | 23.17666667 | 17.73 |
| AT2G25930.1 | i2_LQ_HNbud_c20333/f1p1/2165 | 56.33 | 671 | ELF3 | Photoperiod | 0 | 0 | 0 |
| AT1G17455.1 | i0_HQ_HNbud_c1322/f7p2/811 | 77 | 100 | ELF4-L4 | Photoperiod | 25.91666667 | 29.70666667 | 48.93666667 |
| AT1G17455.1 | i0_HQ_HNbud_c19322/f2p0/753 | 71.93 | 114 | ELF4-L4 | Photoperiod | 4.876666667 | 6.96 | 6.81 |
| AT1G17455.1 | i0_LQ_HNbud_c84649/f1p0/905 | 71.93 | 114 | ELF4-L4 | Photoperiod | #N/A | #N/A | #N/A |
| AT1G17455.1 | i1_LQ_HNbud_c201545/f1p0/1238 | 71.93 | 114 | ELF4-L4 | Photoperiod | 0.973333333 | 1.393333333 | 1.263333333 |
| AT5G62640.1 | i1_LQ_HNbud_c32902/f1p0/1216 | 85.71 | 119 | ELF5 | Photoperiod | 0 | 0 | 0 |
| AT5G62640.1 | i1_HQ_HNbud_c267439/f2p4/1864 | 73.22 | 183 | ELF5 | Photoperiod | #N/A | #N/A | #N/A |
| AT5G62640.1 | i2_LQ_HNbud_c59508/f1p1/2066 | 73.22 | 183 | ELF5 | Photoperiod | 4.9 | 6.416666667 | 6.5 |
| AT5G62640.1 | i1_HQ_HNbud_c264751/f7p4/1982 | 54.76 | 557 | ELF5 | Photoperiod | #N/A | #N/A | #N/A |
| AT5G62640.1 | i1_LQ_HNbud_c69372/f1p4/1782 | 54.76 | 557 | ELF5 | Photoperiod | #N/A | #N/A | #N/A |
| AT5G62640.1 | i1_LQ_HNbud_c72604/f1p3/1933 | 54.76 | 557 | ELF5 | Photoperiod | 5.456666667 | 4.446666667 | 6.243333333 |
| AT5G62640.1 | i2_LQ_HNbud_c15382/f1p1/2034 | 54.76 | 557 | ELF5 | Photoperiod | #N/A | #N/A | #N/A |
| AT5G62640.1 | i2_LQ_HNbud_c5199/f1p1/2114 | 54.76 | 557 | ELF5 | Photoperiod | 3.15 | 6.903333333 | 5.076666667 |
| AT5G62640.1 | i2_LQ_HNbud_c73890/f1p1/2001 | 51.95 | 385 | ELF5 | Photoperiod | #N/A | #N/A | #N/A |
| Os03t0151300_01 | i4_HQ_HNbud_c104/f4p0/4699 | 70.23 | 131 | ELF6 | Photoperiod | 6.933333333 | 8.213333333 | 6.853333333 |
| Os03t0151300_01 | i4_LQ_HNbud_c4212/f1p2/4037 | 70.23 | 131 | ELF6 | Photoperiod | #N/A | #N/A | #N/A |
| Os03t0151300_01 | i4_LQ_HNbud_c733/f1p0/4394 | 70.23 | 131 | ELF6 | Photoperiod | #N/A | #N/A | #N/A |
| AT1G79730.1 | i1_HQ_HNbud_c2577/f3p3/1472 | 60.48 | 420 | ELF7 | Photoperiod | #N/A | #N/A | #N/A |
| AT1G79730.1 | i2_HQ_HNbud_c22571/f3p2/3000 | 58.66 | 479 | ELF7 | Photoperiod | #N/A | #N/A | #N/A |
| AT1G79730.1 | i2_LQ_HNbud_c13554/f1p4/2318 | 58.66 | 479 | ELF7 | Photoperiod | #N/A | #N/A | #N/A |
| AT1G79730.1 | i2_LQ_HNbud_c41825/f1p2/2658 | 58.66 | 479 | ELF7 | Photoperiod | #N/A | #N/A | #N/A |
| AT1G79730.1 | i3_HQ_HNbud_c15231/f2p0/3055 | 58.66 | 479 | ELF7 | Photoperiod | 4.213333333 | 2.123333333 | 1.68 |
| AT1G79730.1 | i3_LQ_HNbud_c18614/f1p3/3017 | 58.66 | 479 | ELF7 | Photoperiod | 15.17 | 12.77666667 | 11.67666667 |
| AT1G79730.1 | i2_HQ_HNbud_c7573/f2p2/2964 | 58.46 | 479 | ELF7 | Photoperiod | 5.086666667 | 5.063333333 | 4.493333333 |
| AT1G79730.1 | i2_LQ_HNbud_c20326/f1p2/2944 | 58.46 | 479 | ELF7 | Photoperiod | #N/A | #N/A | #N/A |
| AT1G79730.1 | i2_LQ_HNbud_c62419/f1p2/2938 | 58.46 | 479 | ELF7 | Photoperiod | #N/A | #N/A | #N/A |
| AT1G79730.1 | i3_LQ_HNbud_c18624/f1p3/3079 | 58.46 | 479 | ELF7 | Photoperiod | 10.32 | 10.45 | 9.543333333 |
| AT4G14690.1 | i0_HQ_HNbud_c30300/f2p3/810 | 55.1 | 196 | ELIP2 | Photoperiod | 1.623333333 | 12.76 | 26.96333333 |
| AT4G14690.1 | i0_LQ_HNbud_c22063/f1p0/822 | 55.1 | 196 | ELIP2 | Photoperiod | 0.593333333 | 8.92 | 27.16333333 |
| AT4G14690.1 | i0_LQ_HNbud_c22427/f1p24/939 | 55.61 | 196 | ELIP2 | Photoperiod | 0.14 | 0.386666667 | 0.283333333 |
| AT2G13540.1 | i2_LQ_HNbud_c6820/f1p2/2465 | 69.02 | 510 | ENS | Photoperiod | 1.773333333 | 3.97 | 4.416666667 |
| AT2G13540.1 | i2_LQ_HNbud_c1007/f13p2/2913 | 64.79 | 852 | ENS | Photoperiod | 3.273333333 | 8.03 | 10.57666667 |
| AT2G13540.1 | i2_HQ_HNbud_c14452/f4p2/2867 | 64.52 | 854 | ENS | Photoperiod | #N/A | #N/A | #N/A |
| AT2G13540.1 | i2_LQ_HNbud_c38374/f1p2/2928 | 64.52 | 854 | ENS | Photoperiod | 2.823333333 | 5.843333333 | 8.33 |
| AT2G13540.1 | i2_LQ_HNbud_c5850/f1p2/2835 | 64.52 | 854 | ENS | Photoperiod | 0 | 0 | 0 |
| AT2G13540.1 | i2_LQ_HNbud_c54672/f1p17/2608 | 64.09 | 763 | ENS | Photoperiod | #N/A | #N/A | #N/A |
| AT2G13540.1 | i2_LQ_HNbud_c33661/f1p4/2972 | 64.07 | 874 | ENS | Photoperiod | 2.723333333 | 6.833333333 | 6.553333333 |
| AT2G13540.1 | i3_LQ_HNbud_c14492/f1p0/3170 | 59.58 | 851 | ENS | Photoperiod | 1.553333333 | 3.326666667 | 3.636666667 |
| AT2G13540.1 | i1_LQ_HNbud_c68288/f1p4/1382 | 57.78 | 334 | ENS | Photoperiod | #N/A | #N/A | #N/A |
| AT2G13540.1 | i1_LQ_HNbud_c105650/f1p4/1146 | 52.78 | 252 | ENS | Photoperiod | #N/A | #N/A | #N/A |
| AT4G15090.1 | i2_LQ_HNbud_c21933/f1p0/2339 | 67.71 | 669 | FAR1 | Photoperiod | 1.8 | 3.933333333 | 3.99 |
| AT4G15090.1 | i2_LQ_HNbud_c4964/f1p0/2304 | 67.7 | 678 | FAR1 | Photoperiod | #N/A | #N/A | #N/A |
| AT4G15090.1 | i2_HQ_HNbud_c2253/f3p1/2885 | 64.1 | 830 | FAR1 | Photoperiod | #N/A | #N/A | #N/A |
| AT4G15090.1 | i2_LQ_HNbud_c35228/f1p42/2899 | 64.1 | 830 | FAR1 | Photoperiod | 2.896666667 | 6.04 | 6.35 |
| AT4G15090.1 | i2_LQ_HNbud_c57408/f1p1/2259 | 62.73 | 644 | FAR1 | Photoperiod | 0 | 0 | 0 |
| AT4G15090.1 | i3_LQ_HNbud_c1852/f2p5/3542 | 51.73 | 808 | FAR1 | Photoperiod | 0.48 | 3.143333333 | 4.73 |
| AT4G15090.1 | i2_HQ_HNbud_c2586/f5p13/3005 | 54.13 | 715 | FAR1 | Photoperiod | #N/A | #N/A | #N/A |
| AT4G15090.1 | i3_HQ_HNbud_c17245/f5p0/3128 | 53.39 | 826 | FAR1 | Photoperiod | 11.74333333 | 13.47333333 | 13.39 |
| AT4G15090.1 | i3_HQ_HNbud_c16772/f5p5/3128 | 53.04 | 839 | FAR1 | Photoperiod | 14.69333333 | 14.39 | 14.21 |
| AT4G15090.1 | i3_LQ_HNbud_c16517/f1p0/3026 | 51.47 | 814 | FAR1 | Photoperiod | 4.996666667 | 5.303333333 | 5.066666667 |
| AT2G21070.1 | i2_LQ_HNbud_c23950/f1p8/2479 | 63.07 | 287 | FIO1 | Photoperiod | #N/A | #N/A | #N/A |
| AT2G21070.1 | i1_HQ_HNbud_c129618/f2p4/1939 | 61.1 | 491 | FIO1 | Photoperiod | #N/A | #N/A | #N/A |
| AT2G21070.1 | i1_HQ_HNbud_c20963/f3p4/2013 | 61.1 | 491 | FIO1 | Photoperiod | #N/A | #N/A | #N/A |
| AT2G21070.1 | i2_LQ_HNbud_c30801/f1p0/2071 | 61.1 | 491 | FIO1 | Photoperiod | 5.426666667 | 9.456666667 | 9.003333333 |
| AT2G21070.1 | i1_LQ_HNbud_c180544/f1p4/1849 | 61.09 | 496 | FIO1 | Photoperiod | 2.52 | 1.89 | 2.176666667 |
| AT2G21070.1 | i1_LQ_HNbud_c152377/f1p4/1962 | 60.95 | 443 | FIO1 | Photoperiod | 0.276666667 | 0.86 | 0 |
| AT2G21070.1 | i1_HQ_HNbud_c26764/f2p4/1791 | 61.09 | 496 | FIO1 | Photoperiod | 2.54 | 2.006666667 | 0 |
| AT2G21070.1 | i1_LQ_HNbud_c50887/f1p4/1949 | 60.48 | 496 | FIO1 | Photoperiod | #N/A | #N/A | #N/A |
| AT2G21070.1 | i1_HQ_HNbud_c37999/f2p4/1841 | 61.09 | 496 | FIO1 | Photoperiod | 0.753333333 | 6.16 | 9.583333333 |
| AT2G21070.1 | i1_LQ_HNbud_c86210/f1p4/1965 | 59.75 | 477 | FIO1 | Photoperiod | 0.146666667 | 0.17 | 0.153333333 |
| AT1G68050.1 | i2_HQ_HNbud_c1354/f5p2/2349 | 82.71 | 584 | FKF1 | Photoperiod | 11.78333333 | 2.453333333 | 5.893333333 |
| AT5G63980.1 | i1_LQ_HNbud_c133739/f1p0/1337 | 86.02 | 329 | FRY1 | Photoperiod | 0 | 0 | 0 |
| AT5G63980.1 | i1_LQ_HNbud_c196434/f1p5/1384 | 80.69 | 347 | FRY1 | Photoperiod | #N/A | #N/A | #N/A |
| AT5G63980.1 | i2_LQ_HNbud_c42132/f1p1/2529 | 80.4 | 347 | FRY1 | Photoperiod | 2.663333333 | 5.893333333 | 7.22 |
| AT5G63980.1 | i1_HQ_HNbud_c120738/f17p5/1425 | 80.98 | 347 | FRY1 | Photoperiod | 5.7 | 22.55666667 | 30.94666667 |
| AT3G19040.1 | i1_LQ_HNbud_c201791/f1p1/1678 | 67.32 | 508 | HAF2 | Photoperiod | #N/A | #N/A | #N/A |
| AT3G19040.1 | i4_HQ_HNbud_c174/f3p0/4168 | 53.24 | 1174 | HAF2 | Photoperiod | 9.89 | 8.936666667 | 7.3 |
| AT3G19040.1 | i4_LQ_HNbud_c4299/f1p0/4046 | 53.2 | 1141 | HAF2 | Photoperiod | 1.306666667 | 1.173333333 | 0.993333333 |
| AT3G19040.1 | i3_LQ_HNbud_c7463/f1p2/3102 | 52.03 | 1082 | HAF2 | Photoperiod | #N/A | #N/A | #N/A |
| AT3G19040.1 | i4_LQ_HNbud_c3855/f1p0/4211 | 51.07 | 1173 | HAF2 | Photoperiod | 0 | 0.023333333 | 0 |
| AT3G19040.1 | i1_LQ_HNbud_c148480/f1p2/1964 | 50.08 | 661 | HAF2 | Photoperiod | #N/A | #N/A | #N/A |
| AT4G16780.1 | i1_LQ_HNbud_c174570/f1p3/1445 | 70.95 | 179 | HAT4 | Photoperiod | 26.45666667 | 34.64 | 24.50666667 |
| AT4G16780.1 | i1_LQ_HNbud_c112898/f1p0/1186 | 67.08 | 161 | HAT4 | Photoperiod | 0 | 0 | 0 |
| AT4G16780.1 | i1_LQ_HNbud_c230329/f1p3/1429 | 66.87 | 166 | HAT4 | Photoperiod | 0 | 0 | 0 |
| AT4G16780.1 | i1_LQ_HNbud_c251069/f1p5/1267 | 66.87 | 166 | HAT4 | Photoperiod | 13.09666667 | 14.76333333 | 15.53333333 |
| AT4G16780.1 | i1_LQ_HNbud_c66255/f1p5/1294 | 66.87 | 166 | HAT4 | Photoperiod | #N/A | #N/A | #N/A |
| AT4G16780.1 | i1_HQ_HNbud_c5486/f2p3/1632 | 65.16 | 244 | HAT4 | Photoperiod | 0.536666667 | 0.356666667 | 0.373333333 |
| AT4G16780.1 | i1_LQ_HNbud_c176879/f1p3/1604 | 65.16 | 244 | HAT4 | Photoperiod | 0.07 | 0 | 0.013333333 |
| AT4G16780.1 | i1_HQ_HNbud_c35137/f2p3/1207 | 61.13 | 301 | HAT4 | Photoperiod | #N/A | #N/A | #N/A |
| AT4G16780.1 | i1_HQ_HNbud_c5989/f11p3/1341 | 61.13 | 301 | HAT4 | Photoperiod | #N/A | #N/A | #N/A |
| AT4G16780.1 | i1_LQ_HNbud_c25109/f1p3/1410 | 61.13 | 301 | HAT4 | Photoperiod | 15.98333333 | 14.27333333 | 8.74 |
| AT5G11260.1 | i0_HQ_HNbud_c24160/f2p5/675 | 77.61 | 134 | HY5 | Photoperiod | 16.96 | 16.66 | 20.94333333 |
| AT5G11260.1 | i0_LQ_HNbud_c22781/f1p0/763 | 51.28 | 117 | HY5 | Photoperiod | 2.43 | 13.07666667 | 10.26 |
| AT5G11260.1 | i0_LQ_HNbud_c51018/f1p0/743 | 51.28 | 117 | HY5 | Photoperiod | 3.733333333 | 12.76666667 | 12.04 |
| AT5G02840.1 | i1_HQ_HNbud_c37087/f3p5/1301 | 67.97 | 256 | LCL1 | Photoperiod | #N/A | #N/A | #N/A |
| AT5G02840.1 | i1_LQ_HNbud_c154581/f1p5/1467 | 67.97 | 256 | LCL1 | Photoperiod | 6.506666667 | 39.79333333 | 57.59 |
| AT5G64813.1 | i1_LQ_HNbud_c180659/f1p3/1827 | 83.33 | 210 | LIP1 | Photoperiod | 1.873333333 | 2.086666667 | 1.863333333 |
| AT5G64813.1 | i1_LQ_HNbud_c111579/f1p3/1787 | 82.89 | 228 | LIP1 | Photoperiod | 0.046666667 | 0 | 0.023333333 |
| AT5G64813.1 | i1_LQ_HNbud_c224355/f1p3/1748 | 76.54 | 341 | LIP1 | Photoperiod | 4.12 | 6.503333333 | 5.453333333 |
| AT5G64813.1 | i1_LQ_HNbud_c90198/f1p3/1705 | 76.54 | 341 | LIP1 | Photoperiod | 0.203333333 | 0.09 | 0.29 |
| AT5G64813.1 | i1_LQ_HNbud_c41284/f1p3/1445 | 76.25 | 341 | LIP1 | Photoperiod | #N/A | #N/A | #N/A |
| AT5G64813.1 | i2_LQ_HNbud_c39168/f1p0/2687 | 75.88 | 311 | LIP1 | Photoperiod | 0.166666667 | 0.16 | 0.173333333 |
| AT5G64813.1 | i1_HQ_HNbud_c100165/f10p3/1560 | 75.66 | 341 | LIP1 | Photoperiod | 4.073333333 | 6.89 | 5.77 |
| AT5G28490.1 | i0_LQ_HNbud_c46858/f1p0/953 | 85.07 | 134 | LSH1 | Photoperiod | 2.393333333 | 4.076666667 | 2.64 |
| AT5G28490.1 | i1_HQ_HNbud_c69314/f2p4/1128 | 79.26 | 135 | LSH1 | Photoperiod | #N/A | #N/A | #N/A |
| AT5G28490.1 | i1_LQ_HNbud_c227013/f1p2/1148 | 79.26 | 135 | LSH1 | Photoperiod | #N/A | #N/A | #N/A |
| AT5G28490.1 | i1_LQ_HNbud_c23148/f1p4/1235 | 79.26 | 135 | LSH1 | Photoperiod | 33.95 | 26.57 | 27.16333333 |
| AT5G28490.1 | i1_LQ_HNbud_c75096/f1p4/1294 | 79.26 | 135 | LSH1 | Photoperiod | 34.58 | 21.00333333 | 22.67 |
| AT5G28490.1 | i1_LQ_HNbud_c99220/f1p4/1121 | 79.26 | 135 | LSH1 | Photoperiod | #N/A | #N/A | #N/A |
| AT5G28490.1 | i1_HQ_HNbud_c17024/f15p0/1205 | 75.32 | 158 | LSH1 | Photoperiod | #N/A | #N/A | #N/A |
| AT5G28490.1 | i1_LQ_HNbud_c133400/f1p0/1265 | 75.32 | 158 | LSH1 | Photoperiod | 20.3 | 23.75333333 | 14.98333333 |
| AT5G28490.1 | i0_LQ_HNbud_c23137/f1p1/851 | 74.68 | 158 | LSH1 | Photoperiod | 22.08 | 13.47 | 8.613333333 |
| AT5G28490.1 | i0_LQ_HNbud_c45481/f1p77/1001 | 72.39 | 134 | LSH1 | Photoperiod | 5.686666667 | 30.3 | 33.35333333 |
| AT1G12910.1 | i1_LQ_HNbud_c50866/f1p7/1823 | 93.22 | 339 | LWD1/ATAN11 | Photoperiod | 1.173333333 | 1.966666667 | 1.763333333 |
| AT1G12910.1 | i1_HQ_HNbud_c81892/f33p7/1291 | 93.06 | 346 | LWD1/ATAN11 | Photoperiod | 9.443333333 | 18.63666667 | 18.07666667 |
| AT1G12910.1 | i1_LQ_HNbud_c152141/f1p7/1689 | 93.06 | 346 | LWD1/ATAN11 | Photoperiod | 0.766666667 | 2.063333333 | 2.64 |
| AT1G12910.1 | i1_LQ_HNbud_c3299/f1p7/1310 | 93.06 | 346 | LWD1/ATAN11 | Photoperiod | 11.56333333 | 21.71666667 | 26.54 |
| AT1G12910.1 | i4_LQ_HNbud_c1934/f1p2/4380 | 93.06 | 346 | LWD1/ATAN11 | Photoperiod | 0.09 | 0.11 | 0.186666667 |
| AT1G12910.1 | i1_HQ_HNbud_c154039/f2p2/1456 | 65.15 | 307 | LWD1/ATAN11 | Photoperiod | 3.293333333 | 9.383333333 | 8.606666667 |
| AT1G12910.1 | i1_HQ_HNbud_c26325/f9p2/1471 | 65.15 | 307 | LWD1/ATAN11 | Photoperiod | 3.92 | 9.756666667 | 9.703333333 |
| AT1G12910.1 | i1_LQ_HNbud_c11462/f1p2/1303 | 65.15 | 307 | LWD1/ATAN11 | Photoperiod | 0 | 0.453333333 | 0 |
| AT1G12910.1 | i1_LQ_HNbud_c48921/f1p2/1423 | 65.15 | 307 | LWD1/ATAN11 | Photoperiod | #N/A | #N/A | #N/A |
| AT1G12910.1 | i1_LQ_HNbud_c89057/f2p2/1332 | 65.15 | 307 | LWD1/ATAN11 | Photoperiod | 2.523333333 | 6.14 | 5.226666667 |
| AT3G01460.1 | i3_LQ_HNbud_c6033/f1p2/3308 | 82.56 | 195 | MBD9 | Photoperiod | 0 | 0 | 0 |
| AT3G01460.1 | i6_HQ_HNbud_c341/f3p0/6932_g0002 | 81.31 | 321 | MBD9 | Photoperiod | #N/A | #N/A | #N/A |
| AT3G01460.1 | i6_HQ_HNbud_c3402/f12p3/6903 | 81.18 | 2179 | MBD9 | Photoperiod | 0 | 0 | 0 |
| AT3G01460.1 | i6_HQ_HNbud_c341/f3p0/6932 | 79.88 | 1372 | MBD9 | Photoperiod | 0 | 0 | 0 |
| AT3G01460.1 | i6_HQ_HNbud_c343/f2p0/6937 | 79.06 | 1729 | MBD9 | Photoperiod | 0 | 0 | 0 |
| AT3G01460.1 | i3_LQ_HNbud_c3650/f1p0/3634 | 53.86 | 466 | MBD9 | Photoperiod | #N/A | #N/A | #N/A |
| AT5G12840.1 | i1_HQ_HNbud_c58372/f4p0/1367 | 51.06 | 282 | NFYA1/HAP2A | Photoperiod | 5.876666667 | 3.536666667 | 2.246666667 |
| AT2G34720.1 | i0_LQ_HNbud_c41157/f2p1/992 | 59.89 | 177 | NFYA4 | Photoperiod | #N/A | #N/A | #N/A |
| AT2G34720.1 | i1_HQ_HNbud_c219485/f5p3/1307 | 59.89 | 177 | NFYA4 | Photoperiod | 10.02 | 8.51 | 8.533333333 |
| AT2G34720.1 | i1_HQ_HNbud_c233759/f6p3/1161 | 59.89 | 177 | NFYA4 | Photoperiod | #N/A | #N/A | #N/A |
| AT2G34720.1 | i1_HQ_HNbud_c242861/f2p3/1024 | 59.89 | 177 | NFYA4 | Photoperiod | #N/A | #N/A | #N/A |
| AT2G34720.1 | i1_LQ_HNbud_c99029/f1p3/1039 | 59.89 | 177 | NFYA4 | Photoperiod | 10.59 | 4.646666667 | 7.24 |
| AT2G34720.1 | i1_LQ_HNbud_c154956/f1p3/1347 | 52.5 | 200 | NFYA4 | Photoperiod | 5.693333333 | 4.473333333 | 5.556666667 |
| AT2G38880.1 | i0_HQ_HNbud_c28099/f2p0/995 | 81.29 | 139 | NFYB1 | Photoperiod | #N/A | #N/A | #N/A |
| AT2G38880.1 | i0_HQ_HNbud_c97840/f7p0/955 | 81.29 | 139 | NFYB1 | Photoperiod | 7.303333333 | 14.78333333 | 14.4 |
| AT2G38880.1 | i0_HQ_HNbud_c98469/f5p0/868 | 81.29 | 139 | NFYB1 | Photoperiod | #N/A | #N/A | #N/A |
| AT2G38880.1 | i0_LQ_HNbud_c110112/f1p0/845 | 81.29 | 139 | NFYB1 | Photoperiod | 0 | 0 | 0.11 |
| AT2G38880.1 | i0_LQ_HNbud_c73497/f1p4/989 | 81.29 | 139 | NFYB1 | Photoperiod | #N/A | #N/A | #N/A |
| AT2G38880.1 | i1_HQ_HNbud_c115674/f2p2/1040 | 81.29 | 139 | NFYB1 | Photoperiod | 9.28 | 14.76 | 12.70333333 |
| AT2G38880.1 | i1_LQ_HNbud_c214047/f1p2/1042 | 81.29 | 139 | NFYB1 | Photoperiod | 9.09 | 9.873333333 | 16.82333333 |
| AT2G38880.1 | i1_LQ_HNbud_c214929/f1p2/1022 | 81.29 | 139 | NFYB1 | Photoperiod | 2.96 | 5.88 | 7.42 |
| AT2G38880.1 | i1_LQ_HNbud_c236082/f1p2/1034 | 81.29 | 139 | NFYB1 | Photoperiod | 0 | 0 | 0 |
| AT2G38880.1 | i1_LQ_HNbud_c279476/f1p2/1008 | 81.29 | 139 | NFYB1 | Photoperiod | #N/A | #N/A | #N/A |
| AT5G47640.1 | i0_LQ_HNbud_c69184/f1p3/952 | 93.1 | 116 | NFYB2 | Photoperiod | 0 | 0 | 0 |
| AT5G47640.1 | i0_LQ_HNbud_c68118/f1p2/996 | 80.16 | 126 | NFYB2 | Photoperiod | 28.13666667 | 18.81 | 18.12666667 |
| AT5G47640.1 | i0_HQ_HNbud_c1972/f2p0/785 | 70.59 | 136 | NFYB2 | Photoperiod | #N/A | #N/A | #N/A |
| AT5G47640.1 | i0_HQ_HNbud_c29028/f3p0/844 | 70.59 | 136 | NFYB2 | Photoperiod | 3.193333333 | 18.19 | 13.23333333 |
| AT4G14540.1 | i0_HQ_HNbud_c2433/f2p0/920 | 81.43 | 140 | NFYB3/HAP3C | Photoperiod | 31.26333333 | 36.82333333 | 31.55666667 |
| AT4G14540.1 | i0_LQ_HNbud_c4201/f1p14/908 | 80.71 | 140 | NFYB3/HAP3C | Photoperiod | 0 | 0.266666667 | 0 |
| AT2G37060.1 | i0_HQ_HNbud_c1698/f5p130/862 | 76 | 175 | NF-YB8 | Photoperiod | 100.2666667 | 14.59666667 | 7 |
| AT2G37060.1 | i1_LQ_HNbud_c155034/f1p0/1405 | 73.84 | 172 | NF-YB8 | Photoperiod | 8.54 | 2.186666667 | 1.52 |
| AT3G48590.1 | i1_HQ_HNbud_c168660/f3p1/1102 | 88.61 | 202 | NF-YC1 | Photoperiod | 34.62666667 | 11.07333333 | 8.32 |
| AT1G56170.1 | i0_LQ_HNbud_c5599/f1p0/994 | 72.13 | 183 | NFYC2/HAP5B | Photoperiod | #N/A | #N/A | #N/A |
| AT1G56170.1 | i1_HQ_HNbud_c263793/f7p3/1159 | 63.51 | 222 | NFYC2/HAP5B | Photoperiod | 30.59333333 | 28.09333333 | 24.33666667 |
| AT1G56170.1 | i1_LQ_HNbud_c187940/f1p3/1043 | 63.06 | 222 | NFYC2/HAP5B | Photoperiod | #N/A | #N/A | #N/A |
| AT1G56170.1 | i1_LQ_HNbud_c205960/f1p3/1189 | 63.06 | 222 | NFYC2/HAP5B | Photoperiod | 5.373333333 | 3.543333333 | 3.75 |
| AT1G56170.1 | i0_HQ_HNbud_c9969/f7p1/998 | 61.75 | 217 | NFYC2/HAP5B | Photoperiod | #N/A | #N/A | #N/A |
| AT1G56170.1 | i1_HQ_HNbud_c235143/f2p3/1028 | 61.75 | 217 | NFYC2/HAP5B | Photoperiod | 37.23666667 | 31.76 | 29.81666667 |
| AT1G56170.1 | i1_LQ_HNbud_c277332/f1p3/1002 | 61.75 | 217 | NFYC2/HAP5B | Photoperiod | #N/A | #N/A | #N/A |
| AT1G56170.1 | i1_LQ_HNbud_c279763/f1p3/1005 | 61.75 | 217 | NFYC2/HAP5B | Photoperiod | #N/A | #N/A | #N/A |
| AT1G56170.1 | i1_LQ_HNbud_c39430/f1p3/1240 | 61.75 | 217 | NFYC2/HAP5B | Photoperiod | 0 | 0.083333333 | 0.076666667 |
| AT1G54830.1 | i0_LQ_HNbud_c44511/f1p0/813 | 69.71 | 175 | NFYC9(NF-YC3) | Photoperiod | #N/A | #N/A | #N/A |
| AT1G54830.1 | i1_LQ_HNbud_c116932/f1p0/1050 | 60.43 | 235 | NFYC9(NF-YC3) | Photoperiod | #N/A | #N/A | #N/A |
| AT1G54830.1 | i1_LQ_HNbud_c85579/f1p0/1100 | 60.43 | 235 | NFYC9(NF-YC3) | Photoperiod | 3.35 | 4.986666667 | 6.19 |
| AT5G20730.1 | i4_LQ_HNbud_c3254/f1p0/4696 | 80.12 | 498 | NPH4 | Photoperiod | 6.016666667 | 11.16666667 | 13.91333333 |
| AT5G20730.1 | i4_LQ_HNbud_c3925/f1p0/4891 | 79.92 | 498 | NPH4 | Photoperiod | 5.97 | 8.303333333 | 7.193333333 |
| AT5G20730.1 | i4_LQ_HNbud_c1960/f1p2/4403 | 78.31 | 498 | NPH4 | Photoperiod | 2.54 | 3.6 | 3.523333333 |
| AT5G20730.1 | i4_LQ_HNbud_c2972/f1p4/4785 | 75.3 | 498 | NPH4 | Photoperiod | #N/A | #N/A | #N/A |
| AT5G20730.1 | i3_LQ_HNbud_c10960/f1p0/3818 | 65.17 | 557 | NPH4 | Photoperiod | #N/A | #N/A | #N/A |
| AT5G20730.1 | i4_HQ_HNbud_c165/f3p0/4133 | 65.17 | 557 | NPH4 | Photoperiod | 12.96333333 | 16.81666667 | 14.72333333 |
| AT5G20730.1 | i4_HQ_HNbud_c4376/f5p6/4198 | 65.15 | 551 | NPH4 | Photoperiod | 14.36333333 | 19.37333333 | 18.46333333 |
| AT5G20730.1 | i4_LQ_HNbud_c1443/f1p0/4082 | 64.97 | 551 | NPH4 | Photoperiod | #N/A | #N/A | #N/A |
| AT5G20730.1 | i3_LQ_HNbud_c9994/f1p9/3807 | 62.61 | 551 | NPH4 | Photoperiod | #N/A | #N/A | #N/A |
| AT5G20730.1 | i7_LQ_HNbud_c853/f1p0/7231 | 54.69 | 565 | NPH4 | Photoperiod | 0.01 | 0.01 | 0.016666667 |
| Os09t0346500_04 | i1_HQ_HNbud_c254844/f8p31/1016 | 87.55 | 265 | OsCAB1R | Photoperiod | 102.4733333 | 514.9066667 | 297.9133333 |
| Os09t0346500_04 | i1_HQ_HNbud_c246035/f10p31/1206 | 88.3 | 265 | OsCAB1R | Photoperiod | 46.16333333 | 90.79333333 | 46.55 |
| Os09t0346500_04 | i2_LQ_HNbud_c13995/f1p27/2338 | 86.31 | 263 | OsCAB1R | Photoperiod | 0.28 | 0.103333333 | 0.216666667 |
| Os09t0346500_04 | i1_LQ_HNbud_c241343/f1p32/1078 | 86.04 | 265 | OsCAB1R | Photoperiod | 0.436666667 | 1.396666667 | 1.063333333 |
| Os09t0346500_04 | i1_LQ_HNbud_c279044/f1p27/1010 | 86.04 | 265 | OsCAB1R | Photoperiod | 0 | 0 | 0 |
| Os09t0346500_04 | i1_LQ_HNbud_c279068/f1p31/1006 | 86.04 | 265 | OsCAB1R | Photoperiod | 0.653333333 | 3.51 | 1.936666667 |
| Os09t0346500_04 | i1_LQ_HNbud_c14902/f1p29/1027 | 85.77 | 260 | OsCAB1R | Photoperiod | #N/A | #N/A | #N/A |
| Os09t0346500_04 | i1_LQ_HNbud_c214747/f1p17/971 | 85.77 | 260 | OsCAB1R | Photoperiod | 13.02666667 | 2.686666667 | 0.93 |
| Os09t0346500_04 | i1_LQ_HNbud_c277450/f1p31/1013 | 85.77 | 260 | OsCAB1R | Photoperiod | #N/A | #N/A | #N/A |
| Os09t0346500_04 | i1_LQ_HNbud_c116175/f1p24/988 | 84.91 | 265 | OsCAB1R | Photoperiod | #N/A | #N/A | #N/A |
| Os01t0566100_01 | i2_LQ_HNbud_c1798/f3p1/2701 | 61.54 | 39 | OsELF3 | Photoperiod | 2.43 | 2.386666667 | 2.176666667 |
| Os01t0566100_01 | i2_LQ_HNbud_c8767/f1p1/3040 | 61.54 | 39 | OsELF3 | Photoperiod | 6.493333333 | 4.073333333 | 3.743333333 |
| Os01t0566100_01 | i3_LQ_HNbud_c16293/f1p0/3046 | 61.54 | 39 | OsELF3 | Photoperiod | 1.55 | 0.92 | 0.556666667 |
| Os01t0566100_01 | i3_LQ_HNbud_c17403/f1p3/3030 | 61.54 | 39 | OsELF3 | Photoperiod | 6.823333333 | 6.426666667 | 6.153333333 |
| Os01t0182600_01 | i2_LQ_HNbud_c38991/f1p3/2615 | 67.04 | 810 | OsGI | Photoperiod | #N/A | #N/A | #N/A |
| Os09t0306700_01 | i1_LQ_HNbud_c51084/f1p0/1577 | 70.24 | 84 | OsPFT1 | Photoperiod | 0 | 0 | 0 |
| Os09t0306700_01 | i2_LQ_HNbud_c57722/f1p0/2862 | 55.56 | 36 | OsPFT1 | Photoperiod | 0 | 0 | 0 |
| AT5G48150.1 | i1_LQ_HNbud_c154352/f1p0/1839 | 87.4 | 500 | PAT1 | Photoperiod | 0 | 0.01 | 0 |
| AT5G48150.1 | i1_LQ_HNbud_c230373/f1p0/1762 | 87.2 | 500 | PAT1 | Photoperiod | #N/A | #N/A | #N/A |
| AT5G48150.1 | i2_HQ_HNbud_c31157/f4p0/2130 | 73.97 | 461 | PAT1 | Photoperiod | 2.693333333 | 2.99 | 2.55 |
| AT5G48150.1 | i2_LQ_HNbud_c23496/f1p1/2677 | 73.75 | 461 | PAT1 | Photoperiod | 3.79 | 5.073333333 | 4.03 |
| AT5G48150.1 | i2_LQ_HNbud_c8536/f1p2/2106 | 73.75 | 461 | PAT1 | Photoperiod | #N/A | #N/A | #N/A |
| AT5G48150.1 | i1_LQ_HNbud_c226221/f1p3/2012 | 70.71 | 478 | PAT1 | Photoperiod | #N/A | #N/A | #N/A |
| AT5G48150.1 | i2_LQ_HNbud_c11038/f1p0/2142 | 70.71 | 478 | PAT1 | Photoperiod | #N/A | #N/A | #N/A |
| AT5G48150.1 | i2_LQ_HNbud_c18756/f1p0/2261 | 70.71 | 478 | PAT1 | Photoperiod | 0.933333333 | 0.726666667 | 1.59 |
| AT5G48150.1 | i2_LQ_HNbud_c25504/f1p0/2163 | 70.71 | 478 | PAT1 | Photoperiod | 6.256666667 | 8.963333333 | 7.413333333 |
| AT5G48150.1 | i1_LQ_HNbud_c5119/f1p2/1971 | 70.71 | 478 | PAT1 | Photoperiod | 3.313333333 | 0.41 | 4.633333333 |
| AT2G18790.1 | i3_LQ_HNbud_c10533/f1p7/3415 | 79.73 | 1036 | PHYB | Photoperiod | #N/A | #N/A | #N/A |
| AT2G18790.1 | i3_HQ_HNbud_c16818/f7p0/3906 | 79.39 | 1121 | PHYB | Photoperiod | #N/A | #N/A | #N/A |
| AT2G18790.1 | i3_LQ_HNbud_c17266/f1p1/4049 | 79.39 | 1121 | PHYB | Photoperiod | 3.35 | 5.136666667 | 5.6 |
| AT2G18790.1 | i3_LQ_HNbud_c17407/f1p0/3736 | 79.36 | 1119 | PHYB | Photoperiod | #N/A | #N/A | #N/A |
| AT2G18790.1 | i4_LQ_HNbud_c4111/f1p15/4026 | 79.36 | 1119 | PHYB | Photoperiod | 5.563333333 | 9.24 | 9.69 |
| AT2G18790.1 | i3_LQ_HNbud_c2316/f1p43/3837 | 79.3 | 1111 | PHYB | Photoperiod | #N/A | #N/A | #N/A |
| AT5G35840.1 | i4_LQ_HNbud_c1485/f1p1/4537 | 65.05 | 1113 | PHYC | Photoperiod | 1.086666667 | 0.12 | 0.173333333 |
| AT4G18130.1 | i3_LQ_HNbud_c3306/f1p1/3685 | 61.31 | 1026 | PHYE | Photoperiod | 0.053333333 | 0.263333333 | 0.31 |
| AT4G18130.1 | i3_HQ_HNbud_c16783/f7p0/3687 | 61.11 | 1098 | PHYE | Photoperiod | 5.963333333 | 4.013333333 | 4.02 |
| AT4G18130.1 | i3_LQ_HNbud_c7664/f1p0/3975 | 58.93 | 1098 | PHYE | Photoperiod | 2.71 | 2.68 | 2.483333333 |
| AT4G18130.1 | i3_LQ_HNbud_c11424/f1p0/3509 | 58.65 | 1098 | PHYE | Photoperiod | 0 | 0 | 0 |
| AT1G09530.1 | i1_LQ_HNbud_c92141/f1p0/1903 | 77.02 | 531 | PIF3 | Photoperiod | 0 | 0 | 0 |
| AT1G09530.1 | i1_LQ_HNbud_c223858/f1p3/1944 | 66.35 | 104 | PIF3 | Photoperiod | 1.76 | 1.46 | 1.773333333 |
| AT1G09530.1 | i1_LQ_HNbud_c227634/f1p1/1470 | 59.4 | 133 | PIF3 | Photoperiod | 0.956666667 | 3.07 | 3.65 |
| AT1G09530.1 | i2_LQ_HNbud_c16824/f2p1/2059 | 59.4 | 133 | PIF3 | Photoperiod | 0 | 0 | 0 |
| AT1G09530.1 | i2_LQ_HNbud_c22818/f1p1/2692 | 59.4 | 133 | PIF3 | Photoperiod | 0.026666667 | 0.093333333 | 0.246666667 |
| AT1G09530.1 | i2_LQ_HNbud_c24589/f1p1/2221 | 59.4 | 133 | PIF3 | Photoperiod | 2.14 | 1.403333333 | 1.593333333 |
| AT1G09530.1 | i2_LQ_HNbud_c9888/f1p4/2224 | 59.4 | 133 | PIF3 | Photoperiod | 1.656666667 | 3.363333333 | 4.096666667 |
| AT2G43010.1 | i1_LQ_HNbud_c156840/f1p0/1682 | 71.35 | 192 | PIF4 | Photoperiod | 0 | 0 | 0 |
| AT2G43010.1 | i1_LQ_HNbud_c71140/f1p0/1439 | 69.46 | 429 | PIF4 | Photoperiod | 0 | 0 | 0 |
| AT5G60100.1 | i2_HQ_HNbud_c70577/f11p2/2944 | 88.89 | 27 | PRR3 | Photoperiod | #N/A | #N/A | #N/A |
| AT5G60100.1 | i2_HQ_HNbud_c71047/f4p3/2934 | 88.89 | 27 | PRR3 | Photoperiod | #N/A | #N/A | #N/A |
| AT5G60100.1 | i2_HQ_HNbud_c70504/f3p0/2809 | 81.25 | 48 | PRR3 | Photoperiod | #N/A | #N/A | #N/A |
| AT5G60100.1 | i2_LQ_HNbud_c71503/f1p0/2817 | 81.25 | 48 | PRR3 | Photoperiod | #N/A | #N/A | #N/A |
| AT5G60100.1 | i2_LQ_HNbud_c71590/f2p3/2894 | 81.25 | 48 | PRR3 | Photoperiod | 0.016666667 | 0 | 0.013333333 |
| AT5G60100.1 | i2_LQ_HNbud_c71837/f1p0/2825 | 81.25 | 48 | PRR3 | Photoperiod | #N/A | #N/A | #N/A |
| AT5G60100.1 | i2_LQ_HNbud_c71849/f1p1/2884 | 81.25 | 48 | PRR3 | Photoperiod | 9.723333333 | 13.79333333 | 25.74666667 |
| AT5G60100.1 | i3_LQ_HNbud_c4136/f1p0/3143 | 81.25 | 48 | PRR3 | Photoperiod | 3.393333333 | 1.713333333 | 1.803333333 |
| AT5G60100.1 | i2_HQ_HNbud_c70530/f3p1/2949 | 81.25 | 48 | PRR3 | Photoperiod | 6.676666667 | 17.58333333 | 28.11 |
| AT5G02810.1 | i2_HQ_HNbud_c17897/f3p1/2563 | 80.87 | 737 | PRR7 | Photoperiod | 0 | 0 | 0 |
| AT5G02810.1 | i2_HQ_HNbud_c42645/f2p1/2529 | 78.73 | 503 | PRR7 | Photoperiod | 0 | 0 | 0 |
| AT5G02810.1 | i2_LQ_HNbud_c50893/f1p3/2869 | 77.37 | 137 | PRR7 | Photoperiod | 0 | 0 | 0 |
| AT2G46790.1 | i2_LQ_HNbud_c43637/f1p3/2574 | 83.33 | 48 | PRR9 | Photoperiod | 0.976666667 | 0.78 | 0.663333333 |
| AT2G46790.1 | i1_LQ_HNbud_c178911/f1p0/1988 | 82.98 | 47 | PRR9 | Photoperiod | #N/A | #N/A | #N/A |
| AT2G46790.1 | i2_HQ_HNbud_c28961/f2p0/2092 | 82.98 | 47 | PRR9 | Photoperiod | 0 | 0 | 0 |
| AT2G46790.1 | i1_LQ_HNbud_c22225/f1p3/2004 | 82.35 | 51 | PRR9 | Photoperiod | 0 | 0.06 | 0 |
| AT2G46790.1 | i2_LQ_HNbud_c25618/f1p5/2189 | 71.83 | 142 | PRR9 | Photoperiod | #N/A | #N/A | #N/A |
| AT2G46790.1 | i2_HQ_HNbud_c36500/f2p5/2385 | 68.13 | 160 | PRR9 | Photoperiod | 10.49666667 | 10.24666667 | 13.08333333 |
| AT2G46790.1 | i2_HQ_HNbud_c48155/f3p2/2087 | 68.13 | 160 | PRR9 | Photoperiod | #N/A | #N/A | #N/A |
| AT2G46790.1 | i2_LQ_HNbud_c13317/f1p2/2147 | 68.13 | 160 | PRR9 | Photoperiod | 0.256666667 | 0 | 0 |
| AT2G46790.1 | i1_HQ_HNbud_c48960/f2p0/1796 | 63.8 | 453 | PRR9 | Photoperiod | 0 | 0 | 0 |
| Os04t0690800_01 | i1_HQ_HNbud_c264088/f7p5/1114 | 71.9 | 274 | psbS2 | Photoperiod | 3.32 | 31.85666667 | 21.69 |
| Os04t0690800_01 | i1_LQ_HNbud_c214620/f1p5/1011 | 71.9 | 274 | psbS2 | Photoperiod | 0.886666667 | 11.46333333 | 6.09 |
| Os04t0690800_01 | i0_HQ_HNbud_c936/f19p0/984 | 71.9 | 274 | psbS2 | Photoperiod | #N/A | #N/A | #N/A |
| Os04t0690800_01 | i0_LQ_HNbud_c83790/f1p2/987 | 71.9 | 274 | psbS2 | Photoperiod | #N/A | #N/A | #N/A |
| Os04t0690800_01 | i1_HQ_HNbud_c256076/f4p5/1052 | 71.9 | 274 | psbS2 | Photoperiod | 0 | 0 | 0.63 |
| Os04t0690800_01 | i1_LQ_HNbud_c160641/f1p5/1026 | 71.9 | 274 | psbS2 | Photoperiod | #N/A | #N/A | #N/A |
| Os04t0690800_01 | i1_LQ_HNbud_c278448/f1p5/1005 | 71.9 | 274 | psbS2 | Photoperiod | #N/A | #N/A | #N/A |
| Os04t0690800_01 | i1_LQ_HNbud_c161756/f1p4/961 | 71.17 | 274 | psbS2 | Photoperiod | #N/A | #N/A | #N/A |
| Os04t0690800_01 | i1_LQ_HNbud_c163895/f1p4/1042 | 71.17 | 274 | psbS2 | Photoperiod | 0 | 4.74 | 0.806666667 |
| Os04t0690800_01 | i1_LQ_HNbud_c187370/f1p4/985 | 71.17 | 274 | psbS2 | Photoperiod | 0 | 1.1 | 0 |
| AT1G13260.1 | i1_HQ_HNbud_c106992/f2p0/1274 | 88.06 | 310 | RAV1 | Photoperiod | 0 | 0 | 0 |
| AT1G13260.1 | i1_LQ_HNbud_c79135/f1p3/1043 | 67.11 | 304 | RAV1 | Photoperiod | #N/A | #N/A | #N/A |
| AT1G13260.1 | i1_LQ_HNbud_c249520/f1p3/1444 | 64.29 | 350 | RAV1 | Photoperiod | 6.106666667 | 2.21 | 4.913333333 |
| AT1G13260.1 | i1_LQ_HNbud_c34849/f6p3/1343 | 64.29 | 350 | RAV1 | Photoperiod | #N/A | #N/A | #N/A |
| AT1G13260.1 | i1_LQ_HNbud_c127738/f2p3/1201 | 64.08 | 348 | RAV1 | Photoperiod | #N/A | #N/A | #N/A |
| AT1G13260.1 | i1_LQ_HNbud_c54685/f1p3/1159 | 63.74 | 353 | RAV1 | Photoperiod | #N/A | #N/A | #N/A |
| AT1G13260.1 | i1_LQ_HNbud_c92608/f1p2/1535 | 63.47 | 323 | RAV1 | Photoperiod | 0 | 0 | 0 |
| AT1G62750.1 | i2_HQ_HNbud_c31604/f13p1/2620 | 95.28 | 784 | SCO1 | Photoperiod | #N/A | #N/A | #N/A |
| AT1G62750.1 | i2_HQ_HNbud_c5780/f4p1/2690 | 95.28 | 784 | SCO1 | Photoperiod | 0 | 0 | 0 |
| AT1G62750.1 | i2_LQ_HNbud_c24742/f1p1/2698 | 94.26 | 784 | SCO1 | Photoperiod | 0 | 0 | 0 |
| AT1G62750.1 | i2_LQ_HNbud_c63669/f1p1/2361 | 90.34 | 704 | SCO1 | Photoperiod | #N/A | #N/A | #N/A |
| AT1G62750.1 | i6_LQ_HNbud_c601/f1p0/6572 | 89.45 | 597 | SCO1 | Photoperiod | 0.07 | 0.176666667 | 0.12 |
| AT1G62750.1 | i2_HQ_HNbud_c18052/f5p1/2554 | 85.48 | 778 | SCO1 | Photoperiod | 3.813333333 | 3.65 | 6.24 |
| AT1G62750.1 | i2_LQ_HNbud_c35526/f1p1/2526 | 84.96 | 778 | SCO1 | Photoperiod | #N/A | #N/A | #N/A |
| AT1G62750.1 | i2_LQ_HNbud_c57923/f1p16/2614 | 84.83 | 778 | SCO1 | Photoperiod | 4.546666667 | 6.39 | 8.863333333 |
| AT1G62750.1 | i5_LQ_HNbud_c1180/f1p5/5206 | 84.7 | 778 | SCO1 | Photoperiod | 0.063333333 | 0.136666667 | 0.103333333 |
| AT1G62750.1 | i2_LQ_HNbud_c20194/f1p1/2658 | 85.48 | 778 | SCO1 | Photoperiod | 0.44 | 2.963333333 | 2.716666667 |
| Os01t0949400_01 | i1_LQ_HNbud_c36068/f1p1/1280 | 66.4 | 247 | Se-13 | Photoperiod | 0.223333333 | 0.396666667 | 0.656666667 |
| Os01t0949400_01 | i1_LQ_HNbud_c67408/f1p2/1247 | 66.4 | 247 | Se-13 | Photoperiod | 0 | 0 | 0 |
| Os01t0949400_01 | i1_HQ_HNbud_c2170/f8p2/1214 | 64.64 | 280 | Se-13 | Photoperiod | 5.62 | 7.793333333 | 9.913333333 |
| AT1G69935.1 | i0_LQ_HNbud_c55327/f1p4/696 | 69.41 | 85 | SHW1 | Photoperiod | 10.24 | 12.02333333 | 11.13 |
| AT2G39250.1 | i1_LQ_HNbud_c107790/f2p2/1973 | 56.82 | 176 | SNZ | Photoperiod | 1.956666667 | 3.056666667 | 3.17 |
| AT2G39250.1 | i2_LQ_HNbud_c27695/f1p1/2235 | 56.82 | 176 | SNZ | Photoperiod | 0.033333333 | 0.513333333 | 0.313333333 |
| AT2G39250.1 | i2_LQ_HNbud_c73537/f1p2/2005 | 50.84 | 179 | SNZ | Photoperiod | 1.75 | 9.91 | 8.093333333 |
| AT2G39250.1 | i1_LQ_HNbud_c110748/f1p3/1916 | 53.93 | 191 | SNZ | Photoperiod | 0.123333333 | 0.38 | 0.116666667 |
| AT2G39250.1 | i1_LQ_HNbud_c39667/f1p3/1879 | 53.93 | 191 | SNZ | Photoperiod | 4.363333333 | 10.99333333 | 8.22 |
| AT2G39250.1 | i2_LQ_HNbud_c15527/f1p2/1996 | 53.93 | 191 | SNZ | Photoperiod | 0.063333333 | 0 | 0 |
| AT2G39250.1 | i2_LQ_HNbud_c19459/f1p3/2149 | 53.93 | 191 | SNZ | Photoperiod | 0 | 0.133333333 | 0.93 |
| AT2G39250.1 | i2_LQ_HNbud_c73717/f1p2/2003 | 53.93 | 191 | SNZ | Photoperiod | 0.133333333 | 0.073333333 | 0 |
| AT2G39250.1 | i2_LQ_HNbud_c9157/f1p19/2375 | 53.93 | 191 | SNZ | Photoperiod | 0.173333333 | 0.326666667 | 0.08 |
| AT2G39250.1 | i1_LQ_HNbud_c110103/f1p3/2000 | 50.84 | 179 | SNZ | Photoperiod | #N/A | #N/A | #N/A |
| AT2G46340.1 | i3_LQ_HNbud_c8223/f1p3/3488 | 54.81 | 810 | SPA | Photoperiod | 0 | 0.47 | 0.076666667 |
| AT5G59560.1 | i1_LQ_HNbud_c66671/f1p0/1290 | 57.35 | 272 | SRR1 | Photoperiod | 0.99 | 1.356666667 | 1.303333333 |
| AT5G59560.1 | i3_LQ_HNbud_c15294/f1p0/3032 | 57.35 | 272 | SRR1 | Photoperiod | 0.97 | 1.226666667 | 1 |
| AT1G06040.1 | i1_LQ_HNbud_c45536/f1p0/1081 | 78.31 | 249 | STO | Photoperiod | 0 | 0 | 0 |
| AT1G06040.1 | i0_LQ_HNbud_c77588/f1p0/994 | 62.1 | 248 | STO | Photoperiod | 18.02333333 | 31.52333333 | 29.28666667 |
| AT1G06040.1 | i1_LQ_HNbud_c180619/f1p4/1265 | 62.1 | 248 | STO | Photoperiod | 7.243333333 | 13.31 | 14.05666667 |
| AT1G06040.1 | i1_HQ_HNbud_c121097/f17p4/1318 | 61.69 | 248 | STO | Photoperiod | 31.01 | 54.16666667 | 50.59333333 |
| AT1G06040.1 | i1_HQ_HNbud_c192203/f10p4/1142 | 61.69 | 248 | STO | Photoperiod | #N/A | #N/A | #N/A |
| AT1G06040.1 | i1_HQ_HNbud_c265499/f5p4/1121 | 61.69 | 248 | STO | Photoperiod | #N/A | #N/A | #N/A |
| AT1G06040.1 | i1_LQ_HNbud_c113004/f1p4/1235 | 61.69 | 248 | STO | Photoperiod | #N/A | #N/A | #N/A |
| AT1G06040.1 | i1_LQ_HNbud_c269094/f1p4/1313 | 61.69 | 248 | STO | Photoperiod | 5.95 | 4.713333333 | 5.563333333 |
| AT1G06040.1 | i1_LQ_HNbud_c89734/f1p4/1256 | 61.69 | 248 | STO | Photoperiod | 17.35666667 | 28.57 | 27.24333333 |
| AT1G06040.1 | i1_HQ_HNbud_c66853/f2p4/1405 | 61.69 | 248 | STO | Photoperiod | 0.9 | 8.13 | 14.10333333 |
| AT5G08330.1 | i1_LQ_HNbud_c55633/f1p0/1421 | 78.48 | 79 | TCP11 | Photoperiod | 1.41 | 3.76 | 3.22 |
| AT5G08330.1 | i1_HQ_HNbud_c154815/f2p1/1222 | 64.95 | 97 | TCP11 | Photoperiod | #N/A | #N/A | #N/A |
| AT5G08330.1 | i1_HQ_HNbud_c190814/f4p1/1581 | 64.95 | 97 | TCP11 | Photoperiod | #N/A | #N/A | #N/A |
| AT5G08330.1 | i1_HQ_HNbud_c1994/f4p1/1469 | 64.95 | 97 | TCP11 | Photoperiod | #N/A | #N/A | #N/A |
| AT5G08330.1 | i1_LQ_HNbud_c152984/f1p2/1677 | 64.95 | 97 | TCP11 | Photoperiod | 5.36 | 11.74666667 | 11.11333333 |
| AT5G08330.1 | i1_LQ_HNbud_c34066/f2p7/1429 | 55.84 | 274 | TCP11 | Photoperiod | 16.34333333 | 33.63666667 | 23.18333333 |
| AT5G08330.1 | i1_LQ_HNbud_c132950/f1p7/1606 | 54.74 | 274 | TCP11 | Photoperiod | #N/A | #N/A | #N/A |
| AT5G08330.1 | i1_LQ_HNbud_c1399/f16p7/1799 | 54.74 | 274 | TCP11 | Photoperiod | #N/A | #N/A | #N/A |
| AT5G08330.1 | i1_LQ_HNbud_c179609/f1p7/1755 | 54.74 | 274 | TCP11 | Photoperiod | 0.766666667 | 3.643333333 | 2.04 |
| AT5G08330.1 | i1_LQ_HNbud_c74898/f1p7/1807 | 54.74 | 274 | TCP11 | Photoperiod | 19.08 | 37.98 | 27.59333333 |
| AT3G22380.1 | i3_LQ_HNbud_c18622/f1p0/3044 | 80.32 | 188 | TIC | Photoperiod | #N/A | #N/A | #N/A |
| AT3G22380.1 | i2_LQ_HNbud_c34803/f1p2/2735 | 80.08 | 768 | TIC | Photoperiod | 0 | 0 | 0 |
| AT3G22380.1 | i3_LQ_HNbud_c5773/f1p3/3213 | 75.61 | 742 | TIC | Photoperiod | 0 | 0 | 0 |
| AT3G22380.1 | i4_LQ_HNbud_c3518/f1p8/4937 | 70.96 | 892 | TIC | Photoperiod | 0 | 0 | 0 |
| AT2G28550.1 | i1_LQ_HNbud_c25083/f1p1/1633 | 81.18 | 457 | TOE1 | Photoperiod | 0 | 0 | 0 |
| AT2G28550.1 | i2_LQ_HNbud_c25949/f1p1/2171 | 53.54 | 452 | TOE1 | Photoperiod | 3.8 | 3.16 | 4.456666667 |
| AT2G28550.1 | i2_LQ_HNbud_c27960/f1p0/2153 | 52.68 | 467 | TOE1 | Photoperiod | 7.45 | 5.726666667 | 5.29 |
| AT2G28550.1 | i2_HQ_HNbud_c64847/f6p0/2096 | 52.56 | 468 | TOE1 | Photoperiod | 2.94 | 7.766666667 | 10.21 |
| AT4G00690.1 | i2_HQ_HNbud_c12689/f3p1/2292 | 62.18 | 238 | ULP1B | Photoperiod | 4.233333333 | 5.606666667 | 4.506666667 |
| AT4G00690.1 | i2_HQ_HNbud_c48521/f2p2/2056 | 62.18 | 238 | ULP1B | Photoperiod | #N/A | #N/A | #N/A |
| AT4G00690.1 | i2_HQ_HNbud_c70370/f2p0/2284 | 62.18 | 238 | ULP1B | Photoperiod | 4.16 | 5.216666667 | 4.936666667 |
| AT4G00690.1 | i2_LQ_HNbud_c72498/f1p0/2100 | 62.18 | 238 | ULP1B | Photoperiod | #N/A | #N/A | #N/A |
| AT4G00690.1 | i2_LQ_HNbud_c22035/f1p21/2165 | 58.33 | 180 | ULP1B | Photoperiod | 0 | 0.033333333 | 0.02 |
| AT4G00690.1 | i2_LQ_HNbud_c51759/f1p1/2072 | 58.33 | 180 | ULP1B | Photoperiod | 0.11 | 0.126666667 | 0.096666667 |
| AT4G00690.1 | i2_LQ_HNbud_c7859/f1p0/2498 | 57.5 | 160 | ULP1B | Photoperiod | 0 | 0 | 0.02 |
| AT4G30200.2 | i2_LQ_HNbud_c9052/f1p0/2329 | 66.97 | 221 | VEL1 | Photoperiod | 0 | 0 | 0 |
| AT4G30200.2 | i2_HQ_HNbud_c1240/f7p0/2506 | 50.49 | 103 | VEL1 | Photoperiod | 2.753333333 | 6.536666667 | 7.086666667 |
| AT5G41360.1 | i1_LQ_HNbud_c155555/f1p4/1820 | 88.13 | 514 | XPB2 | Photoperiod | #N/A | #N/A | #N/A |
| AT5G41360.1 | i2_LQ_HNbud_c41403/f1p2/2435 | 87.59 | 669 | XPB2 | Photoperiod | 0.656666667 | 0.756666667 | 1.046666667 |
| AT5G41360.1 | i3_LQ_HNbud_c15008/f1p1/3761 | 86.15 | 325 | XPB2 | Photoperiod | 0.736666667 | 0.86 | 1.076666667 |
| AT5G41360.1 | i2_LQ_HNbud_c36004/f1p1/2679 | 84.65 | 645 | XPB2 | Photoperiod | 7.946666667 | 10.90666667 | 12.87666667 |
| AT5G41360.1 | i2_LQ_HNbud_c58648/f1p1/2602 | 83.7 | 767 | XPB2 | Photoperiod | 0 | 0 | 0 |
| AT5G41360.1 | i2_HQ_HNbud_c989/f14p1/2683 | 83.68 | 772 | XPB2 | Photoperiod | #N/A | #N/A | #N/A |
| AT5G41360.1 | i2_LQ_HNbud_c26443/f1p1/2635 | 83.68 | 772 | XPB2 | Photoperiod | 10.4 | 10.37666667 | 13.46333333 |
| AT5G41360.1 | i2_LQ_HNbud_c3600/f1p4/2518 | 83.46 | 768 | XPB2 | Photoperiod | 0.65 | 1.976666667 | 1.413333333 |
| AT5G41360.1 | i2_LQ_HNbud_c53116/f1p1/2636 | 81.49 | 767 | XPB2 | Photoperiod | #N/A | #N/A | #N/A |
| AT5G41360.1 | i2_HQ_HNbud_c43131/f2p0/2650 | 79.16 | 619 | XPB2 | Photoperiod | 0 | 0 | 0 |
| AT5G57360.2 | i2_LQ_HNbud_c1942/f1p2/2423 | 79.8 | 604 | ZTL | Photoperiod | 6.113333333 | 2.2 | 3.64 |
| AT5G57360.2 | i2_LQ_HNbud_c22255/f1p3/2671 | 79.8 | 604 | ZTL | Photoperiod | 8.16 | 5.39 | 5.023333333 |
| AT5G57360.2 | i2_LQ_HNbud_c42501/f1p3/2459 | 79.8 | 604 | ZTL | Photoperiod | #N/A | #N/A | #N/A |
| AT5G57360.2 | i2_LQ_HNbud_c50791/f1p0/2521 | 79.8 | 604 | ZTL | Photoperiod | 0.706666667 | 0.236666667 | 0.553333333 |
| AT5G57360.2 | i2_LQ_HNbud_c34260/f1p0/2347 | 79.47 | 604 | ZTL | Photoperiod | 5.456666667 | 2.216666667 | 1.963333333 |
| AT5G57360.2 | i2_HQ_HNbud_c22285/f3p0/2336 | 79.8 | 604 | ZTL | Photoperiod | 14.05666667 | 6.98 | 4.283333333 |
| AT5G57360.2 | i2_LQ_HNbud_c38566/f1p0/2432 | 79.8 | 604 | ZTL | Photoperiod | 6.283333333 | 1.466666667 | 0.82 |
| AT1G59940.1 | i0_LQ_HNbud_c5507/f1p2/826 | 78.79 | 132 | ARR3 | photoperiod, circadian clock | 0 | 0 | 0 |
| AT1G59940.1 | i1_LQ_HNbud_c148089/f1p1/1414 | 67.97 | 128 | ARR3 | photoperiod, circadian clock | 2.08 | 9.316666667 | 7.436666667 |
| AT1G59940.1 | i0_LQ_HNbud_c5156/f1p26/818 | 67.44 | 129 | ARR3 | photoperiod, circadian clock | 0.64 | 4.106666667 | 2.273333333 |
| AT1G59940.1 | i0_LQ_HNbud_c13679/f1p1/1014 | 64.91 | 171 | ARR3 | photoperiod, circadian clock | 2.65 | 10.22333333 | 8.74 |
| AT1G59940.1 | i0_LQ_HNbud_c74195/f1p0/920 | 64.91 | 171 | ARR3 | photoperiod, circadian clock | #N/A | #N/A | #N/A |
| AT1G59940.1 | i1_LQ_HNbud_c45974/f1p0/1064 | 62.96 | 135 | ARR3 | photoperiod, circadian clock | 0 | 0.49 | 0.57 |
| AT1G59940.1 | i1_LQ_HNbud_c280610/f1p2/1009 | 61.14 | 211 | ARR3 | photoperiod, circadian clock | 2.51 | 5.133333333 | 3.88 |
| AT1G59940.1 | i1_LQ_HNbud_c280155/f1p2/1005 | 60.87 | 207 | ARR3 | photoperiod, circadian clock | #N/A | #N/A | #N/A |
| AT1G59940.1 | i1_LQ_HNbud_c29305/f1p2/1061 | 60.87 | 207 | ARR3 | photoperiod, circadian clock | 0.563333333 | 1.56 | 1.406666667 |
| Os08t0157600_01 | i2_LQ_HNbud_c56566/f1p1/2291 | 78.5 | 107 | CCA1/LHY/OsCCA1 | photoperiod, circadian clock | 0 | 0 | 0 |
| Os08t0157600_01 | i2_LQ_HNbud_c24695/f1p0/2387 | 59.76 | 164 | CCA1/LHY/OsCCA1 | photoperiod, circadian clock | 0 | 0.18 | 0 |
| Os08t0157600_01 | i2_HQ_HNbud_c6478/f4p0/2107 | 58.76 | 177 | CCA1/LHY/OsCCA1 | photoperiod, circadian clock | #N/A | #N/A | #N/A |
| Os08t0157600_01 | i2_LQ_HNbud_c38854/f1p0/2616 | 58.76 | 177 | CCA1/LHY/OsCCA1 | photoperiod, circadian clock | 0.146666667 | 1.123333333 | 0.883333333 |
| Os08t0157600_01 | i2_LQ_HNbud_c7558/f1p0/2156 | 58.76 | 177 | CCA1/LHY/OsCCA1 | photoperiod, circadian clock | #N/A | #N/A | #N/A |
| AT5G37260.1 | i2_LQ_HNbud_c2323/f1p7/2321 | 61.21 | 165 | CIR1 | photoperiod, circadian clock | 1.793333333 | 3.75 | 3.076666667 |
| AT5G37260.1 | i2_LQ_HNbud_c24825/f1p1/2350 | 61.21 | 165 | CIR1 | photoperiod, circadian clock | 1.226666667 | 3.21 | 2.263333333 |
| AT5G37260.1 | i1_HQ_HNbud_c6488/f2p2/1435 | 50.52 | 194 | CIR1 | photoperiod, circadian clock | 0 | 0 | 0 |
| Os02t0573200_01 | i2_HQ_HNbud_c64689/f2p5/2434 | 67.37 | 708 | CRY1 | photoperiod, circadian clock | #N/A | #N/A | #N/A |
| Os02t0625000_01 | i2_LQ_HNbud_c72650/f1p2/2458 | 58.61 | 633 | CRY2 | photoperiod, circadian clock | 0 | 0.03 | 0 |
| Os02t0625000_01 | i2_LQ_HNbud_c2398/f1p4/2778 | 58.45 | 633 | CRY2 | photoperiod, circadian clock | 0 | 0 | 0 |
| AT2G40080.1 | i0_LQ_HNbud_c3797/f1p3/610 | 64.56 | 79 | ELF4 | photoperiod, circadian clock | #N/A | #N/A | #N/A |
| AT2G40080.1 | i2_LQ_HNbud_c43565/f1p0/2667 | 64.56 | 79 | ELF4 | photoperiod, circadian clock | 16.33666667 | 7.85 | 7.033333333 |
| AT2G40080.1 | i0_LQ_HNbud_c31232/f1p0/681 | 63.51 | 74 | ELF4 | photoperiod, circadian clock | #N/A | #N/A | #N/A |
| AT2G40080.1 | i0_LQ_HNbud_c42850/f1p0/838 | 63.51 | 74 | ELF4 | photoperiod, circadian clock | 14.26666667 | 7.943333333 | 5.833333333 |
| AT2G40080.1 | i1_LQ_HNbud_c178700/f1p1/1244 | 61.25 | 80 | ELF4 | photoperiod, circadian clock | 1.646666667 | 0.373333333 | 0.566666667 |
| AT2G40080.1 | i0_LQ_HNbud_c25344/f1p0/899 | 56.67 | 90 | ELF4 | photoperiod, circadian clock | 0 | 0.546666667 | 0.71 |
| AT2G40080.1 | i1_LQ_HNbud_c250464/f1p0/1140 | 56.67 | 90 | ELF4 | photoperiod, circadian clock | 0.893333333 | 0.846666667 | 1.483333333 |
| Os01t0971800_00 | i2_LQ_HNbud_c26680/f1p4/2394 | 51.14 | 88 | OsLUX | photoperiod, circadian clock | #N/A | #N/A | #N/A |
| Os01t0971800_00 | i2_LQ_HNbud_c5101/f1p2/2341 | 51.14 | 88 | OsLUX | photoperiod, circadian clock | 0.236666667 | 0.833333333 | 0.216666667 |
| Os01t0971800_00 | i2_LQ_HNbud_c52675/f1p2/2557 | 51.14 | 88 | OsLUX | photoperiod, circadian clock | 4.623333333 | 6.956666667 | 5.7 |
| Os03t0752100_01 | i2_LQ_HNbud_c25361/f1p0/2308 | 58.26 | 218 | OsPHYC | photoperiod, circadian clock | #N/A | #N/A | #N/A |
| Os03t0752100_01 | i2_LQ_HNbud_c40422/f1p0/2138 | 58.26 | 218 | OsPHYC | photoperiod, circadian clock | 0.623333333 | 2.746666667 | 2.186666667 |
| AT5G35840.1 | i3_LQ_HNbud_c13059/f1p9/3916 | 66.53 | 947 | PHYC | photoperiod, circadian clock | 0.776666667 | 1.686666667 | 1.636666667 |
| AT5G35840.1 | i4_HQ_HNbud_c127/f3p0/4837 | 65.14 | 1113 | PHYC | photoperiod, circadian clock | 5.136666667 | 5.36 | 6.526666667 |
| AT5G35840.1 | i4_HQ_HNbud_c194/f2p0/4876 | 65.14 | 1113 | PHYC | photoperiod, circadian clock | #N/A | #N/A | #N/A |
| AT5G35840.1 | i4_LQ_HNbud_c377/f1p0/4773 | 65.14 | 1113 | PHYC | photoperiod, circadian clock | 4.983333333 | 6.333333333 | 5.83 |
| AT5G35840.1 | i4_LQ_HNbud_c2080/f1p0/4256 | 64.96 | 1113 | PHYC | photoperiod, circadian clock | #N/A | #N/A | #N/A |
| AT5G35840.1 | i3_LQ_HNbud_c8657/f1p6/3914 | 64.59 | 1045 | PHYC | photoperiod, circadian clock | 3.656666667 | 2.083333333 | 1.426666667 |
| AT5G35840.1 | i4_LQ_HNbud_c2499/f1p9/4543 | 63.97 | 1113 | PHYC | photoperiod, circadian clock | 2.016666667 | 2.503333333 | 2.366666667 |
| AT5G35840.1 | i4_LQ_HNbud_c2229/f1p1/4981 | 63.07 | 1113 | PHYC | photoperiod, circadian clock | 0.76 | 0.193333333 | 0.593333333 |
| AT5G35840.1 | i4_LQ_HNbud_c1484/f1p5/4446 | 61.13 | 1114 | PHYC | photoperiod, circadian clock | #N/A | #N/A | #N/A |
| Os06t0603000_01 | i0_LQ_HNbud_c9061/f1p0/1000 | 68.97 | 232 | Se-5 | photoperiod, circadian clock, developmental process | #N/A | #N/A | #N/A |
| Os06t0603000_01 | i1_HQ_HNbud_c17802/f16p5/1253 | 68.97 | 232 | Se-5 | photoperiod, circadian clock, developmental process | #N/A | #N/A | #N/A |
| Os06t0603000_01 | i1_HQ_HNbud_c33513/f15p5/1333 | 68.97 | 232 | Se-5 | photoperiod, circadian clock, developmental process | 14.95666667 | 15.87 | 17.44 |
| Os06t0603000_01 | i1_LQ_HNbud_c131367/f1p5/1231 | 68.97 | 232 | Se-5 | photoperiod, circadian clock, developmental process | #N/A | #N/A | #N/A |
| Os06t0603000_01 | i1_LQ_HNbud_c131484/f1p5/1410 | 68.97 | 232 | Se-5 | photoperiod, circadian clock, developmental process | 13.82 | 16.73333333 | 17.90666667 |
| Os06t0603000_01 | i1_LQ_HNbud_c93094/f1p3/1248 | 68.97 | 232 | Se-5 | photoperiod, circadian clock, developmental process | #N/A | #N/A | #N/A |
| Os06t0603000_01 | i1_HQ_HNbud_c103482/f5p3/1325 | 58.38 | 173 | Se-5 | photoperiod, circadian clock, developmental process | 6.5 | 4.053333333 | 2.81 |
| Os06t0603000_01 | i1_LQ_HNbud_c109893/f2p2/1406 | 58.38 | 173 | Se-5 | photoperiod, circadian clock, developmental process | 2.396666667 | 2.27 | 1.4 |
| Os06t0603000_01 | i1_LQ_HNbud_c160595/f1p3/1393 | 57.8 | 173 | Se-5 | photoperiod, circadian clock, developmental process | 2.386666667 | 1.8 | 1.133333333 |
| Os06t0603000_01 | i1_LQ_HNbud_c70621/f1p3/1386 | 57.8 | 173 | Se-5 | photoperiod, circadian clock, developmental process | 2.706666667 | 1.703333333 | 1.353333333 |
| AT5G25810.1 | i0_LQ_HNbud_c12390/f1p0/979 | 77.22 | 79 | TNY | Photoperiod, Gibberellin | 9.51 | 6.246666667 | 4.246666667 |
| AT5G25810.1 | i0_LQ_HNbud_c40261/f1p2/877 | 68.42 | 95 | TNY | Photoperiod, Gibberellin | 0 | 0 | 0 |
| AT5G25810.1 | i1_LQ_HNbud_c278301/f1p1/1008 | 63.53 | 170 | TNY | Photoperiod, Gibberellin | 9.096666667 | 16.70666667 | 14.95666667 |
| AT5G25810.1 | i1_LQ_HNbud_c216821/f1p0/1134 | 58.03 | 193 | TNY | Photoperiod, Gibberellin | 2.286666667 | 3.75 | 2.843333333 |

**TableS4. Flowering gene model in Arabidopsis and rice.**

| Gene ID in Ath/Os | symbol |
| --- | --- |
| Os01t0111600-01 | *OsMFT2* |
| Os01t0129200-01 | *SL1* |
| Os01t0140700-00 | *OsRAV12* |
| Os01t0141000-01 | *OsRAV9* |
| Os01t0182600-01 | *OsGI* |
| Os01t0195000-01 | *OsIDD2* |
| Os01t0201700-01 | *OSMADS3* |
| Os01t0202700-00 | *OsFTL8* |
| Os01t0218500-02 | *OsFTL1/FT* |
| Os01t0229300-01 | *EMF1* |
| Os01t0264000-01 | *CDF1* |
| Os01t0269900-01 | *ARP6* |
| Os01t0277500-01 | *CDF2* |
| Os01t0286100-01 | *OsPIL15* |
| Os01t0566100-01 | *OsELF3* |
| Os01t0626400-01 | *WRKY transcription factor* |
| Os01t0693400-01 | *AP2/EREBP127* |
| Os01t0710000-01 | *FVE* |
| Os01t0713600-01 | *OsLFL1* |
| Os01t0725800-01 | *OsSPA1* |
| Os01t0726400-01 | *OsMADS32* |
| Os01t0748800-00 | *OsFTL9* |
| Os01t0789100-01 | *PGP1* |
| Os01t0813100-00 | *FD* |
| Os01t0813300-00 | *AP2D10* |
| Os01t0834400-01 | *HAP3B* |
| Os01t0835700-01 | *none* |
| Os01t0859300-01 | DPBF2 |
| Os01t0867300-01 | *GBF4* |
| Os01t0883100-01 | *OsMADS2(PI-like)* |
| Os01t0907400-01 | *OsREF6* |
| Os01t0909200-01 | *OsDCL3a* |
| Os01t0922600-01 | *OsSPL4/2* |
| Os01t0922800-01 | *OsMADS51* |
| Os01t0934300-02 | *LD* |
| Os01t0935000-01 | *OsIDD9* |
| Os01t0949400-01 | *Se-13* |
| Os01t0951000-02 | *FY* |
| Os01t0971800-00 | *OsLUX* |
| Os02t0104100-00 | *AGL14* |
| Os02t0133800-01 | *OsPAF* |
| Os02t0139200-01 | *SKB1* |
| Os02t0150800-01 | *OsZTLb* |
| Os02t0152500-01 | *VIN3* |
| Os02t0170500-01 | *HAP5A* |
| Os02t0232300-00 | *OsFTL13* |
| Os02t0531600-01 | *OsRCN2* |
| Os02t0554000-01 | *EFS* |
| Os02t0579600-00 | *MADS27* |
| Os02t0610500-01 | *COL5* |
| Os02t0618200-01 | *OsTOC1* |
| Os02t0625000-01 | *CRY2* |
| Os02t0638650-01 | *AP2* |
| Os02t0657000-01 | *AP2* |
| Os02t0689800-01 | *OsPIE1* |
| Os02t0696900-00 | *MYB* |
| Os02t0731200-01 | *OsMADS57* |
| Os02t0761000-01 | *OsMADS22* |
| Os02t0766700-01 | *ABF4* |
| Os02t0767800-00 | *AP2* |
| Os02t0771100-01 | *COP1* |
| Os02t0776700-01 | *Similar to Single myb histone 6* |
| Os02t0776900-02 | *OsGRF1* |
| Os02t0833600-01 | *ABF3* |
| Os03t0112700-01 | *EHD4* |
| Os03t0121800-00 | *OsDCL1* |
| Os03t0122600-01 | *OsMADS50* |
| Os03t0151300-01 | *ELF6* |
| Os03t0151400-01 | *OsELF6* |
| Os03t0169600-01 | *CDF3（fragment）* |
| Os03t0186600-01 | *OsMDP1* |
| Os03t0193225-01 | *OsFRI* |
| Os03t0207300-01 | *CKA2/DTH3/ATCKA1/ATCKA2/CKA4/CPCK2/ALPHA/CHAIN 3/CKA3/DTH3* |
| Os03t0243300-01 | *RPN10* |
| Os03t0251350-00 | *HAP5C* |
| Os03t0284100-01 | *OsPRR73* |
| Os03t0307800-01 | *OsSWN* |
| Os03t0309200-01 | *OsPHYB* |
| Os03t0313100-01 | *AP2* |
| Os03t0322700-00 | *AREB3* |
| Os03t0410300-01 | *DUF1313 family,ELF4?* |
| Os03t0413000-01 | *HAP3B* |
| Os03t0588200-01 | *OsFRI* |
| Os03t0588800-01 | *OsFRI/Hypothetical conserved gene* |
| Os03t0640100-01 | ATMSI1 |
| Os03t0710800-01 | *OsGF14f* |
| Os03t0719800-01 | *OsPHYA* |
| Os03t0752100-01 | *OsPHYC* |
| Os03t0752800-02 | *OsMADS14/AP1* |
| Os03t0763000-01 | *CKA2* |
| Os03t0770700-01 | *AP2* |
| Os03t0793500-01 | *Hd16/ EL1/CK1e* |
| Os03t0798500-00 | *OsWRKY6* |
| Os03t0818800-00 | *OsIDS1* |
| Os03t0833300-04 | *OsSPL6* |
| Os03t0833300-02 | *OsSPL8/6* |
| Os03t0851400-01 | *OsFRI/Hypothetical conserved gene* |
| Os04t0162100-01 | *EMF2* |
| Os04t0411400-01 | *OsRCN4* |
| Os02t0573200-01 | *CRY1* |
| Os04t0473800-01 | *FCP1* |
| Os04t0488400-01 | *OsFTL5* |
| Os04t0496400-01 | *ELF9* |
| Os04t0497700-01 | *COL4* |
| Os04t0509300-02 | *OsDCL4* |
| Os04t0598300-00 | *RFL* |
| Os04t0614100-00 | *OsMADS31* |
| Os04t0649100-03 | *SNZ* |
| Os04t0690800-01 | *psbS2* |
| Os05t0121600-01 | *TOE1* |
| Os05t0137500-00 | *OsWRKY5* |
| Os05t0145400-01 | *VEL1* |
| Os05t0182800-01 | *OVA?* |
| Os05t0203800-01 | *OsMADS58* |
| Os05t0269500-01 | *HAP2* |
| Os05t0408200-01 | *OsSPL9* |
| Os05t0437700-01 | *AREB3* |
| Os05t0463800-01 | *HAP3B* |
| Os05t0489700-01 | *FDP* |
| Os05t0497300-01 | *AP2/ERF* |
| Os05t0518000-00 | *OsFTL10* |
| Os05t0549800-01 | *FLC* |
| Os05t0571000-01 | *SPA2* |
| Os05t0573500-00 | *HAP3B* |
| Os06t0108500-01 | *AGL14* |
| Os06t0157700-01 | *Hd3a* |
| Os06t0157500-01 | *RFT1* |
| Os06t0160400-01 | *HGW* |
| Os06t0162800-01 | *OsMADS5* |
| Os06t0199402-00 | *none* |
| Os06t0199500-01 | *OsHAL3* |
| Os06t0211200-01 | *ABF1* |
| Os06t0217300-01 | *OsMADS55* |
| Os06t0256500-01 | *G6PIB/PGI/GPI* |
| Os06t0275500-01 | *OsCLF* |
| Os06t0498800-01 | *OsMFT1* |
| Os06t0552900-00 | *OsFTL12* |
| Os06t0603000-01 | *Se-5* |
| Os06t0639200-01 | *TOE3* |
| Os02t0178100-01 | *CO6* |
| Os06t0654900-01 | *CO6* |
| Os06t0667100-01 | *HAP5B* |
| Os06t0694000-01 | *OsZTLa* |
| Os06t0712700-01 | *OsMADS16* |
| Os06t0717200-01 | *FON1* |
| Os06t0720900-01 | *ABF3* |
| Os06t0724000-01 | *ABF3* |
| Os07t0108900-01 | *OsMADS15* |
| Os07t0108900-02 | *OsMADS15* |
| Os07t0158500-00 | *HAP2* |
| Os07t0235800-01 | *TOE2* |
| Os07t0261200-01 | *OsGhd7* |
| Os07t0445600-01 | *CIP7-like* |
| Os07t0445600-02 | *CIP7-like* |
| Os07t0605200-01 | *OsMADS18* |
| Os07t0606600-01 | *HAP3A* |
| Os07t0616000-01 | *CIP7-like* |
| Os07t0669500-01 | *FZP* |
| Os07t0685000-02 | *CDF3（fragment）* |
| Os07t0686100-01 | *AREB3* |
| Os07t0695100-01 | *OsPRR37* |
| Os08t0100500-01 | *HUA2* |
| Os08t0105000-01 | *none* |
| Os08t0112700-01 | *OsMADS26* |
| Os08t0137100-01 | *OsFIE1a* |
| Os08t0137250-01 | *OsFIE1b* |
| Os08t0157600-01 | *CCA1/LHY/OsCCA1* |
| Os08t0174500-02 | *DTH8* |
| Os08t0174500-01 | *Ghd8* |
| Os08t0220600-01 | *VEL2* |
| Os08t0249000-01 | *COL5* |
| Os08t0366200-01 | *DUF1313 family,ELF4?* |
| Os08t0408500-01 | *DBF1/ERF* |
| Os08t0430500-01 | *OsGF14c* |
| Os08t0471950-00 | *ABF2* |
| Os08t0480800-01 | *OsGF14a* |
| Os08t0496500-01 | *HAP5C* |
| Os08t0509600-01 | *OsSPL14* |
| Os08t0513700-01 | *OsSPL15* |
| Os08t0531700-01 | *OsMADS7* |
| Os08t0531900-01 | *OsMADS37* |
| Os08t0549600-00 | *ABF3* |
| Os08t0559300-01 | *OsSPY* |
| Os09t0123200-01 | *OsFCA* |
| Os09t0134500-02 | *ATX1* |
| Os09t0240200-01 | *OsCO3* |
| Os09t0306700-01 | *OsPFT1* |
| Os09t0306800-01 | *VRN2* |
| Os09t0307800-01 | *SDG724, LVP1, OsSET34* |
| Os09t0346500-04 | *OsCAB1R* |
| Os09t0456200-01 | *ABI5* |
| Os09t0480700-01 | *HAP5C* |
| Os09t0507200-01 | *OsMADS8* |
| Os09t0513500-00 | *OsFTL4* |
| Os09t0516300-01 | *FPA* |
| Os09t0532400-01 | *OsPRR95* |
| Os09t0540800-01 | *AtbZIP15* |
| Os09t0560900-01 | *OsSUF4* |
| Os10t0324900-01 | *OsLHP1* |
| Os10t0406300-01 | *CDF3（fragment）* |
| Os10t0419200-01 | *RID1* |
| Os10t0463400-01 | *Ehd1* |
| Os10t0536100-01 | *OsMADS56* |
| Os10t0560400-01 | *OsP* |
| Os10t0564000-01 | *OsPEP* |
| AT4G26000.1Os10t0564000-01 | PEP |
| AT1G55250.1Os10t0565600-01 | *HUB2* |
| Os10t0565600-01 | *OsHUB2* |
| Os10t0577600-01 | *JMJ706* |
| Os12t0152000-00 | *OsRCN3/OsTFL1* |
| Os11t0152500-00 | *OsTFL1* |
| Os11t0157600-01 | *OsPRR59* |
| Os11t0250000-01 | *RBS1* |
| Os11t0293800-00 | *OsFTL11* |
| Os11t0545600-01 | *MRG702* |
| Os11t0545600-03 |  |
| Os11t0546900-01 | *OsGF14d* |
| Os11t0547000-01 | *OsFKF1* |
| Os11t0621500-01 | *ELF4((Fragment))* |
| Os11t0684000-01 | *MYB21/3* |
| Os12t0175400-01 | *Similar to OSMYB2* |
| Os12t0207000-01 | *OsMADS13* |
| Os12t0232300-00 | *OsFTL7* |
| Os12t0501700-00 | *OsMADS20* |
| Os12t0533500-01 | *OsVIL1, OsVIL2* |
| Os12t0618600-01 | *VRN5* |
| AT5G24930.1 | ATCOL4 |
| AT5G25810.1 | *TNY* |
| AT5G25900.1 | *GA3* |
| AT5G27220.1 | Frigida-like protein |
| AT5G27230.1 | *AT5G27230* |
| AT5G27320.1 | *GAr1 /2/3* |
| AT3G05120.1 | *GAr2* |
| AT3G63010.1 | *GAr3* |
| AT5G28450.1 | *AT5G28450* |
| AT5G28490.1 | *LSH1* |
| AT3G04510.1 | *LSH2* |
| AT5G35840.1 | *PHYC* |
| AT5G37055.1 | *SEF* |
| AT5G37260.1 | *CIR1* |
| AT5G37770.1 | *CML24* |
| AT5G37780.1 | *CAM1* |
| AT5G38150.1 | *PMI15* |
| AT5G38480.1 | *none* |
| AT1G57820.1 | *ORTH2* |
| AT5G39550.1 | *VIM3* |
| AT5G39660.1 | *CDF2* |
| AT5G39860.1 | *BNQ1* |
| AT5G15160.1 | *BNQ2* |
| AT3G47710.1 | *BNQ3* |
| AT5G41360.1 | *XPB2* |
| AT5G41790.1 | *CIP1* |
| AT5G42400.1 | *ATXR7* |
| AT5G42910.1 | *bZIP* |
| AT5G44080.1 | *bZIP* |
| AT5G45830.1 | *DOG1* |
| AT5G46210.1 | *CUL4* |
| AT5G46910.1 | *AT5G46910* |
| AT5G47010.1 | *LBA1* |
| AT5G47640.1 | *NFYB2* |
| AT5G48150.1 | *PAT1* |
| AT5G48300.1 | *ADG1* |
| AT5G49020.1 | *PRMT4a* |
| AT3G06930.1 | *PRMT4B* |
| AT5G51230.1 | *EMF2* |
| AT4G25420.1 | *GA20OX1* |
| AT5G51810.1 | *GA20OX2* |
| AT1G60980.1 | *GA20OX4* |
| AT5G07200.1 | *YAP169* |
| AT5G51820.1 | *PGM* |
| AT5G54510.1 | *DFL1* |
| AT5G57360.2 | *ZTL* |
| AT5G57380.1 | *VIN3* |
| AT5G57660.1 | *COL5* |
| AT5G59560.1 | *SRR1* |
| AT5G59710.1 | *VIP2* |
| AT5G59820.1 | *ZAT12* |
| AT5G59845.1 | *AT5G59845* |
| AT5G60100.1 | *PRR3* |
| AT5G60120.1 | *TOE2* |
| AT5G60910.1 | *FUL* |
| AT5G61150.1 | *VIP4* |
| AT5G61850.1 | *LFY* |
| AT5G62040.1 | *BFT* |
| AT5G62430.1 | *CDF1* |
| AT5G62560.1 | *K19B1.17* |
| AT5G62640.1 | *ELF5* |
| AT5G63980.1 | *FRY1* |
| AT5G64813.1 | *LIP1* |
| AT5G65050.1 | *MAF2* |
| AT5G65060.1 | *MAF3* |
| AT5G65070.1 | *MAF4* |
| AT5G65080.1 | *MAF5* |
| AT5G65430.1 | ATMIN10 |
| AT5G65540.1 | *AT5G65540* |
| AT5G67100.1 | *ICU2* |
| AT5G67180.1 | *EAT* |
| AT1G01040.1 | *SIN1* |
| AT1G01640.1 | *T1N6_2* |
| AT1G47990.1 | *GA2OX4/GA2OX6* |
| AT1G02400.1 | *GA2OX6* |
| AT1G03970.1 | GBF4 |
| AT1G04010.1 | *PSAT1* |
| AT1G04440.1 | *CKL13* |
| AT1G06040.1 | *STO* |
| AT1G07980.1 | *CBFs* |
| AT1G08970.1 | *HAP5C* |
| AT1G54830.1 | *NFYC9(NF-YC3)* |
| AT1G56170.1 | *NFYC2/HAP5B* |
| AT1G09530.1 | *PIF3* |
| AT3G62090.1 | *PIL2* |
| AT1G09700.1 | *DRB1* |
| AT1G60220.1 | *OTS1* |
| AT1G10570.1 | *OTS2* |
| AT1G10588.1 | *AT1G10588* |
| AT2G14900.1 | *T26I20.6* |
| AT2G39540.1 | *F12L6.20* |
| AT1G12190.1 | *T28K15.8* |
| AT1G12610.1 | *DDF1* |
| AT1G63030.1 | *DDF2* |
| AT4G25470.1 | *CBF2* |
| AT4G25480.1 | *CBF3* |
| AT4G25490.1 | *CBF1* |
| AT1G12910.1 | *LWD1/ATAN11* |
| AT3G26640.1 | *LWD2* |
| AT1G13260.1 | *RAV1* |
| AT1G25560.1 | *EDF1* |
| AT1G68840.1 | *TEM2* |
| AT1G51120.1 | AP2/B3 transcription |
| AT1G50680.1 | AP2/B3 transcription |
| AT3G25730.1 | EDF3 |
| AT1G14400.1 | *UBC1* |
| AT1G14920.1 | *GAI /RGA/RGL1/RGL2* |
| AT2G01570.1 | *RGA* |
| AT1G66350.1 | *RGL1* |
| AT3G03450.1 | *RGL2* |
| AT1G15550.1 | *GA3OX1/2/3/4* |
| AT1G80340.1 | *GA3ox2* |
| AT4G21690.1 | *GA3OX3* |
| AT1G80330.1 | *GA3OX4* |
| AT1G17455.1 | ELF4-L4 |
| AT1G72630.1 | ELF4-L2 |
| AT1G17760.1 | *ATCSTF77* |
| AT1G18100.1 | *MFT* |
| AT1G18450.1 | *ARP4* |
| AT4G09610.1 | *GASA2* |
| AT1G22690.1 | *T22J18.14* |
| AT1G24260.1 | *SEP3* |
| AT3G02310.1 | *SEPALLATA2* |
| AT1G26310.1 | *CAL* |
| AT1G69120.1 | *AP1* |
| AT1G26790.1 | Dof-type zinc finger DNA-binding family protein |
| AT1G69570.1 | *CDF5* |
| AT3G47500.1 | *CDF3* |
| AT1G26830.1 | *CUL3* |
| AT1G29160.1 | COG1 |
| AT2G34140.1 | CDF4 |
| AT1G78440.1 | *GA2OX1/2/3* |
| AT1G30040.1 | *GA2OX2* |
| AT2G34555.1 | *GA2OX3* |
| AT1G30950.1 | *UFO* |
| AT1G30960.1 | *F17F8.15* |
| AT1G35160.1 | GF14 |
| AT1G78300.1 | 14-3-3OMEGA |
| AT4G09000.1 | GRF1 |
| AT3G02520.1 | general regulatory factor 7 |
| AT5G16050.1 | general regulatory factor 5 |
| AT1G43700.1 | *VIP1* |
| AT1G44090.1 | *GA20OX5* |
| AT1G44446.1 | *CH1* |
| AT1G45249.1 | abscisic acid responsive elements-binding factor 2 |
| AT1G49720.1 | abscisic acid responsive elements-binding factor 2 |
| AT3G19290.1 | ABRE binding factor 4 |
| AT4G34000.1 | abscisic acid responsive elements-binding factor 3 |
| AT1G49480.1 | *RTV1* |
| AT3G18990.1 | *VRN1* |
| AT1G50960.1 | *GA2ox7* |
| AT1G52800.1 | *F14G24.7* |
| AT1G53160.1 | *SPL4* |
| AT3G15270.1 | *SPL5* |
| AT1G55080.1 | *MED9* |
| AT1G59940.1 | *ARR3* |
| AT1G61040.1 | *VIP5* |
| AT1G62750.1 | *SCO1* |
| AT5G20240.1 | *PI* |
| AT4G22140.1 | *EBS* |
| AT4G22950.1 | *AGL19* |
| AT4G11880.1 | *AGL14* |
| AT2G45660.1 | *SOC1* |
| AT4G23340.1 | *AT4G23340* |
| AT4G24210.1 | *SLY1* |
| AT4G24540.1 | *AGL24* |
| AT2G22540.1 | *SVP* |
| AT4G25530.1 | *FWA* |
| AT4G27060.1 | *TOR1* |
| AT4G29130.1 | *HXK1* |
| AT4G29830.1 | *VIP3* |
| AT4G30200.2 | *VEL1* |
| AT2G18880.1 | *none* |
| AT5G10625.1 | *AT5G10625* |
| AT4G31380.1 | *FLP1* |
| AT5G24860.1 | *FPF1* |
| AT4G31500.1 | *ATR4* |
| AT4G32040.1 | *KNAT5* |
| AT4G32980.1 | *ATH1* |
| AT4G33280.1 | *AT4G33280* |
| AT4G34530.1 | *CIB1* |
| AT4G35900.1 | *FD* |
| AT2G17770.1 | *FDP* |
| AT4G36920.1 | *AP2* |
| AT4G37580.1 | *COP3* |
| AT4G37880.1 | *T28I19.160* |
| AT4G37940.1 | *AGL21* |
| AT2G22630.1 | *AGL17* |
| AT3G57230.1 | *AGL16* |
| AT5G01040.1 | *LAC8* |
| AT5G02030.1 | *PNY* |
| AT5G02200.1 | *FHL* |
| AT5G02810.1 | *PRR7* |
| AT5G02840.1 | *LCL1* |
| AT3G09600.1 | *RVE8* |
| AT5G03790.1 | *LMI1* |
| AT5G03840.1 | *TFL1* |
| AT5G05690.1 | *CPD* |
| AT5G06100.1 | *MYB33/65* |
| AT3G11440.1 | *MYB65* |
| AT5G08330.1 | *TCP11* |
| AT5G10140.1 | *FLC* |
| AT1G77080.2 | *FLM/MAF1* |
| AT5G10450.1 | *none* |
| AT5G11260.1 | *HY5* |
| AT5G11530.1 | *EMF1* |
| AT5G12840.1 | *NFYA1/HAP2A* |
| AT5G13790.1 | *AGL15* |
| AT5G14920.1 | *AT5G14920* |
| AT3G10185.1 | *AT3G10185* |
| AT2G30810.1 | *F7F1.2* |
| AT5G15230.1 | *GASA4* |
| AT3G02885.1 | *GASA5* |
| AT1G74670.1 | *GASA6* |
| AT5G15840.1 | *CO* |
| AT5G15850.1 | *COL1* |
| AT3G02380.1 | *COL2* |
| Os06t0275000-01 | *Hd1* |
| AT5G15960.1 | *KIN1* |
| AT5G15970.1 | *KIN2* |
| AT5G16320.1 | *FRL1* |
| AT1G31814.1 | *FRL2* |
| AT5G19550.1 | *AAT2* |
| AT5G20730.1 | *NPH4* |
| AT5G23150.1 | *HUA2* |
| AT5G23260.1 | *none* |
| AT5G24470.1 | *APRR5* |
| AT2G46790.1 | *PRR9* |
| AT3G57920.1 | *SPL15* |
| AT2G42200.1 | *SPL9* |
| AT3G21320.1 | *AT3G21320* |
| AT3G22380.1 | *TIC* |
| AT3G24440.1 | *VIN3-L* |
| AT3G26120.1 | *TEL1* |
| AT3G26744.1 | *ICE1* |
| AT3G26790.1 | *FUS3* |
| AT3G30180.1 | *BR6OX2* |
| AT3G30260.1 | *none* |
| AT3G49600.1 | *UBP26* |
| AT3G53340.1 | *none* |
| AT3G54340.1 | *AP3* |
| AT3G54720.1 | *AMP1* |
| AT3G54990.1 | *SMZ* |
| AT2G39250.1 | *SNZ* |
| AT3G56850.1 | AREB3 |
| AT3G57300.1 | *INO80* |
| AT3G57390.1 | *AGL18* |
| AT3G58780.1 | *SHP1* |
| AT3G59060.1 | *PIL6* |
| AT5G47080.1 | *CKB1* |
| AT4G17640.1 | *CKB2* |
| AT3G60250.1 | *CKB3* |
| AT2G44680.1 | *CKB4* |
| AT3G60870.1 | *AHL18* |
| AT4G00450.1 | *CRP* |
| AT4G00650.1 | *FRI* |
| AT4G00690.1 | *ULP1B* |
| AT4G02020.1 | *SDG10* |
| AT4G02780.1 | *GA1* |
| AT4G03400.1 | *DFL2* |
| AT4G04885.1 | *PCFS4* |
| AT4G04890.1 | *PDF2* |
| AT4G09610 | *GASA2* |
| AT4G12480.1 | *pEARLI* |
| AT4G14110.1 | *COP9* |
| AT4G14540.1 | *NFYB3/HAP3C* |
| AT4G14690.1 | *ELIP2* |
| AT4G15090.1 | *FAR1* |
| AT4G15880.1 | *ESD4* |
| AT4G16280.1 | *FCA* |
| AT4G16780.1 | *HAT4* |
| AT4G16810.1 | *AT4G16810* |
| AT4G16845.1 | *VRN2* |
| AT4G17060.1 | *FIP2* |
| AT2G18790.1 | *PHYB* |
| AT4G16250.1 | *PHYD* |
| AT4G18130.1 | *PHYE* |
| AT4G20400.1 | *JMJ14* |
| AT2G34880.1 | JMJ15 |
| AT4G21200.1 | *GA2OX8* |
| AT2G25930.1 | *ELF3* |
| AT2G24790.1 | *COL3* |
| AT2G06255.1 | ELF4-L3 |
| AT1G62830.1 | *LSD1* |
| AT4G20370.1 | *TSF* |
| AT1G65480.1 | *FT* |
| AT1G69490.1 | *NAP* |
| AT1G69935.1 | *SHW1* |
| AT1G70170.1 | *MMP* |
| AT1G71692.1 | *XAL1* |
| AT1G71800.1 | *VRN2* |
| AT1G72050.1 | *CSTF64* |
| AT1G72830.1 | *HAP2C* |
| AT1G74660.1 | *MIF1* |
| AT1G79280.1 | *NUA* |
| AT1G79460.1 | *GA2/KS* |
| AT1G79730.1 | *ELF7* |
| AT1G80680.1 | *PRE* |
| AT2G01290.1 | *RPI2* |
| AT2G03710.1 | *SEPALLATA4* |
| AT2G04030.1 | *HSP88.1* |
| AT2G06005.1 | *FIP1* |
| AT2G06210.1 | *ELF8* |
| AT2G13540.1 | *ENS* |
| AT2G13570.1 | NF-YB7 |
| AT2G18870.1 | VEL3 |
| AT2G18915.2 | *LKP2* |
| AT2G20180.1 | *PIL5* |
| AT2G21070.1 | *FIO1* |
| AT2G21660.1 | *CCR2* |
| AT2G26710.1 | *CYP72B1* |
| AT2G27550.1 | *ATC* |
| AT2G27990.1 | *PNF* |
| AT2G28550.1 | *TOE1* |
| AT2G29950.1 | *ELF4-L1* |
| AT2G33810.1 | *SPL3* |
| AT2G33835.1 | *FES1* |
| AT2G34720.1 | *NFYA4* |
| AT2G35670.1 | FIE2, FIS2 |
| AT2G37060.1 | NF-YB8 |
| AT2G37678.1 | *FHY1* |
| AT2G38880.1 | *NFYB1* |
| AT2G39810.1 | *HOS1* |
| AT2G40080.1 | *ELF4* |
| AT2G41070.1 | *bZIP* |
| AT2G42530.1 | *COR15b* |
| AT2G42540.1 | *COR15a* |
| AT2G42830.1 | *SHP2* |
| AT2G43010.1 | *PIF4* |
| AT2G44950.1 | *HUB1* |
| AT3G61120.1 | *AGL13* |
| AT2G45650.1 | *AGL6* |
| Os04t0580700-01 | *OsMADS17* |
| AT2G45880.1 | BAM7 |
| AT2G46020.1 | *ATBRM* |
| AT2G47310.1 | *protein_coding* |
| AT2G47700.1 | *RFI2* |
| AT3G01460.1 | *MBD9* |
| AT3G03090.1 | *ATVGT1* |
| AT3G04240.1 | *SEC* |
| Os03t0627500-01 | *OsFLKa* |
| AT3G04680.1 | *CLPS3* |
| AT3G04910.1 | *WNK1* |
| AT3G05040.1 | *hst* |
| AT3G05690.1 | *HAP2B* |
| AT3G06910.1 | *ELS1* |
| AT3G07650.1 | *COL9* |
| Os04t0560300-01 | *OsFLD* |
| AT3G13682.1 | *LDL2* |
| AT3G15850.1 | *FADB* |
| AT3G16640.1 | *TCTP* |
| AT3G18030.1 | ATHAL3 |
| AT3G19040.1 | *HAF2* |
| AT3G19980.1 | *ATFYPP3* |
| AT3G20550.1 | *DDL* |
| AT3G20740.1 | *FIS3* |
| AT1G01060.1 | LHY |
| At1g04400.1 | CRY2 |
| At1g09570.1 | PHYA |
| At1g18090.1 | MFT |
| At1g22770.1 | GI |
| At1g25540.1 | PFT1 |
| At1g68050.1 | FKF1 |
| AT1G68210.1 | APRR6 |
| AT1G77080.1 | FLM/MAF1 |
| AT1G77300.1 | EFS |
| At2g19520.1 | FVE |
| AT2G43410.1 | FPA |
| AT2G46830.1 | CCA1 |
| At3g04610.1 | FLK |
| AT3G10390.1 | FLD |
| At3g11540.1 | SPY |
| At3g12810.1 | PIE1 |
| AT4G00760.1 | APRR8 |
| At4g02560.1 | LD |
| At4g08920.1 | CRY1 |
| AT4G18020.1 | APRR2 |
| AT5G04240.1 | ELF6 |
| AT5G04275.1 | MIR172(EAT) |
| AT5G13480.1 | FY |
| At5g17690.1 | TFL2 |
| AT5G49240.1 | APRR4 |
| AT5G57360.1 | ZTL |
| AT5G61380.1 | TOC1 |
| AT1G04400.2 | *CRY2* |
| AT1G30970.1 | *SUF4* |
| AT1G47250.1 | *none* |
| AT1G53090.1 | *SPA4* |
| AT1G55250.1 | *HUB2* |
| AT2G23070.1 | *none* |
| AT2G23080.1 | *none* |
| AT2G23380.1 | *CLF* |
| AT2G31650.1 | *ATX1* |
| AT2G32950.1 | *COP1* |
| AT2G36270.1 | *none* |
| AT2G46340.1 | *SPA1* |
| AT3G15354.1 | *SPA3* |
| AT3G33520.1 | *ARP6* |
| AT3G44460.1 | *none* |
| AT3G46640.1 | *LUX/PCL1* |
| AT3G48430.1 | *REF6* |
| AT3G48590.1 | *NFYC1/HAP5A* |
| AT3G50000.1 | *none* |
| AT4G11110.1 | *SPA2* |
| AT4G26000.1 | *none* |
| AT4G27430.1 | *CIP7* |
| AT4G31120.1 | *SKB1* |
| AT5G16260.1 | *ELF9* |
| AT5G42790.1 | *none* |
| AT5G58230.1 | *none* |
| AT5G59570.1 | *BOA* |
| AT5G63470.1 | *none* |
| AT5G67380.1 | *none* |
| Os02t580300-01 | *none* |
| Os03t0762000-01 | *DTH3* |
| Os04t0452100-01 | *OsCRY1b* |
| Os04t0462500-01 | *OsGF14b* |
| Os06t0142600-01 | *OsELF3* |
| Os07t0114400-01 | *CKA2* |

**TableS5. primer information.**

| Gene Name | | Gene ID |  | | | Primer Sequence | | Restriction Site |
| --- | --- | --- | --- | --- | --- | --- | --- | --- |
| *PlCO* | | i1_LQ_HNbud_c108459/f1p9/1496 | F | | | CGCCTGCAGATGGGTAGAGAAGAGGGGAGCG | | PstI |
|  |  |  | R | | | CGCACTAGTAAACGATGGAACGATGCCGTATCC | | SpeI |
| TUBULIN | i1_HQ_HNbud_c265122/f127p32/1731 | | | F | GATGTGTGCTGCTGATCCTCGACAC | |  |  |
|  |  |  |  | R | CTCCTGGATCGAAGTCGAGTTGCC | |  |  |
| ARR-A | i0_HQ_HNbud_c26374/f3p3/625 | | | F | CGGTAATGGCGATGAACCCCAT | |  |  |
|  |  |  |  | R | CGAAGCATTCGGCGTCAAGCAG | |  |  |
| AHK | i2_LQ_HNbud_c15767/f1p0/2099 | | | F | GCTCAAAGAGCTTAGCCAAACTGGC | |  |  |
|  |  |  |  | R | TCCGCCTCTTGCCAATTCCAGTG | |  |  |
| ABF | i2_HQ_HNbud_c13957/f3p3/2508 | | | F | GTGAAAGCAGGGATAGTGAGGGAGG | |  |  |
|  |  |  |  | R | GTGGTGGTTGTGGAGCAGACCTGAC | |  |  |
| AUX1 | i1_HQ_HNbud_c1356/f28p9/1925 | | | F | CGGTGGTTCTGCTTATGATGCATGG | |  |  |
|  |  |  |  | R | GCCAGAAAGCATTCCCAGCTGAG | |  |  |
| BSK | i1_HQ_HNbud_c8371/f4p4/1684 | | | F | GGTGAAGCTTGCTTGCGAATGG | |  |  |
|  |  |  |  | R | TGGCAGTAGTGAAATCTTTGGCTCG | |  |  |
| CYCD | i1_HQ_HNbud_c106733/f4p6/1482 | | | F | GGAGGAGACCCAAGTCCCTCTTC | |  |  |
|  |  |  |  | R | GCAAGTTGGTCTTGAATCCAAGCC | |  |  |
| GH3 | i2_LQ_HNbud_c62348/f1p0/2014 | | | F | GGCTAGAGCCGAACTAGTCGATTTC | |  |  |
|  |  |  |  | R | GTCCATTTCCCGGCAGCACTC | |  |  |
| IAA | i1_HQ_HNbud_c264029/f4p5/1574 | | | F | CAGCGAAGGACCTTCCTTCTGG | |  |  |
|  |  |  |  | R | GCTACAGAATCGGCACTTCTCTTGG | |  |  |
| PIF | i2_LQ_HNbud_c24587/f1p3/2148 | | | F | CAGAAGATGATGTGGATGGGAAATGG | |  |  |
|  |  |  |  | R | GCCTGGTTTGGTGTGGGTGTC | |  |  |
| PYL | i0_LQ_HNbud_c70302/f1p11/972 | | | F | TCGATCCATCGACGCACCAC | |  |  |
|  |  |  |  | R | CAAGCCGGACATGACCATCAC | |  |  |
| SAUR | i0_LQ_HNbud_c3262/f1p1/598 | | | F | GGTGTCCGCTTCCCTTCCATG | |  |  |
|  |  |  |  | R | TCTTCCTCTGCTCTTGCAAGTAAGCC | |  |  |
| SnRK2 | i1_HQ_HNbud_c3738/f3p4/1416 | | | F | GCAGTGCTGGTCGGTTTAGTGAAG | |  |  |
|  |  |  |  | R | GGTTGTGAGTGCAACAAAGCAGAC | |  |  |
